# p53 modeling as a route to mesothelioma patients stratification and novel therapeutic identification

**DOI:** 10.1186/s12967-018-1650-0

**Published:** 2018-10-13

**Authors:** Kun Tian, Emyr Bakker, Michelle Hussain, Alice Guazzelli, Hasen Alhebshi, Parisa Meysami, Constantinos Demonacos, Jean-Marc Schwartz, Luciano Mutti, Marija Krstic-Demonacos

**Affiliations:** 1 School of Environment and Life Sciences, University of Salford, Salford, UK; 2 School of Medicine, University of Central Lancashire, Preston, UK; 3 School of Medicine, University of Cardiff, Cardiff, UK; 4 Faculty of Biology, Medicine and Health, University of Manchester, Manchester, UK; 5 Sbarro Institute for Cancer Research and Molecular Medicine, Center for Biotechnology, College of Science and Technology, Temple University, Philadelphia, PA 19122 USA

**Keywords:** Drug repositioning, Bioinformatics, Mesothelioma, TP53, Personalized medicine

## Abstract

**Background:**

Malignant pleural mesothelioma (MPM) is an orphan disease that is difficult to treat using traditional chemotherapy, an approach which has been effective in other types of cancer. Most chemotherapeutics cause DNA damage leading to cell death. Recent discoveries have highlighted a potential role for the p53 tumor suppressor in this disease. Given the pivotal role of p53 in the DNA damage response, here we investigated the predictive power of the p53 interactome model for MPM patients’ stratification.

**Methods:**

We used bioinformatics approaches including omics type analysis of data from MPM cells and from MPM patients in order to predict which pathways are crucial for patients’ survival. Analysis of the PKT206 model of the p53 network was validated by microarrays from the Mero-14 MPM cell line and RNA-seq data from 71 MPM patients, whilst statistical analysis was used to identify the deregulated pathways and predict therapeutic schemes by linking the affected pathway with the patients’ clinical state.

**Results:**

In silico simulations demonstrated successful predictions ranging from 52 to 85% depending on the drug, algorithm or sample used for validation. Clinical outcomes of individual patients stratified in three groups and simulation comparisons identified 30 genes that correlated with survival. In patients carrying wild-type p53 either treated or not treated with chemotherapy, FEN1 and MMP2 exhibited the highest inverse correlation, whereas in untreated patients bearing mutated p53, SIAH1 negatively correlated with survival. Numerous repositioned and experimental drugs targeting FEN1 and MMP2 were identified and selected drugs tested. Epinephrine and myricetin, which target FEN1, have shown cytotoxic effect on Mero-14 cells whereas marimastat and batimastat, which target MMP2 demonstrated a modest but significant inhibitory effect on MPM cell migration. Finally, 8 genes displayed correlation with disease stage, which may have diagnostic implications.

**Conclusions:**

Clinical decisions related to MPM personalized therapy based on individual patients’ genetic profile and previous chemotherapeutic treatment could be reached using computational tools and the predictions reported in this study upon further testing in animal models.

**Electronic supplementary material:**

The online version of this article (10.1186/s12967-018-1650-0) contains supplementary material, which is available to authorized users.

## Author summary

Mesothelioma is a rare type of cancer with a notoriously poor prognosis whose incidence is heavily tied to asbestos exposure. Due to the long latency period from asbestos exposure to mesothelioma development, it is expected that incidence of the disease will peak between 2015 and 2030, which represents a significant unmet need due to the ineffectiveness of current therapies. Here we employ a combination of computer simulation techniques along with data from both mesothelioma cells and from mesothelioma patients to identify genes that are correlated with both patient survival and disease stage. Through the use of drug databases we have also identified a number of drugs that may have therapeutic benefit in mesothelioma, with these suggested therapies tailored to individual patients based on disease stage, genetic status, and type of therapy received. Ultimately this research provides evidence for the effectiveness of an interdisciplinary approach of computational research and clinical medicine, that may upon further testing improve patient outcomes.

## Background

Malignant pleural mesothelioma (MPM) is an aggressive cancer mostly related to asbestos exposure, affecting 2570 patients every year in the UK and approximately 20,000 worldwide. Due to the continuing exposure in certain countries and the latency period from asbestos exposure to mesothelioma development, a rise in MPM incidence worldwide is expected during the coming decades [1]. Currently, there are no effective treatments for MPM and the prognosis of patients is invariably poor. The current standard therapy for MPM (cisplatin/antifolate, as well as potentially surgery and radiotherapy in selected patients) improves the average survival by approximately two and half months [1].

Moreover, modern targeted therapies that have shown benefit in other human tumors, have failed in MPM [2, 3] possibly due to specific biological characteristics of this tumor that should be investigated more extensively. Therefore there is an urgent need to find ways to diagnose MPM at an earlier stage and increase knowledge about the mechanisms underlying carcinogenesis, progression and chemo-radio-resistance of this neoplasm.

Development of new therapies is based mainly on our better understanding of potentially “targetable” molecules and their relevance to clinical outcomes. Recent reports have highlighted important features that may cause specific MPM resistance to therapy including alterations in p53 signaling [4–6] and hypoxic [7] pathways. Genomic and transcriptomic analysis of MPM patients has shown the existence of aberrations in the p53 network, for instance mutations in the ARF pathway, which regulates p53 [4, 8, 9]. Thus, improved understanding of the p53 network and its role in mesothelioma may contribute towards improved therapeutic outcomes.

Different classes of chemotherapeutics are used to induce apoptosis in cancer cells. DNA damaging agents such as the topoisomerase II inhibitor etoposide (ETO) are the most commonly used drugs to treat leukemia and solid tumors due to their capacity to induce TP53 activation [10]. In turn the “guardian of the genome” p53 tumor suppressor induces apoptosis in cells with DNA damage and is found mutated in over half of all human cancers [11]. Thus, intensive investigation of the TP53 signaling is carried out (more than 91,000 published articles related to p53 as of September 2018) and detailed understanding of the alterations that occur in its network when this tumor suppressor is mutated is needed to improve the outcomes of the TP53-network based therapies.

Computational research methodologies offer the possibility of integrating large data sets in a comprehensive manner through modeling. Systems biology aims to accurately model biological phenomena, with approaches such as Boolean modeling being suitable for network generation and dynamic analysis. Genes/proteins, as well as biological stimuli or output processes, are represented by the model’s nodes whilst the interactions between these network elements are the model’s edges. These models are able not only to predict steady states and time-course dynamics of complex systems but also to simulate in vivo loss-of-function mutations through node deletion [12].

We have previously demonstrated the successful application of this modeling approach through the generation of glucocorticoid receptor and TP53 interactomes, which uncovered novel levels of signaling in these two pathways [11, 13]. The TP53 interactome (p53 model constructed by Kun Tian, containing 206 nodes [PKT206]) consists of 206 nodes connected by 738 edges, and generates predictions as to how the relationship between model constituents is altered following loss of network elements. Correct prediction rates [as assessed by microarray validation in human osteosarcoma cell lines using logical steady state analysis (LSSA)] reached rates as high as 71% for this model [11]. The alternative algorithm STSFA (signal transduction score flow algorithm) aimed to overcome the limitations of qualitative analysis brought by Boolean modeling, allowed for analysis in a semi-quantitative manner, and demonstrated improved predictive power over the original LSSA analysis [14]. The STSFA has also been preliminarily used to assess model performance as a predictive clinical tool, through its application to our glucocorticoid receptor interactome using microarray data from thirteen leukemia patients [13, 15, 16]. This preliminary analysis also showed a potential correlation of model predictions with clinical outcomes, which provides further evidence of the strength of this modeling approach to understand cancer, identify novel drug targets and predict responses to treatment.

To further analyze the predictive capacity of our p53 model, we tested its predictive capabilities using an MPM cell line and patients’ transcriptome data and demonstrated the high predictive features of this modeling approach in MPM. We also identified genes that are correlated with survival depending on the patients’ p53 status and previous therapy, linked the expression of specific genes to particular stages of MPM and proposed rational approaches which, upon further testing and validation, could direct the selection of personalized therapeutic schemes.

## Results

### Microarray analysis of MPM cells after treatment with etoposide or gemcitabine

We have previously described the PKT206 model which was validated using U2OS human osteosarcoma cell lines as well as human colon carcinoma HCT 116 cell lines demonstrating high levels of correct predictions [11, 14]. In order to determine if model predictions extend to other cancer types we performed microarray analysis in the Mero-14 human MPM cell line treated with the DNA damaging agents etoposide and gemcitabine (GEM) which are used in the clinical settings to treat various cancers [17–19].

**Fig. 1:**
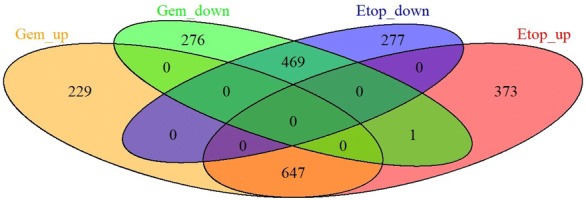
Venn diagram showing genes regulated in similar or different manner in etoposide and gemcitabine treated cells

In Mero-14 cells the expression of 1767 genes was altered upon etoposide treatment out of which 1021 genes were up-regulated and 746 genes were down-regulated (Additional file 1: Table S1, Additional file 2: Table S2 and Additional file 3: Table S3). In gemcitabine-treated Mero-14 cells the expression of 1622 genes was altered compared to the expression level of these genes identified in the control untreated cells. In particular, 876 genes were upregulated and 746 genes were downregulated (Additional file 4: Table S4, Additional file 5: Table S5 and Additional file 6: Table S6). A high number of the same genes (647) were found to be upregulated in cells treated with either etoposide or gemcitabine with 469 identical genes downregulated in either etoposide or gemcitabine-treated cells (Fig. 1). Certain genes specifically up or downregulated by either etoposide or gemcitabine have been identified, whereas the H1 histone family member 0 (H1F0) gene was found to be upregulated in etoposide and downregulated in gemcitabine treated cells (Fig. 1).

PKT206 model validation was undertaken on a genome-wide scale using Mero-14 cells by two approaches: LSSA as well as STSFA in conjunction with high-throughput data such as microarrays [11, 20, 21].

### Superimposition of mesothelioma Mero-14 cell line transcriptome data to the PKT206 model

Superimposition of human Mero-14 malignant mesothelioma cell line gene expression profiles to PKT206 was performed as previously described [11]. Briefly, the in silico DNA damage input was switched ‘ON’ to mimic conditions of cells treated with gemcitabine or etoposide, and ‘OFF’ to mimic cells that were not treated. No p53 knockout scenario was generated as no p53 null cell lines were used to create a p53 wild type versus p53 knockout scenario.

One ninety nine genes were filtered from Mero-14 mesothelioma cell line treated with etoposide and gemcitabine expression profiles and compared to the model [21]. Use of LSSA algorithm to validate the model resulted in 149 correct (74.87%) predictions, 48 (24.12%) small errors and 2 (1.01%) large error following validation based on Mero-14 cells treated with gemcitabine against untreated sample (Table 1, top row). Second analysis was performed in Mero-14 cell lines treated with etoposide (Table 1, bottom row). For the simulation of mesothelioma cells treated with 10 µM etoposide 199 genes were analyzed, a total of 142 correct predictions (71.36%) were obtained with 56 small errors (28.14%) and one large error (0.51%). Large errors occupied the minority of all these predictions and the greatest number of correct predictions was obtained for gemcitabine simulations.

**Table 1:** Model evaluation by LSSA and microarray analysis of mesothelioma cell line Mero-14

Source scenario in Mero-14	Target scenario in Mero-14	LSSA simulation	Total number of genes	Number of correct predictions	p-value of correct predictions	Number of small error predictions	Number of large error predictions
TP53 wt without treatment	TP53 wt with Gem treatment	TP53 wt with DNA damage ON vs TP53 wt with DNA damage OFF	199	149 (74.87%)	4.33 × 10^−33^	48 (24.12%)	2 (1.01%)
TP53 wt without treatment	TP53 wt with etoposide treatment	TP53 wt with DNA damage ON vs TP53 wt with DNA damage OFF	199	142 (71.36%)	5.88 × 10^−28^	56 (28.14%)	1 (0.5%)

In the next set of simulations STSFA was applied to the same set of data as described above (Table 2, Additional file 7: Table S7). Total number of genes analyzed was 191 and simulation results were compared to Mero-14 mesothelioma cell line treated with etoposide and gemcitabine microarray expression profiles. This simulation resulted in 163 correct (85.34%) predictions, 28 (14.66%) small errors and no large errors, following validation based on Mero-14 cells treated with gemcitabine compared to control (Table 2, top row). Second analysis was performed in Mero-14 cell lines treated with etoposide (Table 2, bottom row). 191 genes were analyzed, and 157 correct (82.2%) correct predictions were recorded with 34 (17.8%) small errors and no large errors. Similarly to LSSA simulations, the greatest number of correct predictions was obtained for gemcitabine simulations.

**Table 2:** Model evaluation by STSFA and microarray analysis of mesothelioma cell line Mero-14

Source scenario in Mero-14	Target scenario in Mero-14	STSFA simulation	Total number of genes	Number of correct predictions	p-value of correct predictions	Number of small error predictions	Number of large error predictions
TP53 wt without treatment	TP53 wt with Gem treatment	TP53 wt with DNA damage ON vs TP53 wt with DNA damage OFF	191	163 (85.34%)	6.02 × 10^−50^	28 (14.66%)	0 (0%)
TP53 wt without treatment	TP53 wt with etoposide treatment	TP53 wt with DNA damage ON vs TP53 wt with DNA damage OFF	191	157 (82.2%)	6.8 × 10^−44^	34 (17.8%)	0 (0%)

### Superimposition of mesothelioma tumors expression profiles to the PKT206 using LSSA

As good predictive ratios were obtained from superimposition of in vitro transcriptome data to the PKT206 model we further evaluated its capability to predict differential gene expression changes on patient derived RNA-Seq data obtained from Bueno et al. [4]. RNA sequencing data were processed by the software package edgeR and normalized by the trimmed mean of M values method (TMM). Then differential expression analysis was performed using the negative binomial generalized linear model (glm) method. The differentially expressed genes were filtered by the fold change of 1.5 and p-value < 0.05. Transcriptome data from 71 patients and 200 genes in total were filtered and compared to the results obtained from LSSA (Table 3). Depending on the scenario, correct predictions ranged between 52 and 84% whilst small errors were between 15.5 and 45%. Large errors occupied only a small number of cases (0.5–3%). Therefore this model demonstrated high predictive capacity (up to 84%) when interrogating patient-derived omics type of data.

**Table 3:** Model evaluation by LSSA and RNA-sequencing analysis (Chemoth stands for chemotherapy)

Source scenario in patients	Target scenario in patients	LSSA simulation	Total number of genes	Number of correct predictions	p-value of correct predictions	Number of small error predictions	Number of large error predictions
TP53 wild type treated with chemoth	TP53 mutant treated with chemoth	TP53 null with DNA damage ON vs TP53 wt with DNA damage ON	200	104 (52%)	2.03 × 10^−8^	90 (45%)	6 (3%)
TP53 wild type not treated with chemoth	TP53 mutant not treated with chemoth	TP53 null with DNA damage OFF vs TP53 wt with DNA damage OFF	200	109 (54.5%)	3.77 × 10^−10^	86 (43%)	5 (2.5%)
TP53 mutant not treated with chemoth	TP53 mutant treated with chemoth	TP53 null with DNA damage ON vs TP53 null with DNA damage OFF	200	150 (75%)	1.92 × 10^−33^	46 (23%)	4 (2%)
TP53 wild type not treated with chemoth	TP53 wild type treated with chemoth	TP53 wt with DNA damage ON vs TP53 wt with DNA damage OFF	200	168 (84%)	1.921.92 × 10^−49^	31 (15.5%)	1 (0.5%)

### Superimposition of mesothelioma tumors expression profiles to the PKT206 using STSFA-correlations with survival and tumor stage

The 71 patients described in Bueno et al. [4] were stratified into four groups depending on their p53 status and whether or not they were treated with chemotherapy. These groups were wild-type p53 treated, or not treated with chemotherapy, and mutant p53 treated or not treated with chemotherapy. STSFA scores were calculated for each gene/process and for each patient (Additional file 8: Table S8). Then these scores were correlated with the survival after surgery as indicated in [4]. Genes exhibiting the highest correlative scores with survival are shown in Tables 4, 5. Two methods were employed to obtain this correlation. First, the Pearson coefficient correlation method that correlated gene STSFA score and survival time after surgery was used and results shown in the Table 4 and Additional file 8: Table S8. The second method used was Cox proportional hazards regression analysis, which investigates the association between survival time of patients and other parameters (covariates) using the Cox proportional hazards model (Table 5 and full analysis in Additional file 9: Table S9). Genes in Tables 4, 5 which are negatively related with survival, were also analyzed by the Cox proportional hazards regression analysis and the results are shown in Table 5 below. The values in the beta column indicate the regression coefficients. A positive value indicates the risk of death is higher and a negative value means the hazard is lower. The column of HR (95% CI for HR) summaries the hazard ratio (the exponentiated coefficients) of the covariate and the hazard ratio intervals between the lower 95% confidence bound and the upper 95% bound. The global statistical significance results are also represented by the Wald test value and the p-value of the test.

**Table 4:** Correlation of genes and processes with survival of mesothelioma patients; the Pearson correlation coefficient is shown

TP53 WT T		TP53 WT UT		TP53 MUT UT	
Gene	Survival correlation	Gene	Survival correlation	Gene	Survival correlation
CKB	0.495	DDIT4	− 0.507	E2F1	− 0.498
MUC1	− 0.510	MMP2	− 0.521	FOXM1	− 0.504
FOXM1	− 0.519			NLRC4	− 0.508
E2F1	− 0.526			CDC20	− 0.512
SFN	− 0.530			AURKA	− 0.515
CKS2	− 0.533			RAS	− 0.518
CHEK1	− 0.534			PLAUR	− 0.519
HSP90AB1	− 0.535			MCTS1	− 0.522
RECQL4	− 0.535			GAPDH	− 0.523
PTTG1	− 0.539			EZH2	− 0.534
AURKA	− 0.559			ECT2	− 0.548
PRC1	− 0.563			PRC1	− 0.561
HMMR	− 0.575			APAF1	− 0.566
FEN1	− 0.582			BRCA1	− 0.579
				HSP90AB1	− 0.583
				NCL	− 0.604
				MAPK14	− 0.611
				HIF1A	− 0.624
				SIAH1	− 0.702

Patients treated by chemotherapy are indicated by T; patients untreated by chemotherapy are indicated by UT. Wild type p53 status is labelled WT and mutant is labelled MUT

**Table 5:** Univariate Cox regression analysis

Gene name	Beta coefficient	Hazard ratio (95% confidence interval for HR)	p value
CKB	− 0.0621	0.97 (0.819–1.08)	0.376
MUC1	0.181	1.2 (0.944–1.47)	0.0566
FOXM1	0.462	1.59 (1.28–1.96)	1.89E−05
E2F1	0.143	1.15 (1.06–1.25)	0.000551
SFN	0.0768	1.08 (0.983–1.19)	0.111
CKS2	0.408	1.5 (1.19–1.91)	0.000783
CHEK1	0.55	1.73 (1.3–2.31)	0.000165
HSP90AB1	0.494	1.64 (1.25–2.16)	0.000412
RECQL4	0.442	1.56 (1.25–1.94)	8.71E−05
PTTG1	0.397	1.49 (1.18–1.88)	0.000819
AURKA	0.438	1.55 (1.25–1.93)	8.47E−05
PRC1	0.435	1.54 (1.27–1.88)	1.31E−05
HMMR	0.355	1.43 (1.18–1.73)	0.00029
FEN1	0.345	1.41 (1.05–1.91)	0.024
DDIT4	0.348	1.42 (1.16–1.73)	0.000689
MMP2	0.14	1.15 (1.06–1.24)	0.000414
NLRC4	0.109	1.12 (0.844–1.47)	0.443
CDC20	0.361	1.43 (1.21–1.71)	4.61E−05
RAS	0.32	1.38 (1.11–1.71)	0.0039
PLAUR	0.185	1.2 (0.98–1.48)	0.0779
MCTS1	0.542	1.72 (1.09–2.71)	0.0194
GAPDH	0.119	1.13 (0.86–1.47)	0.388
ECT2	0.49	1.63 (1.3–2.04)	1.87E−05
EZH2	0.184	1.2 (1.09–1.33)	0.000268
APAF1	0.443	1.56 (1.15–2.1)	0.00383
BRCA1	0.203	1.22 (1.11–1.35)	6.32E−05
HIF1A	0.146	1.16 (1.01–1.33)	0.0404
MAPK14	0.139	1.15 (0.803–1.64)	0.447
NCL	0.504	1.66 (1.13–2.43)	0.00978
SIAH1	0.143	1.15 (0.974–1.37)	0.0986

The Cox regression results for genes listed in the Table 4 are shown in the Table 5. The column of beta coefficient shows the beta regression coefficient. The column of HR [95% confidence intervals (CI) for HR] list the hazard ratios (the exponentiated coefficients) and the size of the confidence intervals of the hazard ratios. The column of p value lists the p value for the Wald test method

Different genes were found to correlate with survival depending on the patient’s p53 status (Tables 4, 5). In particular the highest negative correlation (− 0.582) obtained using Pearson correlation with survival for treated patients with wild type p53 was found to be the flap structure-specific endonuclease 1 (FEN1) [22] gene (Table 4 and Additional file 10: Table S10). FEN1 is an endonuclease involved in DNA replication and error prone DNA repair [22]. In not treated with chemotherapy patients carrying wild-type p53 the highest negative correlation with survival was observed for MMP2 (− 0.521), which is a matrix metallopeptidase involved in cell migration and metastasis [23] (Table 4 and Additional file 9: Table S9). Finally, in the not treated with chemotherapy p53 mutated group of patients the highest negative correlation with survival was observed with SIAH1 (− 0.702) gene. SIAH1 is an E3 ligase involved in the p53 signaling, protein degradation, WNT pathway as well as hypoxia and apoptosis [24] (Table 4 and Additional file 10: Table S10). Negative correlation with survival in patients expressing either wild type or mutant p53 was observed for five genes namely the E2F1, FOXM1, PRC1, HSP90AB1 and AURKA.

It was found that 22 genes out of these 30 genes in the Table 5 have highly significant coefficients, such as PRC1, ECT2, FOXM1, CDC20, BRCA1, AURKA, RECQL4, CHEK1, EZH2, HMMR, HSP90AB1, MMP2, E2F1, CKS2, PTTG1, APAF1, RAS, NCL, MCTS1, FEN1, and HIF1A. Only CKB has negative beta coefficients and the other 29 genes have positive beta coefficients, which means CKB is associated with the better survival and the other genes are associated with the poor survival.

Gene ontology (GO) and the Kyoto Encyclopedia of Genes and Genomes (KEGG) were then used to annotate the individual gene products and the classes of gene products respectively to find the pathway(s) these genes are involved in (Additional file 10: Table S10). The KEGG pathways appearing more frequently included cell cycle, DNA repair, metabolism, and microRNA pathways.

Comparison of the data obtained from the analysis of the patients based on the p53 status (Tables 4, 5) with genes altered in Mero-14 cells (Fig. 1, Additional file 1: Table S1, Additional file 2: Table S2, Additional file 3: Table S3, Additional file 4: Table S4, Additional file 5: Table S5 and Additional file 6: Table S6) indicated that PTTG1, AURKA, DDTI4 and CDC20 were downregulated and FEN1, EZH2 and BRCA1 upregulated upon either etoposide or gemcitabine treatment. PLAUR was upregulated in etoposide treated and HIF-1 alpha upregulated in gemcitabine treated Mero-14 cells.

Then we investigated the DRUGSURV database [25] to unveil drugs specifically targeting the genes with the higher negative association with survival in each group (FEN1, MMP2 and SIAH1 in p53 wt treated, p53 wt untreated and p53 mut untreated respectively). The rationale for this investigation was to address the possibility of stratified treatment for patients classified in the three different groups (Table 6 and Additional file 11: Table S11). For example FEN1 can be targeted directly by 10 drugs approved for various conditions suggesting that these drugs could be repositioned for MPM treatment. In addition, there are 12 experimental drugs targeting FEN1 directly, 13 approved drugs that target FEN1 indirectly and 8 experimental drugs that target FEN1 indirectly (Additional file 11: Table S11). MMP2 can be directly targeted by 2 drugs approved by the U.S. Food and Drug Administration (FDA) (Table 6). There are no FDA approved drugs directly targeting SIAH1 whereas there are approved and experimental drugs that target SIAH1 indirectly (Additional file 11: Table S11).

**Table 6:** Approved drugs that target FEN1 and MMP2 directly (DRUGSURV database)

Gene	Drug-target details
FEN1	Epinephrine
	Gentian violet
	Methyldopa
	Dopamine
	Idarubicin
	Norepinephrine
	Masoprocol
	Quinacrine
	Mitoxantrone
	Levodopa
MMP2	Captopril
	Marimastat

In order to determine if the STSFA scores correlate with the stage of the tumor, 203 genes from the model were grouped into stage groups (Additional file 12: Table S12 modified from Bueno et al. [4], and Additional file 13: Table S13) The expression level of eight genes (AIFM2, CDKN1B, KAT2B, MAP4K4, PDRG1, RRM2B, SLC2A4, ZMAT3) was considered significantly different between the stages 3 and 4 with p-value < 0.05 (Additional file 14: Figure S1). AIFM2, CDKN1B, KAT2B, MAP4K4, RRM2B and ZMAT3 were positively correlated (median score of these genes was higher in the stage 4 than in the stage 3) and PDRG1 and SLC2A4 were negatively correlated (median score of these was lower in the stage 4 than in the stage 3). There were no statistically significant differences between other stages.

These genes were then compared with those identified in etoposide or gemcitabine treated Mero-14 cells to be over- or underexpressed compared to untreated cells. This comparison revealed that the PDRG1 gene is upregulated in ETO or GEM treated cells and negatively correlated with stage 4. Its expression is higher in stage 3 compared to stage 4.

In order to determine which pathways are modulated by these genes we used KEGG, the biological biochemical image database (BBID) and Biocarta databases (Additional file 15: Table S14). Histone acetyl transferase PCAF controls gene expression of numerous genes such as p53, nuclear hormone receptors Notch, IFN-beta and others involved in cancer, immune system and metabolism. CDKN1B is p27 cyclin dependent kinase inhibitor and controls cell cycle progression. Additional pathways include MAPK signaling pathway, DNA metabolism (RRM2B) glucose transporters (SLC2A4) and several of these genes are involved in the p53 signaling pathway. Apoptosis-inducing factor 2 (AIFM2) is an oxidoreductase induced by p53 stimulating apoptosis. p53 and DNA-damage regulated 1 (PDRG1) gene is induced by DNA damage and is a p53 transcriptional target.

Along similar lines described above we investigated the DRUGSURV database to unveil drugs targeting specifically PDRG1 (Additional file 16: Table S15). Although there were no approved or experimental drugs that target PDRG1 directly, we identified 12 drugs that target its interaction partner HSP90AA1 and 8 drugs that target its interaction partner MAP3K3.

**Fig. 2:**
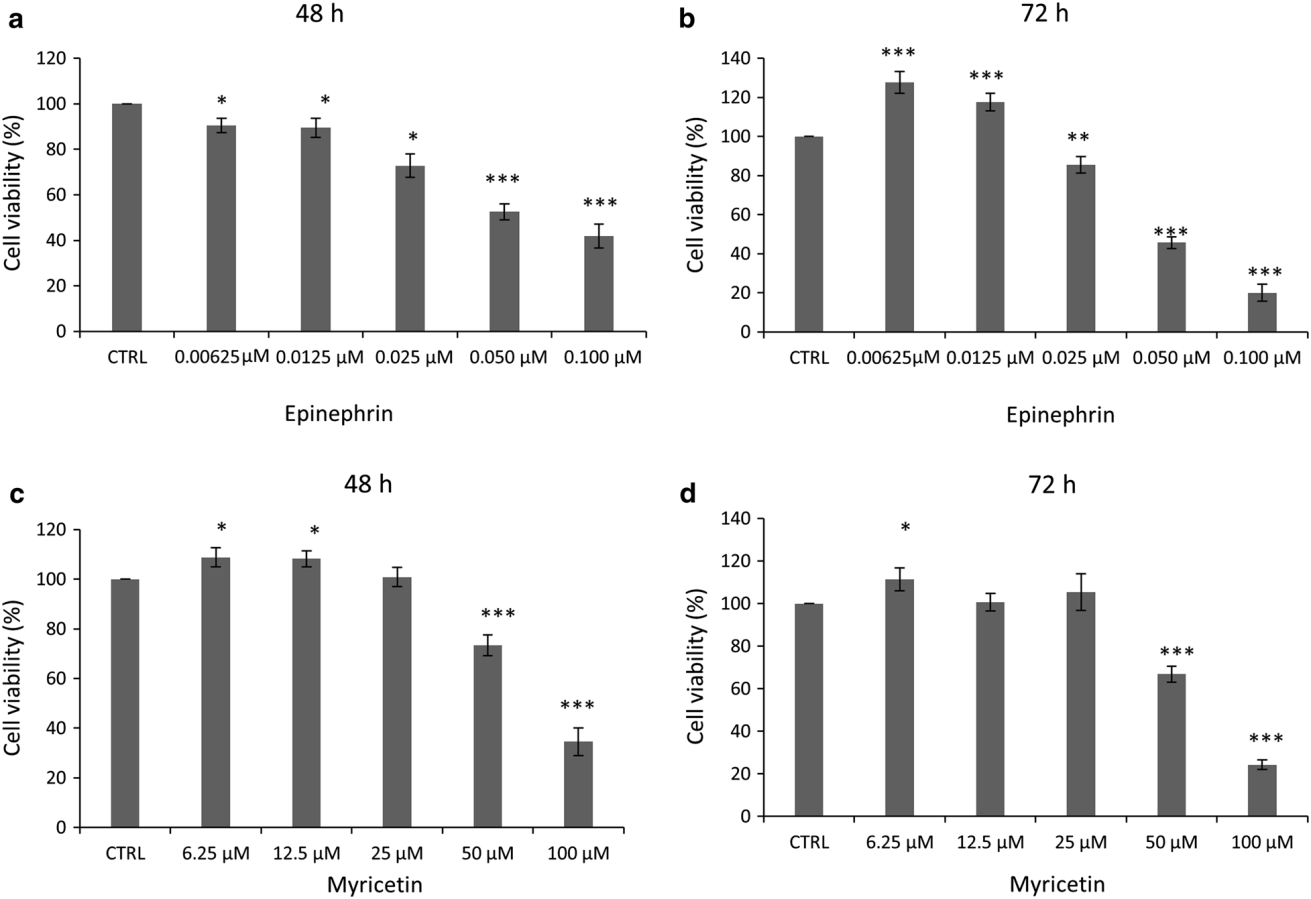
Effect of FEN1 inhibitors on Mero-14 cell survival. SRB assay was used as described in “Methods” to treat Mero-14 cells for 48 h (**a**, **c**) or 72 h (**b**, **d**) with indicated concentrations of epinephrine (**a**, **b**) or Myricetin (**c**, **d**). Error bars represent SEM of three or more independent experiments. p-value ≤ 0.05, 0.01 and 0.001 is indicated as *, ** or ***, respectively

**Fig. 3:**
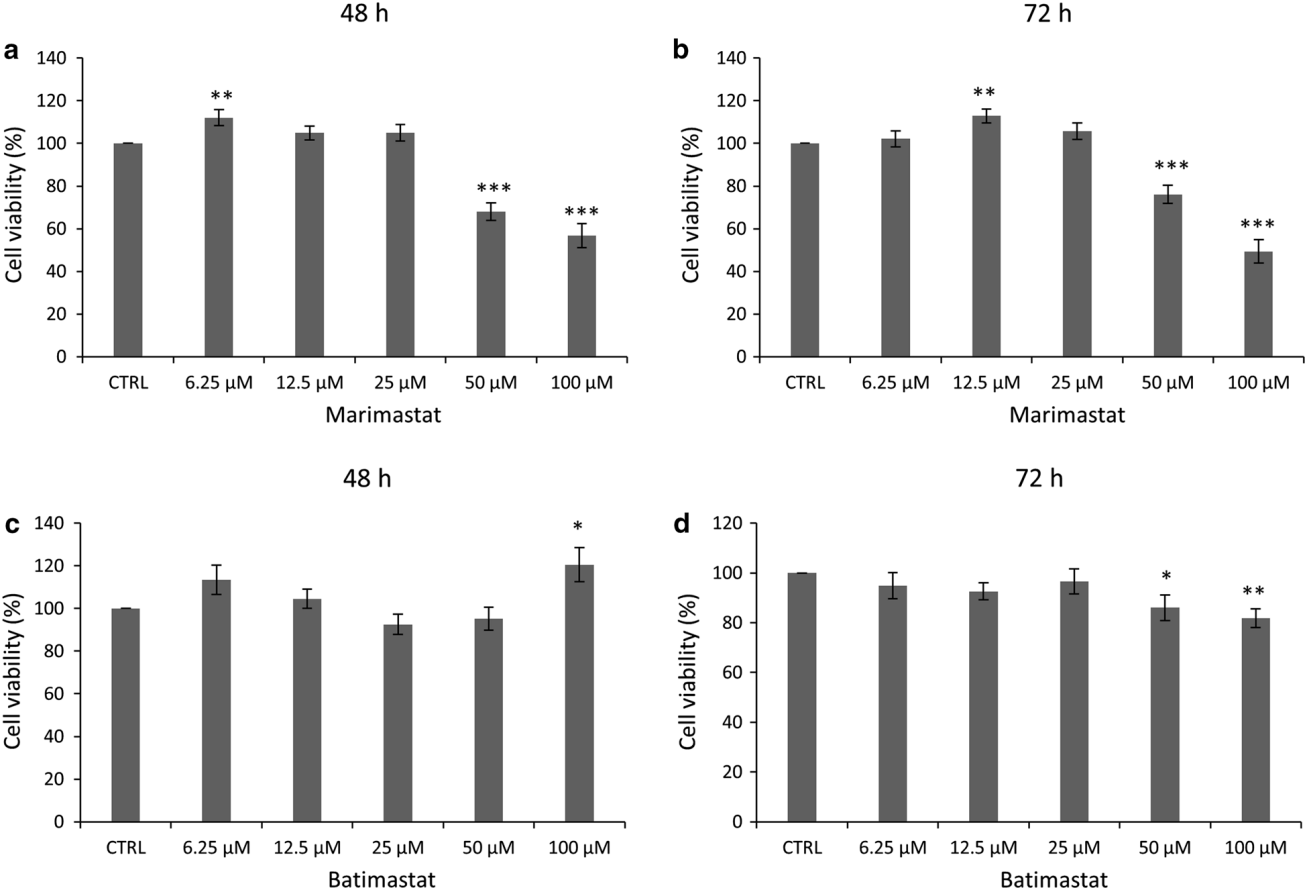
Effect of MMP2 inhibitors on Mero-14 cell survival. SRB assay was used as described in “Methods” to treat Mero-14 cells for 48 h (**a**, **c**) or 72 h (**b**, **d**) with indicated concentrations of Marimastat (**a**, **b**) or Batimastat (**c**, **d**). Error bars represent SEM of three or more independent experiments. p-value < 0.05, 0.01 and 0.001 is indicated as *, ** or ***, respectively

**Fig. 4:**
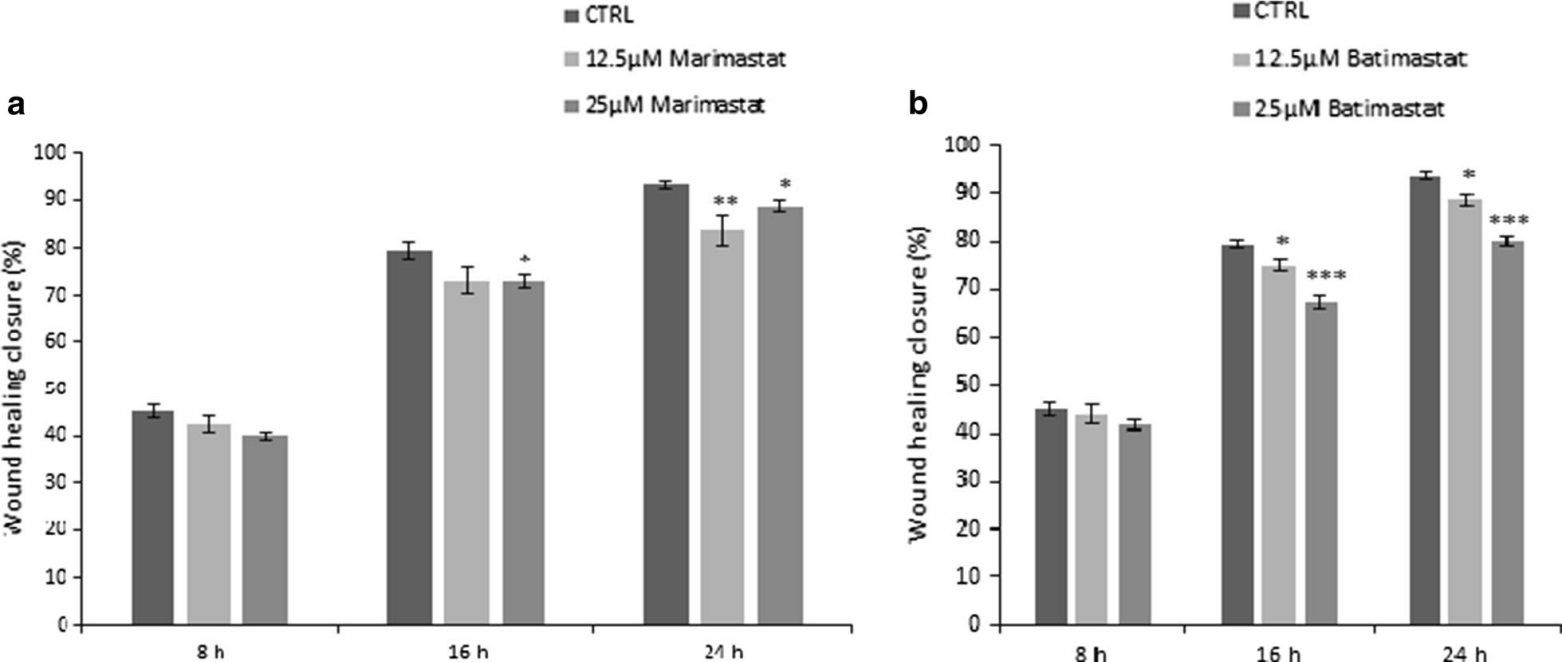
Wound healing assay of Mero-14 cell lines treated with MMP2 inhibitors (marimastat (**a**) and batimastat (**b**)) for 24 h. The wound healing was measured every two hours. Error bars represent SEM of two independent experiments each performed four times. p-value of < 0.05, 0.01 and 0.001 is indicated as *, ** or ***, respectively

Given that both Pearson correlation and Cox regression analysis identified FEN1 and MMP2 as correlating negatively with survival, we used the DRUGSURV database (Table 6 and Additional file 11: Table S11 to identify approved and experimental drugs that target them. We analyzed the effects of epinephrine, a drug approved for various conditions, and predicted to target directly FEN1, as well as myricetin that is an experimental drug predicted to target FEN1 directly, to analyze their effects on Mero-14 cell viability (Fig. 2). Epinephrine inhibited Mero-14 cell survival at high concentrations and more effectively when incubated with cells for 72 h then at 48 h (Fig. 2a, b). Myricetin demonstrated inhibition of cell viability up to approximately 70% after 48 h treatment and up to 80% at 72 h treatment at 100 µM dose, suggesting a cytotoxic effect of this drug on these MPM cells (Fig. 2c, d). In the next set of experiments, we identified drugs that target MMP2 directly; Marimastat, which is approved for clinical use and Batimastat, an experimental drug. Treatment of Mero-14 cells with marimastat caused cellular cytotoxicity of up to 60% when cells were treated with 100 μM dose for 72 h (Fig. 3a, b). Batimastat treatment had marginal effect on cell survival after 72 h of incubation with 100 µM dose (Fig. 3c, d). Given that MMP2 is a protease involved in metastasis, we employed migration assays to determine the role of its inhibitors on cell migration (Fig. 4). Both Batimastat and Marimastat caused modest but significant inhibition of wound closure in Mero-14 cells incubated for 24 h with 12.5 μM and 25 μM concentrations, with Batimastat showing more potent effects These results provide partial validation of the in silico predictions described previously.

## Discussion

The p53 network is inactivated in most cancers [26], thus understanding the role of this network in cancer development and treatment is pivotal for improving cancer therapy. In mesothelioma inactivation of the p53 network is frequent (> 70%) through p53 and the *CDKN2A* locus alterations [4, 6, 9, 27]. Here we use a systems biology approach to integrate clinical data with high-throughput ‘omics’ data in order to understand the role of DNA damage in cancer development and therapy. Our previous modelling approach used in osteosarcoma [11, 14] was expanded to demonstrate the high predictive capacity of the model in the mesothelioma cell line Mero-14 treated with DNA damaging drugs etoposide and gemcitabine. In addition, we used this model to analyze omics profiles of 71 mesothelioma patients and identify predictors of survival and stage of disease. We linked those predictors to experimental and approved drugs that could be used to devise therapeutic schemes for stratified groups of patients based on their p53 status and received chemotherapy. Finally, initial testing of selected drugs effects provided partial validation of model predictions.

We mimicked in vivo mutations or loss of function of p53 using various in silico simulations and predicted the effects on gene signaling pathways and cellular fate caused by perturbations of DNA damage input (ON or OFF). The in silico DNA damage input can represent, for example, the chemo-therapeutic DNA damaging agent, etoposide or gemcitabine. The superimposition of the model predictions to mesothelioma cell line Mero-14 led to a high level correct prediction rate of 74% for LSSA and up to 85% for STSFA (Tables 1 and 2 respectively). This compares to 51–71% prediction rates for LSSA analysis of osteosarcoma and colon cancer cell lines we observed previously [11]. In addition, we identified that in Mero-14 cells treated with etoposide and gemcitabine the majority of genes are regulated in the same way, however a portion of genes is specific to the drug and histone H1, member 0 is regulated in opposite manner (Fig. 1). This may have implications to which drug is chosen to treat particular cancer depending on signaling pathways regulated and genes affected. This approach also confirmed that the predictive capacity of the model is high across osteosarcoma, colon and mesothelioma cancer cell lines.

Some modelling approaches of cellular processes have been reported. For example, Kirouac et al. [28] developed a Boolean model of drug resistance in breast cancer that had Erbb2 amplified and tested the in silico interactome prediction using in vitro and mouse model systems. Fumia et al. [29] constructed a large scale network with several inputs including hypoxia, to predict molecular drivers of carcinogenesis. Mai and Lui [30] constructed a network considering 40 nodes and found that apoptosis is an irreversible process, whilst Schlatter et. [31] developed a large scale literature based Boolean model comprising 125 interactions and 86 nodes and analyzed the behavior of apoptotic pathways. Other p53 Boolean models have also been reported. Poltz and Naumann [32] investigated p53 and NFKB pathways in response to in silico DNA damage inputs simulations of single and double stranded breaks using a Boolean model that was validated by experimental methods, revealing carcinogenesis drivers and candidate targets for chemo-sensitization strategies. However, modelling approaches that can predict potential novel biomarkers and drug targets upon analysis of patients’ data are not frequently reported in literature and databases that hold omics type of data or survival curves for mesothelioma are lacking.

Despite the fact that number of patients analyzed is not high, and different number of patients was present in different groups, given the rarity of mesothelioma, we have used these data to obtain limited but important conclusions. We have identified 30 genes that negatively correlate with survival after surgery (Tables 4, 5). These differ depending on p53 status and therapy. In patients that have wild type p53 and were treated with chemotherapy there are 14 genes that have been identified and they are involved in numerous signaling pathways (Tables 4, 5 and Additional file 10: Table S10), with cell cycle being the most represented followed by DNA repair, microRNA and metabolism. The top scoring gene was FEN1 indicating that high expression of this gene correlates with poor prognosis. This could be potentially because FEN1 gene [22] is part of error prone DNA repair pathway thus increased DNA repair rate is likely to lead to increased mismatch and higher mutational load. This could help to achieve better selection (which is currently badly needed) of patients with MPM that are candidates for immune checkpoint inhibitors. DRUGSURV search identified 10 already approved drugs targeting FEN1 that could be repositioned to treat mesothelioma (Table 6). In patients with wild-type p53 that were not treated with chemotherapy there were two genes identified to negatively correlate with survival (DDIT4 and MMP2) (Tables 4, 5). The highest score was obtained for MMP2 [23], which is involved in cell migration and metastasis, indicating that in this group of patients higher expression of this gene is a negative prognostic factor possibly because of higher chances of metastasis. There are 12 drugs that we found that target MMP2 and can be repositioned for mesothelioma treatment of patients that have wt p53 and have not been treated with chemotherapy (Table 6).

In patients with mutant p53 that have not been treated with chemotherapy 19 genes were identified with significant negative correlation to survival (Table 4). The top scored gene was SIAH1 [24] and its potential involvement may be in affecting WNT pathway or through hypoxia signaling. Although there are no drugs directly targeting SIAH1, 6 already approved drugs that target its interaction partners UBE2N and 9 that target STAT3 could be tested in mesothelioma patients carrying mutated p53. There are several genes identified in both p53 wild type and mutant cases including the E2F1 transcription factor that is a master regulator of cell cycle [33], FOXM1 that is a member of forkhead family of transcription factors that control proliferation [34], and PRC1 involved in control of cytokinesis and chromosomal stability [35]. Given that several drugs that have already been FDA approved can directly target these proteins, this provides the possibility of repositioning these drugs for mesothelioma patients that are stratified according to the p53 status and chemotherapy received (Table 6). Initial testing of epinephrine and myricetin that target FEN1 indicated that they have cytotoxic effect on Mero-14 cells (Fig. 2). Both Batimastat and Marimastat that target MMP2 showed cytotoxic effect and inhibited migration of Mero-14 cells in vitro, with Marimastat having stronger cytotoxic effect, whereas Batimastat was more efficient in inhibiting cell migration (Figs. 3 and 4). Given that both epinephrine and marimastat are used in medicine, there is a possibility of repositioning these drugs for MPM treatment upon further testing.

MPM is difficult to diagnose, therefore early detection is crucial for improving the therapy and identification of novel potential biomarkers will facilitate this process. In order to link tumor progression with gene expression profiles and p53 status, STSFA scores of studied genes were analyzed in different patients groups according to their stages (S12-S13 Tables). The expression level of 8 genes was found to be significantly different between stages 3 and 4. AIFM2, CDKN1B, KAT2B, MAP4K4, RRM2B and ZMAT3 exhibiting higher expression levels in stage 4 compared to stage 3 control cell cycle, p53 and NOTCH pathways. PDRG1 and SLC2A4 gene expression is lower in the stage 4 than in the stage 3 and they regulate DNA damage, p53 pathway and are involved in insulin signaling.

Comparison with genes that are altered by etoposide and gemcitabine in Mero-14 cell line indicated that the PDRG1 gene is upregulated in cells exposed to DNA damage, and although its role in cancer development is not well described potential drugs that can target it indirectly are available (Additional file 16: Table S15).

## Conclusion

We have successfully validated the use of the p53 network model in mesothelioma cell line and patients, and we have shown that the PKT206 model is a promising predictive tool for development of stratified anti-cancer therapies. In addition, we have identified novel deregulated pathways that upon further testing in animal models could offer potential targeted drug therapy combinations. This approach could predict patients’ responses to therapy according to their genetic profile and identify the best therapeutic schemes during the course of the treatment, leading to improved and personalized treatment of mesothelioma patients.

## Methods

### Cell culture

The human mesothelioma cell line Mero-14 that has wild type p53 and expresses p53 protein (Additional file 17: Figure S2) was used as a predicted wild type p53 source and was donated by Prof. Landi (University of Pisa) [36]. Mero-14 cells were cultured in Dulbecco’s Modified Eagle’s Medium (DMEM) (Sigma, UK) supplemented with 10% fetal bovine serum (Gibco, UK), and 1% penicillin/streptomycin (Lonza, USA) and maintained at 37 °C in a 5% CO_2_ humidified atmosphere. The etoposide and gemcitabine were purchased from Sigma, UK.

#### Sulphorhodamine (SRB) assay

Cells were plated at a density of 0.03 × 10^5^ cells/well in a 96-well plate 24 h before treating cells with the desired drugs. Cells were fixed with 10% trichloroacetic acid (TCA) for 1 h, and dried overnight at room temperature followed by staining with SRB for 15 min, washed twice with 1% acetic acid, and air dried for at least 1 h. The incorporated SRB staining was dissolved in 10 mM Tris pH 8.8 solution and then plates were analyzed using a calorimetric microplate reader (Thermo Labsystem Multiskan Ascent) (Akribis Scientific, UK) at a wavelength of 540 nm and 690 nm, according to In Vitro Toxicology Assay Kit Sulforhodamine B based protocol (Sigma, UK).

#### Western blot analysis

Cells were harvested in ice-cold RIPA buffer containing 20 mM BGP, 1 mM PMSF, 1 mM DTT, 5 mM NaPPi and 2 mM NaOV and 1 µg/ml protease and phosphatase inhibitors (Sigma, UK). Bradford assay (Sigma, UK) was used for protein quantification. 30 µg of proteins was loaded into SDS polyacrylamide gel, then transferred to the PDVF membrane. Membranes were incubated overnight for blocking at 4 °C with 5% no-fat dry milk in PBS and then treated with primary and secondary antibodies and blots developed using ECL substrate according to manufacturer’s instructions (Pierce, Thermo Scientific, USA). The following primary antibodies were used for western blotting: β-actin (Abcam, UK) and p-53 (Santa Cruz Biotechnology, Inc.).

#### DNA extraction and total TP53 sequencing analysis

Genomic DNA was isolated from Mero-14 cell line using Phase Lock Gel™ (PLG) (VWR International Ltd, UK) according to the manufacturer’s genomic DNA isolation instruction. The entire coding sequence of the TP53 gene (exons 1–11) was amplified using designed primers by Liu and Bodmer [37].

The standard PCR was performed in a volume of 50 µl containing 200 ng template DNA, 10 pmol of each primer, 25 µl of MyTaq™Red Mix 2X (Bioline, UK) and the appropriate volume of H_2_O. PCR programs were then used to amplify the exons: initial denaturation (1 min at 95 °C), followed by 35 cycles of denaturation (95 °C for 15 s), annealing temperature (Tm; 56 °C for 15 s for all exons, except exon 2 Tm: 57 °C), and extension (72 °C for 30 s), and a final extension of 72 °C for 3 min. PCR products were purified using FavorPrep™Gel/PCR Purification Kit (Favorgen, Germany). 10 ng/µl of purified PCR product were sent to Source BioScience (UK) for Sanger sequencing. Sequence alignment was performed using BioEdit software v7.0.5.3. Gene-Bank accession number NG_017013.2 was used as a reference sequence. In addition, sequence that corresponds to the genomic sequence NC_000017.10 was used for exon alignment.

#### Wound healing assay

For the wound healing assay, Mero-14 cells were seeded at density of 3 × 10^5^ cell/well in 24-well plate and incubated overnight a 37 °C. After achieving confluence, monolayer cells were scratched using a p200 pipette tip. Cells were washed twice with sterile 1× PBS and then treated with marimastat and batimastat both at 12.5 and 25 µM and incubated at 37° C for 24 h in Cytation 3 (BioTeck, UK). The migration of the cells at the edge of the scratch was analyzed at the indicated time. The rate of migration was measured by quantifying the percentage (%) of wound closure area, determined using the software ImageJ, according to the formula:  % of wound closure = [(At = 0 h − At = Δ h)/At = 0 h] × 100%.

### Microarray analysis preparation and processing

Total RNA was extracted from untreated or treated Mero-14 cells with etoposide (10 µM) or gemcitabine (1 µM) for 24 h [38–40] and subjected to The Affymetrix Human Genome U133 plus 2.0 Array (Eurofins, UK) as previously described [15, 41]. The raw data were normalized by robust multi-array average (RMA) algorithm [42] and annotated. As multiple probe ids are annotated to the same gene symbol names, the expression values of same gene from different probes were merged. The median expression value of the same gene in each group was calculated to represent the expression value of these genes. Then the expression value matrix is processed by the Bioconductor platform to identify differentially expressed genes using the limma package, which applies a linear model algorithm for microarray data analysis [43]. Lists of differentially expressed genes were filtered using a threshold of log_2_(1.5) for the log_2_-fold change and a raw p value < 0.05.

### Genome-wide model validation in Mero-14 cell line

#### Model validation using LSSA

As previously described [11], to evaluate the predictive strength of the TP53 model, in silico predictions were compared to experimentally obtained microarray data from mesothelioma cell line Mero-14. To assess the model’s prediction for changes between two states, an *E*_*mod*_ value (this value is obtained from in silico simulations) was calculated which predicted the state change for a node between two scenarios [11]. LSSA assigns a state of inactive (0), undetermined (NaN) or active (1) to each node. For Scenario 1 (i.e. TP53 wild-type DNA damage OFF) node *i* was defined as *S*(*i*)_1_ which could take the values of 0, NaN or 1. For Scenario 2 (i.e. TP53 wild-type DNA damage ON) node *i* was defined as *S*(*i*)_2_ which could take the same values. *E*_*mod*_ was then calculated to predict the state change between the two scenarios where − 1 is downregulated, 0 is unchanged and 1 is upregulated:

$$ E_{mod} = - 1\quad {{\text{if}}}\quad S(i)_{1} = 1\quad {{\text{and}}}\quad S(i)_{2} = 0 $$

$$ E_{mod} = - 1\quad {{\text{if}}}\quad S(i)_{1} = 1\quad {{\text{and}}}\quad S(i)_{2} = {{\text{NaN}}} $$

$$ E_{mod} = - 1\quad {{\text{if}}}\quad S(i)_{1} = {{\text{NaN}}}\quad {{\text{and}}}\quad S(i)_{2} = 0 $$

$$ E_{mod} = 0\quad {{\text{if}}}\quad S(i)_{1} = 1\quad {{\text{and}}}\quad S(i)_{2} = 1 $$

$$ E_{mod} = 0\quad {{\text{if}}}\quad S(i)_{1} = 0\quad {{\text{and}}}\quad S(i)_{2} = 0 $$

$$ E_{mod} = 0\quad {{\text{if}}}\;S(i)_{1} = {{\text{NaN}}}\quad {{\text{and}}}\quad S(i)_{2} = {{\text{NaN}}} $$

$$ E_{mod} = 1\quad {{\text{if}}}\quad S(i)_{1} = 0\quad {{\text{and}}}\quad S(i)_{2} = 1 $$

$$ E_{mod} = 1\quad {{\text{if}}}\quad S(i)_{1} = {{\text{NaN}}}\quad {{\text{and}}}\quad S(i)_{2} = 1 $$

$$ E_{mod} = 1\quad {{\text{if}}}\quad S(i)_{1} = 0\quad {{\text{and}}}\quad S(i)_{2} = {{\text{NaN}}} . $$

The differential expression for a gene between two datasets (i.e. untreated cells and cells treated with etoposide to induce DNA damage) was calculated using the *E*_*exp*_ value (this value is obtained from in vitro experiments) calculated as previously described [11] where − 1 equates to downregulation, 0 to no change and 1 to upregulation. The difference between E_mod_ and E_exp_ can have absolute values of 0, 1 or 2 and was used to evaluate model predictive capacity. A value of 0 was labeled as correct prediction, value of 1 a small error and value of 2 a large error as described in [11].

### Model validation using STSFA

The STSFA score of nodes in the model for each sample was calculated by the Cytoscape plugin, Pathway Scoring Application [20]. The log_2_ medium values of the normalized expression values were scaled up by a factor of 100 to generate microarray experiment inputs of the application as described previously [14]. In the scenario of DNA damage ON, the mean value of all other mapped genes’ score was assigned to the DNA damage input node. Whereas in the DNA damage OFF scenario, the minimum value of all other mapped genes’ score was assigned to the DNA damage node. The minimum value was also assigned to the other un-mapped genes in the model. Then the final score for all the nodes was calculated by the heuristic algorithm [20].

The log_10_ fold change of the STSFA score of each model gene for the scenario was calculated to detect the differentially expressed genes. If this log_10_ fold change value was higher than the upper limit (the mean value of log_10_ fold change plus the standard deviation of the log_10_ fold change) then the gene was considered to be upregulated in the model’s prediction (*E*_*mod*_ = 1), whilst if the value was lower than the lower limit (the mean value of log_10_ fold change minus the standard deviation of the log_10_ fold change) the gene was considered to be downregulated in the model’s prediction (*E*_*mod*_ = − 1), and if the value was between the lower and upper limits then the gene was considered to be unchanged between the two scenarios (*E*_*mod*_ = 0). *E*_*exp*_ was calculated as described previously for the LSSA analysis and the absolute value of *E*_*mod*_ − *E*_*exp*_ was calculated as for the LSSA analysis and could take three possible values: 0 (model prediction was correct); 1 (small error prediction where the model predicts for example upregulation but the gene is unchanged); 2 (large error prediction where the model predicts the opposite of what occurred, i.e. the model predicted downregulation whilst the gene is actually upregulated).

### Clinical model validation using RNA-Seq data from mesothelioma patients

To further validate models using clinical data, published RNA-seq data from Bueno and colleagues [4] was obtained from the European Genome-phenome Archive. The raw RNA-seq data for each patient was downloaded and decrypted using the European Genome-phenome Archive (EGA) download client (EgaDemoClient) and the downloaded fastq files were aligned to the GRCh37 reference genome for *Homo sapiens* using TopHat2 (Additional file 18: Figure S3) [44, 45]. The generated BAM files were processed on the Bioconductor platform to prepare the count matrix. Patients were split into 4 groups: treated with chemotherapy patients with TP53 wild-type (n = 27), not treated with chemotherapy patients with TP53 wild-type (n = 26), treated with chemotherapy patients with TP53 mutant (n = 1) and not treated with chemotherapy patients with TP53 mutant (n = 17). Differential expression analysis for samples of different groups was performed by the functions of the count-based statistical package edgeR (empirical analysis of digital gene expression in R) [46]. Lists of differentially expressed transcripts were filtered using a threshold of log_2_ (1.5) for the fold change and a raw p value lower than 0.05. Transcript identifiers were annotated to gene symbol names by the genome wide annotation for human package, org.Hs.e.g.db [47].

The group comparison results were validated by the LSSA predictions of the PKT206 model as described above. Then the STSFA score of each gene based on individual samples were calculated as described above. Briefly, the log2 transformed read counts of each gene mapped in the PKT206 model were scaled up by a factor of 100 and then imported as the experiment input of the Pathway Scoring Application. The score of the DNA damage input node and the unmapped nodes in the model were assigned by the same method as for Mero-14 microarray STSFA calculation above. The pathway scoring application in the Cytoscape was used to calculate the score of all nodes in the model for each individual patient.

### Analysis of model prediction and correlation with the clinical profile of patients

STSFA scores were correlated with patients survival after surgery (data was published by Bueno and colleagues [4] by calculating the Pearson correlation coefficient and Cox proportional hazards regression analysis. Pearson correlation coefficient provides a measure of the strength of correlation between parameters and can have a value between + 1 and − 1. 1 means there is a positive correlation, 0 there is no correlation, and − 1 means there is negative correlation, with the value of the number indicating the strength of the correlation. Cox proportional hazards regression analyses the simultaneous effects of covariates on the survival that are calculated by this model and quantified as the hazard ratio (HR). HR value equal to 1 indicates no effect between the covariate and the survival. HR < 1 indicates that the covariate is positively associated with the survival (good prognostic factor) and HR value more than 1 indicates the covariate is negatively associated with the survival (bad prognostic factor). The univariate Cox proportional hazards regression analysis is performed by functions of the survival package and the survminer package on the R platform.

Patients were also grouped according to the stage where TNM information was modified according to MPM staging classification (https://emedicine.medscape.com/article/1999306-overview). 203 genes from the PKT206 model were analyzed and their median scores compared between different stages of disease. Analysis of variance (ANOVA) was used to identify genes that have a p value < 0.05. The genes that were most positively or negatively correlated with patients’ survival were loaded in the database for annotation, visualization and integrated discovery (DAVID) [48] and the biological pathways that were altered by those genes were determined by the Kyoto Encyclopedia of Genes and Genomes [49]. Drugs that can target relevant gene products were identified through the use of DRUGSURV database [25].

## Additional files

**Additional file 1: Table S1.** ETO vs control (ctrl) differentially expressed genes.

**Additional file 2: Table S2.** ETO vs ctrl upregulated genes.

**Additional file 3: Table S3.** ETO vs ctrl downregulated genes.

**Additional file 4: Table S4.** Gem vs ctrl differentially expressed genes.

**Additional file 5: Table S5.** Gem vs ctrl upregulated genes.

**Additional file 6: Table S6.** Gem vs ctrl downregulated genes.

**Additional file 7: Table S7.** Mero-14_STSFA_results.

**Additional file 8: Table S8.** Pearson correlation analysis of the STSFA scores.

**Additional file 9: Table S9.** Univariate_Cox_regression analysis.

**Additional file 10: Table S10.** KEGG pathways.

**Additional file 11: Table S11.** Approved drugs that target FEN1, MMP2 and SIAH1 indirectly and experimental drugs that target FEN1, MMP2 and SIAH1 directly or indirectly (DRUGSURV database).

**Additional file 12: Table S12.** Stage of patients.

**Additional file 13: Table S13.** STSFA_score_groups_by_STAGE.xlsx.

**Additional file 14: Figure S1.** InStat analysis of 8 genes significantly correlated with stages. This file covers the statistical analysis (types of test and p-values that were obtained for the statistical analysis of genes correlated with stage.

**Additional file 15: Table S14.** Signaling pathways controlled by genes correlated with tumor stages.

**Additional file 16: Table S15.** Approved and experimental drugs that target PDGR1 indirectly (DRUGSURV database).

**Additional file 17: Figure S2.** TP53 protein is stabilized by DNA damage in Mero-14 cells. (A) Mero-14 cells were untreated or treated with etoposide (20 µM) for 24 h, after which cells were lysed and protein harvested and subjected to Western blotting using antibodies against TP53 and actin. (B) ImageJ quantification of TP53 expression in Mero-14 cells following etoposide treatment. (C) Sequence alignment of the coding sequence of the TP53 gene (exons 1–11) from Mero-14 cell line.

**Additional file 18: Figure S3.** The schematic workflow of the RNA sequencing analysis for the patient’s data. The schematic diagram depicts the workflow for the RNA sequencing analysis of the patients data. The sequencing data in the format of FASTQ file are aligned by the TopHat2 to generate the input BAM files. The BAM files are processed to obtain the count matrix for the differential expression analysis and the statistical analysis based on the STSFA score of each gene. Differentially expressed genes are identified by R script based on the edgeR packages and utilized to validate the LSSA predictions. The STSFA score of each gene in the model are calculated by the Cytoscape platform and processed for the further statistical analysis.
