# Supplementary material for: p53 modeling as a route to mesothelioma patients stratification and novel therapeutic identification
Source: J Transl Med. 2018 Oct 13;16:282. doi: 10.1186/s12967-018-1650-0 (PMC6186085; doi:10.1186/s12967-018-1650-0)
Supplement: Supplementary file 1 — Additional file 1: Table S1. ETO vs control (ctrl) differentially expressed genes. [file 12967_2018_1650_MOESM1_ESM.docx]

**Table S1:** Etop vs ctrl differentially expressed genes

| **logFC** | **P.Value** | **gene_symbol** |
| --- | --- | --- |
| 2.8719325 | 8.57E-09 | SNHG12 /// SNORA16A /// SNORA44 /// SNORA61 |
| 2.6237075 | 8.71E-09 | GADD45A |
| -2.4641845 | 1.75E-08 | ID2 /// ID2B |
| 2.1859853 | 3.05E-08 | PMAIP1 |
| 2.0382498 | 3.58E-08 | DTL |
| 2.069 | 3.76E-08 | CLGN |
| 2.91829 | 4.00E-08 | DKK1 |
| 2.2416725 | 6.42E-08 | ATF3 |
| 2.0570413 | 7.15E-08 | CLDN1 |
| 1.9657958 | 7.78E-08 | CEBPG |
| 2.424589 | 1.19E-07 | OTTHUMG00000172919 /// RP11-340F14.5 |
| 1.8889978 | 1.28E-07 | MTHFD2 |
| 1.7207235 | 1.46E-07 | NUPR1 |
| -1.6363593 | 1.51E-07 | SYT17 |
| 1.6753345 | 1.69E-07 | ZNF367 |
| 1.9959325 | 1.70E-07 | CDKN1A |
| 1.6669678 | 1.80E-07 | PPP1R15A |
| -1.716372 | 2.02E-07 | KANK4 |
| 1.8387425 | 2.09E-07 | C6orf48 |
| 1.9459685 | 2.22E-07 | DDIT3 |
| 2.1325765 | 2.23E-07 | UPP1 |
| 3.1850185 | 2.32E-07 | INHBE |
| 1.6834728 | 2.52E-07 | SESN2 |
| 1.540492 | 2.82E-07 | SNHG1 /// SNORD22 /// SNORD25 /// SNORD26 /// SNORD27 /// SNORD28 /// SNORD29 /// SNORD31 |
| -1.4969665 | 2.87E-07 | CCDC85C |
| -1.648413 | 3.21E-07 | CTB-174D11.3 /// OTTHUMG00000163878 |
| -1.611348 | 3.59E-07 | VIT |
| 1.8103505 | 3.71E-07 | FLRT2 /// LOC100506718 |
| -1.678047 | 3.86E-07 | MGC24103 |
| 1.516467 | 3.88E-07 | PSMC3IP |
| -1.6747813 | 3.99E-07 | LRP2 |
| 1.4141675 | 4.03E-07 | MCM10 |
| 1.464553 | 4.47E-07 | HSPA14 |
| 1.574487 | 4.52E-07 | RRM2 |
| 1.9489263 | 4.59E-07 | PSAT1 |
| 1.4416885 | 4.94E-07 | FBLL1 |
| 1.71192 | 5.11E-07 | PTX3 |
| 1.559677 | 5.77E-07 | BRCA2 |
| 1.3931213 | 5.87E-07 | TMEM194A |
| 1.55149 | 6.05E-07 | RND3 |
| 1.8222125 | 6.07E-07 | IL6 |
| 1.4859935 | 6.16E-07 | SUPV3L1 |
| 1.8134005 | 6.22E-07 | CCNE2 |
| 1.8086025 | 6.34E-07 | CDC6 |
| -1.4558933 | 6.39E-07 | FAM13C |
| 1.3481065 | 6.47E-07 | ATAD2 |
| 1.9611635 | 6.53E-07 | CASP1 |
| -1.661798 | 6.68E-07 | SERPINB9 |
| 1.3754735 | 6.88E-07 | SNAI2 |
| -1.4081263 | 7.16E-07 | FGF18 |
| -1.636054 | 7.35E-07 | HIST1H3H |
| -1.8510155 | 7.81E-07 | PSRC1 |
| 1.2905495 | 7.99E-07 | CCDC174 |
| 1.467821 | 8.15E-07 | APOBEC3B |
| 1.569791 | 8.21E-07 | SNHG15 /// SNORA9 |
| 1.29094 | 8.39E-07 | TIPIN |
| 1.445903 | 8.39E-07 | FGF2 |
| 1.7701918 | 8.72E-07 | FAM129A |
| 1.3032635 | 8.75E-07 | TMEM38B |
| -1.6280875 | 8.76E-07 | GAL3ST1 |
| -1.3232798 | 8.97E-07 | ST6GAL2 |
| 1.381258 | 9.00E-07 | TGFB2 |
| -1.4072898 | 9.04E-07 | DAPK1 |
| 1.382591 | 9.33E-07 | SRFBP1 |
| -2.202597 | 9.37E-07 | KIF20A |
| 1.481352 | 9.48E-07 | E2F8 |
| 2.203156 | 9.57E-07 | CSTA |
| -1.297428 | 9.98E-07 | CYP26A1 |
| 1.237968 | 1.02E-06 | WDHD1 |
| 1.267126 | 1.07E-06 | SNHG17 |
| 1.843474 | 1.07E-06 | RIBC2 |
| 1.6059255 | 1.09E-06 | EXO1 |
| -1.3223245 | 1.15E-06 | RNASE4 |
| 1.507565 | 1.15E-06 | TNFRSF12A |
| 1.2350895 | 1.16E-06 | SLC3A2 |
| -1.302064 | 1.20E-06 | MLLT4-AS1 |
| 1.228804 | 1.20E-06 | RBBP8 |
| -1.8324375 | 1.21E-06 | BNC2 |
| 1.6159345 | 1.22E-06 | TIGAR |
| -1.497528 | 1.22E-06 | PBX1 |
| -1.897846 | 1.23E-06 | OTTHUMG00000032910 /// RP11-157P1.4 |
| -1.215249 | 1.24E-06 | NFATC4 |
| 1.218632 | 1.25E-06 | UBE2T |
| -1.3078805 | 1.29E-06 | ATF7IP2 /// LOC100287628 |
| -1.4063555 | 1.30E-06 | SEMA5A |
| 2.0770855 | 1.32E-06 | LAMP3 |
| 1.3878098 | 1.32E-06 | TRIB3 |
| 1.223903 | 1.32E-06 | CHAC2 |
| 1.2177203 | 1.35E-06 | CARS |
| 1.8494935 | 1.37E-06 | CARD16 /// CASP1 |
| -1.1870485 | 1.39E-06 | MYL9 |
| 1.356562 | 1.46E-06 | TEX30 |
| 1.644828 | 1.46E-06 | AEN |
| -1.450565 | 1.47E-06 | DLGAP5 |
| 1.212034 | 1.50E-06 | RNASEH2A |
| 1.223855 | 1.51E-06 | PRIM1 |
| 1.692425 | 1.52E-06 | HRK |
| 1.3747355 | 1.53E-06 | CDCA5 |
| -1.3772285 | 1.53E-06 | PIF1 |
| 1.4964455 | 1.53E-06 | IL7R |
| -1.2500515 | 1.53E-06 | CDH3 |
| -1.5410858 | 1.58E-06 | ID2 |
| 1.2882335 | 1.59E-06 | TLCD1 |
| 1.6581488 | 1.61E-06 | FAM111B |
| 1.1620955 | 1.62E-06 | DNAJC9 |
| -1.2382233 | 1.62E-06 | NLGN1 |
| -1.298993 | 1.66E-06 | CTD-2292M16.8 /// OTTHUMG00000178843 |
| 1.2840845 | 1.68E-06 | GARS |
| -1.5057905 | 1.74E-06 | KRT15 |
| 1.1857325 | 1.80E-06 | AARS |
| 1.2777325 | 1.81E-06 | IFRD1 |
| 2.8546965 | 1.82E-06 | ANKRD1 |
| 1.608096 | 1.89E-06 | SHISA2 |
| -1.260828 | 1.92E-06 | ST6GALNAC3 |
| 1.2506055 | 1.92E-06 | WARS |
| 1.2396378 | 1.92E-06 | STRIP2 |
| -1.1340545 | 1.95E-06 | ZSCAN31 |
| 1.1865045 | 1.99E-06 | C11orf82 |
| -1.168318 | 2.01E-06 | LOC150622 |
| -1.5532165 | 2.04E-06 | CDH18 |
| 1.2472228 | 2.04E-06 | WDR76 |
| -1.4605575 | 2.05E-06 | SMTNL2 |
| 1.591078 | 2.07E-06 | CXCL3 |
| 1.808128 | 2.07E-06 | IL8 |
| 1.1771075 | 2.09E-06 | GNL2 |
| 1.17355 | 2.16E-06 | C5orf34 |
| -1.2218608 | 2.17E-06 | AGTR1 |
| 1.7229825 | 2.21E-06 | IL13RA2 |
| 1.391165 | 2.24E-06 | SARS |
| 1.562957 | 2.25E-06 | CDC25A |
| 1.155019 | 2.28E-06 | CTPS1 |
| -1.506234 | 2.28E-06 | DAPK1-IT1 |
| 1.1678565 | 2.28E-06 | ZBTB21 |
| -1.4724445 | 2.36E-06 | SEMA6D |
| 1.142719 | 2.36E-06 | MASTL |
| -1.3041715 | 2.37E-06 | SLIT3 |
| -1.280752 | 2.44E-06 | FAT4 |
| 2.089621 | 2.46E-06 | ANKRD20A5P |
| -1.087093 | 2.49E-06 | NANOG |
| -1.1288765 | 2.49E-06 | FRMPD4 |
| -1.1685068 | 2.49E-06 | FAM134B |
| -1.1988005 | 2.50E-06 | GLI2 |
| -1.136104 | 2.57E-06 | BTN3A3 |
| 1.1731525 | 2.58E-06 | DDX21 |
| 1.2614103 | 2.60E-06 | RHEBL1 |
| 1.1338695 | 2.61E-06 | C7orf57 |
| 1.0753165 | 2.63E-06 | DONSON |
| -1.1501765 | 2.65E-06 | PDZD2 |
| 1.1070783 | 2.71E-06 | GFPT1 |
| 1.1617815 | 2.72E-06 | LOC100289092 |
| 1.1666085 | 2.72E-06 | UPF3B |
| 1.0741513 | 2.80E-06 | LOC101060460 /// POLR3C |
| -1.1237235 | 2.86E-06 | LAMA5 |
| 1.328308 | 2.86E-06 | SHMT2 |
| -1.262794 | 2.86E-06 | ERVFRD-1 |
| -1.35235 | 2.89E-06 | PODXL |
| -1.3248845 | 2.93E-06 | OLFML1 |
| 1.2945755 | 2.95E-06 | CDKN2AIP |
| -1.1862835 | 2.98E-06 | LINC00173 |
| 1.130267 | 3.00E-06 | CHAC1 |
| 1.278457 | 3.02E-06 | CDT1 |
| -1.0655255 | 3.02E-06 | TYRP1 |
| -1.065999 | 3.07E-06 | FOXO6 /// FOXO6 |
| 1.2700925 | 3.08E-06 | SKA1 |
| 1.0810315 | 3.11E-06 | GTPBP4 |
| 1.098338 | 3.14E-06 | HBEGF |
| -1.257127 | 3.20E-06 | LOC150622 /// LOC400940 |
| 2.2411035 | 3.21E-06 | INHBA |
| -1.3861643 | 3.23E-06 | LPHN3 |
| -1.199382 | 3.26E-06 | RNF150 |
| -1.3333173 | 3.26E-06 | CDH1 |
| -1.208869 | 3.27E-06 | COL11A1 |
| -1.3073405 | 3.27E-06 | LOC100507311 |
| 1.115502 | 3.28E-06 | CTB-92J24.2 /// OTTHUMG00000183390 |
| 1.0740958 | 3.29E-06 | KLHDC7B |
| 1.422446 | 3.33E-06 | OTTHUMG00000178878 /// RP11-214C8.5 |
| 1.0845055 | 3.34E-06 | IFI30 /// PIK3R2 |
| 1.3416595 | 3.35E-06 | GCH1 |
| 1.1758325 | 3.38E-06 | ESCO2 |
| -1.112398 | 3.39E-06 | LOC100507303 |
| 1.1847565 | 3.40E-06 | SLC7A11 |
| -1.10736 | 3.48E-06 | VASN |
| 1.892538 | 3.49E-06 | PHLDB2 |
| 1.1753305 | 3.54E-06 | CENPQ |
| 1.106202 | 3.56E-06 | PHYH |
| 1.581823 | 3.57E-06 | HSPBAP1 |
| 1.1277393 | 3.64E-06 | PLAU |
| 1.1904205 | 3.65E-06 | CENPM |
| 1.271799 | 3.68E-06 | PTPRG-AS1 |
| 1.1008765 | 3.84E-06 | THAP9-AS1 |
| -1.0754905 | 3.87E-06 | PGK1 |
| 1.0563545 | 3.89E-06 | PNO1 |
| 1.055013 | 3.89E-06 | POLE2 |
| -1.050091 | 3.90E-06 | LRRC7 |
| 1.051755 | 3.90E-06 | MCM7 |
| -1.0659685 | 3.92E-06 | IGSF9 |
| -1.5674975 | 3.93E-06 | HIST1H2BC |
| 1.1781425 | 3.93E-06 | MND1 |
| -1.16511 | 3.95E-06 | GYS1 |
| -1.2391345 | 3.99E-06 | ZDHHC8P1 |
| 1.2660483 | 4.04E-06 | MOCOS |
| 1.0270553 | 4.05E-06 | RWDD2B |
| 1.015878 | 4.09E-06 | MCM2 |
| 1.0232395 | 4.10E-06 | SLC27A2 |
| -1.2512465 | 4.12E-06 | CENPA /// SLC35F6 |
| 1.0142425 | 4.15E-06 | BLM |
| 1.078636 | 4.16E-06 | HAUS8 |
| -1.146969 | 4.20E-06 | NUDT13 |
| 1.9715898 | 4.21E-06 | HKDC1 |
| 1.0660005 | 4.23E-06 | MCAM |
| -1.2479518 | 4.24E-06 | LOC100996341 |
| -1.0840255 | 4.30E-06 | LOC339535 |
| 1.1326395 | 4.31E-06 | PFDN2 |
| 1.100891 | 4.38E-06 | LOC285084 |
| -1.041091 | 4.39E-06 | MLLT3 |
| 1.0485455 | 4.40E-06 | TUFT1 |
| 1.2974275 | 4.40E-06 | GPT2 |
| -1.1122045 | 4.41E-06 | PTTG1 |
| -1.1931135 | 4.42E-06 | ITIH5 |
| -1.310542 | 4.44E-06 | TOB1-AS1 |
| 1.106642 | 4.45E-06 | NGRN |
| -1.1626908 | 4.53E-06 | TMTC2 |
| 1.116521 | 4.55E-06 | LETM2 |
| 1.1163905 | 4.56E-06 | RGS2 |
| -0.9901925 | 4.60E-06 | GSE1 |
| 1.1983733 | 4.60E-06 | ZNF473 |
| -1.1556205 | 4.61E-06 | FAT3 |
| 1.0523895 | 4.62E-06 | FEN1 |
| 1.252369 | 4.64E-06 | RAD51AP1 |
| 0.999377 | 4.65E-06 | PIGW |
| 0.988329 | 4.67E-06 | C9orf91 |
| -1.096268 | 4.71E-06 | TPM1 |
| 1.0918635 | 4.72E-06 | TYMS |
| 1.0494635 | 4.75E-06 | TUBB2B |
| 1.172292 | 4.81E-06 | TAF1A |
| -1.031285 | 4.82E-06 | EBPL |
| -1.078162 | 4.83E-06 | CAHM |
| 1.093706 | 4.86E-06 | ZC3H8 |
| -1.01875 | 4.89E-06 | SUN2 |
| -0.99651 | 4.92E-06 | ZBED5-AS1 |
| -1.0171075 | 4.94E-06 | RPL31 |
| -1.0516865 | 5.01E-06 | PCDHB5 |
| 1.1534335 | 5.07E-06 | C10orf118 |
| 1.3060475 | 5.15E-06 | RCAN1 |
| 1.1185308 | 5.25E-06 | RFC2 |
| 1.336473 | 5.25E-06 | LOC100506342 |
| 0.9757685 | 5.27E-06 | NOLC1 |
| -0.9804075 | 5.29E-06 | EFEMP1 |
| 1.131335 | 5.29E-06 | SELRC1 |
| 1.0472035 | 5.34E-06 | RFWD3 |
| 0.9905745 | 5.35E-06 | ORC6 |
| 1.066693 | 5.45E-06 | GLRX2 |
| 1.236755 | 5.46E-06 | SDSL |
| -1.014175 | 5.63E-06 | MEIS2 |
| -0.9795765 | 5.72E-06 | SLC16A4 |
| -1.041852 | 5.85E-06 | ATP7B |
| -1.0437105 | 5.89E-06 | C1orf213 |
| 1.005979 | 5.98E-06 | CCNB1IP1 |
| 0.9730778 | 5.99E-06 | SOCS4 |
| 1.0457125 | 6.01E-06 | AUNIP |
| 1.0414268 | 6.02E-06 | LOC81691 |
| 1.0172945 | 6.09E-06 | DNMT1 |
| 1.0131773 | 6.12E-06 | ZWILCH |
| 1.1608325 | 6.14E-06 | RFC3 |
| -0.9605268 | 6.21E-06 | PTPRF |
| -1.0047335 | 6.25E-06 | ANKRD29 |
| 1.1033725 | 6.26E-06 | TK1 |
| 0.999438 | 6.42E-06 | CDC45 |
| 0.9537075 | 6.49E-06 | BRIP1 |
| 0.9839915 | 6.55E-06 | EPT1 |
| 1.0146023 | 6.61E-06 | LINC00467 |
| 1.3381145 | 6.76E-06 | FANCB |
| -0.9801715 | 6.77E-06 | FBXO16 /// ZNF395 |
| 0.955596 | 6.82E-06 | SGK1 |
| 0.9989933 | 6.85E-06 | GAS5 /// SNORD44 /// SNORD47 /// SNORD76 /// SNORD77 /// SNORD79 /// SNORD80 /// SNORD81 |
| -0.9309767 | 6.98E-06 | SLC44A1 |
| -1.2031335 | 6.99E-06 | ARHGEF26 |
| 1.0074488 | 7.00E-06 | TMEM40 |
| 0.9762375 | 7.02E-06 | FAM53C |
| 1.1149123 | 7.12E-06 | SLFN13 |
| 1.583852 | 7.12E-06 | AHR |
| -1.26394 | 7.14E-06 | AGAP1 |
| -0.949338 | 7.19E-06 | FLJ11235 |
| 1.497944 | 7.22E-06 | FST |
| 1.0694175 | 7.33E-06 | C1orf54 |
| 0.9267215 | 7.36E-06 | SUV39H2 |
| 0.9251215 | 7.44E-06 | EZH2 |
| 1.1496688 | 7.44E-06 | RIPK2 |
| -0.961474 | 7.70E-06 | CRYZ |
| -1.0607105 | 7.72E-06 | RAB33A |
| 0.952762 | 7.78E-06 | PDRG1 |
| -0.9227135 | 7.79E-06 | SLITRK5 |
| -1.0551595 | 7.81E-06 | SPA17 |
| -1.482168 | 7.84E-06 | MIR100HG |
| 1.937846 | 7.96E-06 | MXD1 |
| -0.9597855 | 8.07E-06 | CEP70 |
| -1.3354615 | 8.12E-06 | CNTN3 |
| 0.9685055 | 8.12E-06 | RAB39B |
| -1.137332 | 8.25E-06 | ROBO1 |
| -0.9398805 | 8.26E-06 | KCNK5 |
| 0.9262935 | 8.30E-06 | SPRTN |
| 1.11782 | 8.33E-06 | APOL6 |
| -1.129664 | 8.39E-06 | EDIL3 |
| 1.0418665 | 8.43E-06 | PBLD |
| 0.9057272 | 8.47E-06 | MIR1292 /// NOP56 /// SNORD110 /// SNORD57 /// SNORD86 |
| 1.0594105 | 8.58E-06 | ZNF670 |
| -1.38845 | 8.59E-06 | TNFAIP8L1 |
| -0.9431997 | 8.62E-06 | ERGIC1 |
| 0.940025 | 8.67E-06 | ATF4 |
| 1.0708758 | 8.70E-06 | PDP1 |
| 1.020655 | 8.70E-06 | SH2B3 |
| -1.010926 | 8.75E-06 | DDIT4 |
| -0.9512585 | 8.83E-06 | BTN3A2 |
| 0.932206 | 8.84E-06 | MTFR2 |
| 1.0247245 | 8.84E-06 | PNP |
| 0.9042148 | 8.84E-06 | MOSPD1 |
| -1.028785 | 8.88E-06 | ARRB1 |
| -0.9977815 | 9.19E-06 | OTTHUMG00000176821 /// RP11-846E15.4 |
| 1.151465 | 9.30E-06 | CREB5 /// LOC401317 |
| 1.0032905 | 9.33E-06 | SARNP |
| 0.999363 | 9.35E-06 | RELB |
| -1.061367 | 9.38E-06 | GRB14 |
| -1.030493 | 9.46E-06 | LOC151009 /// LOC440894 |
| -0.9064483 | 9.50E-06 | PLCXD1 |
| 0.945754 | 9.55E-06 | PPCDC |
| -0.918733 | 9.56E-06 | COX16 /// SYNJ2BP-COX16 |
| -1.075684 | 9.58E-06 | MIRLET7BHG |
| -1.4412105 | 9.68E-06 | INHBB |
| 1.0531255 | 9.69E-06 | MARS |
| -0.905477 | 9.75E-06 | PAK4 |
| 1.3601895 | 9.76E-06 | LRRN3 |
| 0.8881578 | 9.77E-06 | YARS |
| -0.8914325 | 9.85E-06 | ELFN1 |
| 0.920335 | 9.86E-06 | SRP19 |
| -0.9325915 | 9.99E-06 | PTGIS |
| 0.9287835 | 1.00E-05 | E2F7 |
| -0.903251 | 1.01E-05 | TMED4 |
| 0.9459453 | 1.01E-05 | TRMT6 |
| 1.4499465 | 1.02E-05 | RGS17 |
| 1.316503 | 1.03E-05 | LOC374443 |
| 0.9106975 | 1.04E-05 | RPAP3 |
| 1.167747 | 1.04E-05 | ASNS |
| 0.874115 | 1.08E-05 | CENPH |
| 0.9170415 | 1.09E-05 | GOT1 |
| 0.9786405 | 1.10E-05 | PHIP |
| 0.9028805 | 1.11E-05 | PSMG1 |
| 1.536483 | 1.11E-05 | SNHG8 /// SNORA24 |
| -0.9775 | 1.11E-05 | PSD3 |
| 0.910952 | 1.12E-05 | ITGAM |
| 0.8673375 | 1.12E-05 | RBM15 |
| 0.8904755 | 1.14E-05 | CENPN |
| -0.93271 | 1.14E-05 | ENOX1 |
| 0.8959705 | 1.14E-05 | FANCG |
| -0.920813 | 1.14E-05 | DFFB |
| -0.988336 | 1.14E-05 | BTN3A2 /// BTN3A3 |
| -1.2505295 | 1.16E-05 | BAIAP2-AS1 |
| -0.861449 | 1.17E-05 | CXorf24 |
| 0.8726335 | 1.17E-05 | WDR75 |
| -1.086877 | 1.18E-05 | OTTHUMG00000167487 /// RP11-178H8.7 |
| 0.9204685 | 1.18E-05 | PISD |
| 1.1514355 | 1.18E-05 | RBM24 |
| 0.9607365 | 1.19E-05 | MITF |
| 0.9378372 | 1.19E-05 | TAF13 |
| -1.039543 | 1.22E-05 | C19orf33 |
| -0.8696782 | 1.22E-05 | OTTHUMG00000162476 /// RP11-974F13.6 |
| 0.910765 | 1.22E-05 | NAA50 |
| 0.958404 | 1.22E-05 | TCF19 |
| 1.0280225 | 1.22E-05 | HELLS |
| 1.011237 | 1.23E-05 | CNRIP1 |
| -0.9090707 | 1.23E-05 | MTMR4 |
| 0.970888 | 1.23E-05 | RAB9A |
| 0.895688 | 1.24E-05 | CXCL1 |
| 1.769432 | 1.26E-05 | BEX1 |
| 1.3651095 | 1.27E-05 | TMEM140 |
| 0.854423 | 1.27E-05 | POLA1 |
| -0.8798455 | 1.28E-05 | FBXL7 |
| 0.8735855 | 1.28E-05 | AVPI1 |
| 0.86537 | 1.29E-05 | PCNA |
| 1.226554 | 1.30E-05 | BIK |
| 0.8499855 | 1.31E-05 | MDM1 |
| -0.846934 | 1.32E-05 | ACADSB |
| -1.0411793 | 1.34E-05 | GLS |
| -1.4488365 | 1.34E-05 | SATB1 |
| 1.9785585 | 1.36E-05 | TAC1 |
| 0.935125 | 1.38E-05 | PTP4A1 |
| -0.943581 | 1.38E-05 | GPI |
| 0.8881685 | 1.38E-05 | USP53 |
| -0.8912245 | 1.39E-05 | GULP1 |
| 0.933113 | 1.39E-05 | PTGER4 |
| 0.8968995 | 1.40E-05 | THAP10 |
| 0.9219498 | 1.40E-05 | ULBP2 |
| 1.1641875 | 1.40E-05 | OSBPL6 |
| 0.898289 | 1.41E-05 | PHC3 |
| -0.868273 | 1.41E-05 | OSBPL1A |
| 0.867567 | 1.41E-05 | INPP1 |
| 0.86352 | 1.42E-05 | FAM132B |
| 1.22601 | 1.42E-05 | DUSP3 |
| 0.8470452 | 1.44E-05 | ARL5B |
| 0.951731 | 1.44E-05 | GOLT1B |
| 0.877137 | 1.44E-05 | RMI1 |
| -1.0374005 | 1.45E-05 | TBC1D5 |
| -0.917806 | 1.45E-05 | OXTR |
| -1.3788135 | 1.45E-05 | FSTL1 |
| 0.871035 | 1.46E-05 | SENP1 |
| 0.9141265 | 1.46E-05 | TMEM138 |
| 1.7788795 | 1.47E-05 | C11orf96 |
| 0.831535 | 1.49E-05 | WDR74 |
| -1.3827723 | 1.49E-05 | H19 /// MIR675 |
| -0.9405915 | 1.50E-05 | ROR1 |
| 0.8714782 | 1.51E-05 | RNF138 |
| -1.0918825 | 1.51E-05 | AURKA |
| -0.9033475 | 1.51E-05 | GLIS2 |
| 0.9987555 | 1.51E-05 | BROX |
| 0.8413105 | 1.52E-05 | SWAP70 |
| -0.860698 | 1.53E-05 | CARNS1 |
| -0.8318282 | 1.53E-05 | FA2H |
| 1.090138 | 1.54E-05 | TXNL4B |
| 0.915429 | 1.54E-05 | ABHD3 |
| 1.38782 | 1.55E-05 | USP18 |
| 0.8632265 | 1.56E-05 | PPP1R15B |
| -0.8331468 | 1.57E-05 | ZFX |
| 0.923635 | 1.58E-05 | LRRC57 |
| 1.1926535 | 1.58E-05 | UBIAD1 |
| 0.8809795 | 1.59E-05 | SLC22A15 |
| 1.3455645 | 1.60E-05 | LONRF3 |
| -0.891187 | 1.60E-05 | BCAR3 |
| 0.8314425 | 1.61E-05 | ADAT1 |
| -0.8644873 | 1.61E-05 | LRRC1 |
| 0.856804 | 1.62E-05 | MED10 |
| 0.8317593 | 1.62E-05 | SLC20A1 |
| 0.9122935 | 1.63E-05 | RRS1 |
| 0.862051 | 1.64E-05 | UHRF1 |
| 0.874957 | 1.65E-05 | CYP20A1 |
| -0.984682 | 1.66E-05 | FBXL2 |
| 0.9090995 | 1.67E-05 | SLC16A6 |
| 1.0481905 | 1.68E-05 | SLC6A15 |
| -0.967387 | 1.68E-05 | CRB2 |
| 0.943728 | 1.70E-05 | C16orf93 |
| 0.837132 | 1.70E-05 | MLF1IP |
| -0.8311538 | 1.72E-05 | FAM172A |
| 0.9232545 | 1.72E-05 | GINS3 |
| 1.051791 | 1.72E-05 | KLF6 |
| -0.921502 | 1.73E-05 | TSPAN13 |
| 0.8430585 | 1.73E-05 | MELK |
| 0.8364505 | 1.74E-05 | MIR4723 /// TMEM199 |
| -0.9707245 | 1.74E-05 | FAM49B |
| -0.8167387 | 1.74E-05 | PTPN14 |
| -0.873502 | 1.74E-05 | DTX4 |
| 0.8417955 | 1.76E-05 | URB2 |
| -0.9695257 | 1.76E-05 | LIMCH1 |
| 1.148822 | 1.77E-05 | ELOVL4 |
| 1.222179 | 1.77E-05 | PINX1 |
| 0.8208935 | 1.78E-05 | PRRG4 |
| -0.904663 | 1.79E-05 | SLC39A10 |
| -0.9405675 | 1.79E-05 | SEPT9 |
| 0.8576555 | 1.80E-05 | RAB27A |
| 0.810503 | 1.81E-05 | STIL |
| -0.9347645 | 1.82E-05 | LOC389834 /// MAFIP /// TEKT4P2 |
| -0.87618 | 1.82E-05 | MIR10A |
| -0.837593 | 1.82E-05 | PTTG3P |
| 1.0248913 | 1.83E-05 | ZNF567 |
| -0.834953 | 1.86E-05 | CRELD1 |
| -0.8212993 | 1.86E-05 | SLC40A1 |
| 1.2437235 | 1.87E-05 | ADRB2 |
| 0.826219 | 1.87E-05 | RPA2 |
| -0.8719445 | 1.89E-05 | MN1 |
| 0.9392433 | 1.89E-05 | NRIP3 |
| 0.8263015 | 1.92E-05 | NOP2 |
| -0.8264165 | 1.93E-05 | PGM1 |
| 0.8018108 | 1.93E-05 | CCDC59 |
| 0.8426625 | 1.94E-05 | PHF5A |
| 0.9323885 | 1.94E-05 | FAM24B |
| 0.8381502 | 1.94E-05 | PTPDC1 |
| 0.874561 | 1.94E-05 | BDNF |
| 0.802405 | 1.94E-05 | SRSF7 |
| 0.847512 | 1.96E-05 | NGF |
| 0.8426025 | 1.97E-05 | SENP5 |
| -1.0092423 | 1.97E-05 | CLDN15 |
| 0.857476 | 1.98E-05 | DNAJC1 |
| 1.4060745 | 2.01E-05 | PTPRR |
| 0.803551 | 2.01E-05 | LCP1 |
| 1.133405 | 2.02E-05 | TNFRSF9 |
| 0.8554575 | 2.03E-05 | LOC377711 /// MROH1 |
| -1.122055 | 2.05E-05 | CRLS1 |
| -0.9382045 | 2.05E-05 | C7orf60 |
| -0.823636 | 2.09E-05 | LRRN4 |
| -0.790014 | 2.09E-05 | FIG4 |
| -1.100191 | 2.10E-05 | BCR |
| 0.945882 | 2.11E-05 | FKBP5 |
| 0.810106 | 2.12E-05 | BCAS2 |
| -0.8413288 | 2.12E-05 | BCAR1 |
| -0.8211967 | 2.12E-05 | STON1 |
| 0.985012 | 2.13E-05 | ZNF280C |
| 0.794019 | 2.14E-05 | NIP7 |
| 0.8009445 | 2.15E-05 | RNF166 |
| 1.0981538 | 2.15E-05 | LPL |
| 0.8563858 | 2.16E-05 | AKIP1 |
| 0.7976495 | 2.16E-05 | ZNF682 |
| -0.9315095 | 2.17E-05 | PLLP |
| -0.915717 | 2.18E-05 | ANKH |
| 0.808291 | 2.19E-05 | MRPS31P5 /// THSD1 |
| -1.4071805 | 2.19E-05 | NCKAP5 |
| 0.8111035 | 2.19E-05 | CAMTA2 |
| -0.8218607 | 2.19E-05 | RFX7 |
| 0.840665 | 2.21E-05 | RPL22L1 |
| 0.854031 | 2.21E-05 | EXOC8 |
| 1.0512773 | 2.23E-05 | CCDC15 |
| -0.855059 | 2.24E-05 | NME3 |
| -0.831564 | 2.24E-05 | C10orf114 |
| -0.958172 | 2.25E-05 | ZMYND8 |
| 1.7022825 | 2.25E-05 | SLC25A25 |
| -0.8042793 | 2.26E-05 | NIPSNAP1 |
| 0.781837 | 2.28E-05 | RNF25 |
| -0.9535345 | 2.28E-05 | OTTHUMG00000170856 /// RP11-33E12.2 |
| -0.808404 | 2.29E-05 | MSI2 |
| 0.779643 | 2.29E-05 | MAP1LC3B |
| 0.8446665 | 2.30E-05 | NUP153 |
| 1.1246755 | 2.30E-05 | RNMTL1 |
| 0.7956045 | 2.31E-05 | ZCCHC8 |
| 0.88581 | 2.31E-05 | IFI16 |
| -0.926838 | 2.35E-05 | UNC5B-AS1 |
| -0.777197 | 2.36E-05 | AKR1C3 |
| 1.0841935 | 2.37E-05 | TLR3 |
| 0.8020345 | 2.37E-05 | NABP1 |
| 1.0947095 | 2.38E-05 | GDAP1 |
| 0.780396 | 2.40E-05 | ZNF655 |
| -0.9665555 | 2.40E-05 | ZNF804A |
| 0.8365595 | 2.41E-05 | CSRP2 |
| 0.81023 | 2.41E-05 | USP37 |
| 0.8427612 | 2.43E-05 | JMY |
| 0.8628215 | 2.43E-05 | RRN3 |
| 0.8328735 | 2.43E-05 | LOC100509445 /// LOC728715 /// OVOS2 |
| -0.821288 | 2.43E-05 | TMEM256 |
| 0.7995205 | 2.44E-05 | BRCA1 |
| -0.8328555 | 2.46E-05 | LRRFIP1 |
| -0.8363085 | 2.46E-05 | PRKAG2 |
| -0.8893625 | 2.47E-05 | PLEKHA2 |
| 1.3077345 | 2.47E-05 | MICB |
| -0.8828535 | 2.48E-05 | ANXA8 /// LOC100996760 /// LOC101060462 |
| 1.1879655 | 2.48E-05 | RTP4 |
| -1.107059 | 2.50E-05 | DOK7 |
| -0.9951345 | 2.51E-05 | UPK3B |
| 0.82654 | 2.54E-05 | GJC1 |
| 0.8435625 | 2.57E-05 | TRMT1 |
| 0.8903645 | 2.58E-05 | RPF2 |
| -0.997485 | 2.59E-05 | OTTHUMG00000175814 /// RP11-13L2.4 |
| -0.794883 | 2.59E-05 | LOC100287896 |
| 0.845124 | 2.60E-05 | IL4I1 |
| 0.8386788 | 2.60E-05 | SDC1 |
| -0.76567 | 2.60E-05 | ZNF618 |
| 0.785755 | 2.61E-05 | POLE |
| -0.902451 | 2.61E-05 | RPS15A |
| -0.9215725 | 2.63E-05 | TMEM191A |
| 0.7620775 | 2.67E-05 | CCDC137 |
| -0.8790395 | 2.68E-05 | CCNB1 |
| 0.866184 | 2.70E-05 | POLR3F |
| -0.801301 | 2.71E-05 | ABCA3 |
| -0.771608 | 2.72E-05 | LDOC1 |
| -0.791333 | 2.72E-05 | CCNG2 |
| -0.820789 | 2.74E-05 | FAM83D |
| 0.956156 | 2.75E-05 | ARNTL2 |
| -0.892747 | 2.76E-05 | YPEL2 |
| 0.7640715 | 2.78E-05 | MGME1 |
| -0.8259845 | 2.78E-05 | AK9 |
| 0.8244123 | 2.79E-05 | DDX20 |
| -1.1295505 | 2.79E-05 | PROC |
| 0.7976765 | 2.81E-05 | BRIX1 |
| 0.823991 | 2.83E-05 | SKA3 |
| -0.760564 | 2.86E-05 | MYH9 |
| 0.817186 | 2.87E-05 | EID3 |
| 0.7876835 | 2.87E-05 | NFXL1 |
| 0.888988 | 2.88E-05 | EIF4EBP1 |
| -0.8860965 | 2.88E-05 | MUS81 |
| 0.905245 | 2.89E-05 | SERTAD1 |
| 0.795638 | 2.89E-05 | TUBB2A |
| 2.659527 | 2.89E-05 | PTGS2 |
| 1.0440205 | 2.90E-05 | MYBL2 |
| 0.7749125 | 2.90E-05 | ATP2B1 |
| 0.7591638 | 2.90E-05 | ZFR |
| 0.8345815 | 2.90E-05 | IL18 |
| 0.83508 | 2.91E-05 | IRF1 |
| -0.77516 | 2.92E-05 | S100A10 |
| -0.8755885 | 2.92E-05 | S100A4 |
| 0.763047 | 2.96E-05 | SRPRB |
| 0.8043545 | 2.97E-05 | NCAPG2 |
| 0.807864 | 2.97E-05 | CCDC86 |
| 0.7961723 | 2.99E-05 | ARHGEF2 |
| 0.7780705 | 3.00E-05 | IWS1 |
| 0.8961225 | 3.03E-05 | SRD5A1 |
| -0.7714707 | 3.05E-05 | FAM168A |
| 1.3942175 | 3.06E-05 | RRAD |
| 0.840357 | 3.07E-05 | SNX8 |
| -1.3140735 | 3.07E-05 | C4orf3 |
| 1.020376 | 3.08E-05 | EDN1 |
| 0.832592 | 3.17E-05 | CHAF1A |
| -1.19214 | 3.18E-05 | EXT1 |
| 0.883361 | 3.19E-05 | NOC3L |
| 1.117666 | 3.22E-05 | DSN1 |
| 0.760297 | 3.23E-05 | FGFR1OP |
| -1.0677558 | 3.24E-05 | SHB |
| -0.840971 | 3.24E-05 | NRDE2 |
| -1.0743925 | 3.25E-05 | CNTN5 |
| -0.8790015 | 3.25E-05 | CBX7 |
| 0.8272368 | 3.25E-05 | SFR1 |
| -0.7571273 | 3.29E-05 | PDGFC |
| 0.77062 | 3.30E-05 | ZWINT |
| 0.7771435 | 3.31E-05 | PCK2 |
| 0.7740415 | 3.32E-05 | MICALL1 |
| 1.0822645 | 3.33E-05 | TNFSF15 |
| 0.9096075 | 3.34E-05 | DUSP5 |
| 0.7676865 | 3.35E-05 | SMIM13 |
| 0.9616085 | 3.36E-05 | XAF1 |
| 1.042934 | 3.38E-05 | LRRC49 |
| 0.775259 | 3.38E-05 | NXT1 |
| 1.3026625 | 3.38E-05 | IFIT2 |
| 0.818253 | 3.43E-05 | OTTHUMG00000018458 /// RP11-152N13.5 |
| 0.790482 | 3.43E-05 | TRIM26 |
| -0.752832 | 3.43E-05 | SASH1 |
| 0.762831 | 3.44E-05 | JPH1 |
| 0.796937 | 3.44E-05 | NEDD4 |
| 0.7518535 | 3.45E-05 | CMC2 |
| 1.021986 | 3.46E-05 | TTBK2 |
| -0.893057 | 3.46E-05 | OTTHUMG00000167230 /// RP11-736K20.4 |
| 0.740333 | 3.47E-05 | NMD3 |
| 0.802856 | 3.49E-05 | P2RX7 |
| -0.7662275 | 3.51E-05 | ST3GAL1 |
| -0.7479602 | 3.52E-05 | CASP6 |
| 0.750831 | 3.53E-05 | RIOK3 |
| 0.7321607 | 3.55E-05 | CIRH1A |
| -0.764793 | 3.56E-05 | RBM38 |
| 0.937765 | 3.56E-05 | ZFAS1 |
| 0.7949515 | 3.58E-05 | SCLT1 |
| 0.7633585 | 3.59E-05 | KNTC1 |
| 0.9401138 | 3.62E-05 | PTGES |
| 0.7453578 | 3.63E-05 | LYAR |
| 0.740234 | 3.64E-05 | CENPO |
| 0.780081 | 3.65E-05 | GGCT |
| -0.7465865 | 3.67E-05 | CENPE |
| -1.726822 | 3.71E-05 | GPM6A |
| 0.7542685 | 3.73E-05 | PALB2 |
| 0.7407993 | 3.74E-05 | FBXO5 |
| -0.8149545 | 3.75E-05 | LYPD6B |
| 0.7498947 | 3.75E-05 | TIMELESS |
| -0.7401015 | 3.75E-05 | LOC692247 |
| -0.786325 | 3.76E-05 | CXXC5 |
| 0.817165 | 3.77E-05 | CHAF1B |
| 1.000034 | 3.78E-05 | FANCI |
| 1.1246293 | 3.80E-05 | FLT1 |
| 0.7664355 | 3.83E-05 | PPTC7 |
| 1.318085 | 3.85E-05 | PTHLH |
| 0.731395 | 3.85E-05 | RIT1 |
| -1.02236 | 3.86E-05 | TENM2 |
| 0.75619 | 3.86E-05 | USP36 |
| 0.945113 | 3.89E-05 | FAM86B1 /// FAM86B2 /// FAM86C1 /// FAM86DP /// FAM86FP |
| -0.7799903 | 3.90E-05 | WDPCP |
| -0.7213338 | 3.91E-05 | TBCE |
| -0.971077 | 3.92E-05 | FNTB |
| -1.335915 | 3.93E-05 | CAPN6 |
| 0.7587628 | 3.96E-05 | USP31 |
| 0.7646243 | 3.98E-05 | SDE2 |
| -0.7558555 | 4.01E-05 | TLDC1 |
| 1.342326 | 4.02E-05 | LOC100506377 |
| 0.746493 | 4.02E-05 | ZNF331 |
| -1.109747 | 4.02E-05 | PPP1R3C |
| -0.782445 | 4.04E-05 | NPEPPS |
| 0.7362707 | 4.07E-05 | DDR2 |
| -0.766536 | 4.07E-05 | CA11 |
| 0.7331105 | 4.10E-05 | LOC284023 |
| -0.72675 | 4.10E-05 | TPRN |
| 0.810769 | 4.11E-05 | GEN1 |
| 0.798619 | 4.13E-05 | NUPL1 |
| 0.8695703 | 4.13E-05 | KIAA0101 |
| 0.8230685 | 4.14E-05 | LOC100131067 |
| -0.711668 | 4.15E-05 | NRBP2 |
| 0.768457 | 4.15E-05 | CREM |
| 0.8696135 | 4.16E-05 | C3orf52 |
| 0.829577 | 4.18E-05 | LOC100506392 |
| 0.8010805 | 4.19E-05 | ZCCHC10 |
| -0.7459708 | 4.20E-05 | BAIAP2 |
| 0.8859887 | 4.26E-05 | MAFF |
| 0.7557185 | 4.28E-05 | XK |
| -0.754751 | 4.28E-05 | HEG1 |
| 0.71936 | 4.32E-05 | LENG1 |
| -0.771078 | 4.34E-05 | FZD2 |
| 1.210992 | 4.35E-05 | TNFAIP3 |
| 0.7523355 | 4.35E-05 | SURF2 |
| -0.7245225 | 4.39E-05 | WASF3 |
| 0.7613055 | 4.40E-05 | CTSL2 |
| -0.8715583 | 4.40E-05 | C1GALT1C1 |
| -0.7461215 | 4.42E-05 | LOC254057 |
| 0.7121175 | 4.43E-05 | RIPPLY3 |
| 0.72855 | 4.45E-05 | LRRC58 |
| 0.7185747 | 4.48E-05 | FANCA |
| -0.874997 | 4.49E-05 | IL7 |
| 0.729279 | 4.49E-05 | RMI2 |
| 0.9151438 | 4.54E-05 | ECM2 |
| 1.612791 | 4.54E-05 | CXCL10 |
| 0.8672958 | 4.55E-05 | GRPEL2 |
| 1.0155315 | 4.58E-05 | EIF3C |
| 0.7008625 | 4.59E-05 | COL16A1 |
| 0.7266485 | 4.61E-05 | SLC1A4 |
| -0.7147865 | 4.62E-05 | MED11 |
| 1.000789 | 4.63E-05 | RASEF |
| 0.737023 | 4.64E-05 | NDUFAF2 |
| 0.714235 | 4.66E-05 | RAB23 |
| -0.746325 | 4.67E-05 | CTB-31O20.2 /// OTTHUMG00000175708 |
| 0.7880045 | 4.68E-05 | CASP7 |
| -0.705148 | 4.68E-05 | SEMA3F |
| -0.9374335 | 4.70E-05 | SORL1 |
| 0.7036752 | 4.74E-05 | REV3L |
| 0.770651 | 4.76E-05 | AIMP2 |
| 0.7372312 | 4.80E-05 | FAS |
| 0.7088165 | 4.80E-05 | HAT1 |
| 0.7473872 | 4.81E-05 | RABGGTB /// SNORD45A /// SNORD45B /// SNORD45C |
| -0.714271 | 4.83E-05 | JARID2 |
| -0.8228455 | 4.85E-05 | SEMA3C |
| -0.702844 | 4.87E-05 | BCHE |
| -0.7214845 | 4.88E-05 | LOC100505715 |
| 1.257731 | 4.88E-05 | PLA2G4A |
| 0.7236345 | 4.90E-05 | ZNF259 /// ZNF259P1 |
| 0.7053883 | 4.90E-05 | DSCC1 |
| 1.026869 | 4.90E-05 | P2RX4 |
| -0.8976822 | 4.90E-05 | LOC100507577 /// LONP2 |
| -0.883067 | 4.94E-05 | ITGA3 |
| 0.777346 | 4.96E-05 | TMEM47 |
| 0.6939153 | 4.97E-05 | CCDC93 |
| 0.7900313 | 5.00E-05 | SRA1 |
| 0.8236745 | 5.01E-05 | PKMYT1 |
| 0.9743885 | 5.02E-05 | C1QTNF9B-AS1 |
| 0.7679345 | 5.03E-05 | POLE3 |
| -0.8484595 | 5.03E-05 | TAGLN |
| -0.9147613 | 5.04E-05 | MICAL2 |
| -0.6946253 | 5.06E-05 | OTTHUMG00000176823 /// RP11-846E15.2 |
| -0.7384695 | 5.07E-05 | PLIN3 |
| 0.915658 | 5.09E-05 | PGBD1 |
| 0.691879 | 5.09E-05 | MMD |
| 0.7607915 | 5.12E-05 | NAF1 |
| -0.72981 | 5.14E-05 | DCLK2 |
| 1.025609 | 5.17E-05 | PNPT1 |
| -0.7816025 | 5.19E-05 | CAV2 |
| 0.7298725 | 5.19E-05 | ZNF443 |
| -1.4906008 | 5.22E-05 | BNIP3 |
| -0.695026 | 5.22E-05 | LOC101060235 /// TMSB15A /// TMSB15B |
| -1.029273 | 5.23E-05 | ATG4C |
| 0.792771 | 5.23E-05 | TRIM35 |
| -0.7011275 | 5.23E-05 | OTTHUMG00000177151 /// RP11-715J22.6 |
| 0.816689 | 5.29E-05 | FAM50A |
| 0.8704525 | 5.30E-05 | RARRES3 |
| -0.967501 | 5.31E-05 | FAM64A |
| -0.800084 | 5.31E-05 | TKT |
| -0.69594 | 5.32E-05 | ANXA8 /// ANXA8L1 /// ANXA8L2 |
| -0.8064663 | 5.39E-05 | STOX2 |
| -1.1231815 | 5.39E-05 | SSBP3 |
| -0.6975705 | 5.39E-05 | ATXN10 |
| 0.7949255 | 5.40E-05 | BUD13 |
| -0.87083 | 5.47E-05 | KRT8 |
| 0.7716955 | 5.48E-05 | C1orf109 |
| 0.7176575 | 5.49E-05 | ZNF311 |
| -0.692047 | 5.49E-05 | COL18A1 |
| 0.7024075 | 5.50E-05 | PSME3 |
| 1.0212795 | 5.52E-05 | SPHK1 |
| 0.6820357 | 5.52E-05 | FIP1L1 |
| -0.8484845 | 5.54E-05 | ZNF395 |
| 0.8186538 | 5.54E-05 | LOC100129518 /// SOD2 |
| 0.692827 | 5.55E-05 | NUFIP1 |
| 1.4558263 | 5.58E-05 | KLF4 |
| 0.7125705 | 5.58E-05 | LPIN2 |
| 0.7551655 | 5.58E-05 | PASK |
| 0.7159185 | 5.61E-05 | RABGGTB |
| -0.913032 | 5.64E-05 | GPSM2 |
| 1.1944785 | 5.66E-05 | DACT1 |
| -0.8538845 | 5.68E-05 | ERBB4 |
| 0.7426695 | 5.69E-05 | MYC |
| -0.930906 | 5.70E-05 | ARL15 |
| 0.7592075 | 5.70E-05 | RECQL |
| 0.7583395 | 5.72E-05 | WTAP |
| 1.061939 | 5.73E-05 | TCP11L1 |
| 1.422974 | 5.73E-05 | DLGAP1-AS2 |
| 0.676968 | 5.74E-05 | TOPORS |
| -1.0092595 | 5.75E-05 | NFIA |
| 0.7517755 | 5.75E-05 | SLC25A28 |
| -0.697648 | 5.75E-05 | E2F5 |
| -0.965658 | 5.76E-05 | NREP |
| -0.6934035 | 5.81E-05 | HIST1H2BH |
| -0.7012258 | 5.85E-05 | C3orf70 |
| 0.7506427 | 5.85E-05 | TNPO1 |
| -0.6866878 | 5.86E-05 | PEX11A |
| 0.8986953 | 5.89E-05 | FAM111A |
| 0.9439 | 5.90E-05 | C12orf44 |
| 0.700465 | 5.90E-05 | ZNF689 |
| 0.7007645 | 5.97E-05 | MRPS31 |
| 0.781199 | 5.99E-05 | RNF114 |
| -0.785936 | 5.99E-05 | SETBP1 |
| -0.8240293 | 6.02E-05 | CTIF |
| 1.0439355 | 6.06E-05 | OAS2 |
| 1.6778915 | 6.07E-05 | CXCL11 |
| 0.7389963 | 6.07E-05 | MCM8 |
| -0.8436695 | 6.08E-05 | MCC |
| -0.754828 | 6.08E-05 | MIR181A2HG |
| 0.7309005 | 6.10E-05 | LRR1 |
| -0.993508 | 6.11E-05 | HIST2H2BE |
| -0.681972 | 6.11E-05 | DBP |
| 0.6881435 | 6.11E-05 | GYG1 |
| 0.696676 | 6.12E-05 | SBDS /// SBDSP1 |
| -0.7406962 | 6.15E-05 | TSPAN31 |
| 1.104707 | 6.21E-05 | SLC19A2 |
| -1.273703 | 6.23E-05 | FAM162A |
| 0.6951055 | 6.25E-05 | RUSC1 |
| -0.735077 | 6.25E-05 | OSER1-AS1 |
| -0.8482115 | 6.26E-05 | C1orf21 |
| 0.856513 | 6.26E-05 | NFIL3 |
| -0.9238815 | 6.28E-05 | LINC00521 |
| -0.7644567 | 6.30E-05 | YY1 |
| 0.7059265 | 6.31E-05 | COQ10A |
| 0.6924635 | 6.31E-05 | SLC25A19 |
| 0.770528 | 6.33E-05 | CD83 |
| 1.426482 | 6.35E-05 | HRK /// LOC283454 |
| 0.812605 | 6.35E-05 | NMI |
| -0.6794065 | 6.35E-05 | DOCK4 |
| 0.8078313 | 6.44E-05 | PHLDA1 |
| 0.7556105 | 6.45E-05 | NUP155 |
| -0.996888 | 6.51E-05 | CSMD3 |
| 0.6714153 | 6.56E-05 | POLQ |
| 0.69031 | 6.57E-05 | HSPA9 |
| -0.808669 | 6.59E-05 | TTC28 |
| 0.68072 | 6.66E-05 | TRAPPC6B |
| -0.6824115 | 6.68E-05 | CAT |
| 0.672357 | 6.71E-05 | MAK16 |
| -1.283287 | 6.76E-05 | PLK1 |
| -0.8230885 | 6.77E-05 | ALPK2 |
| 0.660312 | 6.78E-05 | TCOF1 |
| 0.667195 | 6.79E-05 | VRK1 |
| 1.3223598 | 6.80E-05 | OASL |
| -0.7132585 | 6.82E-05 | LINC00685 |
| 0.8137325 | 6.83E-05 | LOC100506714 |
| 0.746502 | 6.84E-05 | POLD3 |
| 0.749941 | 6.84E-05 | LAMA1 |
| 0.7533327 | 6.85E-05 | SVIP |
| -0.7535185 | 6.86E-05 | PLGLB1 /// PLGLB2 |
| -0.706387 | 6.88E-05 | ST3GAL5 |
| 0.688918 | 6.88E-05 | C1orf112 |
| -0.7243075 | 6.90E-05 | GRAMD4 |
| 0.7943525 | 6.93E-05 | MAP1B |
| -0.670197 | 6.93E-05 | PEX2 |
| 0.7479825 | 6.95E-05 | ERI1 |
| -0.7655825 | 6.95E-05 | RSBN1 |
| 0.8080112 | 6.98E-05 | ZNF697 |
| -0.6961525 | 7.00E-05 | LOC100507054 |
| 0.7198205 | 7.00E-05 | PAK1IP1 |
| 0.688063 | 7.04E-05 | GMNN |
| -0.6957985 | 7.05E-05 | FSTL3 |
| -0.750507 | 7.11E-05 | ATP2B4 |
| -0.7368465 | 7.12E-05 | SELENBP1 |
| -0.7225695 | 7.12E-05 | IPW /// LOC100506948 /// SNORD107 /// SNORD115-13 /// SNORD115-26 /// SNORD115-7 /// SNORD116-28 /// SNRPN |
| -0.7297562 | 7.15E-05 | SERPINB1 |
| 0.769224 | 7.17E-05 | USP1 |
| 0.7610725 | 7.19E-05 | ZNF441 |
| -0.7804995 | 7.27E-05 | ISOC2 |
| -0.7165815 | 7.28E-05 | COL4A6 |
| -1.3118775 | 7.29E-05 | B3GALT2 |
| 0.7007445 | 7.30E-05 | CRY1 |
| -1.332717 | 7.35E-05 | FKBP7 |
| 0.6527293 | 7.37E-05 | FKRP |
| -1.061253 | 7.38E-05 | LOC151009 |
| 0.828914 | 7.42E-05 | CENPJ |
| 0.6811023 | 7.42E-05 | CA13 /// LOC100507258 |
| 0.7089715 | 7.44E-05 | LOC442075 |
| 0.6895138 | 7.45E-05 | S1PR3 |
| 0.684611 | 7.46E-05 | ABRACL |
| 0.678914 | 7.53E-05 | CLIP1 |
| 0.6674155 | 7.58E-05 | EXOSC8 |
| -1.02518 | 7.59E-05 | CLIC3 |
| 0.6831045 | 7.62E-05 | LOC100996643 /// MTHFD1L |
| -0.808361 | 7.64E-05 | ST6GALNAC5 |
| -0.8048458 | 7.65E-05 | KIF14 |
| 0.6694775 | 7.67E-05 | AZIN1 |
| 0.6698235 | 7.68E-05 | UAP1L1 |
| 0.7612885 | 7.69E-05 | ANKRD20A1 /// ANKRD20A11P /// ANKRD20A2 /// ANKRD20A3 /// ANKRD20A4 /// ANKRD20A5P /// ANKRD20A9P /// LOC101059935 |
| -1.4550393 | 7.69E-05 | MIR210HG |
| 0.878777 | 7.69E-05 | ATAD5 |
| -0.669425 | 7.72E-05 | PKM |
| 1.5202393 | 7.76E-05 | ISG20 |
| 0.680803 | 7.77E-05 | HAUS3 |
| -0.647721 | 7.79E-05 | GAS1 |
| 0.8323325 | 7.79E-05 | ZNF257 |
| -0.6639575 | 7.82E-05 | ANXA4 |
| -0.8248357 | 7.82E-05 | TPD52L1 |
| -0.7418845 | 7.83E-05 | TOX2 |
| 0.6597255 | 7.88E-05 | NET1 |
| -0.6867875 | 7.93E-05 | HSD17B2 |
| -0.707139 | 7.97E-05 | OTTHUMG00000019884 /// RP11-112J3.16 |
| 0.8764425 | 8.00E-05 | MIER3 |
| -0.8879625 | 8.00E-05 | CA9 |
| -0.6651485 | 8.07E-05 | ANKRD13C |
| -0.7894665 | 8.12E-05 | FAM19A5 |
| -0.8682175 | 8.13E-05 | LOC100507486 |
| 0.7031113 | 8.13E-05 | C12orf4 |
| -0.6504085 | 8.13E-05 | CTD-2336O2.1 /// OTTHUMG00000163625 |
| -0.710418 | 8.21E-05 | TTC30B |
| 0.7060855 | 8.22E-05 | DR1 |
| 0.6629645 | 8.25E-05 | TSR1 |
| 0.7017395 | 8.27E-05 | NDUFS3 /// PTPMT1 |
| 0.743768 | 8.28E-05 | GAN |
| -0.646501 | 8.29E-05 | GMDS |
| 0.6835935 | 8.30E-05 | ZNF625 /// ZNF625-ZNF20 |
| 0.6559727 | 8.30E-05 | FIGNL1 |
| -0.9067765 | 8.34E-05 | ALDH6A1 |
| -0.6998655 | 8.36E-05 | TPD52 |
| 0.644277 | 8.40E-05 | SLU7 |
| -0.812565 | 8.43E-05 | MIR4800 /// MXD4 |
| 0.737265 | 8.44E-05 | SLC25A32 |
| -0.6385155 | 8.45E-05 | NACC2 |
| -0.906372 | 8.49E-05 | TET1 |
| -0.7829575 | 8.51E-05 | PHACTR1 |
| -0.668776 | 8.51E-05 | LOC100507316 |
| -1.4138385 | 8.52E-05 | RNFT2 |
| 0.6388128 | 8.56E-05 | UTP23 |
| 0.663373 | 8.57E-05 | UTP15 |
| -0.644705 | 8.57E-05 | SPESP1 |
| -0.6562878 | 8.58E-05 | ITPR2 |
| 0.644955 | 8.59E-05 | C2orf49 |
| -1.0394325 | 8.63E-05 | CENPA |
| 0.762041 | 8.64E-05 | NEIL3 |
| 1.1867803 | 8.65E-05 | ADAMTS5 |
| 0.704787 | 8.67E-05 | MCM5 |
| 0.674263 | 8.72E-05 | ZBTB43 |
| -0.7600015 | 8.74E-05 | PTPN13 |
| 0.869512 | 8.76E-05 | PSPC1 |
| 0.779525 | 8.83E-05 | KPNA4 |
| 0.760374 | 8.84E-05 | LUC7L |
| -1.1244625 | 8.86E-05 | TTC30A |
| 0.6602035 | 8.87E-05 | MAD2L1BP |
| 0.830733 | 8.88E-05 | ORC1 |
| 0.8680125 | 8.89E-05 | PPRC1 |
| -0.9271285 | 8.89E-05 | PPL |
| -1.0041175 | 8.93E-05 | LOC100506990 |
| 0.7133673 | 8.96E-05 | BAZ1A |
| -0.70263 | 8.99E-05 | LOC101060527 /// NAIP |
| 0.732807 | 9.01E-05 | GINS4 |
| 0.6486805 | 9.05E-05 | TMEM181 |
| -0.6622503 | 9.08E-05 | NPNT |
| 0.6522525 | 9.08E-05 | KRCC1 |
| 0.7377785 | 9.11E-05 | ORC5 |
| 0.796552 | 9.20E-05 | HES6 |
| 0.6289785 | 9.21E-05 | POLR3D |
| 0.838851 | 9.22E-05 | SSSCA1 |
| -0.6787598 | 9.27E-05 | WT1 |
| 0.9569985 | 9.28E-05 | IFI35 |
| 0.6743815 | 9.29E-05 | EIF6 |
| 0.7085437 | 9.30E-05 | CCNT1 |
| 0.8345435 | 9.32E-05 | DIO2 |
| -0.6791953 | 9.32E-05 | SMAD3 |
| -0.715099 | 9.37E-05 | DHRS3 |
| 1.4719493 | 9.41E-05 | TNFAIP6 |
| 0.670886 | 9.42E-05 | UBR7 |
| 0.655823 | 9.43E-05 | PCGF6 |
| 1.0050005 | 9.45E-05 | HERC5 |
| 0.6291303 | 9.49E-05 | NSUN5 |
| 0.672946 | 9.53E-05 | PALLD |
| 0.6509595 | 9.55E-05 | OSBPL11 |
| -0.950786 | 9.61E-05 | LAMB1 |
| -0.6835025 | 9.62E-05 | ARHGAP18 |
| -1.093109 | 9.64E-05 | HIST1H2AC |
| -0.730284 | 9.67E-05 | DIP2C |
| 0.8067995 | 9.69E-05 | MCM4 |
| 0.6565125 | 9.70E-05 | XPO5 |
| 0.676975 | 9.71E-05 | ZNF326 |
| 0.7319862 | 9.71E-05 | SLC7A6OS |
| 0.758214 | 9.71E-05 | LOC100506661 |
| 0.6488068 | 9.73E-05 | CAND2 |
| -0.666655 | 9.74E-05 | HCN1 |
| 0.8996555 | 9.76E-05 | ANKLE1 |
| -1.0248475 | 9.81E-05 | OTTHUMG00000180314 /// RP1-193H18.2 |
| -0.6801592 | 9.86E-05 | TMEM161B-AS1 |
| 0.6313633 | 9.91E-05 | SAMD4A |
| -1.4307355 | 9.91E-05 | KIAA1984 |
| -0.85412 | 9.95E-05 | SULF1 |
| -0.6595 | 9.96E-05 | THRA |
| -0.8899167 | 0.0001 | THNSL1 |
| -0.779541 | 0.0001 | COL5A1 |
| -0.6699605 | 0.0001 | ACTN4 |
| 0.7509865 | 0.0001 | SAMHD1 |
| -0.630515 | 0.0001 | CHRNB1 |
| -0.6212917 | 0.0001 | SLCO3A1 |
| -0.704727 | 0.0001 | MYOF |
| 0.914025 | 0.0001 | SHOX2 |
| 0.653174 | 0.0001 | FDX1L |
| 0.7184135 | 0.0001 | CDC7 |
| 0.62552 | 0.0001 | MTPAP |
| -0.624916 | 0.0001 | ADAMTS3 |
| -1.2666495 | 0.0001 | DUSP5P1 |
| 0.6247935 | 0.0001 | CIDEC |
| -0.78361 | 0.0001 | SLC7A7 |
| 0.6284125 | 0.0001 | ARF6 |
| 0.6373775 | 0.0001 | BEX2 |
| 0.6446487 | 0.0001 | YRDC |
| 0.642767 | 0.0001 | CDK11A /// CDK11B |
| 0.7262848 | 0.0001 | CYP1B1 |
| 1.0717115 | 0.0001 | IFIT3 |
| 0.6784815 | 0.0001 | AMIGO2 |
| 0.641318 | 0.0001 | PPIG |
| 0.637945 | 0.00011 | COPS3 |
| 0.637948 | 0.00011 | IFRD2 |
| -0.6426225 | 0.00011 | HCFC1R1 |
| 0.773874 | 0.00011 | CDCA7 |
| 0.6165663 | 0.00011 | CDC23 |
| 0.754131 | 0.00011 | ZNF280B |
| 0.6266542 | 0.00011 | RGMB |
| 0.652984 | 0.00011 | YKT6 |
| 0.7275175 | 0.00011 | ZNHIT2 |
| 0.7108738 | 0.00011 | BIRC3 |
| -0.7169895 | 0.00011 | USP48 |
| 0.656888 | 0.00011 | TIMM44 |
| -0.6714668 | 0.00011 | GALNS |
| -0.6281895 | 0.00011 | CRIP2 |
| -0.637891 | 0.00011 | BMPR1A |
| 0.649289 | 0.00011 | LMBRD2 |
| -0.6596025 | 0.00011 | KIAA1522 |
| -0.931303 | 0.00011 | GBE1 |
| 0.768973 | 0.00011 | C19orf48 /// SNORD88C |
| 0.870347 | 0.00011 | SLC30A1 |
| -0.6712308 | 0.00011 | PDPN |
| 0.614028 | 0.00011 | ZFP3 |
| 0.6279325 | 0.00011 | CD55 |
| 0.7297747 | 0.00011 | SEC22B |
| -1.1537765 | 0.00011 | FOS |
| 0.674898 | 0.00011 | NCAPD3 |
| 0.6544745 | 0.00011 | MBD4 |
| 0.7986625 | 0.00011 | RAD51 |
| 0.6215455 | 0.00011 | FBXO45 |
| -0.6461355 | 0.00011 | LMBRD1 |
| -1.056184 | 0.00011 | BCL11A |
| 0.6173835 | 0.00011 | GINS1 |
| -0.688874 | 0.00011 | PLOD1 |
| -0.697826 | 0.00011 | CFH /// CFHR1 |
| -0.7230848 | 0.00011 | ZBTB20 |
| 0.7645227 | 0.00012 | SMC6 |
| -0.905578 | 0.00012 | WT1-AS |
| -0.7400985 | 0.00012 | CTSH |
| -0.6700745 | 0.00012 | CDC20 |
| 0.623523 | 0.00012 | MRPL37 |
| 0.6086972 | 0.00012 | CCNE1 |
| 0.6272735 | 0.00012 | YTHDF1 |
| 0.6388745 | 0.00012 | UAP1 |
| -0.6713475 | 0.00012 | KBTBD7 |
| 0.623519 | 0.00012 | SPC25 |
| -0.9906657 | 0.00012 | C10orf54 |
| 0.6923645 | 0.00012 | TAF4B |
| 0.6879585 | 0.00012 | ZNF23 |
| 0.74144 | 0.00012 | MYO3A |
| 0.7556335 | 0.00012 | MSH2 |
| -0.7067935 | 0.00012 | MRPS28 |
| -0.6487377 | 0.00012 | PARD3 |
| 0.6684885 | 0.00012 | IER5 |
| 1.229394 | 0.00012 | SAT1 |
| 0.91058 | 0.00012 | PHGDH |
| 0.7295385 | 0.00012 | TFAM |
| 0.653379 | 0.00012 | SMCO4 |
| 0.603214 | 0.00012 | MID1IP1 |
| 0.927326 | 0.00012 | OTTHUMG00000183927 /// RP11-248J18.2 |
| 0.736017 | 0.00012 | TMEM154 |
| 0.721192 | 0.00012 | COCH |
| 0.6223335 | 0.00012 | SUGT1 |
| 0.661325 | 0.00012 | ZNF259 |
| 0.7376665 | 0.00012 | RAD54L |
| 0.6027297 | 0.00012 | LOC101060478 /// RNF115 |
| -0.6506853 | 0.00012 | GAS6 |
| 0.616556 | 0.00012 | TCHP |
| -0.8102245 | 0.00012 | RAB11FIP4 |
| 0.6656755 | 0.00012 | ETHE1 |
| -0.638572 | 0.00013 | MORF4L2-AS1 |
| 0.879053 | 0.00013 | RASSF1 |
| -0.6201835 | 0.00013 | AMOTL2 |
| 0.626052 | 0.00013 | ATR |
| -0.6317192 | 0.00013 | MEGF6 |
| -0.742297 | 0.00013 | TMCC1 |
| -0.60433 | 0.00013 | MEX3A |
| 0.64329 | 0.00013 | DUSP12 |
| -0.608895 | 0.00013 | SPANXB1 /// SPANXB2 /// SPANXF1 |
| -0.6010777 | 0.00013 | FUT8 |
| 0.600619 | 0.00013 | IMPA1 |
| 0.6205415 | 0.00013 | SAR1A |
| 0.625974 | 0.00013 | CPEB1 |
| -0.614676 | 0.00013 | ZAK |
| -0.9699055 | 0.00013 | HIST1H2BD |
| 0.6868855 | 0.00013 | BLZF1 |
| -0.708374 | 0.00013 | SYTL5 |
| -0.6549775 | 0.00013 | C11orf54 |
| -0.654085 | 0.00013 | NXN |
| -0.743369 | 0.00013 | FYN |
| -0.6103693 | 0.00013 | LOC100506965 |
| 0.6966165 | 0.00013 | RRAGC |
| -0.6304525 | 0.00013 | PTRHD1 |
| -0.610919 | 0.00013 | SALL2 |
| -0.6089618 | 0.00013 | COL4A2 |
| 1.22396 | 0.00013 | IL32 |
| 0.5948605 | 0.00014 | HSPB11 |
| 0.5936995 | 0.00014 | NVL |
| 0.8863115 | 0.00014 | SLC35F2 |
| -0.6346745 | 0.00014 | CPQ |
| 0.6187 | 0.00014 | DDX5 /// MIR3064 /// MIR5047 |
| 0.7090505 | 0.00014 | C12orf29 |
| -0.7351533 | 0.00014 | PLXNB2 |
| 0.807047 | 0.00014 | SLC31A2 |
| 0.715401 | 0.00014 | PSMC3 |
| 0.7004073 | 0.00014 | FAM122C |
| -0.5978125 | 0.00014 | C15orf52 |
| 0.6769795 | 0.00014 | TMEM200A |
| 0.6666428 | 0.00014 | KLF11 |
| -0.8875765 | 0.00014 | OTTHUMG00000179824 /// RP11-173M1.8 |
| 0.7026485 | 0.00014 | TOE1 |
| 0.6853595 | 0.00014 | ARL13B |
| -0.6152545 | 0.00014 | C6orf120 |
| -0.7211525 | 0.00014 | CDKN1C |
| -1.0079475 | 0.00014 | SAPCD2 |
| -0.5873505 | 0.00014 | IL10RB |
| -0.6498695 | 0.00014 | CDK19 |
| 0.6776445 | 0.00014 | BOD1L1 |
| -0.6104965 | 0.00014 | FAM196A |
| 0.6489645 | 0.00014 | CHUK |
| -0.587542 | 0.00014 | PTPRG |
| -0.8341182 | 0.00014 | GSTA4 |
| -0.5990725 | 0.00014 | LOC101060440 /// LOC101060471 /// LOC101060522 /// LOC440434 /// NPEPPS /// TBC1D3 |
| 0.59481 | 0.00014 | RFC4 |
| 0.6205915 | 0.00014 | FANCM |
| 0.6395565 | 0.00015 | E2F6 |
| -0.6441875 | 0.00015 | IGFBP2 |
| -0.6236895 | 0.00015 | EHD1 |
| -0.7217475 | 0.00015 | FAM224A /// FAM224B |
| -0.604557 | 0.00015 | FLNA |
| 0.6376897 | 0.00015 | FBF1 |
| -0.8072725 | 0.00015 | KCND2 |
| -0.605492 | 0.00015 | FLNC |
| 0.6351625 | 0.00015 | KIAA1279 |
| -0.6038015 | 0.00015 | B9D2 |
| 0.8965605 | 0.00015 | JUNB |
| 0.6218295 | 0.00015 | ZNF57 |
| 0.6542465 | 0.00015 | RAD1 |
| 0.632766 | 0.00015 | SNAP29 |
| 0.744197 | 0.00015 | PDCD2L |
| 0.889795 | 0.00015 | SLC25A33 |
| 0.619034 | 0.00015 | OGFOD1 |
| 0.5957848 | 0.00015 | FAM76A |
| 0.7635152 | 0.00015 | CAMTA1 |
| -0.622314 | 0.00015 | KRT19 |
| 0.627358 | 0.00015 | HES4 |
| -0.880897 | 0.00015 | PDK1 |
| 0.6293107 | 0.00015 | PIDD1 |
| 0.7471135 | 0.00015 | NEXN |
| 0.8496895 | 0.00015 | DUSP2 |
| -0.9725255 | 0.00015 | FPGT |
| -0.671204 | 0.00015 | EPM2AIP1 |
| 0.6268855 | 0.00016 | PMS1 |
| 0.689314 | 0.00016 | FAM169A |
| 0.6790793 | 0.00016 | TRAF4 |
| 0.938551 | 0.00016 | C18orf54 |
| 0.5965665 | 0.00016 | MRPL33 |
| -0.622672 | 0.00016 | GUSBP3 /// GUSBP9 /// LOC100653061 /// LOC101060519 |
| -0.6486068 | 0.00016 | LIX1L /// LOC101060547 |
| -0.7751333 | 0.00016 | BTN3A1 |
| -0.6214372 | 0.00016 | LOC389834 |
| 0.655751 | 0.00016 | EMG1 |
| 0.6011362 | 0.00016 | DNAJC15 |
| 0.607766 | 0.00016 | CDCA4 |
| 0.5907342 | 0.00016 | RNF19B |
| -0.592314 | 0.00016 | PPP3CA |
| -0.608723 | 0.00016 | PPP2R4 |
| -0.591791 | 0.00016 | LINC00493 |
| -0.627282 | 0.00016 | HK1 |
| -0.9573135 | 0.00016 | CCDC80 |
| -0.6035555 | 0.00016 | CNOT8 |
| 0.6413505 | 0.00016 | SPIN4 |
| 0.710551 | 0.00016 | TTC4 |
| -0.601335 | 0.00016 | STIM2 |
| -0.6246208 | 0.00016 | RHOU |
| 0.792636 | 0.00016 | CHRNA5 |
| 0.717773 | 0.00016 | TPR |
| 0.634552 | 0.00016 | DUSP10 |
| 0.5916445 | 0.00016 | OGFRL1 |
| -0.740554 | 0.00016 | DEPDC1 |
| 1.431178 | 0.00016 | NEURL3 |
| 0.5921395 | 0.00016 | HOXB9 |
| 0.683165 | 0.00016 | BYSL |
| 0.5981375 | 0.00017 | CPSF3 |
| -0.697537 | 0.00017 | SNUPN |
| 0.7055795 | 0.00017 | PLAGL1 |
| -0.649973 | 0.00017 | HNRNPU-AS1 |
| 0.6606847 | 0.00017 | SLC4A7 |
| -1.0138175 | 0.00017 | PYGM |
| -0.89077 | 0.00017 | RAB17 |
| -0.639083 | 0.00017 | LSM14A |
| 0.7080068 | 0.00017 | PAQR3 |
| -0.9598215 | 0.00017 | OTTHUMG00000175832 /// RP11-274H2.5 |
| 0.8018523 | 0.00017 | EXO5 |
| -0.733005 | 0.00017 | CTC-429P9.3 /// OTTHUMG00000182633 |
| 0.6738235 | 0.00017 | RBBP5 |
| -0.9247477 | 0.00017 | EFNA5 |
| 1.6052925 | 0.00017 | GEM |
| 0.602026 | 0.00017 | XBP1 |
| -0.80622 | 0.00017 | IDH1 |
| 0.589415 | 0.00017 | CCDC101 |
| -0.636248 | 0.00017 | SPNS2 |
| -0.9397015 | 0.00017 | PVALB |
| 0.8302245 | 0.00017 | OTTHUMG00000015043 /// RP11-554D15.1 |
| -0.87148 | 0.00017 | RPL31 /// TBC1D8 |
| -0.69498 | 0.00017 | NDRG3 |
| -0.625063 | 0.00017 | TDRD3 |
| -0.745574 | 0.00017 | PLXDC2 |
| -0.7975695 | 0.00017 | SERTM1 |
| 0.625618 | 0.00018 | LSM6 |
| -0.6908245 | 0.00018 | ZNF25 |
| 1.3682105 | 0.00018 | DDX60L |
| 0.8812485 | 0.00018 | LRCH2 |
| 0.6728555 | 0.00018 | PUS3 |
| 0.599661 | 0.00018 | NUPL2 |
| -0.6028555 | 0.00018 | ARHGAP17 |
| -0.6071665 | 0.00018 | CHMP2A |
| -0.8582345 | 0.00018 | LINC00086 /// LINC00087 |
| -0.611431 | 0.00018 | FAM8A1 |
| 0.621605 | 0.00018 | C5orf22 |
| 0.6471625 | 0.00018 | DPH3 |
| 0.6781485 | 0.00018 | DNA2 |
| 0.688173 | 0.00018 | TMEM106C |
| -0.658711 | 0.00018 | FBXL19 |
| -0.6925415 | 0.00018 | PIK3C3 |
| 0.654305 | 0.00018 | ANKRD20A11P |
| 0.7324485 | 0.00018 | CCDC82 |
| 0.6501065 | 0.00018 | AC099850.1 /// OTTHUMG00000132079 |
| -0.5851223 | 0.00018 | GRIP1 |
| 0.6026205 | 0.00018 | SAC3D1 |
| -0.6761885 | 0.00018 | TNIK |
| -0.6358063 | 0.00018 | USP3 |
| -0.8130435 | 0.00018 | BMP4 |
| 0.714777 | 0.00018 | ASRGL1 |
| -0.6359165 | 0.00018 | THAP2 |
| 0.611676 | 0.00019 | RAB21 |
| 0.984113 | 0.00019 | C8orf48 |
| 0.5959625 | 0.00019 | LOC100506639 /// ZNF131 |
| -0.7041885 | 0.00019 | FRMD4A |
| -0.9747855 | 0.00019 | PDGFRB |
| 0.8568755 | 0.00019 | SUSD5 |
| 0.588175 | 0.00019 | PRR24 |
| -0.6812525 | 0.00019 | WNT2B |
| 0.5887075 | 0.00019 | SCO1 |
| -0.6658825 | 0.00019 | ARL6IP5 |
| -0.7201505 | 0.00019 | NDRG4 |
| 0.5961645 | 0.00019 | NAPG |
| 0.850868 | 0.00019 | NCEH1 |
| -0.7558328 | 0.00019 | SGOL2 |
| 0.6945635 | 0.00019 | AP1S2 |
| -0.630684 | 0.00019 | CYFIP2 |
| -0.803177 | 0.00019 | LOC100128822 |
| 0.6648697 | 0.00019 | ZNF711 |
| -0.6354237 | 0.00019 | PAPOLA |
| 0.766903 | 0.00019 | SCLY |
| -0.5935548 | 0.00019 | SOX12 |
| 0.597518 | 0.00019 | SOGA2 |
| 0.6083045 | 0.00019 | ZNF85 |
| -0.7212015 | 0.00019 | TLE2 |
| -0.614706 | 0.00019 | EPB41L4A |
| 0.635849 | 0.00019 | DDB2 |
| 0.5879345 | 0.00019 | CELSR3 |
| 0.64675 | 0.0002 | NR2C2AP |
| -0.638702 | 0.0002 | BOK |
| 0.6442593 | 0.0002 | CCPG1 /// DYX1C1-CCPG1 |
| -0.6051535 | 0.0002 | ARHGEF17 |
| -0.759158 | 0.0002 | PRR5 |
| 0.612534 | 0.0002 | CRYBB2 /// CRYBB2P1 |
| 0.6667305 | 0.0002 | ZNF675 |
| 0.6127145 | 0.0002 | MTO1 |
| -0.7117568 | 0.0002 | KCNT2 |
| 0.6052825 | 0.0002 | PCGF5 |
| 0.7393345 | 0.0002 | CITED2 |
| -0.7639548 | 0.0002 | TGFBR3 |
| 0.5892665 | 0.0002 | MAPK6 |
| 0.979456 | 0.0002 | ZNF571 |
| 0.6014385 | 0.0002 | SLC25A16 |
| -0.7030165 | 0.0002 | DNM2 |
| 0.7135155 | 0.0002 | CLUHP3 |
| 0.632573 | 0.0002 | MIS12 |
| 0.6510983 | 0.0002 | CNST |
| -0.5874785 | 0.00021 | FAM127A |
| -0.603982 | 0.00021 | OTTHUMG00000172405 /// RP11-752G15.7 |
| 0.638327 | 0.00021 | NFE2L2 |
| -0.6751565 | 0.00021 | GAPDHP73 /// GAPDHP73 |
| -0.658473 | 0.00021 | H2BFS |
| -1.0602105 | 0.00021 | C12orf76 |
| -0.6252088 | 0.00021 | SLC25A23 |
| 0.6830725 | 0.00021 | PROSER1 |
| -0.9828935 | 0.00021 | MXI1 |
| -0.6424715 | 0.00021 | FBXO46 |
| -0.619515 | 0.00021 | EXD2 |
| -0.5859595 | 0.00021 | NICN1 |
| 0.5882403 | 0.00021 | HCCS |
| 0.755463 | 0.00021 | FAM86A |
| 0.6225852 | 0.00021 | OAS3 |
| 0.608951 | 0.00021 | FRG1 /// LOC100289097 /// LOC100996779 |
| 0.5911585 | 0.00021 | SNHG11 /// SNORA39 /// SNORA60 |
| -0.6133875 | 0.00021 | ANKRD23 |
| -0.8505715 | 0.00021 | SBSPON |
| 0.8330133 | 0.00022 | CTSC |
| 0.627328 | 0.00022 | CHTF18 |
| -0.6095222 | 0.00022 | CXADR |
| 0.608227 | 0.00022 | GK3P |
| 0.6094513 | 0.00022 | PPM1E |
| 0.644148 | 0.00022 | DDX11 |
| 1.73915 | 0.00022 | MIR155 /// MIR155HG |
| -0.6535945 | 0.00022 | ASB9 |
| 0.726631 | 0.00022 | C9orf142 |
| -0.5961905 | 0.00022 | BNC1 |
| 0.7480393 | 0.00022 | SNX16 |
| -0.6898178 | 0.00022 | PDGFD |
| 0.596159 | 0.00022 | ADPRHL2 |
| -0.8294833 | 0.00022 | CDCA3 |
| -0.590802 | 0.00023 | PPP1R14B |
| -0.594958 | 0.00023 | SEMA4B |
| 0.6153842 | 0.00023 | NUP62CL |
| 0.5972925 | 0.00023 | AC017002.2 /// OTTHUMG00000153694 |
| -0.6904133 | 0.00023 | NR2F2 |
| -0.8913825 | 0.00023 | LOC100507165 |
| 0.615417 | 0.00023 | FH |
| 0.6011345 | 0.00023 | HINT2 |
| -0.6407135 | 0.00023 | ATRNL1 |
| 0.702391 | 0.00023 | ANKRD30B |
| -0.702374 | 0.00023 | PABPC5 |
| 0.6293025 | 0.00023 | LOC283658 /// PYGO1 |
| -0.614203 | 0.00023 | KLHL31 |
| 0.795256 | 0.00023 | ALKBH3 |
| -0.904574 | 0.00023 | CTA-29F11.1 /// OTTHUMG00000172744 |
| -0.626395 | 0.00023 | TMEM98 |
| -0.678613 | 0.00024 | LOC401397 |
| -0.633951 | 0.00024 | PDHB |
| 0.6012355 | 0.00024 | PSMG3 |
| -0.7190095 | 0.00024 | CRIM1 |
| 0.7487257 | 0.00024 | SLFN5 |
| 0.618425 | 0.00024 | LZTS3 |
| 0.6246675 | 0.00024 | FAM161B |
| -0.5939245 | 0.00024 | CDR2L |
| -0.8303315 | 0.00024 | PTPRK |
| -0.6522495 | 0.00024 | PRSS16 |
| -0.7934118 | 0.00024 | KDM4C |
| -0.837445 | 0.00024 | SOX11 |
| -0.7085765 | 0.00024 | CCNF |
| -0.6065755 | 0.00024 | OTTHUMG00000177465 /// RP11-353N14.2 |
| -1.1990775 | 0.00024 | GJA1 |
| 0.720203 | 0.00025 | NUP43 |
| -0.679429 | 0.00025 | AC092620.2 /// OTTHUMG00000153633 |
| 0.6122928 | 0.00025 | CDADC1 |
| -0.856115 | 0.00025 | TMEM132B |
| -0.688972 | 0.00025 | POTEKP |
| 0.6212605 | 0.00025 | FAM178A |
| 0.5965217 | 0.00025 | LIN52 |
| 0.684169 | 0.00025 | QPCT |
| 0.9456995 | 0.00025 | KIF24 |
| 0.6176457 | 0.00025 | MIR1304 /// SNORA1 /// SNORA18 /// SNORA32 /// SNORA40 /// SNORA8 /// SNORD5 /// TAF1D |
| 0.6845395 | 0.00026 | GBP3 |
| -0.631339 | 0.00026 | CFH |
| -0.921173 | 0.00026 | LOC100506934 |
| 0.6185535 | 0.00026 | DNAJB9 |
| 1.245121 | 0.00026 | RSAD2 |
| 0.6040715 | 0.00026 | ZNF597 |
| 0.7141895 | 0.00026 | CCPG1 |
| -0.827473 | 0.00026 | OTTHUMG00000162817 /// RP11-549J18.1 |
| -0.6206905 | 0.00026 | PRKCB |
| -0.58997 | 0.00026 | HOTAIRM1 |
| -0.5980558 | 0.00026 | C11orf70 |
| 0.7097205 | 0.00026 | AP3B2 |
| 0.6474165 | 0.00027 | NFKBIE |
| -0.592799 | 0.00027 | TNFRSF1A |
| 0.598351 | 0.00027 | MKI67IP |
| -0.6523043 | 0.00027 | RAD51-AS1 |
| -0.795623 | 0.00027 | CCDC50 |
| -0.589518 | 0.00027 | CADM1 |
| 0.6682975 | 0.00027 | TARS |
| -0.7162305 | 0.00027 | AVIL |
| -0.7084025 | 0.00027 | CBLN2 |
| 0.624751 | 0.00027 | MAFG |
| -0.585855 | 0.00027 | FSCN1 |
| 0.6195335 | 0.00027 | ESF1 |
| 0.9289635 | 0.00028 | EPHA4 |
| 1.165274 | 0.00028 | DUSP6 |
| -0.5901698 | 0.00028 | ERMP1 |
| -0.6359035 | 0.00028 | PIK3C2B |
| 0.7647205 | 0.00028 | ZSCAN12 |
| -0.6126383 | 0.00028 | ITSN1 |
| 0.6218195 | 0.00028 | SLC7A3 |
| 0.5959065 | 0.00028 | NTMT1 |
| 1.2347615 | 0.00028 | ZNF530 |
| 0.8279755 | 0.00028 | ASF1B |
| -0.68515 | 0.00029 | SLC9A3R1 |
| -0.6263053 | 0.00029 | ANKRD37 |
| 0.7291288 | 0.00029 | MYBL1 |
| 1.0898438 | 0.00029 | KYNU |
| -0.680422 | 0.00029 | LOC150381 |
| -0.6679205 | 0.00029 | NBEA |
| 0.5881027 | 0.00029 | DCLRE1B |
| -0.5887987 | 0.00029 | PCBP2 |
| -0.7083 | 0.00029 | PRO2964 |
| 0.594913 | 0.00029 | MARCH1 |
| -0.6085495 | 0.0003 | YPEL1 |
| 0.637554 | 0.0003 | SNRPA1 |
| 0.616056 | 0.0003 | MTX3 |
| -0.5993455 | 0.0003 | LINC00260 |
| -0.6028425 | 0.0003 | TMEM2 |
| 0.632827 | 0.0003 | SRBD1 |
| 0.7714397 | 0.0003 | THAP9 |
| 0.6311695 | 0.0003 | KCNMB3 |
| -0.595838 | 0.0003 | NTNG2 |
| -0.629444 | 0.0003 | OTTHUMG00000021298 /// RP11-268G12.1 |
| 0.6301105 | 0.0003 | ZNF593 |
| -0.611108 | 0.0003 | MGMT |
| 0.6086485 | 0.0003 | ZNF850 |
| 0.6498785 | 0.0003 | NPY |
| 1.0148345 | 0.00031 | ETV5 |
| -0.6714595 | 0.00031 | APLN |
| -0.815173 | 0.00031 | OTTHUMG00000176931 /// RP11-319G9.3 |
| -1.0397835 | 0.00031 | MLLT4 |
| -0.6200708 | 0.00031 | ABCC1 |
| -0.6121483 | 0.00031 | LRBA |
| -0.741605 | 0.00031 | LDHA |
| -0.7117285 | 0.00031 | FAM189A1 |
| -0.6906005 | 0.00031 | C7orf55 |
| 0.6546635 | 0.00031 | ABHD5 |
| 0.5907955 | 0.00032 | PIGA |
| 0.7296525 | 0.00032 | CDR2 |
| -0.687469 | 0.00032 | CCNY |
| 0.838672 | 0.00032 | HS3ST3A1 |
| -0.6223035 | 0.00033 | TCF12 |
| 0.7027085 | 0.00033 | CHM |
| 0.8057545 | 0.00033 | WDR53 |
| -0.5940365 | 0.00033 | EBLN2 |
| 0.7203795 | 0.00034 | POU4F2 |
| -1.071238 | 0.00034 | HIST2H2AA3 /// HIST2H2AA4 |
| 0.655507 | 0.00034 | CEP76 |
| -0.6688865 | 0.00034 | HIST1H2BC /// HIST1H2BE /// HIST1H2BF /// HIST1H2BG /// HIST1H2BI |
| 0.6015415 | 0.00034 | CDC5L |
| -0.7131885 | 0.00034 | ANKRD13B |
| -1.111175 | 0.00034 | TMEM255A |
| -0.638262 | 0.00034 | FLJ10038 |
| 0.7827772 | 0.00034 | GBP1 |
| -0.6318665 | 0.00034 | G2E3 |
| 0.6024132 | 0.00034 | JUN |
| 0.6259785 | 0.00034 | CAMK2D |
| 0.62629 | 0.00035 | METTL1 |
| -0.670275 | 0.00035 | ANKZF1 |
| 1.35113 | 0.00035 | HSD17B6 |
| 0.597949 | 0.00035 | SKIL |
| 0.6007695 | 0.00035 | TNFRSF10B |
| -0.6134605 | 0.00036 | PRKCDBP |
| -0.6772415 | 0.00037 | OTTHUMG00000180272 /// RP11-567M16.4 |
| -0.791676 | 0.00037 | AMN1 |
| -0.9210975 | 0.00037 | LOC646903 |
| 0.644814 | 0.00037 | SPAG1 |
| 0.585339 | 0.00037 | CDK5RAP1 |
| -0.746315 | 0.00037 | QPRT |
| -0.7294728 | 0.00037 | KLHL4 |
| 0.728994 | 0.00038 | SAE1 |
| 0.7745523 | 0.00038 | GZF1 |
| 0.5961245 | 0.00038 | TIGD2 |
| -0.6379725 | 0.00038 | KLK10 |
| 0.6031918 | 0.00038 | EED |
| -0.6053223 | 0.00038 | KIAA1432 |
| -0.6222345 | 0.00038 | C2CD2 |
| -0.639987 | 0.00038 | G6PD |
| -0.6503575 | 0.00039 | MPPED2 |
| 0.6174888 | 0.00039 | LOC100506651 |
| 0.6041215 | 0.00039 | AZI2 |
| -0.7086107 | 0.00039 | CDKAL1 |
| 0.729123 | 0.00039 | TLE4 |
| -0.6166802 | 0.00039 | PPP3CB |
| 1.0568645 | 0.0004 | H1F0 |
| 0.654486 | 0.0004 | IER2 |
| 0.607216 | 0.0004 | ISL1 |
| 0.9819635 | 0.0004 | ZSCAN16 |
| 0.806551 | 0.0004 | TMEM217 |
| -0.9244885 | 0.0004 | MGAT3 |
| -0.774751 | 0.00041 | LYRM9 |
| 0.6308448 | 0.00041 | EFNB2 |
| 0.780336 | 0.00041 | KLF10 |
| -0.6890005 | 0.00041 | CCDC89 |
| -0.6939415 | 0.00041 | NEO1 |
| -0.816205 | 0.00043 | CRIP1 |
| -0.755802 | 0.00043 | NR2F2-AS1 |
| -0.874236 | 0.00043 | UPK1B |
| -0.754565 | 0.00043 | HIST1H2BC /// HIST1H2BE /// HIST1H2BF /// HIST1H2BG /// HIST1H2BI /// NCALD |
| 1.1559113 | 0.00043 | PLA2G4C |
| -0.741265 | 0.00044 | SNRPN /// SNURF |
| 0.741484 | 0.00044 | SOD2 |
| 0.6225625 | 0.00045 | EML6 |
| -0.6445298 | 0.00045 | TRIM34 /// TRIM6-TRIM34 |
| 0.6132312 | 0.00045 | MIR503HG |
| -0.6705855 | 0.00045 | DANCR |
| 0.6379525 | 0.00045 | MSH5 /// MSH5-SAPCD1 /// SAPCD1 |
| 0.821599 | 0.00045 | CCDC18 |
| 0.7148935 | 0.00046 | PLAUR |
| -0.6794927 | 0.00046 | C20orf112 |
| -0.6781965 | 0.00046 | PLGLA /// PLGLB1 /// PLGLB2 |
| -0.670641 | 0.00046 | ACO1 |
| 0.6026117 | 0.00046 | FAM126B |
| -0.6621145 | 0.00046 | C7orf55 /// C7orf55-LUC7L2 |
| 0.6177965 | 0.00046 | PPIL4 |
| 0.668059 | 0.00047 | ZNF662 |
| -0.7477603 | 0.00047 | TPBG |
| -0.9614715 | 0.00047 | VAPA |
| -0.946287 | 0.00048 | CPB1 |
| 0.7310065 | 0.00048 | PRDM13 |
| -0.9387185 | 0.00048 | PFKFB3 |
| 1.0561675 | 0.00049 | ZNF442 |
| -0.6700925 | 0.00049 | ANG |
| 0.6296448 | 0.00049 | SETDB2 |
| -0.7255745 | 0.0005 | EHHADH |
| 0.714588 | 0.0005 | MIR17 /// MIR17HG /// MIR18A /// MIR19A /// MIR19B1 /// MIR20A /// MIR92A1 |
| -0.6676625 | 0.0005 | PLOD2 |
| -0.756439 | 0.0005 | MYO10 |
| 0.6878425 | 0.0005 | TUBG1 |
| 0.641498 | 0.00051 | PLEKHF2 |
| 0.594009 | 0.00051 | PIK3R3 |
| -0.609425 | 0.00051 | BTD |
| -0.626638 | 0.00051 | INADL |
| -0.6073765 | 0.00052 | EFNA1 |
| 0.895596 | 0.00052 | SLC1A5 |
| -0.8802035 | 0.00053 | KCNMA1 |
| 0.6695655 | 0.00053 | NAA16 |
| -0.5977978 | 0.00053 | HIST1H2AD /// HIST1H3A /// HIST1H3B /// HIST1H3C /// HIST1H3D /// HIST1H3E /// HIST1H3F /// HIST1H3G /// HIST1H3H /// HIST1H3I /// HIST1H3J |
| -0.606863 | 0.00053 | PCOLCE |
| -0.786498 | 0.00053 | TNNI3K |
| 0.682178 | 0.00054 | CEP290 |
| -0.6883695 | 0.00054 | PPFIBP2 |
| 0.714901 | 0.00054 | ZNF649 |
| 0.971141 | 0.00054 | ZBTB42 |
| 0.6089585 | 0.00054 | NUDT21 |
| 0.611347 | 0.00054 | GSG2 |
| -0.641812 | 0.00055 | ZNF703 |
| 0.9020125 | 0.00055 | FOSL1 |
| -0.622659 | 0.00055 | DIAPH2 |
| 1.2850405 | 0.00056 | EGR1 |
| -0.760454 | 0.00057 | CA8 |
| 0.600493 | 0.00057 | SYNGR3 |
| 0.748596 | 0.00058 | JAG1 |
| 0.887645 | 0.00059 | ADRB1 |
| -0.8315995 | 0.00059 | FRY |
| -0.9442605 | 0.0006 | TNS3 |
| 0.6194165 | 0.0006 | RIOK1 |
| -0.5888278 | 0.0006 | FANCF |
| 0.9805995 | 0.0006 | CCL20 |
| 1.4907515 | 0.0006 | PCSK1 |
| -0.678444 | 0.0006 | GPC6 |
| -0.595952 | 0.0006 | DPP10 |
| -0.9379415 | 0.00061 | ITGB4 |
| 0.5964868 | 0.00061 | ZC3H12C |
| -0.817798 | 0.00061 | INSIG2 |
| 0.5928305 | 0.00061 | CCDC134 |
| 0.645508 | 0.00061 | OTTHUMG00000183089 /// RP11-399K21.12 |
| 0.609602 | 0.00062 | SLC16A7 |
| 0.6829525 | 0.00062 | SLMO1 |
| -0.651753 | 0.00062 | C14orf132 |
| 0.598187 | 0.00062 | ZNFX1 |
| 0.8615245 | 0.00062 | FAM161A |
| 0.739693 | 0.00063 | RPL39L |
| -0.6550232 | 0.00064 | SSBP2 |
| 0.8264485 | 0.00064 | CMPK2 |
| 0.594866 | 0.00065 | MRPL39 |
| 0.609929 | 0.00065 | STAG3L4 |
| -1.0095425 | 0.00066 | OTTHUMG00000176930 /// RP11-319G9.2 |
| -0.743953 | 0.00067 | PRR15 |
| 0.7391185 | 0.00067 | ISG20L2 |
| 0.6002875 | 0.00068 | PCSK5 |
| 0.803432 | 0.00069 | ARIH2OS |
| -0.6327695 | 0.00069 | SBF2 |
| 0.927028 | 0.00069 | IFIH1 |
| -0.6950175 | 0.0007 | GPR160 |
| 0.6145883 | 0.00071 | TAF3 |
| 0.611225 | 0.00073 | LY96 |
| 0.6318402 | 0.00074 | C10orf88 |
| 0.711586 | 0.00074 | CYB5R2 |
| 0.672683 | 0.00074 | TOP3A |
| 0.759653 | 0.00075 | BATF3 |
| -0.8547272 | 0.00076 | LOC646762 |
| -0.677525 | 0.00076 | ADM |
| 0.6229055 | 0.00076 | SLC43A2 |
| -0.5982 | 0.00078 | NQO1 |
| 0.766145 | 0.00078 | ADPRM |
| 0.9349597 | 0.00081 | MARVELD2 |
| 0.587192 | 0.00081 | NR4A2 |
| -0.66581 | 0.00082 | LOC100510707 /// LOC101060287 /// LOC101060303 /// LOC101060321 /// LOC101060351 /// LOC101060367 /// LOC101060376 /// LOC101060389 /// LOC101060403 /// LOC101060421 /// LOC101060440 /// LOC101060471 /// LOC101060489 /// LOC101060506 /// LOC101060522 /// TBC1D3 /// TBC1D3C /// TBC1D3F /// TBC1D3G /// TBC1D3H |
| -0.6943315 | 0.00083 | SORCS2 |
| -0.6717415 | 0.00083 | LINC00115 |
| 0.624464 | 0.00084 | OTTHUMG00000175497 /// RP6-24A23.7 |
| -0.5994435 | 0.00085 | OTTHUMG00000176421 /// RP11-1006G14.4 |
| 0.72207 | 0.00085 | CYLD |
| -0.5941635 | 0.00085 | FBXO9 |
| -0.8931805 | 0.00085 | OTTHUMG00000176923 /// RP11-264L1.1 |
| 1.066101 | 0.00086 | FAM105A |
| -0.5850435 | 0.00087 | CDC25B |
| 0.767085 | 0.00087 | KIF27 |
| 0.5959905 | 0.00088 | HSPH1 |
| 0.6292285 | 0.00088 | PSPH |
| 0.7293895 | 0.00088 | LINC00669 |
| 0.920412 | 0.00089 | TM6SF1 |
| -0.60176 | 0.00089 | MDFI |
| -0.7095395 | 0.00089 | OTTHUMG00000176937 /// RP11-679B19.1 |
| -0.826638 | 0.0009 | PKI55 |
| 0.638924 | 0.00092 | AREG /// AREGB |
| -0.771474 | 0.00093 | SLITRK1 |
| 0.6521357 | 0.00094 | GAS2L3 |
| 0.6251085 | 0.00094 | C10orf2 |
| 0.785935 | 0.00095 | LOC101060503 /// TXNIP |
| -0.6071505 | 0.00096 | DCTN3 |
| -0.7893235 | 0.00098 | IPW /// LOC100506948 /// SNORD107 /// SNORD115-13 /// SNORD115-26 /// SNORD115-7 /// SNORD116-28 |
| 0.6375898 | 0.00099 | CD274 |
| 1.2797038 | 0.001 | TFPI2 |
| 1.1812385 | 0.00101 | CCL3 /// CCL3L1 /// CCL3L3 /// LOC101060267 |
| -0.6621295 | 0.00102 | TRIM16L |
| 0.637552 | 0.00102 | OTTHUMG00000173051 /// RP5-991G20.4 |
| 0.6405683 | 0.00103 | OAS1 |
| 0.5896015 | 0.00107 | TES |
| -0.7057375 | 0.00107 | DPP6 |
| 1.278577 | 0.00108 | CNN1 |
| -0.639043 | 0.00108 | OTTHUMG00000161674 /// OTTHUMG00000161676 /// RP11-164P12.4 /// RP11-164P12.5 |
| -0.6614577 | 0.0011 | CAV1 |
| 0.628643 | 0.0011 | NHS |
| -0.6021025 | 0.00114 | FAM102B |
| -0.984228 | 0.00115 | PCDHB16 |
| -0.7787355 | 0.00118 | C1orf191 |
| 0.6477085 | 0.0012 | FOSL2 |
| -0.6622395 | 0.00122 | MYO5B |
| -0.6219648 | 0.00123 | SCFD2 |
| -0.5987725 | 0.00123 | FSTL5 |
| 0.6675805 | 0.00127 | CA2 |
| 1.0183345 | 0.0013 | LOC645638 |
| -0.8204095 | 0.0013 | LOC338620 |
| -0.8267743 | 0.00131 | NOL3 |
| -0.8315485 | 0.00131 | TMEM120A |
| -0.59069 | 0.00135 | FBXW4P1 |
| 0.6541815 | 0.00135 | AOC2 |
| 0.598899 | 0.00135 | TONSL |
| -1.1141135 | 0.00136 | LINC00842 |
| 0.5991625 | 0.00136 | RP9 /// RP9P |
| -0.5898378 | 0.00136 | LOC100996628 /// SHROOM3 |
| 0.7246025 | 0.00138 | IPO11 /// LRRC70 |
| -0.683454 | 0.0014 | ADAM1A /// ADAM1A |
| -0.5928475 | 0.00141 | MBD5 |
| -0.5943295 | 0.00141 | AES |
| 0.714078 | 0.00143 | PAPPA |
| -0.863318 | 0.00143 | KBTBD3 |
| 0.593032 | 0.00144 | ATL3 |
| 0.9025233 | 0.00145 | EPSTI1 |
| -0.6367695 | 0.00148 | AGFG1 |
| -0.6291863 | 0.00148 | SNX33 |
| -0.8610505 | 0.00152 | OTTHUMG00000176181 /// RP11-119F7.5 |
| 0.6924895 | 0.00155 | LIMK2 |
| -0.6321645 | 0.00155 | OTTHUMG00000172119 /// RP11-208K4.2 |
| 0.6450325 | 0.00159 | IL15RA |
| -0.658821 | 0.00159 | FAM83A |
| 0.617443 | 0.00159 | AHI1 |
| -0.602638 | 0.0016 | XPR1 |
| -0.7113728 | 0.0016 | AK4 /// LOC100507855 |
| -0.612647 | 0.00161 | GNAZ |
| -0.587558 | 0.00163 | RIMKLB |
| -0.6314745 | 0.00165 | PAN3-AS1 |
| -1.4104265 | 0.00166 | NRN1 |
| -0.7358565 | 0.00167 | SFXN3 |
| 1.007549 | 0.00168 | RSPO3 |
| 0.6699675 | 0.00171 | OTTHUMG00000168442 /// RP11-705C15.3 |
| 0.5934732 | 0.00171 | ELOVL2 |
| -0.6633432 | 0.00173 | PFKFB4 |
| 0.7378895 | 0.00176 | SH2D3C |
| 0.7269605 | 0.00177 | ZNF79 |
| 0.6703372 | 0.00177 | IL11 |
| 0.664808 | 0.00178 | CCNO |
| -0.6836185 | 0.00179 | GUCY1B3 |
| -0.7267178 | 0.00183 | KDM5B |
| -0.642388 | 0.00184 | LBH |
| 1.2435535 | 0.00185 | IL24 |
| 0.5861723 | 0.00187 | SOX7 |
| 0.670349 | 0.00187 | ZFAND2A |
| -0.594993 | 0.00191 | NAGS |
| -1.0979225 | 0.00193 | NDRG1 |
| 0.754308 | 0.00193 | SLAMF7 |
| -0.6538895 | 0.00194 | BNIP3L |
| -0.6085435 | 0.00194 | TM9SF3 |
| 0.6431305 | 0.00197 | TYW5 |
| -0.5937765 | 0.00198 | FAM226A /// FAM226B |
| 0.7271165 | 0.002 | NPAT |
| -0.598814 | 0.00207 | AFAP1 |
| 0.771144 | 0.00208 | PVRL4 |
| -0.831958 | 0.00209 | DOK6 |
| 0.681113 | 0.00209 | EBI3 |
| -0.8014205 | 0.00212 | GHR |
| -0.664874 | 0.00212 | OTTHUMG00000175836 /// RP11-145F16.2 |
| 0.6410415 | 0.00212 | HMOX1 |
| 0.6229195 | 0.00218 | ERCC6L2 |
| -0.6635585 | 0.00221 | SPATA17 |
| 0.59341 | 0.00224 | CNNM4 |
| 0.6977228 | 0.00227 | C12orf39 |
| -0.733952 | 0.00233 | LOC202025 |
| 0.627417 | 0.00245 | HLF |
| 0.8103902 | 0.00247 | TNFSF13B |
| 0.672837 | 0.00248 | SYBU |
| -0.6086315 | 0.0025 | TMEM102 |
| 0.6512673 | 0.00253 | SHISA9 |
| 0.632338 | 0.00254 | PSG4 |
| -0.6191685 | 0.00256 | HIST1H2AM |
| 0.6530465 | 0.00261 | FZD8 |
| 0.646577 | 0.00261 | TRAPPC13 |
| 0.8683382 | 0.00265 | BTC |
| -0.6075848 | 0.00271 | SLC2A14 /// SLC2A3 |
| 0.6262475 | 0.00281 | S100A2 |
| -0.681695 | 0.00293 | PCDHB14 |
| -0.884716 | 0.00297 | SLC2A1 |
| 0.5878085 | 0.00297 | IRF7 |
| -0.61335 | 0.00299 | CAB39L |
| -0.5981595 | 0.00301 | SMYD3 |
| -0.5934665 | 0.00303 | SPAG4 |
| 0.865682 | 0.00303 | TNFSF10 |
| -0.629954 | 0.00306 | ZNF174 |
| 0.6167475 | 0.00312 | APOL3 |
| -1.3861575 | 0.00313 | PPFIA4 |
| -0.62696 | 0.00323 | MYLK |
| 0.632302 | 0.00331 | FLJ00104 |
| 0.613663 | 0.00341 | SLC22A4 |
| -0.706096 | 0.00356 | HIST1H2AB /// HIST1H2AE |
| -0.592687 | 0.00373 | ENPP5 |
| 0.733681 | 0.00373 | EGR2 |
| -0.6660425 | 0.00381 | ECHDC3 |
| -0.586679 | 0.00386 | CAPNS2 |
| -0.9422305 | 0.00395 | ALDH8A1 |
| 0.6254075 | 0.00396 | SALL1 |
| -0.6623515 | 0.00422 | ARHGEF26-AS1 |
| 0.747023 | 0.00425 | YWHAH |
| 0.7583765 | 0.00431 | ARRDC4 |
| -0.680776 | 0.00431 | LOC286272 |
| 0.6229493 | 0.00445 | ASAH2B |
| 0.6815245 | 0.00449 | SAMD9L |
| 0.597091 | 0.0045 | C4orf21 |
| 0.5863525 | 0.00453 | CES2 |
| -0.603712 | 0.0047 | FUK |
| -0.59015 | 0.00478 | LOC100130987 |
| 0.6120328 | 0.00486 | ALG10 |
| 0.6772595 | 0.00487 | OR51B6 |
| 0.720169 | 0.00512 | CCL5 |
| 0.623175 | 0.00602 | PI3 |
| 0.6383325 | 0.00629 | LOC728537 |
| 0.7653315 | 0.00706 | HS3ST3B1 |
| 0.616169 | 0.00755 | LOC100506538 /// NDUFAF6 |
| 0.9904385 | 0.00772 | CCL4 |
| 0.6168255 | 0.008 | SOX9 |
| -0.688622 | 0.00951 | SERPING1 |
| 0.5996255 | 0.01029 | MYO6 |
| 0.7290355 | 0.01242 | OTTHUMG00000066821 /// RP11-87H9.3 |
| -0.70245 | 0.01266 | MAF |
| -0.598267 | 0.01292 | C11orf45 |
| -0.5882035 | 0.01331 | OTTHUMG00000175555 /// RP6-201G10.2 |
| 0.617516 | 0.01388 | MX2 |
| 0.7933655 | 0.01454 | CCDC96 |
| 0.633094 | 0.01467 | CHGB |
| 0.6075825 | 0.01473 | GMPR |
| -0.642893 | 0.0161 | SCG5 |
| -1.09808 | 0.01619 | STC1 |
| 0.5854675 | 0.0168 | ZNF256 |
| 0.6016685 | 0.01903 | GAS5 |
| 0.719492 | 0.02162 | C5 |
| 1.106323 | 0.02902 | C8orf4 |
| -0.6578525 | 0.03593 | LINC00622 |
| -0.7431345 | 0.03716 | GNE |
| -0.6368025 | 0.0429 | ANGPTL4 |
| -0.589234 | 0.04654 | TFRC |
