# Supplementary material for: p53 modeling as a route to mesothelioma patients stratification and novel therapeutic identification
Source: J Transl Med. 2018 Oct 13;16:282. doi: 10.1186/s12967-018-1650-0 (PMC6186085; doi:10.1186/s12967-018-1650-0)
Supplement: Supplementary file 2 — Additional file 2: Table S2. ETO vs ctrl upregulated genes. [file 12967_2018_1650_MOESM2_ESM.docx]

**Table S2:** Etop vs ctrl upregulated genes

| **logFC** | **P.Value** | **gene_symbol** |
| --- | --- | --- |
| 2.871933 | 8.57E-09 | SNHG12 /// SNORA16A /// SNORA44 /// SNORA61 |
| 2.623708 | 8.71E-09 | GADD45A |
| 2.185985 | 3.05E-08 | PMAIP1 |
| 2.03825 | 3.58E-08 | DTL |
| 2.069 | 3.76E-08 | CLGN |
| 2.91829 | 4.00E-08 | DKK1 |
| 2.241673 | 6.42E-08 | ATF3 |
| 2.057041 | 7.15E-08 | CLDN1 |
| 1.965796 | 7.78E-08 | CEBPG |
| 2.424589 | 1.19E-07 | OTTHUMG00000172919 /// RP11-340F14.5 |
| 1.888998 | 1.28E-07 | MTHFD2 |
| 1.720724 | 1.46E-07 | NUPR1 |
| 1.675335 | 1.69E-07 | ZNF367 |
| 1.995933 | 1.70E-07 | CDKN1A |
| 1.666968 | 1.80E-07 | PPP1R15A |
| 1.838743 | 2.09E-07 | C6orf48 |
| 1.945969 | 2.22E-07 | DDIT3 |
| 2.132577 | 2.23E-07 | UPP1 |
| 3.185019 | 2.32E-07 | INHBE |
| 1.683473 | 2.52E-07 | SESN2 |
| 1.540492 | 2.82E-07 | SNHG1 /// SNORD22 /// SNORD25 /// SNORD26 /// SNORD27 /// SNORD28 /// SNORD29 /// SNORD31 |
| 1.810351 | 3.71E-07 | FLRT2 /// LOC100506718 |
| 1.516467 | 3.88E-07 | PSMC3IP |
| 1.414168 | 4.03E-07 | MCM10 |
| 1.464553 | 4.47E-07 | HSPA14 |
| 1.574487 | 4.52E-07 | RRM2 |
| 1.948926 | 4.59E-07 | PSAT1 |
| 1.441689 | 4.94E-07 | FBLL1 |
| 1.71192 | 5.11E-07 | PTX3 |
| 1.559677 | 5.77E-07 | BRCA2 |
| 1.393121 | 5.87E-07 | TMEM194A |
| 1.55149 | 6.05E-07 | RND3 |
| 1.822213 | 6.07E-07 | IL6 |
| 1.485994 | 6.16E-07 | SUPV3L1 |
| 1.813401 | 6.22E-07 | CCNE2 |
| 1.808603 | 6.34E-07 | CDC6 |
| 1.348107 | 6.47E-07 | ATAD2 |
| 1.961164 | 6.53E-07 | CASP1 |
| 1.375474 | 6.88E-07 | SNAI2 |
| 1.29055 | 7.99E-07 | CCDC174 |
| 1.467821 | 8.15E-07 | APOBEC3B |
| 1.569791 | 8.21E-07 | SNHG15 /// SNORA9 |
| 1.29094 | 8.39E-07 | TIPIN |
| 1.445903 | 8.39E-07 | FGF2 |
| 1.770192 | 8.72E-07 | FAM129A |
| 1.303264 | 8.75E-07 | TMEM38B |
| 1.381258 | 9.00E-07 | TGFB2 |
| 1.382591 | 9.33E-07 | SRFBP1 |
| 1.481352 | 9.48E-07 | E2F8 |
| 2.203156 | 9.57E-07 | CSTA |
| 1.237968 | 1.02E-06 | WDHD1 |
| 1.267126 | 1.07E-06 | SNHG17 |
| 1.843474 | 1.07E-06 | RIBC2 |
| 1.605926 | 1.09E-06 | EXO1 |
| 1.507565 | 1.15E-06 | TNFRSF12A |
| 1.23509 | 1.16E-06 | SLC3A2 |
| 1.228804 | 1.20E-06 | RBBP8 |
| 1.615935 | 1.22E-06 | TIGAR |
| 1.218632 | 1.25E-06 | UBE2T |
| 2.077086 | 1.32E-06 | LAMP3 |
| 1.38781 | 1.32E-06 | TRIB3 |
| 1.223903 | 1.32E-06 | CHAC2 |
| 1.21772 | 1.35E-06 | CARS |
| 1.849494 | 1.37E-06 | CARD16 /// CASP1 |
| 1.356562 | 1.46E-06 | TEX30 |
| 1.644828 | 1.46E-06 | AEN |
| 1.212034 | 1.50E-06 | RNASEH2A |
| 1.223855 | 1.51E-06 | PRIM1 |
| 1.692425 | 1.52E-06 | HRK |
| 1.374736 | 1.53E-06 | CDCA5 |
| 1.496446 | 1.53E-06 | IL7R |
| 1.288234 | 1.59E-06 | TLCD1 |
| 1.658149 | 1.61E-06 | FAM111B |
| 1.162096 | 1.62E-06 | DNAJC9 |
| 1.284085 | 1.68E-06 | GARS |
| 1.185733 | 1.80E-06 | AARS |
| 1.277733 | 1.81E-06 | IFRD1 |
| 2.854697 | 1.82E-06 | ANKRD1 |
| 1.608096 | 1.89E-06 | SHISA2 |
| 1.250606 | 1.92E-06 | WARS |
| 1.239638 | 1.92E-06 | STRIP2 |
| 1.186505 | 1.99E-06 | C11orf82 |
| 1.247223 | 2.04E-06 | WDR76 |
| 1.591078 | 2.07E-06 | CXCL3 |
| 1.808128 | 2.07E-06 | IL8 |
| 1.177108 | 2.09E-06 | GNL2 |
| 1.17355 | 2.16E-06 | C5orf34 |
| 1.722983 | 2.21E-06 | IL13RA2 |
| 1.391165 | 2.24E-06 | SARS |
| 1.562957 | 2.25E-06 | CDC25A |
| 1.155019 | 2.28E-06 | CTPS1 |
| 1.167857 | 2.28E-06 | ZBTB21 |
| 1.142719 | 2.36E-06 | MASTL |
| 2.089621 | 2.46E-06 | ANKRD20A5P |
| 1.173153 | 2.58E-06 | DDX21 |
| 1.26141 | 2.60E-06 | RHEBL1 |
| 1.13387 | 2.61E-06 | C7orf57 |
| 1.075317 | 2.63E-06 | DONSON |
| 1.107078 | 2.71E-06 | GFPT1 |
| 1.161782 | 2.72E-06 | LOC100289092 |
| 1.166609 | 2.72E-06 | UPF3B |
| 1.074151 | 2.80E-06 | LOC101060460 /// POLR3C |
| 1.328308 | 2.86E-06 | SHMT2 |
| 1.294576 | 2.95E-06 | CDKN2AIP |
| 1.130267 | 3.00E-06 | CHAC1 |
| 1.278457 | 3.02E-06 | CDT1 |
| 1.270093 | 3.08E-06 | SKA1 |
| 1.081032 | 3.11E-06 | GTPBP4 |
| 1.098338 | 3.14E-06 | HBEGF |
| 2.241104 | 3.21E-06 | INHBA |
| 1.115502 | 3.28E-06 | CTB-92J24.2 /// OTTHUMG00000183390 |
| 1.074096 | 3.29E-06 | KLHDC7B |
| 1.422446 | 3.33E-06 | OTTHUMG00000178878 /// RP11-214C8.5 |
| 1.084506 | 3.34E-06 | IFI30 /// PIK3R2 |
| 1.34166 | 3.35E-06 | GCH1 |
| 1.175833 | 3.38E-06 | ESCO2 |
| 1.184757 | 3.40E-06 | SLC7A11 |
| 1.892538 | 3.49E-06 | PHLDB2 |
| 1.175331 | 3.54E-06 | CENPQ |
| 1.106202 | 3.56E-06 | PHYH |
| 1.581823 | 3.57E-06 | HSPBAP1 |
| 1.127739 | 3.64E-06 | PLAU |
| 1.190421 | 3.65E-06 | CENPM |
| 1.271799 | 3.68E-06 | PTPRG-AS1 |
| 1.100877 | 3.84E-06 | THAP9-AS1 |
| 1.056355 | 3.89E-06 | PNO1 |
| 1.055013 | 3.89E-06 | POLE2 |
| 1.051755 | 3.90E-06 | MCM7 |
| 1.178143 | 3.93E-06 | MND1 |
| 1.266048 | 4.04E-06 | MOCOS |
| 1.027055 | 4.05E-06 | RWDD2B |
| 1.015878 | 4.09E-06 | MCM2 |
| 1.02324 | 4.10E-06 | SLC27A2 |
| 1.014243 | 4.15E-06 | BLM |
| 1.078636 | 4.16E-06 | HAUS8 |
| 1.97159 | 4.21E-06 | HKDC1 |
| 1.066001 | 4.23E-06 | MCAM |
| 1.13264 | 4.31E-06 | PFDN2 |
| 1.100891 | 4.38E-06 | LOC285084 |
| 1.048546 | 4.40E-06 | TUFT1 |
| 1.297428 | 4.40E-06 | GPT2 |
| 1.106642 | 4.45E-06 | NGRN |
| 1.116521 | 4.55E-06 | LETM2 |
| 1.116391 | 4.56E-06 | RGS2 |
| 1.198373 | 4.60E-06 | ZNF473 |
| 1.05239 | 4.62E-06 | FEN1 |
| 1.252369 | 4.64E-06 | RAD51AP1 |
| 0.999377 | 4.65E-06 | PIGW |
| 0.988329 | 4.67E-06 | C9orf91 |
| 1.091864 | 4.72E-06 | TYMS |
| 1.049464 | 4.75E-06 | TUBB2B |
| 1.172292 | 4.81E-06 | TAF1A |
| 1.093706 | 4.86E-06 | ZC3H8 |
| 1.153434 | 5.07E-06 | C10orf118 |
| 1.306048 | 5.15E-06 | RCAN1 |
| 1.118531 | 5.25E-06 | RFC2 |
| 1.336473 | 5.25E-06 | LOC100506342 |
| 0.975768 | 5.27E-06 | NOLC1 |
| 1.131335 | 5.29E-06 | SELRC1 |
| 1.047204 | 5.34E-06 | RFWD3 |
| 0.990574 | 5.35E-06 | ORC6 |
| 1.066693 | 5.45E-06 | GLRX2 |
| 1.236755 | 5.46E-06 | SDSL |
| 1.005979 | 5.98E-06 | CCNB1IP1 |
| 0.973078 | 5.99E-06 | SOCS4 |
| 1.045713 | 6.01E-06 | AUNIP |
| 1.041427 | 6.02E-06 | LOC81691 |
| 1.017295 | 6.09E-06 | DNMT1 |
| 1.013177 | 6.12E-06 | ZWILCH |
| 1.160833 | 6.14E-06 | RFC3 |
| 1.103373 | 6.26E-06 | TK1 |
| 0.999438 | 6.42E-06 | CDC45 |
| 0.953707 | 6.49E-06 | BRIP1 |
| 0.983992 | 6.55E-06 | EPT1 |
| 1.014602 | 6.61E-06 | LINC00467 |
| 1.338115 | 6.76E-06 | FANCB |
| 0.955596 | 6.82E-06 | SGK1 |
| 0.998993 | 6.85E-06 | GAS5 /// SNORD44 /// SNORD47 /// SNORD76 /// SNORD77 /// SNORD79 /// SNORD80 /// SNORD81 |
| 1.007449 | 7.00E-06 | TMEM40 |
| 0.976238 | 7.02E-06 | FAM53C |
| 1.114912 | 7.12E-06 | SLFN13 |
| 1.583852 | 7.12E-06 | AHR |
| 1.497944 | 7.22E-06 | FST |
| 1.069418 | 7.33E-06 | C1orf54 |
| 0.926721 | 7.36E-06 | SUV39H2 |
| 0.925121 | 7.44E-06 | EZH2 |
| 1.149669 | 7.44E-06 | RIPK2 |
| 0.952762 | 7.78E-06 | PDRG1 |
| 1.937846 | 7.96E-06 | MXD1 |
| 0.968505 | 8.12E-06 | RAB39B |
| 0.926293 | 8.30E-06 | SPRTN |
| 1.11782 | 8.33E-06 | APOL6 |
| 1.041867 | 8.43E-06 | PBLD |
| 0.905727 | 8.47E-06 | MIR1292 /// NOP56 /// SNORD110 /// SNORD57 /// SNORD86 |
| 1.059411 | 8.58E-06 | ZNF670 |
| 0.940025 | 8.67E-06 | ATF4 |
| 1.070876 | 8.70E-06 | PDP1 |
| 1.020655 | 8.70E-06 | SH2B3 |
| 0.932206 | 8.84E-06 | MTFR2 |
| 1.024725 | 8.84E-06 | PNP |
| 0.904215 | 8.84E-06 | MOSPD1 |
| 1.151465 | 9.30E-06 | CREB5 /// LOC401317 |
| 1.003291 | 9.33E-06 | SARNP |
| 0.999363 | 9.35E-06 | RELB |
| 0.945754 | 9.55E-06 | PPCDC |
| 1.053126 | 9.69E-06 | MARS |
| 1.36019 | 9.76E-06 | LRRN3 |
| 0.888158 | 9.77E-06 | YARS |
| 0.920335 | 9.86E-06 | SRP19 |
| 0.928783 | 1.00E-05 | E2F7 |
| 0.945945 | 1.01E-05 | TRMT6 |
| 1.449947 | 1.02E-05 | RGS17 |
| 1.316503 | 1.03E-05 | LOC374443 |
| 0.910697 | 1.04E-05 | RPAP3 |
| 1.167747 | 1.04E-05 | ASNS |
| 0.874115 | 1.08E-05 | CENPH |
| 0.917041 | 1.09E-05 | GOT1 |
| 0.97864 | 1.10E-05 | PHIP |
| 0.90288 | 1.11E-05 | PSMG1 |
| 1.536483 | 1.11E-05 | SNHG8 /// SNORA24 |
| 0.910952 | 1.12E-05 | ITGAM |
| 0.867338 | 1.12E-05 | RBM15 |
| 0.890476 | 1.14E-05 | CENPN |
| 0.89597 | 1.14E-05 | FANCG |
| 0.872633 | 1.17E-05 | WDR75 |
| 0.920468 | 1.18E-05 | PISD |
| 1.151436 | 1.18E-05 | RBM24 |
| 0.960736 | 1.19E-05 | MITF |
| 0.937837 | 1.19E-05 | TAF13 |
| 0.910765 | 1.22E-05 | NAA50 |
| 0.958404 | 1.22E-05 | TCF19 |
| 1.028023 | 1.22E-05 | HELLS |
| 1.011237 | 1.23E-05 | CNRIP1 |
| 0.970888 | 1.23E-05 | RAB9A |
| 0.895688 | 1.24E-05 | CXCL1 |
| 1.769432 | 1.26E-05 | BEX1 |
| 1.36511 | 1.27E-05 | TMEM140 |
| 0.854423 | 1.27E-05 | POLA1 |
| 0.873586 | 1.28E-05 | AVPI1 |
| 0.86537 | 1.29E-05 | PCNA |
| 1.226554 | 1.30E-05 | BIK |
| 0.849985 | 1.31E-05 | MDM1 |
| 1.978559 | 1.36E-05 | TAC1 |
| 0.935125 | 1.38E-05 | PTP4A1 |
| 0.888169 | 1.38E-05 | USP53 |
| 0.933113 | 1.39E-05 | PTGER4 |
| 0.896899 | 1.40E-05 | THAP10 |
| 0.92195 | 1.40E-05 | ULBP2 |
| 1.164188 | 1.40E-05 | OSBPL6 |
| 0.898289 | 1.41E-05 | PHC3 |
| 0.867567 | 1.41E-05 | INPP1 |
| 0.86352 | 1.42E-05 | FAM132B |
| 1.22601 | 1.42E-05 | DUSP3 |
| 0.847045 | 1.44E-05 | ARL5B |
| 0.951731 | 1.44E-05 | GOLT1B |
| 0.877137 | 1.44E-05 | RMI1 |
| 0.871035 | 1.46E-05 | SENP1 |
| 0.914127 | 1.46E-05 | TMEM138 |
| 1.77888 | 1.47E-05 | C11orf96 |
| 0.831535 | 1.49E-05 | WDR74 |
| 0.871478 | 1.51E-05 | RNF138 |
| 0.998755 | 1.51E-05 | BROX |
| 0.841311 | 1.52E-05 | SWAP70 |
| 1.090138 | 1.54E-05 | TXNL4B |
| 0.915429 | 1.54E-05 | ABHD3 |
| 1.38782 | 1.55E-05 | USP18 |
| 0.863227 | 1.56E-05 | PPP1R15B |
| 0.923635 | 1.58E-05 | LRRC57 |
| 1.192654 | 1.58E-05 | UBIAD1 |
| 0.88098 | 1.59E-05 | SLC22A15 |
| 1.345565 | 1.60E-05 | LONRF3 |
| 0.831442 | 1.61E-05 | ADAT1 |
| 0.856804 | 1.62E-05 | MED10 |
| 0.831759 | 1.62E-05 | SLC20A1 |
| 0.912294 | 1.63E-05 | RRS1 |
| 0.862051 | 1.64E-05 | UHRF1 |
| 0.874957 | 1.65E-05 | CYP20A1 |
| 0.909099 | 1.67E-05 | SLC16A6 |
| 1.048191 | 1.68E-05 | SLC6A15 |
| 0.943728 | 1.70E-05 | C16orf93 |
| 0.837132 | 1.70E-05 | MLF1IP |
| 0.923254 | 1.72E-05 | GINS3 |
| 1.051791 | 1.72E-05 | KLF6 |
| 0.843059 | 1.73E-05 | MELK |
| 0.836451 | 1.74E-05 | MIR4723 /// TMEM199 |
| 0.841795 | 1.76E-05 | URB2 |
| 1.148822 | 1.77E-05 | ELOVL4 |
| 1.222179 | 1.77E-05 | PINX1 |
| 0.820894 | 1.78E-05 | PRRG4 |
| 0.857655 | 1.80E-05 | RAB27A |
| 0.810503 | 1.81E-05 | STIL |
| 1.024891 | 1.83E-05 | ZNF567 |
| 1.243724 | 1.87E-05 | ADRB2 |
| 0.826219 | 1.87E-05 | RPA2 |
| 0.939243 | 1.89E-05 | NRIP3 |
| 0.826302 | 1.92E-05 | NOP2 |
| 0.801811 | 1.93E-05 | CCDC59 |
| 0.842662 | 1.94E-05 | PHF5A |
| 0.932389 | 1.94E-05 | FAM24B |
| 0.83815 | 1.94E-05 | PTPDC1 |
| 0.874561 | 1.94E-05 | BDNF |
| 0.802405 | 1.94E-05 | SRSF7 |
| 0.847512 | 1.96E-05 | NGF |
| 0.842602 | 1.97E-05 | SENP5 |
| 0.857476 | 1.98E-05 | DNAJC1 |
| 1.406075 | 2.01E-05 | PTPRR |
| 0.803551 | 2.01E-05 | LCP1 |
| 1.133405 | 2.02E-05 | TNFRSF9 |
| 0.855458 | 2.03E-05 | LOC377711 /// MROH1 |
| 0.945882 | 2.11E-05 | FKBP5 |
| 0.810106 | 2.12E-05 | BCAS2 |
| 0.985012 | 2.13E-05 | ZNF280C |
| 0.794019 | 2.14E-05 | NIP7 |
| 0.800945 | 2.15E-05 | RNF166 |
| 1.098154 | 2.15E-05 | LPL |
| 0.856386 | 2.16E-05 | AKIP1 |
| 0.79765 | 2.16E-05 | ZNF682 |
| 0.808291 | 2.19E-05 | MRPS31P5 /// THSD1 |
| 0.811104 | 2.19E-05 | CAMTA2 |
| 0.840665 | 2.21E-05 | RPL22L1 |
| 0.854031 | 2.21E-05 | EXOC8 |
| 1.051277 | 2.23E-05 | CCDC15 |
| 1.702283 | 2.25E-05 | SLC25A25 |
| 0.781837 | 2.28E-05 | RNF25 |
| 0.779643 | 2.29E-05 | MAP1LC3B |
| 0.844667 | 2.30E-05 | NUP153 |
| 1.124676 | 2.30E-05 | RNMTL1 |
| 0.795604 | 2.31E-05 | ZCCHC8 |
| 0.88581 | 2.31E-05 | IFI16 |
| 1.084194 | 2.37E-05 | TLR3 |
| 0.802034 | 2.37E-05 | NABP1 |
| 1.09471 | 2.38E-05 | GDAP1 |
| 0.780396 | 2.40E-05 | ZNF655 |
| 0.83656 | 2.41E-05 | CSRP2 |
| 0.81023 | 2.41E-05 | USP37 |
| 0.842761 | 2.43E-05 | JMY |
| 0.862822 | 2.43E-05 | RRN3 |
| 0.832874 | 2.43E-05 | LOC100509445 /// LOC728715 /// OVOS2 |
| 0.799521 | 2.44E-05 | BRCA1 |
| 1.307735 | 2.47E-05 | MICB |
| 1.187966 | 2.48E-05 | RTP4 |
| 0.82654 | 2.54E-05 | GJC1 |
| 0.843563 | 2.57E-05 | TRMT1 |
| 0.890365 | 2.58E-05 | RPF2 |
| 0.845124 | 2.60E-05 | IL4I1 |
| 0.838679 | 2.60E-05 | SDC1 |
| 0.785755 | 2.61E-05 | POLE |
| 0.762077 | 2.67E-05 | CCDC137 |
| 0.866184 | 2.70E-05 | POLR3F |
| 0.956156 | 2.75E-05 | ARNTL2 |
| 0.764072 | 2.78E-05 | MGME1 |
| 0.824412 | 2.79E-05 | DDX20 |
| 0.797677 | 2.81E-05 | BRIX1 |
| 0.823991 | 2.83E-05 | SKA3 |
| 0.817186 | 2.87E-05 | EID3 |
| 0.787683 | 2.87E-05 | NFXL1 |
| 0.888988 | 2.88E-05 | EIF4EBP1 |
| 0.905245 | 2.89E-05 | SERTAD1 |
| 0.795638 | 2.89E-05 | TUBB2A |
| 2.659527 | 2.89E-05 | PTGS2 |
| 1.044021 | 2.90E-05 | MYBL2 |
| 0.774912 | 2.90E-05 | ATP2B1 |
| 0.759164 | 2.90E-05 | ZFR |
| 0.834582 | 2.90E-05 | IL18 |
| 0.83508 | 2.91E-05 | IRF1 |
| 0.763047 | 2.96E-05 | SRPRB |
| 0.804355 | 2.97E-05 | NCAPG2 |
| 0.807864 | 2.97E-05 | CCDC86 |
| 0.796172 | 2.99E-05 | ARHGEF2 |
| 0.778071 | 3.00E-05 | IWS1 |
| 0.896123 | 3.03E-05 | SRD5A1 |
| 1.394218 | 3.06E-05 | RRAD |
| 0.840357 | 3.07E-05 | SNX8 |
| 1.020376 | 3.08E-05 | EDN1 |
| 0.832592 | 3.17E-05 | CHAF1A |
| 0.883361 | 3.19E-05 | NOC3L |
| 1.117666 | 3.22E-05 | DSN1 |
| 0.760297 | 3.23E-05 | FGFR1OP |
| 0.827237 | 3.25E-05 | SFR1 |
| 0.77062 | 3.30E-05 | ZWINT |
| 0.777143 | 3.31E-05 | PCK2 |
| 0.774042 | 3.32E-05 | MICALL1 |
| 1.082265 | 3.33E-05 | TNFSF15 |
| 0.909608 | 3.34E-05 | DUSP5 |
| 0.767687 | 3.35E-05 | SMIM13 |
| 0.961608 | 3.36E-05 | XAF1 |
| 1.042934 | 3.38E-05 | LRRC49 |
| 0.775259 | 3.38E-05 | NXT1 |
| 1.302663 | 3.38E-05 | IFIT2 |
| 0.818253 | 3.43E-05 | OTTHUMG00000018458 /// RP11-152N13.5 |
| 0.790482 | 3.43E-05 | TRIM26 |
| 0.762831 | 3.44E-05 | JPH1 |
| 0.796937 | 3.44E-05 | NEDD4 |
| 0.751854 | 3.45E-05 | CMC2 |
| 1.021986 | 3.46E-05 | TTBK2 |
| 0.740333 | 3.47E-05 | NMD3 |
| 0.802856 | 3.49E-05 | P2RX7 |
| 0.750831 | 3.53E-05 | RIOK3 |
| 0.732161 | 3.55E-05 | CIRH1A |
| 0.937765 | 3.56E-05 | ZFAS1 |
| 0.794951 | 3.58E-05 | SCLT1 |
| 0.763359 | 3.59E-05 | KNTC1 |
| 0.940114 | 3.62E-05 | PTGES |
| 0.745358 | 3.63E-05 | LYAR |
| 0.740234 | 3.64E-05 | CENPO |
| 0.780081 | 3.65E-05 | GGCT |
| 0.754269 | 3.73E-05 | PALB2 |
| 0.740799 | 3.74E-05 | FBXO5 |
| 0.749895 | 3.75E-05 | TIMELESS |
| 0.817165 | 3.77E-05 | CHAF1B |
| 1.000034 | 3.78E-05 | FANCI |
| 1.124629 | 3.80E-05 | FLT1 |
| 0.766436 | 3.83E-05 | PPTC7 |
| 1.318085 | 3.85E-05 | PTHLH |
| 0.731395 | 3.85E-05 | RIT1 |
| 0.75619 | 3.86E-05 | USP36 |
| 0.945113 | 3.89E-05 | FAM86B1 /// FAM86B2 /// FAM86C1 /// FAM86DP /// FAM86FP |
| 0.758763 | 3.96E-05 | USP31 |
| 0.764624 | 3.98E-05 | SDE2 |
| 1.342326 | 4.02E-05 | LOC100506377 |
| 0.746493 | 4.02E-05 | ZNF331 |
| 0.736271 | 4.07E-05 | DDR2 |
| 0.73311 | 4.10E-05 | LOC284023 |
| 0.810769 | 4.11E-05 | GEN1 |
| 0.798619 | 4.13E-05 | NUPL1 |
| 0.86957 | 4.13E-05 | KIAA0101 |
| 0.823069 | 4.14E-05 | LOC100131067 |
| 0.768457 | 4.15E-05 | CREM |
| 0.869613 | 4.16E-05 | C3orf52 |
| 0.829577 | 4.18E-05 | LOC100506392 |
| 0.80108 | 4.19E-05 | ZCCHC10 |
| 0.885989 | 4.26E-05 | MAFF |
| 0.755718 | 4.28E-05 | XK |
| 0.71936 | 4.32E-05 | LENG1 |
| 1.210992 | 4.35E-05 | TNFAIP3 |
| 0.752335 | 4.35E-05 | SURF2 |
| 0.761306 | 4.40E-05 | CTSL2 |
| 0.712118 | 4.43E-05 | RIPPLY3 |
| 0.72855 | 4.45E-05 | LRRC58 |
| 0.718575 | 4.48E-05 | FANCA |
| 0.729279 | 4.49E-05 | RMI2 |
| 0.915144 | 4.54E-05 | ECM2 |
| 1.612791 | 4.54E-05 | CXCL10 |
| 0.867296 | 4.55E-05 | GRPEL2 |
| 1.015532 | 4.58E-05 | EIF3C |
| 0.700862 | 4.59E-05 | COL16A1 |
| 0.726649 | 4.61E-05 | SLC1A4 |
| 1.000789 | 4.63E-05 | RASEF |
| 0.737023 | 4.64E-05 | NDUFAF2 |
| 0.714235 | 4.66E-05 | RAB23 |
| 0.788004 | 4.68E-05 | CASP7 |
| 0.703675 | 4.74E-05 | REV3L |
| 0.770651 | 4.76E-05 | AIMP2 |
| 0.737231 | 4.80E-05 | FAS |
| 0.708816 | 4.80E-05 | HAT1 |
| 0.747387 | 4.81E-05 | RABGGTB /// SNORD45A /// SNORD45B /// SNORD45C |
| 1.257731 | 4.88E-05 | PLA2G4A |
| 0.723635 | 4.90E-05 | ZNF259 /// ZNF259P1 |
| 0.705388 | 4.90E-05 | DSCC1 |
| 1.026869 | 4.90E-05 | P2RX4 |
| 0.777346 | 4.96E-05 | TMEM47 |
| 0.693915 | 4.97E-05 | CCDC93 |
| 0.790031 | 5.00E-05 | SRA1 |
| 0.823674 | 5.01E-05 | PKMYT1 |
| 0.974389 | 5.02E-05 | C1QTNF9B-AS1 |
| 0.767935 | 5.03E-05 | POLE3 |
| 0.915658 | 5.09E-05 | PGBD1 |
| 0.691879 | 5.09E-05 | MMD |
| 0.760791 | 5.12E-05 | NAF1 |
| 1.025609 | 5.17E-05 | PNPT1 |
| 0.729873 | 5.19E-05 | ZNF443 |
| 0.792771 | 5.23E-05 | TRIM35 |
| 0.816689 | 5.29E-05 | FAM50A |
| 0.870453 | 5.30E-05 | RARRES3 |
| 0.794926 | 5.40E-05 | BUD13 |
| 0.771696 | 5.48E-05 | C1orf109 |
| 0.717658 | 5.49E-05 | ZNF311 |
| 0.702408 | 5.50E-05 | PSME3 |
| 1.02128 | 5.52E-05 | SPHK1 |
| 0.682036 | 5.52E-05 | FIP1L1 |
| 0.818654 | 5.54E-05 | LOC100129518 /// SOD2 |
| 0.692827 | 5.55E-05 | NUFIP1 |
| 1.455826 | 5.58E-05 | KLF4 |
| 0.712571 | 5.58E-05 | LPIN2 |
| 0.755166 | 5.58E-05 | PASK |
| 0.715919 | 5.61E-05 | RABGGTB |
| 1.194479 | 5.66E-05 | DACT1 |
| 0.74267 | 5.69E-05 | MYC |
| 0.759208 | 5.70E-05 | RECQL |
| 0.758339 | 5.72E-05 | WTAP |
| 1.061939 | 5.73E-05 | TCP11L1 |
| 1.422974 | 5.73E-05 | DLGAP1-AS2 |
| 0.676968 | 5.74E-05 | TOPORS |
| 0.751776 | 5.75E-05 | SLC25A28 |
| 0.750643 | 5.85E-05 | TNPO1 |
| 0.898695 | 5.89E-05 | FAM111A |
| 0.9439 | 5.90E-05 | C12orf44 |
| 0.700465 | 5.90E-05 | ZNF689 |
| 0.700764 | 5.97E-05 | MRPS31 |
| 0.781199 | 5.99E-05 | RNF114 |
| 1.043936 | 6.06E-05 | OAS2 |
| 1.677892 | 6.07E-05 | CXCL11 |
| 0.738996 | 6.07E-05 | MCM8 |
| 0.730901 | 6.10E-05 | LRR1 |
| 0.688143 | 6.11E-05 | GYG1 |
| 0.696676 | 6.12E-05 | SBDS /// SBDSP1 |
| 1.104707 | 6.21E-05 | SLC19A2 |
| 0.695105 | 6.25E-05 | RUSC1 |
| 0.856513 | 6.26E-05 | NFIL3 |
| 0.705927 | 6.31E-05 | COQ10A |
| 0.692464 | 6.31E-05 | SLC25A19 |
| 0.770528 | 6.33E-05 | CD83 |
| 1.426482 | 6.35E-05 | HRK /// LOC283454 |
| 0.812605 | 6.35E-05 | NMI |
| 0.807831 | 6.44E-05 | PHLDA1 |
| 0.755611 | 6.45E-05 | NUP155 |
| 0.671415 | 6.56E-05 | POLQ |
| 0.69031 | 6.57E-05 | HSPA9 |
| 0.68072 | 6.66E-05 | TRAPPC6B |
| 0.672357 | 6.71E-05 | MAK16 |
| 0.660312 | 6.78E-05 | TCOF1 |
| 0.667195 | 6.79E-05 | VRK1 |
| 1.32236 | 6.80E-05 | OASL |
| 0.813732 | 6.83E-05 | LOC100506714 |
| 0.746502 | 6.84E-05 | POLD3 |
| 0.749941 | 6.84E-05 | LAMA1 |
| 0.753333 | 6.85E-05 | SVIP |
| 0.688918 | 6.88E-05 | C1orf112 |
| 0.794353 | 6.93E-05 | MAP1B |
| 0.747983 | 6.95E-05 | ERI1 |
| 0.808011 | 6.98E-05 | ZNF697 |
| 0.719821 | 7.00E-05 | PAK1IP1 |
| 0.688063 | 7.04E-05 | GMNN |
| 0.769224 | 7.17E-05 | USP1 |
| 0.761073 | 7.19E-05 | ZNF441 |
| 0.700745 | 7.30E-05 | CRY1 |
| 0.652729 | 7.37E-05 | FKRP |
| 0.828914 | 7.42E-05 | CENPJ |
| 0.681102 | 7.42E-05 | CA13 /// LOC100507258 |
| 0.708972 | 7.44E-05 | LOC442075 |
| 0.689514 | 7.45E-05 | S1PR3 |
| 0.684611 | 7.46E-05 | ABRACL |
| 0.678914 | 7.53E-05 | CLIP1 |
| 0.667415 | 7.58E-05 | EXOSC8 |
| 0.683105 | 7.62E-05 | LOC100996643 /// MTHFD1L |
| 0.669477 | 7.67E-05 | AZIN1 |
| 0.669824 | 7.68E-05 | UAP1L1 |
| 0.761289 | 7.69E-05 | ANKRD20A1 /// ANKRD20A11P /// ANKRD20A2 /// ANKRD20A3 /// ANKRD20A4 /// ANKRD20A5P /// ANKRD20A9P /// LOC101059935 |
| 0.878777 | 7.69E-05 | ATAD5 |
| 1.520239 | 7.76E-05 | ISG20 |
| 0.680803 | 7.77E-05 | HAUS3 |
| 0.832333 | 7.79E-05 | ZNF257 |
| 0.659726 | 7.88E-05 | NET1 |
| 0.876442 | 8.00E-05 | MIER3 |
| 0.703111 | 8.13E-05 | C12orf4 |
| 0.706085 | 8.22E-05 | DR1 |
| 0.662965 | 8.25E-05 | TSR1 |
| 0.701739 | 8.27E-05 | NDUFS3 /// PTPMT1 |
| 0.743768 | 8.28E-05 | GAN |
| 0.683594 | 8.30E-05 | ZNF625 /// ZNF625-ZNF20 |
| 0.655973 | 8.30E-05 | FIGNL1 |
| 0.644277 | 8.40E-05 | SLU7 |
| 0.737265 | 8.44E-05 | SLC25A32 |
| 0.638813 | 8.56E-05 | UTP23 |
| 0.663373 | 8.57E-05 | UTP15 |
| 0.644955 | 8.59E-05 | C2orf49 |
| 0.762041 | 8.64E-05 | NEIL3 |
| 1.18678 | 8.65E-05 | ADAMTS5 |
| 0.704787 | 8.67E-05 | MCM5 |
| 0.674263 | 8.72E-05 | ZBTB43 |
| 0.869512 | 8.76E-05 | PSPC1 |
| 0.779525 | 8.83E-05 | KPNA4 |
| 0.760374 | 8.84E-05 | LUC7L |
| 0.660204 | 8.87E-05 | MAD2L1BP |
| 0.830733 | 8.88E-05 | ORC1 |
| 0.868012 | 8.89E-05 | PPRC1 |
| 0.713367 | 8.96E-05 | BAZ1A |
| 0.732807 | 9.01E-05 | GINS4 |
| 0.648681 | 9.05E-05 | TMEM181 |
| 0.652252 | 9.08E-05 | KRCC1 |
| 0.737778 | 9.11E-05 | ORC5 |
| 0.796552 | 9.20E-05 | HES6 |
| 0.628979 | 9.21E-05 | POLR3D |
| 0.838851 | 9.22E-05 | SSSCA1 |
| 0.956998 | 9.28E-05 | IFI35 |
| 0.674381 | 9.29E-05 | EIF6 |
| 0.708544 | 9.30E-05 | CCNT1 |
| 0.834544 | 9.32E-05 | DIO2 |
| 1.471949 | 9.41E-05 | TNFAIP6 |
| 0.670886 | 9.42E-05 | UBR7 |
| 0.655823 | 9.43E-05 | PCGF6 |
| 1.005001 | 9.45E-05 | HERC5 |
| 0.62913 | 9.49E-05 | NSUN5 |
| 0.672946 | 9.53E-05 | PALLD |
| 0.65096 | 9.55E-05 | OSBPL11 |
| 0.8068 | 9.69E-05 | MCM4 |
| 0.656512 | 9.70E-05 | XPO5 |
| 0.676975 | 9.71E-05 | ZNF326 |
| 0.731986 | 9.71E-05 | SLC7A6OS |
| 0.758214 | 9.71E-05 | LOC100506661 |
| 0.648807 | 9.73E-05 | CAND2 |
| 0.899655 | 9.76E-05 | ANKLE1 |
| 0.631363 | 9.91E-05 | SAMD4A |
| 0.750987 | 0.000101 | SAMHD1 |
| 0.914025 | 0.000101 | SHOX2 |
| 0.653174 | 0.000102 | FDX1L |
| 0.718413 | 0.000102 | CDC7 |
| 0.62552 | 0.000102 | MTPAP |
| 0.624794 | 0.000103 | CIDEC |
| 0.628413 | 0.000104 | ARF6 |
| 0.637378 | 0.000104 | BEX2 |
| 0.644649 | 0.000104 | YRDC |
| 0.642767 | 0.000105 | CDK11A /// CDK11B |
| 0.726285 | 0.000105 | CYP1B1 |
| 1.071712 | 0.000105 | IFIT3 |
| 0.678482 | 0.000105 | AMIGO2 |
| 0.641318 | 0.000105 | PPIG |
| 0.637945 | 0.000105 | COPS3 |
| 0.637948 | 0.000105 | IFRD2 |
| 0.773874 | 0.000106 | CDCA7 |
| 0.616566 | 0.000106 | CDC23 |
| 0.754131 | 0.000106 | ZNF280B |
| 0.626654 | 0.000107 | RGMB |
| 0.652984 | 0.000107 | YKT6 |
| 0.727518 | 0.000107 | ZNHIT2 |
| 0.710874 | 0.000107 | BIRC3 |
| 0.656888 | 0.000108 | TIMM44 |
| 0.649289 | 0.000109 | LMBRD2 |
| 0.768973 | 0.00011 | C19orf48 /// SNORD88C |
| 0.870347 | 0.00011 | SLC30A1 |
| 0.614028 | 0.00011 | ZFP3 |
| 0.627933 | 0.00011 | CD55 |
| 0.729775 | 0.000111 | SEC22B |
| 0.674898 | 0.000111 | NCAPD3 |
| 0.654474 | 0.000112 | MBD4 |
| 0.798662 | 0.000112 | RAD51 |
| 0.621546 | 0.000112 | FBXO45 |
| 0.617383 | 0.000113 | GINS1 |
| 0.764523 | 0.000115 | SMC6 |
| 0.623523 | 0.000116 | MRPL37 |
| 0.608697 | 0.000116 | CCNE1 |
| 0.627273 | 0.000116 | YTHDF1 |
| 0.638875 | 0.000116 | UAP1 |
| 0.623519 | 0.000116 | SPC25 |
| 0.692364 | 0.000119 | TAF4B |
| 0.687959 | 0.000119 | ZNF23 |
| 0.74144 | 0.000119 | MYO3A |
| 0.755634 | 0.000119 | MSH2 |
| 0.668489 | 0.000119 | IER5 |
| 1.229394 | 0.00012 | SAT1 |
| 0.91058 | 0.00012 | PHGDH |
| 0.729539 | 0.00012 | TFAM |
| 0.653379 | 0.000121 | SMCO4 |
| 0.603214 | 0.000121 | MID1IP1 |
| 0.927326 | 0.000121 | OTTHUMG00000183927 /// RP11-248J18.2 |
| 0.736017 | 0.000121 | TMEM154 |
| 0.721192 | 0.000121 | COCH |
| 0.622333 | 0.000121 | SUGT1 |
| 0.661325 | 0.000123 | ZNF259 |
| 0.737667 | 0.000123 | RAD54L |
| 0.60273 | 0.000123 | LOC101060478 /// RNF115 |
| 0.616556 | 0.000124 | TCHP |
| 0.665676 | 0.000125 | ETHE1 |
| 0.879053 | 0.000126 | RASSF1 |
| 0.626052 | 0.000128 | ATR |
| 0.64329 | 0.000128 | DUSP12 |
| 0.600619 | 0.000129 | IMPA1 |
| 0.620542 | 0.000129 | SAR1A |
| 0.625974 | 0.000129 | CPEB1 |
| 0.686886 | 0.00013 | BLZF1 |
| 0.696617 | 0.000132 | RRAGC |
| 1.22396 | 0.000135 | IL32 |
| 0.594861 | 0.000135 | HSPB11 |
| 0.5937 | 0.000136 | NVL |
| 0.886312 | 0.000136 | SLC35F2 |
| 0.6187 | 0.000136 | DDX5 /// MIR3064 /// MIR5047 |
| 0.709051 | 0.000137 | C12orf29 |
| 0.807047 | 0.000137 | SLC31A2 |
| 0.715401 | 0.000137 | PSMC3 |
| 0.700407 | 0.000137 | FAM122C |
| 0.676979 | 0.000138 | TMEM200A |
| 0.666643 | 0.000138 | KLF11 |
| 0.702649 | 0.000139 | TOE1 |
| 0.68536 | 0.00014 | ARL13B |
| 0.677644 | 0.000144 | BOD1L1 |
| 0.648964 | 0.000144 | CHUK |
| 0.59481 | 0.000145 | RFC4 |
| 0.620591 | 0.000145 | FANCM |
| 0.639557 | 0.000145 | E2F6 |
| 0.63769 | 0.000147 | FBF1 |
| 0.635163 | 0.000148 | KIAA1279 |
| 0.89656 | 0.000149 | JUNB |
| 0.62183 | 0.000149 | ZNF57 |
| 0.654246 | 0.000149 | RAD1 |
| 0.632766 | 0.000149 | SNAP29 |
| 0.744197 | 0.00015 | PDCD2L |
| 0.889795 | 0.00015 | SLC25A33 |
| 0.619034 | 0.00015 | OGFOD1 |
| 0.595785 | 0.000151 | FAM76A |
| 0.763515 | 0.000152 | CAMTA1 |
| 0.627358 | 0.000153 | HES4 |
| 0.629311 | 0.000153 | PIDD1 |
| 0.747113 | 0.000154 | NEXN |
| 0.84969 | 0.000154 | DUSP2 |
| 0.626885 | 0.000155 | PMS1 |
| 0.689314 | 0.000155 | FAM169A |
| 0.679079 | 0.000155 | TRAF4 |
| 0.938551 | 0.000156 | C18orf54 |
| 0.596567 | 0.000156 | MRPL33 |
| 0.655751 | 0.000159 | EMG1 |
| 0.601136 | 0.00016 | DNAJC15 |
| 0.607766 | 0.00016 | CDCA4 |
| 0.590734 | 0.000161 | RNF19B |
| 0.64135 | 0.000163 | SPIN4 |
| 0.710551 | 0.000163 | TTC4 |
| 0.792636 | 0.000163 | CHRNA5 |
| 0.717773 | 0.000163 | TPR |
| 0.634552 | 0.000163 | DUSP10 |
| 0.591645 | 0.000163 | OGFRL1 |
| 1.431178 | 0.000164 | NEURL3 |
| 0.592139 | 0.000164 | HOXB9 |
| 0.683165 | 0.000165 | BYSL |
| 0.598138 | 0.000165 | CPSF3 |
| 0.70558 | 0.000166 | PLAGL1 |
| 0.660685 | 0.000167 | SLC4A7 |
| 0.708007 | 0.000169 | PAQR3 |
| 0.801852 | 0.00017 | EXO5 |
| 0.673823 | 0.000172 | RBBP5 |
| 1.605293 | 0.000172 | GEM |
| 0.602026 | 0.000172 | XBP1 |
| 0.589415 | 0.000173 | CCDC101 |
| 0.830225 | 0.000173 | OTTHUMG00000015043 /// RP11-554D15.1 |
| 0.625618 | 0.000176 | LSM6 |
| 1.368211 | 0.000178 | DDX60L |
| 0.881249 | 0.000178 | LRCH2 |
| 0.672855 | 0.000178 | PUS3 |
| 0.599661 | 0.000178 | NUPL2 |
| 0.621605 | 0.000181 | C5orf22 |
| 0.647163 | 0.000181 | DPH3 |
| 0.678149 | 0.000182 | DNA2 |
| 0.688173 | 0.000182 | TMEM106C |
| 0.654305 | 0.000182 | ANKRD20A11P |
| 0.732448 | 0.000183 | CCDC82 |
| 0.650107 | 0.000183 | AC099850.1 /// OTTHUMG00000132079 |
| 0.602621 | 0.000183 | SAC3D1 |
| 0.714777 | 0.000185 | ASRGL1 |
| 0.611676 | 0.000185 | RAB21 |
| 0.984113 | 0.000185 | C8orf48 |
| 0.595962 | 0.000186 | LOC100506639 /// ZNF131 |
| 0.856875 | 0.000188 | SUSD5 |
| 0.588175 | 0.000188 | PRR24 |
| 0.588708 | 0.000188 | SCO1 |
| 0.596165 | 0.000189 | NAPG |
| 0.850868 | 0.00019 | NCEH1 |
| 0.694564 | 0.000191 | AP1S2 |
| 0.66487 | 0.000191 | ZNF711 |
| 0.766903 | 0.000192 | SCLY |
| 0.597518 | 0.000192 | SOGA2 |
| 0.608305 | 0.000193 | ZNF85 |
| 0.635849 | 0.000194 | DDB2 |
| 0.587935 | 0.000194 | CELSR3 |
| 0.64675 | 0.000196 | NR2C2AP |
| 0.644259 | 0.000196 | CCPG1 /// DYX1C1-CCPG1 |
| 0.612534 | 0.000198 | CRYBB2 /// CRYBB2P1 |
| 0.666731 | 0.000198 | ZNF675 |
| 0.612715 | 0.000198 | MTO1 |
| 0.605282 | 0.000198 | PCGF5 |
| 0.739334 | 0.000198 | CITED2 |
| 0.589266 | 0.000199 | MAPK6 |
| 0.979456 | 0.000199 | ZNF571 |
| 0.601439 | 0.0002 | SLC25A16 |
| 0.713516 | 0.000202 | CLUHP3 |
| 0.632573 | 0.000204 | MIS12 |
| 0.651098 | 0.000204 | CNST |
| 0.638327 | 0.000205 | NFE2L2 |
| 0.683072 | 0.000207 | PROSER1 |
| 0.58824 | 0.00021 | HCCS |
| 0.755463 | 0.00021 | FAM86A |
| 0.622585 | 0.000213 | OAS3 |
| 0.608951 | 0.000214 | FRG1 /// LOC100289097 /// LOC100996779 |
| 0.591159 | 0.000214 | SNHG11 /// SNORA39 /// SNORA60 |
| 0.833013 | 0.000215 | CTSC |
| 0.627328 | 0.000216 | CHTF18 |
| 0.608227 | 0.000217 | GK3P |
| 0.609451 | 0.000217 | PPM1E |
| 0.644148 | 0.000218 | DDX11 |
| 1.73915 | 0.000218 | MIR155 /// MIR155HG |
| 0.726631 | 0.00022 | C9orf142 |
| 0.748039 | 0.000223 | SNX16 |
| 0.596159 | 0.000224 | ADPRHL2 |
| 0.615384 | 0.000226 | NUP62CL |
| 0.597293 | 0.000227 | AC017002.2 /// OTTHUMG00000153694 |
| 0.615417 | 0.000228 | FH |
| 0.601135 | 0.000228 | HINT2 |
| 0.702391 | 0.000229 | ANKRD30B |
| 0.629303 | 0.000231 | LOC283658 /// PYGO1 |
| 0.795256 | 0.000233 | ALKBH3 |
| 0.601236 | 0.000238 | PSMG3 |
| 0.748726 | 0.000238 | SLFN5 |
| 0.618425 | 0.000238 | LZTS3 |
| 0.624668 | 0.000239 | FAM161B |
| 0.720203 | 0.000245 | NUP43 |
| 0.612293 | 0.000248 | CDADC1 |
| 0.621261 | 0.000251 | FAM178A |
| 0.596522 | 0.000251 | LIN52 |
| 0.684169 | 0.000252 | QPCT |
| 0.945699 | 0.000253 | KIF24 |
| 0.617646 | 0.000254 | MIR1304 /// SNORA1 /// SNORA18 /// SNORA32 /// SNORA40 /// SNORA8 /// SNORD5 /// TAF1D |
| 0.684539 | 0.000255 | GBP3 |
| 0.618554 | 0.000258 | DNAJB9 |
| 1.245121 | 0.000258 | RSAD2 |
| 0.604072 | 0.00026 | ZNF597 |
| 0.71419 | 0.00026 | CCPG1 |
| 0.709721 | 0.000264 | AP3B2 |
| 0.647417 | 0.000266 | NFKBIE |
| 0.598351 | 0.000266 | MKI67IP |
| 0.668298 | 0.00027 | TARS |
| 0.624751 | 0.000274 | MAFG |
| 0.619533 | 0.000275 | ESF1 |
| 0.928963 | 0.000276 | EPHA4 |
| 1.165274 | 0.000277 | DUSP6 |
| 0.76472 | 0.00028 | ZSCAN12 |
| 0.62182 | 0.000281 | SLC7A3 |
| 0.595906 | 0.000282 | NTMT1 |
| 1.234762 | 0.000283 | ZNF530 |
| 0.827975 | 0.000284 | ASF1B |
| 0.729129 | 0.000289 | MYBL1 |
| 1.089844 | 0.00029 | KYNU |
| 0.588103 | 0.000291 | DCLRE1B |
| 0.594913 | 0.000293 | MARCH1 |
| 0.637554 | 0.000297 | SNRPA1 |
| 0.616056 | 0.000297 | MTX3 |
| 0.632827 | 0.000298 | SRBD1 |
| 0.77144 | 0.000299 | THAP9 |
| 0.63117 | 0.0003 | KCNMB3 |
| 0.630111 | 0.000301 | ZNF593 |
| 0.608648 | 0.000304 | ZNF850 |
| 0.649879 | 0.000305 | NPY |
| 1.014835 | 0.000306 | ETV5 |
| 0.654664 | 0.000314 | ABHD5 |
| 0.590796 | 0.000316 | PIGA |
| 0.729652 | 0.000319 | CDR2 |
| 0.838672 | 0.000323 | HS3ST3A1 |
| 0.702709 | 0.000328 | CHM |
| 0.805755 | 0.000331 | WDR53 |
| 0.72038 | 0.000336 | POU4F2 |
| 0.655507 | 0.000337 | CEP76 |
| 0.601541 | 0.00034 | CDC5L |
| 0.782777 | 0.000343 | GBP1 |
| 0.602413 | 0.000343 | JUN |
| 0.625979 | 0.000344 | CAMK2D |
| 0.62629 | 0.000345 | METTL1 |
| 1.35113 | 0.000347 | HSD17B6 |
| 0.597949 | 0.00035 | SKIL |
| 0.600769 | 0.000354 | TNFRSF10B |
| 0.644814 | 0.000372 | SPAG1 |
| 0.585339 | 0.000373 | CDK5RAP1 |
| 0.728994 | 0.000377 | SAE1 |
| 0.774552 | 0.000377 | GZF1 |
| 0.596125 | 0.00038 | TIGD2 |
| 0.603192 | 0.000381 | EED |
| 0.617489 | 0.000388 | LOC100506651 |
| 0.604122 | 0.000391 | AZI2 |
| 0.729123 | 0.000393 | TLE4 |
| 1.056865 | 0.000397 | H1F0 |
| 0.654486 | 0.000397 | IER2 |
| 0.607216 | 0.000397 | ISL1 |
| 0.981964 | 0.0004 | ZSCAN16 |
| 0.806551 | 0.000401 | TMEM217 |
| 0.630845 | 0.000405 | EFNB2 |
| 0.780336 | 0.00041 | KLF10 |
| 1.155911 | 0.000432 | PLA2G4C |
| 0.741484 | 0.000444 | SOD2 |
| 0.622563 | 0.000446 | EML6 |
| 0.613231 | 0.000451 | MIR503HG |
| 0.637952 | 0.000453 | MSH5 /// MSH5-SAPCD1 /// SAPCD1 |
| 0.821599 | 0.000453 | CCDC18 |
| 0.714894 | 0.000456 | PLAUR |
| 0.602612 | 0.000458 | FAM126B |
| 0.617796 | 0.00046 | PPIL4 |
| 0.668059 | 0.000469 | ZNF662 |
| 0.731007 | 0.000482 | PRDM13 |
| 1.056168 | 0.000488 | ZNF442 |
| 0.629645 | 0.000494 | SETDB2 |
| 0.714588 | 0.000498 | MIR17 /// MIR17HG /// MIR18A /// MIR19A /// MIR19B1 /// MIR20A /// MIR92A1 |
| 0.687842 | 0.000503 | TUBG1 |
| 0.641498 | 0.000508 | PLEKHF2 |
| 0.594009 | 0.00051 | PIK3R3 |
| 0.895596 | 0.000521 | SLC1A5 |
| 0.669566 | 0.000527 | NAA16 |
| 0.682178 | 0.000535 | CEP290 |
| 0.714901 | 0.000539 | ZNF649 |
| 0.971141 | 0.000539 | ZBTB42 |
| 0.608959 | 0.000541 | NUDT21 |
| 0.611347 | 0.000545 | GSG2 |
| 0.902013 | 0.000554 | FOSL1 |
| 1.285041 | 0.000562 | EGR1 |
| 0.600493 | 0.000571 | SYNGR3 |
| 0.748596 | 0.000584 | JAG1 |
| 0.887645 | 0.000586 | ADRB1 |
| 0.619416 | 0.000597 | RIOK1 |
| 0.9806 | 0.000598 | CCL20 |
| 1.490752 | 0.000601 | PCSK1 |
| 0.596487 | 0.000607 | ZC3H12C |
| 0.592831 | 0.000612 | CCDC134 |
| 0.645508 | 0.000614 | OTTHUMG00000183089 /// RP11-399K21.12 |
| 0.609602 | 0.000617 | SLC16A7 |
| 0.682953 | 0.000618 | SLMO1 |
| 0.598187 | 0.000621 | ZNFX1 |
| 0.861524 | 0.000625 | FAM161A |
| 0.739693 | 0.00063 | RPL39L |
| 0.826449 | 0.000638 | CMPK2 |
| 0.594866 | 0.000646 | MRPL39 |
| 0.609929 | 0.000646 | STAG3L4 |
| 0.739119 | 0.000672 | ISG20L2 |
| 0.600288 | 0.000684 | PCSK5 |
| 0.803432 | 0.000685 | ARIH2OS |
| 0.927028 | 0.00069 | IFIH1 |
| 0.614588 | 0.00071 | TAF3 |
| 0.611225 | 0.000733 | LY96 |
| 0.63184 | 0.000737 | C10orf88 |
| 0.711586 | 0.000739 | CYB5R2 |
| 0.672683 | 0.000742 | TOP3A |
| 0.759653 | 0.000748 | BATF3 |
| 0.622905 | 0.000764 | SLC43A2 |
| 0.766145 | 0.000782 | ADPRM |
| 0.93496 | 0.000807 | MARVELD2 |
| 0.587192 | 0.000807 | NR4A2 |
| 0.624464 | 0.000842 | OTTHUMG00000175497 /// RP6-24A23.7 |
| 0.72207 | 0.000851 | CYLD |
| 1.066101 | 0.000865 | FAM105A |
| 0.767085 | 0.000872 | KIF27 |
| 0.595991 | 0.000876 | HSPH1 |
| 0.629229 | 0.000877 | PSPH |
| 0.72939 | 0.000883 | LINC00669 |
| 0.920412 | 0.000889 | TM6SF1 |
| 0.638924 | 0.000923 | AREG /// AREGB |
| 0.652136 | 0.000937 | GAS2L3 |
| 0.625108 | 0.000939 | C10orf2 |
| 0.785935 | 0.000952 | LOC101060503 /// TXNIP |
| 0.63759 | 0.000993 | CD274 |
| 1.279704 | 0.001002 | TFPI2 |
| 1.181239 | 0.001013 | CCL3 /// CCL3L1 /// CCL3L3 /// LOC101060267 |
| 0.637552 | 0.001023 | OTTHUMG00000173051 /// RP5-991G20.4 |
| 0.640568 | 0.001029 | OAS1 |
| 0.589602 | 0.001066 | TES |
| 1.278577 | 0.00108 | CNN1 |
| 0.628643 | 0.001101 | NHS |
| 0.647708 | 0.001197 | FOSL2 |
| 0.667581 | 0.001265 | CA2 |
| 1.018335 | 0.0013 | LOC645638 |
| 0.654182 | 0.001352 | AOC2 |
| 0.598899 | 0.001352 | TONSL |
| 0.599162 | 0.001359 | RP9 /// RP9P |
| 0.724603 | 0.00138 | IPO11 /// LRRC70 |
| 0.714078 | 0.001429 | PAPPA |
| 0.593032 | 0.001437 | ATL3 |
| 0.902523 | 0.001455 | EPSTI1 |
| 0.692489 | 0.001547 | LIMK2 |
| 0.645032 | 0.001591 | IL15RA |
| 0.617443 | 0.001595 | AHI1 |
| 1.007549 | 0.001681 | RSPO3 |
| 0.669968 | 0.001711 | OTTHUMG00000168442 /// RP11-705C15.3 |
| 0.593473 | 0.001712 | ELOVL2 |
| 0.73789 | 0.001762 | SH2D3C |
| 0.726961 | 0.001767 | ZNF79 |
| 0.670337 | 0.001775 | IL11 |
| 0.664808 | 0.001785 | CCNO |
| 1.243554 | 0.00185 | IL24 |
| 0.586172 | 0.001872 | SOX7 |
| 0.670349 | 0.001872 | ZFAND2A |
| 0.754308 | 0.001932 | SLAMF7 |
| 0.64313 | 0.001971 | TYW5 |
| 0.727117 | 0.001998 | NPAT |
| 0.771144 | 0.002085 | PVRL4 |
| 0.681113 | 0.002092 | EBI3 |
| 0.641042 | 0.002125 | HMOX1 |
| 0.622919 | 0.002177 | ERCC6L2 |
| 0.59341 | 0.002243 | CNNM4 |
| 0.697723 | 0.002273 | C12orf39 |
| 0.627417 | 0.002448 | HLF |
| 0.81039 | 0.002465 | TNFSF13B |
| 0.672837 | 0.002479 | SYBU |
| 0.651267 | 0.002526 | SHISA9 |
| 0.632338 | 0.002541 | PSG4 |
| 0.653047 | 0.002606 | FZD8 |
| 0.646577 | 0.002612 | TRAPPC13 |
| 0.868338 | 0.002647 | BTC |
| 0.626248 | 0.002807 | S100A2 |
| 0.587809 | 0.002975 | IRF7 |
| 0.865682 | 0.003033 | TNFSF10 |
| 0.616748 | 0.003123 | APOL3 |
| 0.632302 | 0.003314 | FLJ00104 |
| 0.613663 | 0.003409 | SLC22A4 |
| 0.733681 | 0.003731 | EGR2 |
| 0.625408 | 0.003957 | SALL1 |
| 0.747023 | 0.004252 | YWHAH |
| 0.758377 | 0.004307 | ARRDC4 |
| 0.622949 | 0.004447 | ASAH2B |
| 0.681525 | 0.004485 | SAMD9L |
| 0.597091 | 0.004496 | C4orf21 |
| 0.586352 | 0.004529 | CES2 |
| 0.612033 | 0.004861 | ALG10 |
| 0.677259 | 0.004872 | OR51B6 |
| 0.720169 | 0.005122 | CCL5 |
| 0.623175 | 0.006018 | PI3 |
| 0.638332 | 0.006294 | LOC728537 |
| 0.765331 | 0.007063 | HS3ST3B1 |
| 0.616169 | 0.00755 | LOC100506538 /// NDUFAF6 |
| 0.990438 | 0.007724 | CCL4 |
| 0.616826 | 0.008004 | SOX9 |
| 0.599626 | 0.010288 | MYO6 |
| 0.729036 | 0.012419 | OTTHUMG00000066821 /// RP11-87H9.3 |
| 0.617516 | 0.013876 | MX2 |
| 0.793366 | 0.014535 | CCDC96 |
| 0.633094 | 0.014667 | CHGB |
| 0.607583 | 0.014729 | GMPR |
| 0.585467 | 0.016799 | ZNF256 |
| 0.601668 | 0.01903 | GAS5 |
| 0.719492 | 0.02162 | C5 |
| 1.106323 | 0.029024 | C8orf4 |
