# Supplementary material for: p53 modeling as a route to mesothelioma patients stratification and novel therapeutic identification
Source: J Transl Med. 2018 Oct 13;16:282. doi: 10.1186/s12967-018-1650-0 (PMC6186085; doi:10.1186/s12967-018-1650-0)
Supplement: Supplementary file 3 — Additional file 3: Table S3. ETO vs ctrl downregulated genes. [file 12967_2018_1650_MOESM3_ESM.docx]

**Table S3:** Etop vs ctrl downregulated genes

| **logFC** | **P.Value** | **gene_symbol** |
| --- | --- | --- |
| -2.46418 | 1.75E-08 | ID2 /// ID2B |
| -1.63636 | 1.51E-07 | SYT17 |
| -1.71637 | 2.02E-07 | KANK4 |
| -1.49697 | 2.87E-07 | CCDC85C |
| -1.64841 | 3.21E-07 | CTB-174D11.3 /// OTTHUMG00000163878 |
| -1.61135 | 3.59E-07 | VIT |
| -1.67805 | 3.86E-07 | MGC24103 |
| -1.67478 | 3.99E-07 | LRP2 |
| -1.45589 | 6.39E-07 | FAM13C |
| -1.6618 | 6.68E-07 | SERPINB9 |
| -1.40813 | 7.16E-07 | FGF18 |
| -1.63605 | 7.35E-07 | HIST1H3H |
| -1.85102 | 7.81E-07 | PSRC1 |
| -1.62809 | 8.76E-07 | GAL3ST1 |
| -1.32328 | 8.97E-07 | ST6GAL2 |
| -1.40729 | 9.04E-07 | DAPK1 |
| -2.2026 | 9.37E-07 | KIF20A |
| -1.29743 | 9.98E-07 | CYP26A1 |
| -1.32232 | 1.15E-06 | RNASE4 |
| -1.30206 | 1.20E-06 | MLLT4-AS1 |
| -1.83244 | 1.21E-06 | BNC2 |
| -1.49753 | 1.22E-06 | PBX1 |
| -1.89785 | 1.23E-06 | OTTHUMG00000032910 /// RP11-157P1.4 |
| -1.21525 | 1.24E-06 | NFATC4 |
| -1.30788 | 1.29E-06 | ATF7IP2 /// LOC100287628 |
| -1.40636 | 1.30E-06 | SEMA5A |
| -1.18705 | 1.39E-06 | MYL9 |
| -1.45057 | 1.47E-06 | DLGAP5 |
| -1.37723 | 1.53E-06 | PIF1 |
| -1.25005 | 1.53E-06 | CDH3 |
| -1.54109 | 1.58E-06 | ID2 |
| -1.23822 | 1.62E-06 | NLGN1 |
| -1.29899 | 1.66E-06 | CTD-2292M16.8 /// OTTHUMG00000178843 |
| -1.50579 | 1.74E-06 | KRT15 |
| -1.26083 | 1.92E-06 | ST6GALNAC3 |
| -1.13405 | 1.95E-06 | ZSCAN31 |
| -1.16832 | 2.01E-06 | LOC150622 |
| -1.55322 | 2.04E-06 | CDH18 |
| -1.46056 | 2.05E-06 | SMTNL2 |
| -1.22186 | 2.17E-06 | AGTR1 |
| -1.50623 | 2.28E-06 | DAPK1-IT1 |
| -1.47244 | 2.36E-06 | SEMA6D |
| -1.30417 | 2.37E-06 | SLIT3 |
| -1.28075 | 2.44E-06 | FAT4 |
| -1.08709 | 2.49E-06 | NANOG |
| -1.12888 | 2.49E-06 | FRMPD4 |
| -1.16851 | 2.49E-06 | FAM134B |
| -1.1988 | 2.50E-06 | GLI2 |
| -1.1361 | 2.57E-06 | BTN3A3 |
| -1.15018 | 2.65E-06 | PDZD2 |
| -1.12372 | 2.86E-06 | LAMA5 |
| -1.26279 | 2.86E-06 | ERVFRD-1 |
| -1.35235 | 2.89E-06 | PODXL |
| -1.32488 | 2.93E-06 | OLFML1 |
| -1.18628 | 2.98E-06 | LINC00173 |
| -1.06553 | 3.02E-06 | TYRP1 |
| -1.066 | 3.07E-06 | FOXO6 /// FOXO6 |
| -1.25713 | 3.20E-06 | LOC150622 /// LOC400940 |
| -1.38616 | 3.23E-06 | LPHN3 |
| -1.19938 | 3.26E-06 | RNF150 |
| -1.33332 | 3.26E-06 | CDH1 |
| -1.20887 | 3.27E-06 | COL11A1 |
| -1.30734 | 3.27E-06 | LOC100507311 |
| -1.1124 | 3.39E-06 | LOC100507303 |
| -1.10736 | 3.48E-06 | VASN |
| -1.07549 | 3.87E-06 | PGK1 |
| -1.05009 | 3.90E-06 | LRRC7 |
| -1.06597 | 3.92E-06 | IGSF9 |
| -1.5675 | 3.93E-06 | HIST1H2BC |
| -1.16511 | 3.95E-06 | GYS1 |
| -1.23913 | 3.99E-06 | ZDHHC8P1 |
| -1.25125 | 4.12E-06 | CENPA /// SLC35F6 |
| -1.14697 | 4.20E-06 | NUDT13 |
| -1.24795 | 4.24E-06 | LOC100996341 |
| -1.08403 | 4.30E-06 | LOC339535 |
| -1.04109 | 4.39E-06 | MLLT3 |
| -1.1122 | 4.41E-06 | PTTG1 |
| -1.19311 | 4.42E-06 | ITIH5 |
| -1.31054 | 4.44E-06 | TOB1-AS1 |
| -1.16269 | 4.53E-06 | TMTC2 |
| -0.99019 | 4.60E-06 | GSE1 |
| -1.15562 | 4.61E-06 | FAT3 |
| -1.09627 | 4.71E-06 | TPM1 |
| -1.03129 | 4.82E-06 | EBPL |
| -1.07816 | 4.83E-06 | CAHM |
| -1.01875 | 4.89E-06 | SUN2 |
| -0.99651 | 4.92E-06 | ZBED5-AS1 |
| -1.01711 | 4.94E-06 | RPL31 |
| -1.05169 | 5.01E-06 | PCDHB5 |
| -0.98041 | 5.29E-06 | EFEMP1 |
| -1.01418 | 5.63E-06 | MEIS2 |
| -0.97958 | 5.72E-06 | SLC16A4 |
| -1.04185 | 5.85E-06 | ATP7B |
| -1.04371 | 5.89E-06 | C1orf213 |
| -0.96053 | 6.21E-06 | PTPRF |
| -1.00473 | 6.25E-06 | ANKRD29 |
| -0.98017 | 6.77E-06 | FBXO16 /// ZNF395 |
| -0.93098 | 6.98E-06 | SLC44A1 |
| -1.20313 | 6.99E-06 | ARHGEF26 |
| -1.26394 | 7.14E-06 | AGAP1 |
| -0.94934 | 7.19E-06 | FLJ11235 |
| -0.96147 | 7.70E-06 | CRYZ |
| -1.06071 | 7.72E-06 | RAB33A |
| -0.92271 | 7.79E-06 | SLITRK5 |
| -1.05516 | 7.81E-06 | SPA17 |
| -1.48217 | 7.84E-06 | MIR100HG |
| -0.95979 | 8.07E-06 | CEP70 |
| -1.33546 | 8.12E-06 | CNTN3 |
| -1.13733 | 8.25E-06 | ROBO1 |
| -0.93988 | 8.26E-06 | KCNK5 |
| -1.12966 | 8.39E-06 | EDIL3 |
| -1.38845 | 8.59E-06 | TNFAIP8L1 |
| -0.9432 | 8.62E-06 | ERGIC1 |
| -1.01093 | 8.75E-06 | DDIT4 |
| -0.95126 | 8.83E-06 | BTN3A2 |
| -1.02879 | 8.88E-06 | ARRB1 |
| -0.99778 | 9.19E-06 | OTTHUMG00000176821 /// RP11-846E15.4 |
| -1.06137 | 9.38E-06 | GRB14 |
| -1.03049 | 9.46E-06 | LOC151009 /// LOC440894 |
| -0.90645 | 9.50E-06 | PLCXD1 |
| -0.91873 | 9.56E-06 | COX16 /// SYNJ2BP-COX16 |
| -1.07568 | 9.58E-06 | MIRLET7BHG |
| -1.44121 | 9.68E-06 | INHBB |
| -0.90548 | 9.75E-06 | PAK4 |
| -0.89143 | 9.85E-06 | ELFN1 |
| -0.93259 | 9.99E-06 | PTGIS |
| -0.90325 | 1.01E-05 | TMED4 |
| -0.9775 | 1.11E-05 | PSD3 |
| -0.93271 | 1.14E-05 | ENOX1 |
| -0.92081 | 1.14E-05 | DFFB |
| -0.98834 | 1.14E-05 | BTN3A2 /// BTN3A3 |
| -1.25053 | 1.16E-05 | BAIAP2-AS1 |
| -0.86145 | 1.17E-05 | CXorf24 |
| -1.08688 | 1.18E-05 | OTTHUMG00000167487 /// RP11-178H8.7 |
| -1.03954 | 1.22E-05 | C19orf33 |
| -0.86968 | 1.22E-05 | OTTHUMG00000162476 /// RP11-974F13.6 |
| -0.90907 | 1.23E-05 | MTMR4 |
| -0.87985 | 1.28E-05 | FBXL7 |
| -0.84693 | 1.32E-05 | ACADSB |
| -1.04118 | 1.34E-05 | GLS |
| -1.44884 | 1.34E-05 | SATB1 |
| -0.94358 | 1.38E-05 | GPI |
| -0.89122 | 1.39E-05 | GULP1 |
| -0.86827 | 1.41E-05 | OSBPL1A |
| -1.0374 | 1.45E-05 | TBC1D5 |
| -0.91781 | 1.45E-05 | OXTR |
| -1.37881 | 1.45E-05 | FSTL1 |
| -1.38277 | 1.49E-05 | H19 /// MIR675 |
| -0.94059 | 1.50E-05 | ROR1 |
| -1.09188 | 1.51E-05 | AURKA |
| -0.90335 | 1.51E-05 | GLIS2 |
| -0.8607 | 1.53E-05 | CARNS1 |
| -0.83183 | 1.53E-05 | FA2H |
| -0.83315 | 1.57E-05 | ZFX |
| -0.89119 | 1.60E-05 | BCAR3 |
| -0.86449 | 1.61E-05 | LRRC1 |
| -0.98468 | 1.66E-05 | FBXL2 |
| -0.96739 | 1.68E-05 | CRB2 |
| -0.83115 | 1.72E-05 | FAM172A |
| -0.9215 | 1.73E-05 | TSPAN13 |
| -0.97072 | 1.74E-05 | FAM49B |
| -0.81674 | 1.74E-05 | PTPN14 |
| -0.8735 | 1.74E-05 | DTX4 |
| -0.96953 | 1.76E-05 | LIMCH1 |
| -0.90466 | 1.79E-05 | SLC39A10 |
| -0.94057 | 1.79E-05 | SEPT9 |
| -0.93476 | 1.82E-05 | LOC389834 /// MAFIP /// TEKT4P2 |
| -0.87618 | 1.82E-05 | MIR10A |
| -0.83759 | 1.82E-05 | PTTG3P |
| -0.83495 | 1.86E-05 | CRELD1 |
| -0.8213 | 1.86E-05 | SLC40A1 |
| -0.87194 | 1.89E-05 | MN1 |
| -0.82642 | 1.93E-05 | PGM1 |
| -1.00924 | 1.97E-05 | CLDN15 |
| -1.12206 | 2.05E-05 | CRLS1 |
| -0.9382 | 2.05E-05 | C7orf60 |
| -0.82364 | 2.09E-05 | LRRN4 |
| -0.79001 | 2.09E-05 | FIG4 |
| -1.10019 | 2.10E-05 | BCR |
| -0.84133 | 2.12E-05 | BCAR1 |
| -0.8212 | 2.12E-05 | STON1 |
| -0.93151 | 2.17E-05 | PLLP |
| -0.91572 | 2.18E-05 | ANKH |
| -1.40718 | 2.19E-05 | NCKAP5 |
| -0.82186 | 2.19E-05 | RFX7 |
| -0.85506 | 2.24E-05 | NME3 |
| -0.83156 | 2.24E-05 | C10orf114 |
| -0.95817 | 2.25E-05 | ZMYND8 |
| -0.80428 | 2.26E-05 | NIPSNAP1 |
| -0.95353 | 2.28E-05 | OTTHUMG00000170856 /// RP11-33E12.2 |
| -0.8084 | 2.29E-05 | MSI2 |
| -0.92684 | 2.35E-05 | UNC5B-AS1 |
| -0.7772 | 2.36E-05 | AKR1C3 |
| -0.96656 | 2.40E-05 | ZNF804A |
| -0.82129 | 2.43E-05 | TMEM256 |
| -0.83286 | 2.46E-05 | LRRFIP1 |
| -0.83631 | 2.46E-05 | PRKAG2 |
| -0.88936 | 2.47E-05 | PLEKHA2 |
| -0.88285 | 2.48E-05 | ANXA8 /// LOC100996760 /// LOC101060462 |
| -1.10706 | 2.50E-05 | DOK7 |
| -0.99513 | 2.51E-05 | UPK3B |
| -0.99749 | 2.59E-05 | OTTHUMG00000175814 /// RP11-13L2.4 |
| -0.79488 | 2.59E-05 | LOC100287896 |
| -0.76567 | 2.60E-05 | ZNF618 |
| -0.90245 | 2.61E-05 | RPS15A |
| -0.92157 | 2.63E-05 | TMEM191A |
| -0.87904 | 2.68E-05 | CCNB1 |
| -0.8013 | 2.71E-05 | ABCA3 |
| -0.77161 | 2.72E-05 | LDOC1 |
| -0.79133 | 2.72E-05 | CCNG2 |
| -0.82079 | 2.74E-05 | FAM83D |
| -0.89275 | 2.76E-05 | YPEL2 |
| -0.82598 | 2.78E-05 | AK9 |
| -1.12955 | 2.79E-05 | PROC |
| -0.76056 | 2.86E-05 | MYH9 |
| -0.8861 | 2.88E-05 | MUS81 |
| -0.77516 | 2.92E-05 | S100A10 |
| -0.87559 | 2.92E-05 | S100A4 |
| -0.77147 | 3.05E-05 | FAM168A |
| -1.31407 | 3.07E-05 | C4orf3 |
| -1.19214 | 3.18E-05 | EXT1 |
| -1.06776 | 3.24E-05 | SHB |
| -0.84097 | 3.24E-05 | NRDE2 |
| -1.07439 | 3.25E-05 | CNTN5 |
| -0.879 | 3.25E-05 | CBX7 |
| -0.75713 | 3.29E-05 | PDGFC |
| -0.75283 | 3.43E-05 | SASH1 |
| -0.89306 | 3.46E-05 | OTTHUMG00000167230 /// RP11-736K20.4 |
| -0.76623 | 3.51E-05 | ST3GAL1 |
| -0.74796 | 3.52E-05 | CASP6 |
| -0.76479 | 3.56E-05 | RBM38 |
| -0.74659 | 3.67E-05 | CENPE |
| -1.72682 | 3.71E-05 | GPM6A |
| -0.81495 | 3.75E-05 | LYPD6B |
| -0.7401 | 3.75E-05 | LOC692247 |
| -0.78633 | 3.76E-05 | CXXC5 |
| -1.02236 | 3.86E-05 | TENM2 |
| -0.77999 | 3.90E-05 | WDPCP |
| -0.72133 | 3.91E-05 | TBCE |
| -0.97108 | 3.92E-05 | FNTB |
| -1.33592 | 3.93E-05 | CAPN6 |
| -0.75586 | 4.01E-05 | TLDC1 |
| -1.10975 | 4.02E-05 | PPP1R3C |
| -0.78244 | 4.04E-05 | NPEPPS |
| -0.76654 | 4.07E-05 | CA11 |
| -0.72675 | 4.10E-05 | TPRN |
| -0.71167 | 4.15E-05 | NRBP2 |
| -0.74597 | 4.20E-05 | BAIAP2 |
| -0.75475 | 4.28E-05 | HEG1 |
| -0.77108 | 4.34E-05 | FZD2 |
| -0.72452 | 4.39E-05 | WASF3 |
| -0.87156 | 4.40E-05 | C1GALT1C1 |
| -0.74612 | 4.42E-05 | LOC254057 |
| -0.875 | 4.49E-05 | IL7 |
| -0.71479 | 4.62E-05 | MED11 |
| -0.74633 | 4.67E-05 | CTB-31O20.2 /// OTTHUMG00000175708 |
| -0.70515 | 4.68E-05 | SEMA3F |
| -0.93743 | 4.70E-05 | SORL1 |
| -0.71427 | 4.83E-05 | JARID2 |
| -0.82285 | 4.85E-05 | SEMA3C |
| -0.70284 | 4.87E-05 | BCHE |
| -0.72148 | 4.88E-05 | LOC100505715 |
| -0.89768 | 4.90E-05 | LOC100507577 /// LONP2 |
| -0.88307 | 4.94E-05 | ITGA3 |
| -0.84846 | 5.03E-05 | TAGLN |
| -0.91476 | 5.04E-05 | MICAL2 |
| -0.69463 | 5.06E-05 | OTTHUMG00000176823 /// RP11-846E15.2 |
| -0.73847 | 5.07E-05 | PLIN3 |
| -0.72981 | 5.14E-05 | DCLK2 |
| -0.7816 | 5.19E-05 | CAV2 |
| -1.4906 | 5.22E-05 | BNIP3 |
| -0.69503 | 5.22E-05 | LOC101060235 /// TMSB15A /// TMSB15B |
| -1.02927 | 5.23E-05 | ATG4C |
| -0.70113 | 5.23E-05 | OTTHUMG00000177151 /// RP11-715J22.6 |
| -0.9675 | 5.31E-05 | FAM64A |
| -0.80008 | 5.31E-05 | TKT |
| -0.69594 | 5.32E-05 | ANXA8 /// ANXA8L1 /// ANXA8L2 |
| -0.80647 | 5.39E-05 | STOX2 |
| -1.12318 | 5.39E-05 | SSBP3 |
| -0.69757 | 5.39E-05 | ATXN10 |
| -0.87083 | 5.47E-05 | KRT8 |
| -0.69205 | 5.49E-05 | COL18A1 |
| -0.84848 | 5.54E-05 | ZNF395 |
| -0.91303 | 5.64E-05 | GPSM2 |
| -0.85388 | 5.68E-05 | ERBB4 |
| -0.93091 | 5.70E-05 | ARL15 |
| -1.00926 | 5.75E-05 | NFIA |
| -0.69765 | 5.75E-05 | E2F5 |
| -0.96566 | 5.76E-05 | NREP |
| -0.6934 | 5.81E-05 | HIST1H2BH |
| -0.70123 | 5.85E-05 | C3orf70 |
| -0.68669 | 5.86E-05 | PEX11A |
| -0.78594 | 5.99E-05 | SETBP1 |
| -0.82403 | 6.02E-05 | CTIF |
| -0.84367 | 6.08E-05 | MCC |
| -0.75483 | 6.08E-05 | MIR181A2HG |
| -0.99351 | 6.11E-05 | HIST2H2BE |
| -0.68197 | 6.11E-05 | DBP |
| -0.7407 | 6.15E-05 | TSPAN31 |
| -1.2737 | 6.23E-05 | FAM162A |
| -0.73508 | 6.25E-05 | OSER1-AS1 |
| -0.84821 | 6.26E-05 | C1orf21 |
| -0.92388 | 6.28E-05 | LINC00521 |
| -0.76446 | 6.30E-05 | YY1 |
| -0.67941 | 6.35E-05 | DOCK4 |
| -0.99689 | 6.51E-05 | CSMD3 |
| -0.80867 | 6.59E-05 | TTC28 |
| -0.68241 | 6.68E-05 | CAT |
| -1.28329 | 6.76E-05 | PLK1 |
| -0.82309 | 6.77E-05 | ALPK2 |
| -0.71326 | 6.82E-05 | LINC00685 |
| -0.75352 | 6.86E-05 | PLGLB1 /// PLGLB2 |
| -0.70639 | 6.88E-05 | ST3GAL5 |
| -0.72431 | 6.90E-05 | GRAMD4 |
| -0.6702 | 6.93E-05 | PEX2 |
| -0.76558 | 6.95E-05 | RSBN1 |
| -0.69615 | 7.00E-05 | LOC100507054 |
| -0.6958 | 7.05E-05 | FSTL3 |
| -0.75051 | 7.11E-05 | ATP2B4 |
| -0.73685 | 7.12E-05 | SELENBP1 |
| -0.72257 | 7.12E-05 | IPW /// LOC100506948 /// SNORD107 /// SNORD115-13 /// SNORD115-26 /// SNORD115-7 /// SNORD116-28 /// SNRPN |
| -0.72976 | 7.15E-05 | SERPINB1 |
| -0.7805 | 7.27E-05 | ISOC2 |
| -0.71658 | 7.28E-05 | COL4A6 |
| -1.31188 | 7.29E-05 | B3GALT2 |
| -1.33272 | 7.35E-05 | FKBP7 |
| -1.06125 | 7.38E-05 | LOC151009 |
| -1.02518 | 7.59E-05 | CLIC3 |
| -0.80836 | 7.64E-05 | ST6GALNAC5 |
| -0.80485 | 7.65E-05 | KIF14 |
| -1.45504 | 7.69E-05 | MIR210HG |
| -0.66943 | 7.72E-05 | PKM |
| -0.64772 | 7.79E-05 | GAS1 |
| -0.66396 | 7.82E-05 | ANXA4 |
| -0.82484 | 7.82E-05 | TPD52L1 |
| -0.74188 | 7.83E-05 | TOX2 |
| -0.68679 | 7.93E-05 | HSD17B2 |
| -0.70714 | 7.97E-05 | OTTHUMG00000019884 /// RP11-112J3.16 |
| -0.88796 | 8.00E-05 | CA9 |
| -0.66515 | 8.07E-05 | ANKRD13C |
| -0.78947 | 8.12E-05 | FAM19A5 |
| -0.86822 | 8.13E-05 | LOC100507486 |
| -0.65041 | 8.13E-05 | CTD-2336O2.1 /// OTTHUMG00000163625 |
| -0.71042 | 8.21E-05 | TTC30B |
| -0.6465 | 8.29E-05 | GMDS |
| -0.90678 | 8.34E-05 | ALDH6A1 |
| -0.69987 | 8.36E-05 | TPD52 |
| -0.81257 | 8.43E-05 | MIR4800 /// MXD4 |
| -0.63852 | 8.45E-05 | NACC2 |
| -0.90637 | 8.49E-05 | TET1 |
| -0.78296 | 8.51E-05 | PHACTR1 |
| -0.66878 | 8.51E-05 | LOC100507316 |
| -1.41384 | 8.52E-05 | RNFT2 |
| -0.64471 | 8.57E-05 | SPESP1 |
| -0.65629 | 8.58E-05 | ITPR2 |
| -1.03943 | 8.63E-05 | CENPA |
| -0.76 | 8.74E-05 | PTPN13 |
| -1.12446 | 8.86E-05 | TTC30A |
| -0.92713 | 8.89E-05 | PPL |
| -1.00412 | 8.93E-05 | LOC100506990 |
| -0.70263 | 8.99E-05 | LOC101060527 /// NAIP |
| -0.66225 | 9.08E-05 | NPNT |
| -0.67876 | 9.27E-05 | WT1 |
| -0.6792 | 9.32E-05 | SMAD3 |
| -0.7151 | 9.37E-05 | DHRS3 |
| -0.95079 | 9.61E-05 | LAMB1 |
| -0.6835 | 9.62E-05 | ARHGAP18 |
| -1.09311 | 9.64E-05 | HIST1H2AC |
| -0.73028 | 9.67E-05 | DIP2C |
| -0.66666 | 9.74E-05 | HCN1 |
| -1.02485 | 9.81E-05 | OTTHUMG00000180314 /// RP1-193H18.2 |
| -0.68016 | 9.86E-05 | TMEM161B-AS1 |
| -1.43074 | 9.91E-05 | KIAA1984 |
| -0.85412 | 9.95E-05 | SULF1 |
| -0.6595 | 9.96E-05 | THRA |
| -0.88992 | 0.0001 | THNSL1 |
| -0.77954 | 0.0001 | COL5A1 |
| -0.66996 | 0.0001 | ACTN4 |
| -0.63052 | 0.000101 | CHRNB1 |
| -0.62129 | 0.000101 | SLCO3A1 |
| -0.70473 | 0.000101 | MYOF |
| -0.62492 | 0.000102 | ADAMTS3 |
| -1.26665 | 0.000103 | DUSP5P1 |
| -0.78361 | 0.000103 | SLC7A7 |
| -0.64262 | 0.000106 | HCFC1R1 |
| -0.71699 | 0.000108 | USP48 |
| -0.67147 | 0.000108 | GALNS |
| -0.62819 | 0.000108 | CRIP2 |
| -0.63789 | 0.000109 | BMPR1A |
| -0.6596 | 0.000109 | KIAA1522 |
| -0.9313 | 0.000109 | GBE1 |
| -0.67123 | 0.00011 | PDPN |
| -1.15378 | 0.000111 | FOS |
| -0.64614 | 0.000112 | LMBRD1 |
| -1.05618 | 0.000113 | BCL11A |
| -0.68887 | 0.000114 | PLOD1 |
| -0.69783 | 0.000114 | CFH /// CFHR1 |
| -0.72308 | 0.000115 | ZBTB20 |
| -0.90558 | 0.000115 | WT1-AS |
| -0.7401 | 0.000115 | CTSH |
| -0.67007 | 0.000116 | CDC20 |
| -0.67135 | 0.000116 | KBTBD7 |
| -0.99067 | 0.000117 | C10orf54 |
| -0.70679 | 0.000119 | MRPS28 |
| -0.64874 | 0.000119 | PARD3 |
| -0.65069 | 0.000124 | GAS6 |
| -0.81022 | 0.000125 | RAB11FIP4 |
| -0.63857 | 0.000126 | MORF4L2-AS1 |
| -0.62018 | 0.000128 | AMOTL2 |
| -0.63172 | 0.000128 | MEGF6 |
| -0.7423 | 0.000128 | TMCC1 |
| -0.60433 | 0.000128 | MEX3A |
| -0.6089 | 0.000128 | SPANXB1 /// SPANXB2 /// SPANXF1 |
| -0.60108 | 0.000128 | FUT8 |
| -0.61468 | 0.000129 | ZAK |
| -0.96991 | 0.00013 | HIST1H2BD |
| -0.70837 | 0.000131 | SYTL5 |
| -0.65498 | 0.000131 | C11orf54 |
| -0.65409 | 0.000131 | NXN |
| -0.74337 | 0.000131 | FYN |
| -0.61037 | 0.000132 | LOC100506965 |
| -0.63045 | 0.000133 | PTRHD1 |
| -0.61092 | 0.000133 | SALL2 |
| -0.60896 | 0.000133 | COL4A2 |
| -0.63467 | 0.000136 | CPQ |
| -0.73515 | 0.000137 | PLXNB2 |
| -0.59781 | 0.000138 | C15orf52 |
| -0.88758 | 0.000139 | OTTHUMG00000179824 /// RP11-173M1.8 |
| -0.61525 | 0.000141 | C6orf120 |
| -0.72115 | 0.000141 | CDKN1C |
| -1.00795 | 0.000142 | SAPCD2 |
| -0.58735 | 0.000142 | IL10RB |
| -0.64987 | 0.000143 | CDK19 |
| -0.6105 | 0.000144 | FAM196A |
| -0.58754 | 0.000144 | PTPRG |
| -0.83412 | 0.000145 | GSTA4 |
| -0.59907 | 0.000145 | LOC101060440 /// LOC101060471 /// LOC101060522 /// LOC440434 /// NPEPPS /// TBC1D3 |
| -0.64419 | 0.000146 | IGFBP2 |
| -0.62369 | 0.000146 | EHD1 |
| -0.72175 | 0.000146 | FAM224A /// FAM224B |
| -0.60456 | 0.000147 | FLNA |
| -0.80727 | 0.000147 | KCND2 |
| -0.60549 | 0.000147 | FLNC |
| -0.6038 | 0.000149 | B9D2 |
| -0.62231 | 0.000153 | KRT19 |
| -0.8809 | 0.000153 | PDK1 |
| -0.97253 | 0.000154 | FPGT |
| -0.6712 | 0.000155 | EPM2AIP1 |
| -0.62267 | 0.000157 | GUSBP3 /// GUSBP9 /// LOC100653061 /// LOC101060519 |
| -0.64861 | 0.000158 | LIX1L /// LOC101060547 |
| -0.77513 | 0.000158 | BTN3A1 |
| -0.62144 | 0.000159 | LOC389834 |
| -0.59231 | 0.000161 | PPP3CA |
| -0.60872 | 0.000161 | PPP2R4 |
| -0.59179 | 0.000161 | LINC00493 |
| -0.62728 | 0.000161 | HK1 |
| -0.95731 | 0.000162 | CCDC80 |
| -0.60356 | 0.000162 | CNOT8 |
| -0.60134 | 0.000163 | STIM2 |
| -0.62462 | 0.000163 | RHOU |
| -0.74055 | 0.000164 | DEPDC1 |
| -0.69754 | 0.000166 | SNUPN |
| -0.64997 | 0.000166 | HNRNPU-AS1 |
| -1.01382 | 0.000167 | PYGM |
| -0.89077 | 0.000168 | RAB17 |
| -0.63908 | 0.000168 | LSM14A |
| -0.95982 | 0.000169 | OTTHUMG00000175832 /// RP11-274H2.5 |
| -0.733 | 0.000171 | CTC-429P9.3 /// OTTHUMG00000182633 |
| -0.92475 | 0.000172 | EFNA5 |
| -0.80622 | 0.000172 | IDH1 |
| -0.63625 | 0.000173 | SPNS2 |
| -0.9397 | 0.000173 | PVALB |
| -0.87148 | 0.000174 | RPL31 /// TBC1D8 |
| -0.69498 | 0.000174 | NDRG3 |
| -0.62506 | 0.000174 | TDRD3 |
| -0.74557 | 0.000175 | PLXDC2 |
| -0.79757 | 0.000175 | SERTM1 |
| -0.69082 | 0.000176 | ZNF25 |
| -0.60286 | 0.000179 | ARHGAP17 |
| -0.60717 | 0.000179 | CHMP2A |
| -0.85823 | 0.00018 | LINC00086 /// LINC00087 |
| -0.61143 | 0.00018 | FAM8A1 |
| -0.65871 | 0.000182 | FBXL19 |
| -0.69254 | 0.000182 | PIK3C3 |
| -0.58512 | 0.000183 | GRIP1 |
| -0.67619 | 0.000184 | TNIK |
| -0.63581 | 0.000184 | USP3 |
| -0.81304 | 0.000184 | BMP4 |
| -0.63592 | 0.000185 | THAP2 |
| -0.70419 | 0.000186 | FRMD4A |
| -0.97479 | 0.000188 | PDGFRB |
| -0.68125 | 0.000188 | WNT2B |
| -0.66588 | 0.000189 | ARL6IP5 |
| -0.72015 | 0.000189 | NDRG4 |
| -0.75583 | 0.00019 | SGOL2 |
| -0.63068 | 0.000191 | CYFIP2 |
| -0.80318 | 0.000191 | LOC100128822 |
| -0.63542 | 0.000191 | PAPOLA |
| -0.59355 | 0.000192 | SOX12 |
| -0.7212 | 0.000193 | TLE2 |
| -0.61471 | 0.000193 | EPB41L4A |
| -0.6387 | 0.000196 | BOK |
| -0.60515 | 0.000197 | ARHGEF17 |
| -0.75916 | 0.000197 | PRR5 |
| -0.71176 | 0.000198 | KCNT2 |
| -0.76395 | 0.000198 | TGFBR3 |
| -0.70302 | 0.000201 | DNM2 |
| -0.58748 | 0.000205 | FAM127A |
| -0.60398 | 0.000205 | OTTHUMG00000172405 /// RP11-752G15.7 |
| -0.67516 | 0.000205 | GAPDHP73 /// GAPDHP73 |
| -0.65847 | 0.000207 | H2BFS |
| -1.06021 | 0.000207 | C12orf76 |
| -0.62521 | 0.000207 | SLC25A23 |
| -0.98289 | 0.000208 | MXI1 |
| -0.64247 | 0.000208 | FBXO46 |
| -0.61952 | 0.000209 | EXD2 |
| -0.58596 | 0.000209 | NICN1 |
| -0.61339 | 0.000215 | ANKRD23 |
| -0.85057 | 0.000215 | SBSPON |
| -0.60952 | 0.000216 | CXADR |
| -0.65359 | 0.000219 | ASB9 |
| -0.59619 | 0.000222 | BNC1 |
| -0.68982 | 0.000224 | PDGFD |
| -0.82948 | 0.000225 | CDCA3 |
| -0.5908 | 0.000225 | PPP1R14B |
| -0.59496 | 0.000226 | SEMA4B |
| -0.69041 | 0.000227 | NR2F2 |
| -0.89138 | 0.000227 | LOC100507165 |
| -0.64071 | 0.000228 | ATRNL1 |
| -0.70237 | 0.00023 | PABPC5 |
| -0.6142 | 0.000233 | KLHL31 |
| -0.90457 | 0.000235 | CTA-29F11.1 /// OTTHUMG00000172744 |
| -0.62639 | 0.000235 | TMEM98 |
| -0.67861 | 0.000236 | LOC401397 |
| -0.63395 | 0.000237 | PDHB |
| -0.71901 | 0.000238 | CRIM1 |
| -0.59392 | 0.00024 | CDR2L |
| -0.83033 | 0.00024 | PTPRK |
| -0.65225 | 0.000242 | PRSS16 |
| -0.79341 | 0.000242 | KDM4C |
| -0.83745 | 0.000243 | SOX11 |
| -0.70858 | 0.000243 | CCNF |
| -0.60658 | 0.000244 | OTTHUMG00000177465 /// RP11-353N14.2 |
| -1.19908 | 0.000244 | GJA1 |
| -0.67943 | 0.000248 | AC092620.2 /// OTTHUMG00000153633 |
| -0.85612 | 0.000248 | TMEM132B |
| -0.68897 | 0.000248 | POTEKP |
| -0.63134 | 0.000256 | CFH |
| -0.92117 | 0.000258 | LOC100506934 |
| -0.82747 | 0.00026 | OTTHUMG00000162817 /// RP11-549J18.1 |
| -0.62069 | 0.000262 | PRKCB |
| -0.58997 | 0.000263 | HOTAIRM1 |
| -0.59806 | 0.000264 | C11orf70 |
| -0.5928 | 0.000266 | TNFRSF1A |
| -0.6523 | 0.000267 | RAD51-AS1 |
| -0.79562 | 0.000267 | CCDC50 |
| -0.58952 | 0.00027 | CADM1 |
| -0.71623 | 0.000271 | AVIL |
| -0.7084 | 0.000271 | CBLN2 |
| -0.58585 | 0.000274 | FSCN1 |
| -0.59017 | 0.000277 | ERMP1 |
| -0.6359 | 0.00028 | PIK3C2B |
| -0.61264 | 0.00028 | ITSN1 |
| -0.68515 | 0.000285 | SLC9A3R1 |
| -0.62631 | 0.000286 | ANKRD37 |
| -0.68042 | 0.000291 | LOC150381 |
| -0.66792 | 0.000291 | NBEA |
| -0.5888 | 0.000291 | PCBP2 |
| -0.7083 | 0.000293 | PRO2964 |
| -0.60855 | 0.000295 | YPEL1 |
| -0.59935 | 0.000298 | LINC00260 |
| -0.60284 | 0.000298 | TMEM2 |
| -0.59584 | 0.0003 | NTNG2 |
| -0.62944 | 0.000301 | OTTHUMG00000021298 /// RP11-268G12.1 |
| -0.61111 | 0.000303 | MGMT |
| -0.67146 | 0.000306 | APLN |
| -0.81517 | 0.000309 | OTTHUMG00000176931 /// RP11-319G9.3 |
| -1.03978 | 0.00031 | MLLT4 |
| -0.62007 | 0.00031 | ABCC1 |
| -0.61215 | 0.000312 | LRBA |
| -0.74161 | 0.000312 | LDHA |
| -0.71173 | 0.000314 | FAM189A1 |
| -0.6906 | 0.000314 | C7orf55 |
| -0.68747 | 0.000321 | CCNY |
| -0.6223 | 0.000327 | TCF12 |
| -0.59404 | 0.000333 | EBLN2 |
| -1.07124 | 0.000337 | HIST2H2AA3 /// HIST2H2AA4 |
| -0.66889 | 0.000339 | HIST1H2BC /// HIST1H2BE /// HIST1H2BF /// HIST1H2BG /// HIST1H2BI |
| -0.71319 | 0.00034 | ANKRD13B |
| -1.11118 | 0.00034 | TMEM255A |
| -0.63826 | 0.000341 | FLJ10038 |
| -0.63187 | 0.000343 | G2E3 |
| -0.67027 | 0.000346 | ANKZF1 |
| -0.61346 | 0.000363 | PRKCDBP |
| -0.67724 | 0.000368 | OTTHUMG00000180272 /// RP11-567M16.4 |
| -0.79168 | 0.000368 | AMN1 |
| -0.9211 | 0.00037 | LOC646903 |
| -0.74632 | 0.000374 | QPRT |
| -0.72947 | 0.000375 | KLHL4 |
| -0.63797 | 0.000381 | KLK10 |
| -0.60532 | 0.000382 | KIAA1432 |
| -0.62223 | 0.000383 | C2CD2 |
| -0.63999 | 0.000383 | G6PD |
| -0.65036 | 0.000385 | MPPED2 |
| -0.70861 | 0.000391 | CDKAL1 |
| -0.61668 | 0.000395 | PPP3CB |
| -0.92449 | 0.000403 | MGAT3 |
| -0.77475 | 0.000405 | LYRM9 |
| -0.689 | 0.000412 | CCDC89 |
| -0.69394 | 0.000412 | NEO1 |
| -0.81621 | 0.000427 | CRIP1 |
| -0.7558 | 0.000428 | NR2F2-AS1 |
| -0.87424 | 0.000429 | UPK1B |
| -0.75457 | 0.00043 | HIST1H2BC /// HIST1H2BE /// HIST1H2BF /// HIST1H2BG /// HIST1H2BI /// NCALD |
| -0.74127 | 0.000443 | SNRPN /// SNURF |
| -0.64453 | 0.000447 | TRIM34 /// TRIM6-TRIM34 |
| -0.67059 | 0.000452 | DANCR |
| -0.67949 | 0.000457 | C20orf112 |
| -0.6782 | 0.000457 | PLGLA /// PLGLB1 /// PLGLB2 |
| -0.67064 | 0.000458 | ACO1 |
| -0.66211 | 0.000458 | C7orf55 /// C7orf55-LUC7L2 |
| -0.74776 | 0.000471 | TPBG |
| -0.96147 | 0.000472 | VAPA |
| -0.94629 | 0.000478 | CPB1 |
| -0.93872 | 0.000482 | PFKFB3 |
| -0.67009 | 0.000492 | ANG |
| -0.72557 | 0.000495 | EHHADH |
| -0.66766 | 0.000498 | PLOD2 |
| -0.75644 | 0.000499 | MYO10 |
| -0.60943 | 0.00051 | BTD |
| -0.62664 | 0.000513 | INADL |
| -0.60738 | 0.000516 | EFNA1 |
| -0.8802 | 0.000526 | KCNMA1 |
| -0.5978 | 0.000527 | HIST1H2AD /// HIST1H3A /// HIST1H3B /// HIST1H3C /// HIST1H3D /// HIST1H3E /// HIST1H3F /// HIST1H3G /// HIST1H3H /// HIST1H3I /// HIST1H3J |
| -0.60686 | 0.000533 | PCOLCE |
| -0.7865 | 0.000534 | TNNI3K |
| -0.68837 | 0.000536 | PPFIBP2 |
| -0.64181 | 0.00055 | ZNF703 |
| -0.62266 | 0.000555 | DIAPH2 |
| -0.76045 | 0.000571 | CA8 |
| -0.8316 | 0.000588 | FRY |
| -0.94426 | 0.000596 | TNS3 |
| -0.58883 | 0.000597 | FANCF |
| -0.67844 | 0.000603 | GPC6 |
| -0.59595 | 0.000605 | DPP10 |
| -0.93794 | 0.000606 | ITGB4 |
| -0.8178 | 0.00061 | INSIG2 |
| -0.65175 | 0.000618 | C14orf132 |
| -0.65502 | 0.000635 | SSBP2 |
| -1.00954 | 0.000665 | OTTHUMG00000176930 /// RP11-319G9.2 |
| -0.74395 | 0.000669 | PRR15 |
| -0.63277 | 0.00069 | SBF2 |
| -0.69502 | 0.000704 | GPR160 |
| -0.85473 | 0.000757 | LOC646762 |
| -0.67752 | 0.000763 | ADM |
| -0.5982 | 0.000775 | NQO1 |
| -0.66581 | 0.000819 | LOC100510707 /// LOC101060287 /// LOC101060303 /// LOC101060321 /// LOC101060351 /// LOC101060367 /// LOC101060376 /// LOC101060389 /// LOC101060403 /// LOC101060421 /// LOC101060440 /// LOC101060471 /// LOC101060489 /// LOC101060506 /// LOC101060522 /// TBC1D3 /// TBC1D3C /// TBC1D3F /// TBC1D3G /// TBC1D3H |
| -0.69433 | 0.00083 | SORCS2 |
| -0.67174 | 0.000833 | LINC00115 |
| -0.59944 | 0.00085 | OTTHUMG00000176421 /// RP11-1006G14.4 |
| -0.59416 | 0.000853 | FBXO9 |
| -0.89318 | 0.000853 | OTTHUMG00000176923 /// RP11-264L1.1 |
| -0.58504 | 0.000867 | CDC25B |
| -0.60176 | 0.000892 | MDFI |
| -0.70954 | 0.000894 | OTTHUMG00000176937 /// RP11-679B19.1 |
| -0.82664 | 0.0009 | PKI55 |
| -0.77147 | 0.000929 | SLITRK1 |
| -0.60715 | 0.000959 | DCTN3 |
| -0.78932 | 0.000976 | IPW /// LOC100506948 /// SNORD107 /// SNORD115-13 /// SNORD115-26 /// SNORD115-7 /// SNORD116-28 |
| -0.66213 | 0.001021 | TRIM16L |
| -0.70574 | 0.001069 | DPP6 |
| -0.63904 | 0.00108 | OTTHUMG00000161674 /// OTTHUMG00000161676 /// RP11-164P12.4 /// RP11-164P12.5 |
| -0.66146 | 0.001097 | CAV1 |
| -0.6021 | 0.00114 | FAM102B |
| -0.98423 | 0.001152 | PCDHB16 |
| -0.77874 | 0.001175 | C1orf191 |
| -0.66224 | 0.001218 | MYO5B |
| -0.62196 | 0.001231 | SCFD2 |
| -0.59877 | 0.001232 | FSTL5 |
| -0.82041 | 0.001303 | LOC338620 |
| -0.82677 | 0.001305 | NOL3 |
| -0.83155 | 0.00131 | TMEM120A |
| -0.59069 | 0.001349 | FBXW4P1 |
| -1.11411 | 0.001359 | LINC00842 |
| -0.58984 | 0.001363 | LOC100996628 /// SHROOM3 |
| -0.68345 | 0.0014 | ADAM1A /// ADAM1A |
| -0.59285 | 0.001407 | MBD5 |
| -0.59433 | 0.001408 | AES |
| -0.86332 | 0.001435 | KBTBD3 |
| -0.63677 | 0.001476 | AGFG1 |
| -0.62919 | 0.00148 | SNX33 |
| -0.86105 | 0.001524 | OTTHUMG00000176181 /// RP11-119F7.5 |
| -0.63216 | 0.001552 | OTTHUMG00000172119 /// RP11-208K4.2 |
| -0.65882 | 0.001593 | FAM83A |
| -0.60264 | 0.001604 | XPR1 |
| -0.71137 | 0.001605 | AK4 /// LOC100507855 |
| -0.61265 | 0.001606 | GNAZ |
| -0.58756 | 0.00163 | RIMKLB |
| -0.63147 | 0.001646 | PAN3-AS1 |
| -1.41043 | 0.001659 | NRN1 |
| -0.73586 | 0.001671 | SFXN3 |
| -0.66334 | 0.001733 | PFKFB4 |
| -0.68362 | 0.00179 | GUCY1B3 |
| -0.72672 | 0.001829 | KDM5B |
| -0.64239 | 0.001843 | LBH |
| -0.59499 | 0.00191 | NAGS |
| -1.09792 | 0.001925 | NDRG1 |
| -0.65389 | 0.001941 | BNIP3L |
| -0.60854 | 0.001942 | TM9SF3 |
| -0.59378 | 0.001977 | FAM226A /// FAM226B |
| -0.59881 | 0.002066 | AFAP1 |
| -0.83196 | 0.00209 | DOK6 |
| -0.80142 | 0.002117 | GHR |
| -0.66487 | 0.00212 | OTTHUMG00000175836 /// RP11-145F16.2 |
| -0.66356 | 0.002211 | SPATA17 |
| -0.73395 | 0.002326 | LOC202025 |
| -0.60863 | 0.0025 | TMEM102 |
| -0.61917 | 0.002562 | HIST1H2AM |
| -0.60758 | 0.002712 | SLC2A14 /// SLC2A3 |
| -0.6817 | 0.002932 | PCDHB14 |
| -0.88472 | 0.002968 | SLC2A1 |
| -0.61335 | 0.002992 | CAB39L |
| -0.59816 | 0.003014 | SMYD3 |
| -0.59347 | 0.003031 | SPAG4 |
| -0.62995 | 0.00306 | ZNF174 |
| -1.38616 | 0.003126 | PPFIA4 |
| -0.62696 | 0.003226 | MYLK |
| -0.7061 | 0.003556 | HIST1H2AB /// HIST1H2AE |
| -0.59269 | 0.003727 | ENPP5 |
| -0.66604 | 0.003808 | ECHDC3 |
| -0.58668 | 0.003859 | CAPNS2 |
| -0.94223 | 0.00395 | ALDH8A1 |
| -0.66235 | 0.004215 | ARHGEF26-AS1 |
| -0.68078 | 0.004314 | LOC286272 |
| -0.60371 | 0.004695 | FUK |
| -0.59015 | 0.004779 | LOC100130987 |
| -0.68862 | 0.009509 | SERPING1 |
| -0.70245 | 0.012658 | MAF |
| -0.59827 | 0.012917 | C11orf45 |
| -0.5882 | 0.013306 | OTTHUMG00000175555 /// RP6-201G10.2 |
| -0.64289 | 0.016097 | SCG5 |
| -1.09808 | 0.016195 | STC1 |
| -0.65785 | 0.035934 | LINC00622 |
| -0.74313 | 0.037165 | GNE |
| -0.6368 | 0.042903 | ANGPTL4 |
| -0.58923 | 0.046539 | TFRC |
