# Supplementary material for: p53 modeling as a route to mesothelioma patients stratification and novel therapeutic identification
Source: J Transl Med. 2018 Oct 13;16:282. doi: 10.1186/s12967-018-1650-0 (PMC6186085; doi:10.1186/s12967-018-1650-0)
Supplement: Supplementary file 5 — Additional file 5: Table S5. Gem vs ctrl upregulated genes. [file 12967_2018_1650_MOESM5_ESM.docx]

**Table S5:** Gem vs ctrl upregulated genes

| **logFC** | **P.Value** | **gene_symbol** |
| --- | --- | --- |
| 2.800063 | 1.97E-08 | GADD45A |
| 2.47166 | 4.84E-08 | ATF3 |
| 2.660147 | 1.05E-07 | SNHG12 /// SNORA16A /// SNORA44 /// SNORA61 |
| 2.054928 | 1.37E-07 | DTL |
| 2.18023 | 1.43E-07 | DKK1 |
| 2.296702 | 1.89E-07 | UPP1 |
| 3.911391 | 2.88E-07 | ANKRD1 |
| 2.20345 | 3.92E-07 | IL8 |
| 1.77086 | 3.96E-07 | IL6 |
| 2.102899 | 4.50E-07 | CDKN1A |
| 1.927629 | 4.66E-07 | PTX3 |
| 1.96948 | 4.78E-07 | ZNF367 |
| 1.851009 | 4.88E-07 | AEN |
| 1.676783 | 5.00E-07 | E2F8 |
| 2.066169 | 5.00E-07 | DUSP5 |
| 1.694526 | 5.42E-07 | PMAIP1 |
| 1.693039 | 5.45E-07 | PPP1R15A |
| 2.106857 | 6.62E-07 | CCNE2 |
| 1.871168 | 7.45E-07 | LOC100506342 |
| 1.615393 | 8.15E-07 | C6orf48 |
| 1.548725 | 8.30E-07 | HSPA14 |
| 2.093664 | 8.40E-07 | AHR |
| 1.679833 | 8.79E-07 | CDC25A |
| 1.806201 | 9.45E-07 | TNFAIP3 |
| 1.642253 | 9.77E-07 | HRK |
| 1.515652 | 9.92E-07 | RRM2 |
| 1.563376 | 1.03E-06 | TUFT1 |
| 1.796647 | 1.27E-06 | ESCO2 |
| 1.711832 | 1.28E-06 | DDIT3 |
| 1.573083 | 1.29E-06 | CEBPG |
| 1.453542 | 1.48E-06 | RCAN1 |
| 1.405394 | 1.53E-06 | TMEM194A |
| 1.409 | 1.57E-06 | ATAD2 |
| 1.877538 | 1.58E-06 | C11orf96 |
| 1.406385 | 1.62E-06 | CDKN2AIP |
| 1.380933 | 1.65E-06 | HBEGF |
| 1.483078 | 1.65E-06 | KLF6 |
| 1.43463 | 1.85E-06 | TNFRSF12A |
| 1.762933 | 1.86E-06 | CDC6 |
| 1.886125 | 1.95E-06 | ADRB2 |
| 1.354029 | 2.01E-06 | MCM10 |
| 1.791677 | 2.02E-06 | CXCL3 |
| 1.439404 | 2.28E-06 | NCEH1 |
| 1.40545 | 2.29E-06 | ZNF670 |
| 1.380658 | 2.33E-06 | SUPV3L1 |
| 1.315098 | 2.34E-06 | TCF19 |
| 1.350852 | 2.43E-06 | CCL2 |
| 1.859502 | 2.52E-06 | TIGAR |
| 1.327093 | 2.69E-06 | TMEM40 |
| 1.290294 | 2.76E-06 | DONSON |
| 2.269401 | 2.81E-06 | CNN1 |
| 1.258207 | 2.91E-06 | SNHG17 |
| 1.48018 | 2.97E-06 | FAM111B |
| 1.732157 | 2.98E-06 | LAMP3 |
| 1.262573 | 2.99E-06 | HSD17B7P2 |
| 1.492825 | 3.14E-06 | TGFB2 |
| 1.52654 | 3.18E-06 | OTTHUMG00000172919 /// RP11-340F14.5 |
| 1.245213 | 3.23E-06 | RBBP8 |
| 1.294552 | 3.31E-06 | PSMC3IP |
| 1.336203 | 3.33E-06 | CHAC2 |
| 1.393266 | 3.35E-06 | SNHG15 /// SNORA9 |
| 1.23886 | 3.45E-06 | RND3 |
| 1.326209 | 3.52E-06 | FAM129A |
| 2.481281 | 3.56E-06 | SLC25A25 |
| 1.400512 | 3.66E-06 | FLRT2 /// LOC100506718 |
| 1.446864 | 3.75E-06 | NPPB |
| 1.422593 | 3.84E-06 | TEX30 |
| 1.230446 | 3.96E-06 | IL18 |
| 1.61522 | 4.01E-06 | TTBK2 |
| 1.285761 | 4.03E-06 | TMEM38B |
| 1.207457 | 4.09E-06 | UBE2T |
| 1.213009 | 4.16E-06 | ZNF655 |
| 1.223847 | 4.19E-06 | TIPIN |
| 1.207113 | 4.19E-06 | FAM53C |
| 1.243215 | 4.31E-06 | MCAM |
| 1.182974 | 4.55E-06 | GTPBP4 |
| 1.527579 | 4.81E-06 | PLAU |
| 1.292834 | 4.82E-06 | CTPS1 |
| 1.182868 | 4.84E-06 | MTHFD2 |
| 1.217625 | 5.08E-06 | MASTL |
| 1.156287 | 5.23E-06 | SNHG1 /// SNORD22 /// SNORD25 /// SNORD26 /// SNORD27 /// SNORD28 /// SNORD29 /// SNORD31 |
| 1.160806 | 5.48E-06 | PISD |
| 1.178871 | 5.54E-06 | GLRX2 |
| 1.145944 | 5.63E-06 | TMEM171 |
| 1.165995 | 5.65E-06 | ORC6 |
| 1.12101 | 5.88E-06 | SRP19 |
| 1.220842 | 5.92E-06 | TAF1A |
| 1.158451 | 5.95E-06 | MRPS31P5 /// THSD1 |
| 1.165527 | 5.97E-06 | C5orf34 |
| 2.042832 | 6.07E-06 | GEM |
| 1.185263 | 6.07E-06 | TMEM138 |
| 1.146144 | 6.10E-06 | MAFF |
| 1.15121 | 6.20E-06 | SESN2 |
| 1.391994 | 6.23E-06 | BEX1 |
| 1.214979 | 6.29E-06 | HAUS8 |
| 1.156053 | 6.31E-06 | RFWD3 |
| 1.167895 | 6.33E-06 | GCH1 |
| 1.509143 | 6.37E-06 | CLDN1 |
| 1.453813 | 6.87E-06 | EXO1 |
| 1.500217 | 7.27E-06 | SHISA2 |
| 1.167427 | 7.37E-06 | LONRF3 |
| 1.231337 | 7.37E-06 | MITF |
| 1.134441 | 7.75E-06 | CCDC174 |
| 1.141716 | 7.98E-06 | ABHD3 |
| 1.203643 | 8.06E-06 | IFRD1 |
| 1.167746 | 8.10E-06 | IL7R |
| 1.150144 | 8.12E-06 | SAT1 |
| 1.18762 | 8.21E-06 | SKA1 |
| 1.345024 | 8.29E-06 | MARCH1 |
| 1.271916 | 8.36E-06 | SERTAD1 |
| 1.412518 | 8.38E-06 | HRK /// LOC283454 |
| 1.12158 | 8.48E-06 | CRY1 |
| 1.075563 | 8.57E-06 | CDC45 |
| 1.096253 | 8.82E-06 | POLE2 |
| 1.062533 | 9.03E-06 | DDX21 |
| 1.499661 | 9.05E-06 | HKDC1 |
| 1.339728 | 9.30E-06 | LOC81691 |
| 1.084756 | 9.46E-06 | UPF3B |
| 1.227556 | 9.61E-06 | FBLL1 |
| 1.120469 | 9.68E-06 | TNNC1 |
| 1.17392 | 9.69E-06 | FST |
| 1.04516 | 9.77E-06 | SARNP |
| 1.180542 | 9.97E-06 | CDCA5 |
| 1.1119 | 1.01E-05 | AKIP1 /// NUAK2 |
| 1.121824 | 1.04E-05 | MYBL2 |
| 1.183659 | 1.05E-05 | BRCA2 |
| 1.074412 | 1.07E-05 | SLC31A2 |
| 1.330408 | 1.09E-05 | SRFBP1 |
| 1.171326 | 1.13E-05 | LAMA1 |
| 1.443965 | 1.14E-05 | NEURL3 |
| 1.21624 | 1.16E-05 | GBP1 |
| 1.033707 | 1.23E-05 | SLC7A11 |
| 1.104261 | 1.23E-05 | CENPM |
| 1.0696 | 1.24E-05 | DSN1 |
| 1.032811 | 1.33E-05 | FAM24B |
| 1.222417 | 1.34E-05 | FLT1 |
| 0.984362 | 1.35E-05 | PPP1R15B |
| 1.045819 | 1.36E-05 | LOC100289092 |
| 1.175005 | 1.37E-05 | RIPK2 |
| 0.977052 | 1.43E-05 | EPT1 |
| 0.979475 | 1.44E-05 | BLM |
| 1.045852 | 1.45E-05 | MOSPD1 |
| 1.02944 | 1.45E-05 | LOC645638 |
| 1.418264 | 1.47E-05 | CAMK2D |
| 1.000827 | 1.50E-05 | STIL |
| 1.359106 | 1.51E-05 | RIBC2 |
| 1.08657 | 1.56E-05 | CLGN |
| 1.884819 | 1.56E-05 | ANKRD20A5P |
| 0.965786 | 1.58E-05 | ZWILCH |
| 1.278264 | 1.58E-05 | RAD51AP1 |
| 1.542403 | 1.59E-05 | HSPBAP1 |
| 1.415149 | 1.60E-05 | BIK |
| 2.01306 | 1.60E-05 | INHBE |
| 1.03954 | 1.61E-05 | SLC3A2 |
| 1.061023 | 1.61E-05 | OSBPL6 |
| 1.188272 | 1.65E-05 | RRAD |
| 1.048664 | 1.68E-05 | TTTY15 |
| 0.970218 | 1.68E-05 | RFC3 |
| 1.106273 | 1.69E-05 | BRIP1 |
| 1.157574 | 1.69E-05 | TMEM217 |
| 1.010661 | 1.74E-05 | CYB5R2 |
| 0.965364 | 1.74E-05 | ABL2 |
| 1.041063 | 1.74E-05 | RAB9A |
| 0.993755 | 1.75E-05 | PNO1 |
| 0.981802 | 1.80E-05 | NCAPG2 |
| 0.94167 | 1.81E-05 | SWAP70 |
| 0.961942 | 1.87E-05 | SPRTN |
| 0.955085 | 1.88E-05 | UAP1 |
| 0.940474 | 1.90E-05 | CCNE1 |
| 0.960074 | 1.97E-05 | NUPR1 |
| 0.928049 | 2.00E-05 | FHL2 |
| 1.184399 | 2.01E-05 | ULBP2 |
| 0.981123 | 2.02E-05 | SNX8 |
| 0.951172 | 2.03E-05 | AZIN1 |
| 0.971886 | 2.05E-05 | FGF2 |
| 0.944832 | 2.07E-05 | POLA1 |
| 1.032279 | 2.10E-05 | CCDC68 |
| 1.523933 | 2.11E-05 | EGR1 |
| 1.214315 | 2.12E-05 | CXCL10 |
| 1.020916 | 2.13E-05 | GNL2 |
| 1.096901 | 2.13E-05 | WDR76 |
| 1.334331 | 2.18E-05 | PINX1 |
| 0.911048 | 2.22E-05 | NGRN |
| 0.961058 | 2.23E-05 | JUN |
| 0.993508 | 2.28E-05 | CXCL1 |
| 1.270415 | 2.29E-05 | CREB5 /// LOC401317 |
| 0.957729 | 2.30E-05 | FEN1 |
| 0.928095 | 2.32E-05 | DNMT1 |
| 0.929195 | 2.33E-05 | UAP1L1 |
| 0.95951 | 2.35E-05 | USP18 |
| 1.02414 | 2.36E-05 | RFC2 |
| 0.953512 | 2.38E-05 | TUBB2A |
| 1.042558 | 2.39E-05 | CD83 |
| 1.287728 | 2.45E-05 | FANCB |
| 1.078795 | 2.46E-05 | STRIP2 |
| 1.141671 | 2.50E-05 | LETM2 |
| 1.077984 | 2.50E-05 | SDC1 |
| 0.971938 | 2.51E-05 | PSAT1 |
| 0.914953 | 2.58E-05 | SLC25A16 |
| 0.920642 | 2.59E-05 | C3orf52 |
| 0.889795 | 2.61E-05 | FANCI |
| 1.023389 | 2.63E-05 | NABP1 |
| 0.939609 | 2.64E-05 | C1orf112 |
| 0.986102 | 2.65E-05 | CTH |
| 0.938334 | 2.69E-05 | RSAD2 |
| 0.927067 | 2.79E-05 | DNAJC9 |
| 0.872992 | 2.85E-05 | EZH2 |
| 0.919852 | 2.88E-05 | CTB-92J24.2 /// OTTHUMG00000183390 |
| 0.898921 | 2.88E-05 | JPH1 |
| 0.898856 | 2.90E-05 | RNASEH2A |
| 1.044444 | 2.90E-05 | ARL13B |
| 1.070596 | 2.96E-05 | RGS17 |
| 0.895521 | 2.96E-05 | RNF138 |
| 1.03708 | 2.97E-05 | CDT1 |
| 0.86648 | 2.97E-05 | CHUK |
| 0.861939 | 3.02E-05 | SBDS /// SBDSP1 |
| 0.964401 | 3.05E-05 | MDM1 |
| 0.865275 | 3.06E-05 | VRK1 |
| 0.878903 | 3.12E-05 | PDRG1 |
| 0.909801 | 3.12E-05 | TRMT6 |
| 0.934236 | 3.14E-05 | TYMS |
| 0.901232 | 3.14E-05 | CENPQ |
| 1.211217 | 3.16E-05 | CRHBP |
| 0.904536 | 3.18E-05 | ZBTB21 |
| 0.879642 | 3.23E-05 | LOC284023 |
| 0.863993 | 3.26E-05 | RAB23 |
| 0.854953 | 3.26E-05 | POLQ |
| 0.881172 | 3.30E-05 | DNA2 |
| 0.852186 | 3.37E-05 | CENPN |
| 1.004288 | 3.42E-05 | AUNIP |
| 0.857013 | 3.48E-05 | ADAT1 |
| 0.928402 | 3.49E-05 | VSTM1 |
| 0.847811 | 3.50E-05 | ARL5B |
| 1.013248 | 3.58E-05 | PRIM2 |
| 0.878026 | 3.61E-05 | ZFP36 |
| 0.893852 | 3.63E-05 | OSTM1 |
| 1.194028 | 3.64E-05 | OTTHUMG00000183927 /// RP11-248J18.2 |
| 1.086151 | 3.66E-05 | DACT1 |
| 1.067439 | 3.68E-05 | NHS |
| 0.866437 | 3.73E-05 | PFDN2 |
| 1.015316 | 3.76E-05 | ATP8B1 |
| 0.85421 | 3.77E-05 | NCAPD3 |
| 0.847158 | 3.78E-05 | PHYH |
| 0.863571 | 3.78E-05 | ZNF443 |
| 0.831225 | 3.82E-05 | RAD18 |
| 0.846939 | 3.84E-05 | PSMG1 |
| 0.844975 | 3.87E-05 | GYG1 |
| 0.840118 | 3.88E-05 | DDX20 |
| 1.066828 | 3.94E-05 | TXNL4B |
| 0.854144 | 3.95E-05 | PALB2 |
| 0.895968 | 3.95E-05 | FKBP5 |
| 0.824909 | 4.00E-05 | PIGW |
| 1.000948 | 4.01E-05 | SRBD1 |
| 1.305623 | 4.02E-05 | SNHG8 /// SNORA24 |
| 0.881474 | 4.07E-05 | NGF |
| 1.007125 | 4.14E-05 | C11orf82 |
| 1.04367 | 4.18E-05 | MOCOS |
| 0.916621 | 4.19E-05 | HIVEP2 |
| 0.845912 | 4.20E-05 | C12orf4 |
| 1.068922 | 4.29E-05 | AVPI1 |
| 0.826973 | 4.31E-05 | CMC2 |
| 0.846522 | 4.33E-05 | BAZ1A |
| 0.911386 | 4.35E-05 | MTFR2 |
| 0.873451 | 4.35E-05 | UHRF1 |
| 0.854033 | 4.36E-05 | PTGER4 |
| 0.863175 | 4.37E-05 | TK1 |
| 0.818345 | 4.38E-05 | PCNA |
| 0.827209 | 4.47E-05 | COPS3 |
| 0.924935 | 4.49E-05 | DUSP10 |
| 0.834257 | 4.52E-05 | SLC25A19 |
| 0.813141 | 4.54E-05 | RMI1 |
| 0.828854 | 4.57E-05 | NMD3 |
| 0.845996 | 4.64E-05 | E2F7 |
| 0.812129 | 4.65E-05 | MELK |
| 0.811402 | 4.65E-05 | MCM2 |
| 1.188621 | 4.71E-05 | ELOVL4 |
| 0.792257 | 4.83E-05 | CCDC59 |
| 1.294595 | 4.87E-05 | CHM |
| 0.986056 | 4.90E-05 | OTTHUMG00000178878 /// RP11-214C8.5 |
| 0.857754 | 4.94E-05 | LINC00152 /// LOC541471 |
| 0.791079 | 5.00E-05 | URB2 |
| 0.951731 | 5.07E-05 | CDR2 |
| 1.667442 | 5.08E-05 | EGR2 |
| 0.786941 | 5.10E-05 | BCAS2 |
| 0.799442 | 5.15E-05 | GOT1 |
| 1.043959 | 5.20E-05 | ISG20 |
| 0.876737 | 5.33E-05 | PNP |
| 1.040263 | 5.36E-05 | LOC377711 /// MROH1 |
| 0.868751 | 5.36E-05 | PAK1IP1 |
| 0.895072 | 5.39E-05 | LOC101060460 /// POLR3C |
| 0.861763 | 5.40E-05 | CYP20A1 |
| 1.081653 | 5.42E-05 | SPHK1 |
| 0.911459 | 5.45E-05 | CNST |
| 1.013296 | 5.48E-05 | DDX60L |
| 0.825416 | 5.55E-05 | BTG2 |
| 0.786463 | 5.56E-05 | SLC22A15 |
| 0.787074 | 5.56E-05 | USP31 |
| 0.874551 | 5.59E-05 | NRIP3 |
| 0.894299 | 5.67E-05 | NNMT |
| 0.78354 | 5.68E-05 | RIPPLY3 |
| 0.812528 | 5.70E-05 | ZNF330 |
| 0.796036 | 5.73E-05 | HSPB11 |
| 0.830372 | 5.76E-05 | RIOK3 |
| 0.836488 | 5.80E-05 | WARS |
| 0.900479 | 5.92E-05 | SARS |
| 0.803521 | 5.93E-05 | NXT1 |
| 0.993991 | 6.00E-05 | TAF13 |
| 0.87033 | 6.06E-05 | GARS |
| 0.907415 | 6.12E-05 | LIMA1 |
| 0.81373 | 6.20E-05 | MLF1IP |
| 0.769853 | 6.27E-05 | THAP9-AS1 |
| 0.77214 | 6.28E-05 | ZWINT |
| 0.785459 | 6.32E-05 | FAM111A |
| 0.800422 | 6.34E-05 | ZNF57 |
| 0.784516 | 6.45E-05 | PAG1 |
| 0.908212 | 6.58E-05 | IRF1 |
| 0.832932 | 6.63E-05 | NFKBIB |
| 0.755659 | 6.71E-05 | BRIX1 |
| 0.763071 | 6.74E-05 | MAP1LC3B |
| 0.77025 | 6.78E-05 | FBXO5 |
| 0.789055 | 6.88E-05 | GTF2F2 |
| 0.856154 | 6.90E-05 | PASK |
| 0.815149 | 6.97E-05 | RAB27A |
| 0.84441 | 7.05E-05 | RPF2 |
| 0.851293 | 7.07E-05 | CSRP2 |
| 0.753111 | 7.12E-05 | CNKSR3 |
| 0.800753 | 7.13E-05 | CCT6P1 /// CCT6P3 |
| 0.867236 | 7.17E-05 | PDP2 |
| 0.741121 | 7.22E-05 | ZFPM2 |
| 1.028435 | 7.23E-05 | EDN1 |
| 1.615478 | 7.24E-05 | MXD1 |
| 0.856811 | 7.33E-05 | DSCC1 |
| 0.997048 | 7.58E-05 | CASP1 |
| 0.750103 | 7.59E-05 | POLE |
| 0.972373 | 7.60E-05 | IL24 |
| 0.879396 | 7.61E-05 | NOC3L |
| 0.771293 | 7.61E-05 | GGCT |
| 0.756749 | 7.63E-05 | C9orf142 |
| 0.733665 | 7.69E-05 | CAND2 |
| 0.737111 | 7.70E-05 | FANCA |
| 0.787076 | 7.77E-05 | RABGGTB /// SNORD45A /// SNORD45B /// SNORD45C |
| 0.830374 | 7.80E-05 | MND1 |
| 1.103916 | 7.88E-05 | GDAP1 |
| 0.78885 | 7.92E-05 | ZNF326 |
| 0.793556 | 7.94E-05 | TOPORS |
| 0.767443 | 7.98E-05 | SLC35G1 |
| 0.840064 | 7.99E-05 | TLCD1 |
| 0.746639 | 8.16E-05 | BIRC3 |
| 0.88874 | 8.28E-05 | ORC1 |
| 0.990364 | 8.28E-05 | KLF5 |
| 0.76713 | 8.32E-05 | TPST1 |
| 0.754438 | 8.33E-05 | AARS |
| 0.797095 | 8.38E-05 | LRRC49 |
| 0.912585 | 8.52E-05 | DUSP3 |
| 1.303211 | 8.58E-05 | INHBA |
| 0.733528 | 8.59E-05 | IMPA1 |
| 0.736588 | 8.72E-05 | MIR4723 /// TMEM199 |
| 0.741366 | 8.73E-05 | GORAB |
| 0.727227 | 8.79E-05 | PCGF6 |
| 0.717115 | 8.79E-05 | PIDD1 |
| 0.848843 | 8.93E-05 | KIAA0101 |
| 0.833778 | 9.00E-05 | CHAF1B |
| 0.731927 | 9.08E-05 | GINS3 |
| 0.837144 | 9.14E-05 | MCM4 |
| 0.712654 | 9.14E-05 | RPA2 |
| 1.118962 | 9.22E-05 | CARD16 /// CASP1 |
| 0.735088 | 9.35E-05 | ASF1B |
| 0.709433 | 9.43E-05 | NVL |
| 0.808204 | 9.45E-05 | TRIB3 |
| 0.715621 | 9.49E-05 | CCDC137 |
| 0.766809 | 9.57E-05 | SPAG1 |
| 0.733416 | 9.63E-05 | CDC7 |
| 0.982075 | 9.65E-05 | CYLD |
| 0.75793 | 9.83E-05 | LOC645984 |
| 0.94129 | 9.94E-05 | FNIP2 |
| 0.708519 | 0.0001 | ASRGL1 |
| 0.771822 | 0.000101 | WHAMM |
| 0.723856 | 0.000101 | ETHE1 |
| 0.703919 | 0.000101 | CIRH1A |
| 0.77734 | 0.000101 | SUV39H2 |
| 0.714307 | 0.000102 | MED10 |
| 0.739217 | 0.000102 | SGK1 |
| 0.754482 | 0.000102 | LOC101060478 /// RNF115 |
| 1.034037 | 0.000102 | WDHD1 |
| 1.201668 | 0.000103 | SLC25A33 |
| 0.812778 | 0.000103 | NFKBIE |
| 0.759309 | 0.000104 | NEIL3 |
| 0.72563 | 0.000104 | NDUFAF2 |
| 0.71104 | 0.000104 | PRICKLE1 |
| 0.80782 | 0.000104 | CCDC86 |
| 0.696844 | 0.000105 | FANCE |
| 0.863007 | 0.000105 | CLUHP3 |
| 0.740275 | 0.000106 | ZC3H8 |
| 1.612099 | 0.000107 | CCL20 |
| 0.703862 | 0.000109 | PTP4A1 |
| 1.241361 | 0.000109 | C1orf54 |
| 0.747201 | 0.000109 | HERC5 |
| 0.824243 | 0.00011 | TUBA4A |
| 0.694745 | 0.00011 | ATF4 |
| 0.762463 | 0.000111 | MIR1304 /// SNORA1 /// SNORA18 /// SNORA32 /// SNORA40 /// SNORA8 /// SNORD5 /// TAF1D |
| 0.696522 | 0.000112 | KNTC1 |
| 0.731473 | 0.000113 | ZNF200 |
| 0.701986 | 0.000114 | ERCC4 |
| 0.877468 | 0.000114 | ZNF473 |
| 0.731984 | 0.000114 | PPCDC |
| 0.723736 | 0.000114 | TRPC4 |
| 0.715043 | 0.000116 | GMNN |
| 0.837623 | 0.000116 | FICD |
| 0.910719 | 0.000117 | ADPRM |
| 0.789496 | 0.000117 | GOLT1B |
| 0.841551 | 0.000118 | NDUFAF5 |
| 0.751228 | 0.000118 | LRRC57 |
| 0.707593 | 0.000119 | CSPG5 |
| 0.813328 | 0.000119 | KCNG3 |
| 1.095103 | 0.000121 | ZNF257 |
| 0.991452 | 0.000121 | CCDC15 |
| 0.738875 | 0.000121 | CTC-260E6.4 /// OTTHUMG00000175724 |
| 1.229389 | 0.000122 | PTPRR |
| 0.720731 | 0.000122 | PLCB4 |
| 0.693959 | 0.000123 | MCM7 |
| 0.687967 | 0.000123 | GNPDA1 |
| 0.832318 | 0.000124 | GFOD1 |
| 0.707809 | 0.000124 | SHISA9 |
| 0.687506 | 0.000124 | CIDEC |
| 0.747768 | 0.000126 | ZCCHC8 |
| 1.305474 | 0.000126 | PHLDB2 |
| 0.827179 | 0.000129 | TAF4B |
| 0.751876 | 0.00013 | FH |
| 0.860119 | 0.000131 | DIAPH3 |
| 0.728347 | 0.000132 | MSANTD3-TMEFF1 /// TMEFF1 |
| 0.672566 | 0.000132 | USP53 |
| 0.866195 | 0.000132 | ZNF280C |
| 0.814338 | 0.000132 | LPL |
| 0.813666 | 0.000133 | DPF3 |
| 0.729721 | 0.000135 | RRN3 |
| 0.699737 | 0.000135 | SMC6 |
| 0.66949 | 0.000136 | BRICD5 |
| 0.781254 | 0.000138 | RRAGC |
| 0.664873 | 0.000138 | KCNMB3 |
| 0.908271 | 0.000138 | PHIP |
| 0.704718 | 0.000139 | IER2 |
| 1.211819 | 0.000139 | CXCL11 |
| 0.715472 | 0.00014 | RELB |
| 0.699968 | 0.000141 | TUBG1 |
| 0.707664 | 0.000142 | KLHL15 |
| 0.742474 | 0.000144 | RRS1 |
| 0.668335 | 0.000146 | HAT1 |
| 0.734384 | 0.000147 | PNPT1 |
| 0.945814 | 0.000147 | SDSL |
| 0.714511 | 0.00015 | SRSF7 |
| 0.658688 | 0.00015 | BRCA1 |
| 0.711104 | 0.00015 | IDH3A |
| 1.114925 | 0.000151 | FOSL1 |
| 0.706921 | 0.000151 | ABRACL |
| 0.799796 | 0.000153 | HCFC2 |
| 0.764189 | 0.000154 | POLR3F |
| 0.691518 | 0.000155 | PHF5A |
| 0.650995 | 0.000156 | C16orf93 |
| 0.766721 | 0.000157 | NUPL1 |
| 0.652745 | 0.000157 | BARD1 |
| 0.738627 | 0.000157 | GTF2B |
| 0.791412 | 0.000158 | IL13RA2 |
| 0.665066 | 0.000158 | RRM1 |
| 0.664242 | 0.000158 | IWS1 |
| 0.838164 | 0.000159 | AOC2 |
| 0.663845 | 0.000159 | HIF1A |
| 0.663818 | 0.00016 | S1PR3 |
| 2.406619 | 0.00016 | TAC1 |
| 0.675921 | 0.00016 | SRA1 |
| 1.056784 | 0.00016 | DUSP6 |
| 0.660247 | 0.00016 | CCNB1IP1 |
| 0.715572 | 0.000161 | PRIM1 |
| 0.716125 | 0.000161 | SPIN4 |
| 0.667159 | 0.000162 | CARS |
| 0.818362 | 0.000162 | NEXN |
| 0.692029 | 0.000163 | UBR7 |
| 0.655602 | 0.000163 | KLHDC7B |
| 0.764439 | 0.000165 | SELRC1 |
| 0.736668 | 0.000167 | ZNF430 |
| 0.655752 | 0.000168 | ZNF823 |
| 0.790236 | 0.000168 | TMEM140 |
| 0.647734 | 0.000169 | CPEB1 |
| 0.727539 | 0.00017 | LHX2 |
| 1.125344 | 0.00017 | TUBB2B |
| 0.887173 | 0.000172 | IL23A |
| 0.683255 | 0.000173 | BDNF |
| 0.954195 | 0.000174 | CSRNP1 |
| 1.223094 | 0.000176 | SLC19A2 |
| 0.713106 | 0.000176 | DNAJC1 |
| 0.679177 | 0.000178 | ZNF689 |
| 0.938304 | 0.00018 | STXBP6 |
| 0.729895 | 0.00018 | SLC20A1 |
| 0.744283 | 0.000181 | RHEBL1 |
| 0.682381 | 0.000181 | EXOSC9 |
| 0.63212 | 0.000183 | NET1 |
| 1.002981 | 0.000183 | SH2D5 |
| 0.633448 | 0.000184 | TBC1D2 |
| 0.655938 | 0.000184 | NCAPG |
| 0.823914 | 0.000184 | HELLS |
| 0.672292 | 0.000184 | RAB21 |
| 0.657631 | 0.000185 | TMEM55B |
| 1.009942 | 0.000185 | DUSP2 |
| 0.728871 | 0.000186 | JUP /// KRT17 |
| 0.649208 | 0.000186 | USP36 |
| 0.695937 | 0.000187 | REV3L |
| 0.749966 | 0.000188 | FANCG |
| 0.657326 | 0.000188 | ZNF622 |
| 0.772291 | 0.000189 | CCNYL1 |
| 0.722161 | 0.000189 | CENPH |
| 0.710529 | 0.000189 | RPAP3 |
| 0.663712 | 0.000189 | MRPL33 |
| 0.669952 | 0.00019 | NAA50 |
| 0.751127 | 0.00019 | MIR22 /// MIR22HG |
| 0.848841 | 0.00019 | JUNB |
| 0.695154 | 0.00019 | RECQL |
| 0.768431 | 0.00019 | SOCS4 |
| 0.688087 | 0.000191 | SRGAP2 /// SRGAP2C /// SRGAP2D |
| 0.655717 | 0.000191 | SHMT2 |
| 1.154345 | 0.000191 | IL32 |
| 0.807727 | 0.000194 | HES6 |
| 0.686951 | 0.000196 | FAM86B1 /// FAM86B2 /// FAM86C1 /// FAM86DP /// FAM86FP |
| 1.265021 | 0.000198 | SLAMF7 |
| 0.638262 | 0.000199 | LOC285812 |
| 0.67297 | 0.000199 | ZNF280B |
| 0.715715 | 0.000201 | CELF1 |
| 0.640482 | 0.000202 | CTGF |
| 0.62176 | 0.000202 | USP39 |
| 0.65544 | 0.000205 | TDG |
| 0.6204 | 0.000207 | SPC25 |
| 0.681564 | 0.000207 | FAM50A |
| 0.654452 | 0.000208 | ADAMTS1 |
| 0.661888 | 0.000208 | MIR1292 /// NOP56 /// SNORD110 /// SNORD57 /// SNORD86 |
| 0.652911 | 0.000209 | PRR5L |
| 0.669848 | 0.000211 | LOC100506639 /// ZNF131 |
| 0.639632 | 0.000211 | MICALL1 |
| 0.644582 | 0.000213 | GEN1 |
| 0.637874 | 0.000214 | SURF2 |
| 0.652958 | 0.000215 | KDELC2 |
| 0.74954 | 0.000216 | SENP1 |
| 0.724554 | 0.000217 | PAPPA |
| 0.627104 | 0.000218 | IQCJ-SCHIP1 /// SCHIP1 |
| 0.646567 | 0.000218 | NUP155 |
| 0.632166 | 0.000218 | C12orf29 |
| 1.029976 | 0.000219 | SNX16 |
| 0.855218 | 0.000219 | PALLD |
| 0.691884 | 0.00022 | HSD17B7 |
| 0.6858 | 0.000221 | ZNF93 |
| 0.722636 | 0.000221 | TP53INP2 |
| 0.613387 | 0.000222 | TRAPPC6B |
| 0.652853 | 0.000222 | MBD4 |
| 0.761801 | 0.000222 | RAB15 |
| 0.725259 | 0.000224 | BATF3 |
| 0.709973 | 0.000224 | ZBTB43 |
| 0.875281 | 0.000224 | C12orf44 |
| 0.65726 | 0.000224 | GAS5 /// SNORD44 /// SNORD47 /// SNORD76 /// SNORD77 /// SNORD79 /// SNORD80 /// SNORD81 |
| 0.619073 | 0.000225 | HSPA9 |
| 0.641014 | 0.000228 | PHLDA1 |
| 0.625625 | 0.000228 | CENPK |
| 0.672539 | 0.000228 | ST3GAL6 |
| 0.662828 | 0.000229 | SAC3D1 |
| 0.709105 | 0.00023 | THAP10 |
| 0.651258 | 0.000232 | RNF114 |
| 0.748668 | 0.000233 | OTTHUMG00000180151 /// RP11-28F1.2 |
| 1.065914 | 0.000235 | PTPRG-AS1 |
| 0.618912 | 0.000237 | EIF2AK3 |
| 0.620004 | 0.000237 | ZNF697 |
| 0.633751 | 0.000238 | RNF25 |
| 0.898708 | 0.00024 | UBIAD1 |
| 0.652164 | 0.00024 | RBPMS2 |
| 0.617506 | 0.000241 | EIF6 |
| 0.627621 | 0.000241 | YARS |
| 0.721465 | 0.000243 | MAP1B |
| 0.601764 | 0.000244 | TIMM44 |
| 0.699432 | 0.000245 | CPSF3 |
| 0.954101 | 0.000246 | CD274 |
| 0.648327 | 0.000251 | SLC35F2 |
| 0.636853 | 0.000252 | LOC100129518 /// SOD2 |
| 0.615597 | 0.000253 | IQCB1 |
| 0.678037 | 0.000254 | PTBP2 |
| 0.643226 | 0.000254 | SDE2 |
| 0.860224 | 0.000256 | HSD17B6 |
| 0.631434 | 0.000257 | CRELD2 |
| 0.698378 | 0.000258 | SLC25A32 |
| 0.671824 | 0.000261 | PIGA |
| 0.676713 | 0.000262 | COCH |
| 0.629816 | 0.000262 | PKMYT1 |
| 0.682185 | 0.000263 | HAUS2 |
| 0.609531 | 0.000263 | FAM46B |
| 0.635461 | 0.000263 | PPRC1 |
| 0.660919 | 0.000264 | RP9 |
| 0.594865 | 0.000265 | ARL8B |
| 0.61045 | 0.000268 | DDX5 /// MIR3064 /// MIR5047 |
| 0.625011 | 0.00027 | RBM15 |
| 0.652268 | 0.000271 | ZNF267 |
| 0.619489 | 0.000272 | YTHDF3 |
| 0.629058 | 0.000272 | TMEM54 |
| 0.72375 | 0.000273 | RAB27B |
| 0.600042 | 0.000274 | NOLC1 |
| 0.60796 | 0.000274 | PSME3 |
| 0.679385 | 0.000275 | AGPAT9 |
| 0.61276 | 0.000279 | GINS1 |
| 0.743799 | 0.000279 | IFI30 /// PIK3R2 |
| 0.613348 | 0.00028 | MSANTD3 |
| 0.635632 | 0.000282 | LRR1 |
| 0.644746 | 0.000284 | FBF1 |
| 0.906318 | 0.000284 | PTHLH |
| 0.655783 | 0.000286 | POU4F2 |
| 0.59606 | 0.000286 | SNRPG |
| 0.739616 | 0.000286 | FSD1L |
| 0.592771 | 0.000289 | DNAJB11 |
| 0.667763 | 0.000289 | NTMT1 |
| 0.726407 | 0.000291 | GFPT1 |
| 0.638936 | 0.000291 | KLF10 |
| 0.776131 | 0.000292 | MCM8 |
| 0.638334 | 0.000292 | LGALS3 |
| 0.62777 | 0.000292 | ZNF259 /// ZNF259P1 |
| 0.625038 | 0.000293 | ZNF23 |
| 0.748052 | 0.000293 | NUP43 |
| 0.60567 | 0.000302 | FRG1 /// LOC100289097 /// LOC100996779 |
| 0.612432 | 0.000304 | CCNL1 |
| 0.750909 | 0.000304 | LOC100509445 /// LOC728715 /// OVOS2 |
| 0.590939 | 0.000304 | AREG /// AREGB |
| 0.610293 | 0.000305 | AIMP2 |
| 0.626808 | 0.000305 | ZMYND19 |
| 0.769595 | 0.000308 | MYBL1 |
| 1.152426 | 0.000308 | RBM24 |
| 0.723699 | 0.000309 | HSPB8 |
| 0.661083 | 0.000315 | ASNS |
| 0.586631 | 0.000316 | MRPL37 |
| 0.598186 | 0.000318 | ELOVL7 |
| 0.601547 | 0.00032 | HCCS |
| 0.603102 | 0.00032 | RNF166 |
| 0.73137 | 0.000321 | THUMPD2 |
| 0.633863 | 0.000325 | MKI67IP |
| 0.592223 | 0.000326 | OSER1 |
| 0.783747 | 0.000326 | KIAA1731 |
| 0.613813 | 0.000328 | E2F6 |
| 0.58913 | 0.00033 | ZNF107 |
| 0.66463 | 0.00033 | EXPH5 |
| 0.602723 | 0.000333 | ITGAM |
| 0.606365 | 0.000333 | RSRC2 |
| 0.63599 | 0.000334 | WDR75 |
| 0.660533 | 0.000336 | SPIRE2 |
| 0.634885 | 0.000337 | ZFAS1 |
| 0.713031 | 0.00034 | NUPL2 |
| 0.703397 | 0.000342 | MURC |
| 0.613667 | 0.000343 | DCUN1D3 |
| 1.750204 | 0.000343 | MMP10 |
| 0.662002 | 0.000344 | ILKAP |
| 0.628727 | 0.000345 | DHFR |
| 0.884914 | 0.000349 | TNFSF15 |
| 0.67758 | 0.00035 | SLC7A6OS |
| 0.77101 | 0.00035 | PSPC1 |
| 0.615935 | 0.000351 | COMMD4 |
| 0.727082 | 0.000351 | PUS3 |
| 1.838918 | 0.000352 | C7orf57 |
| 0.6053 | 0.000355 | DERA |
| 0.670356 | 0.000355 | IL11 |
| 1.672088 | 0.000357 | MIR155 /// MIR155HG |
| 1.222081 | 0.000358 | LINC00669 |
| 0.66954 | 0.000359 | RBBP5 |
| 0.594416 | 0.000365 | HSPH1 |
| 0.593442 | 0.00037 | MGME1 |
| 0.603312 | 0.000371 | RFC4 |
| 0.596987 | 0.000373 | FAS |
| 0.585395 | 0.000374 | XPO5 |
| 0.587826 | 0.000378 | EMP3 |
| 0.779805 | 0.000379 | FCRLB |
| 0.814664 | 0.000379 | SLFN13 |
| 0.595513 | 0.000383 | P2RX4 |
| 0.68045 | 0.000385 | APOBEC3B |
| 0.608429 | 0.000393 | DPH3 |
| 0.721653 | 0.000396 | NRXN3 |
| 0.776528 | 0.000397 | ZMYM5 |
| 0.795223 | 0.000403 | ARNTL2 |
| 0.645332 | 0.000403 | GK |
| 0.701162 | 0.00041 | ERI1 |
| 0.59554 | 0.000412 | HEXB |
| 0.585171 | 0.000413 | NOP2 |
| 0.603456 | 0.000415 | TOE1 |
| 1.348142 | 0.000416 | PTGS2 |
| 0.59295 | 0.000416 | ATP6V0D1 |
| 0.593832 | 0.000418 | GPR137C |
| 0.627876 | 0.000418 | SLC25A29 |
| 0.606844 | 0.000424 | ZBTB8OS |
| 0.609617 | 0.000429 | PPTC7 |
| 0.593669 | 0.000431 | GRHL1 |
| 0.64376 | 0.000436 | FAM169A |
| 0.842273 | 0.000442 | OTTHUMG00000024019 /// RP11-456P18.2 |
| 0.625316 | 0.000443 | INPP1 |
| 0.598126 | 0.000444 | ZNF625 /// ZNF625-ZNF20 |
| 0.606467 | 0.000454 | PPM1E |
| 0.795213 | 0.000456 | ZNF675 |
| 0.783855 | 0.000459 | RNMTL1 |
| 0.61091 | 0.000462 | GOLGA3 |
| 0.592727 | 0.000476 | CDK11A /// CDK11B |
| 1.239515 | 0.00048 | IL20 |
| 0.60271 | 0.000485 | C19orf40 |
| 0.625403 | 0.000496 | MSH2 |
| 0.58517 | 0.000499 | PCID2 |
| 0.601829 | 0.000501 | EID3 |
| 0.782569 | 0.000501 | PBLD |
| 0.609749 | 0.000507 | CCRN4L |
| 0.595805 | 0.00051 | METTL21A |
| 0.773639 | 0.000516 | TFPI2 |
| 0.642968 | 0.000516 | SIPA1L2 |
| 0.63958 | 0.000519 | AP1S3 |
| 0.639456 | 0.000528 | FOSB |
| 1.040374 | 0.000528 | ADRB1 |
| 0.60785 | 0.000528 | KIAA0226L |
| 0.609987 | 0.000529 | ETS1 |
| 0.832956 | 0.00053 | PMS1 |
| 0.622066 | 0.000531 | EP400NL |
| 0.5861 | 0.000534 | ZUFSP |
| 0.588719 | 0.000535 | POLR3K |
| 0.88684 | 0.000542 | ZFAND2A |
| 0.633556 | 0.000545 | ENC1 |
| 0.618049 | 0.000545 | SOGA2 |
| 0.638625 | 0.000558 | STRA13 |
| 0.626859 | 0.000559 | KDELR3 |
| 0.708258 | 0.000563 | LRRC8E |
| 0.604076 | 0.000565 | MIS12 |
| 0.748764 | 0.000568 | ZNF597 |
| 0.622647 | 0.000571 | LRP5L |
| 0.623449 | 0.000574 | CHAC1 |
| 0.612701 | 0.000578 | ANKLE1 |
| 0.842611 | 0.00058 | GAS2L3 |
| 0.612801 | 0.000587 | UFD1L |
| 0.749397 | 0.000592 | SCLY |
| 0.687802 | 0.000596 | ATAD5 |
| 0.697089 | 0.000597 | F3 |
| 0.628403 | 0.000597 | LMCD1 |
| 0.675234 | 0.000605 | ESF1 |
| 0.753922 | 0.00061 | GZF1 |
| 0.778285 | 0.000624 | CEP76 |
| 0.637247 | 0.000625 | LOC442075 |
| 0.658869 | 0.000636 | OASL |
| 0.661756 | 0.000637 | CMPK2 |
| 0.779123 | 0.000638 | GADD45G |
| 0.712026 | 0.000638 | PQLC2 |
| 0.595469 | 0.000639 | MARS |
| 0.656949 | 0.00065 | YOD1 |
| 0.735157 | 0.000651 | LOC283278 |
| 0.687022 | 0.000658 | LACTB2 |
| 0.587634 | 0.000659 | GINS4 |
| 0.586923 | 0.000667 | NR2C2AP |
| 0.6622 | 0.000667 | SHISA8 |
| 0.661302 | 0.000679 | NAF1 |
| 0.611941 | 0.000688 | ABTB2 |
| 0.586837 | 0.000689 | RAD54L |
| 0.586606 | 0.000689 | RGS2 |
| 0.952138 | 0.000691 | LOC100507672 |
| 0.647554 | 0.000694 | EXOC8 |
| 0.934605 | 0.000697 | RAB30 |
| 0.646783 | 0.000698 | HPSE |
| 0.68495 | 0.00071 | TRIM35 |
| 0.76943 | 0.000712 | DCTN5 |
| 0.713168 | 0.000726 | CHGB |
| 0.613175 | 0.000731 | NFE2L2 |
| 0.603721 | 0.000737 | NFIL3 |
| 0.602788 | 0.000739 | POLD3 |
| 0.684267 | 0.000741 | LIG4 |
| 0.639447 | 0.000742 | RAD51 |
| 0.62552 | 0.000766 | TRAF4 |
| 0.660839 | 0.000769 | EIF3C |
| 0.766218 | 0.000777 | ANKRD30B |
| 0.749806 | 0.00078 | TNFAIP6 |
| 0.6594 | 0.000785 | AP3B2 |
| 0.674087 | 0.000794 | CREB5 |
| 0.673357 | 0.000804 | C16orf59 |
| 0.783838 | 0.000808 | LRRN3 |
| 0.6155 | 0.000817 | TARS |
| 0.625351 | 0.000832 | IL6R |
| 0.659164 | 0.000841 | SLC1A4 |
| 0.680823 | 0.000848 | TYW5 |
| 0.717789 | 0.000857 | RASSF1 |
| 0.761759 | 0.000859 | KALRN |
| 0.693501 | 0.000865 | PALM2 |
| 0.739896 | 0.000866 | TRAPPC13 |
| 0.662037 | 0.000866 | PLEKHA7 |
| 0.703551 | 0.000871 | LOC100131067 |
| 0.61669 | 0.000899 | CCL5 |
| 0.757847 | 0.000909 | ELOVL2 |
| 0.807304 | 0.000919 | ZNF567 |
| 0.655052 | 0.000922 | ANKRD20A1 /// ANKRD20A11P /// ANKRD20A2 /// ANKRD20A3 /// ANKRD20A4 /// ANKRD20A5P /// ANKRD20A9P /// LOC101059935 |
| 0.902851 | 0.000922 | KBTBD8 |
| 0.923636 | 0.000931 | INPP4B |
| 0.703187 | 0.000983 | EPB41L5 |
| 0.614006 | 0.001003 | HAS3 |
| 0.916887 | 0.001005 | RASAL2 |
| 0.686279 | 0.001006 | FAM86A |
| 0.60502 | 0.001014 | FAM58A |
| 0.633013 | 0.001024 | GUSBP1 /// GUSBP4 /// LOC100996497 |
| 0.646137 | 0.001037 | ZNF35 |
| 0.667366 | 0.001054 | FAM126B |
| 0.727636 | 0.001067 | LOC100506392 |
| 0.751399 | 0.001114 | CCL3 /// CCL3L1 /// CCL3L3 /// LOC101060267 |
| 0.720125 | 0.001189 | ABHD5 |
| 0.637741 | 0.001195 | ISG20L2 |
| 0.884349 | 0.001222 | SH2D3C |
| 0.660444 | 0.001237 | NAA16 |
| 0.592549 | 0.00124 | HUS1 |
| 0.617902 | 0.001247 | ANKRD13A |
| 0.59322 | 0.001266 | SLC22A4 |
| 0.84042 | 0.001297 | LOC374443 |
| 0.627291 | 0.001303 | AS3MT |
| 0.73479 | 0.001331 | PHC3 |
| 1.032477 | 0.001344 | TNFRSF9 |
| 0.625389 | 0.001344 | CLSPN |
| 0.756331 | 0.001432 | WDR53 |
| 0.586028 | 0.001436 | PPM1D |
| 0.70842 | 0.001458 | SAMD4A |
| 0.635681 | 0.001473 | OTTHUMG00000172986 /// RP11-819C21.1 |
| 0.824952 | 0.001476 | EBI3 |
| 0.609627 | 0.001577 | C5orf22 |
| 0.806131 | 0.00159 | OR51B6 |
| 1.104206 | 0.001616 | LIPG |
| 0.746017 | 0.001654 | MICB |
| 0.672072 | 0.001695 | PRADC1 |
| 0.718331 | 0.001712 | ARRDC4 |
| 0.58862 | 0.001772 | VGLL3 |
| 0.591916 | 0.001776 | CAMTA1 |
| 0.75649 | 0.001819 | SNAI2 |
| 0.599412 | 0.001948 | CPEB4 |
| 0.827129 | 0.002004 | OTTHUMG00000170055 /// RP11-626I20.3 |
| 0.656704 | 0.002044 | LINC00467 |
| 0.814566 | 0.002049 | RASEF |
| 1.102188 | 0.002132 | KIF27 |
| 0.863174 | 0.002142 | DLGAP1-AS2 |
| 0.66095 | 0.002159 | CHRNA5 |
| 0.651432 | 0.002163 | LAYN |
| 0.701088 | 0.002298 | PLA2G4C |
| 0.742693 | 0.002439 | EEA1 |
| 0.594211 | 0.00245 | UBE2W |
| 0.639146 | 0.002488 | ITPKA |
| 0.586095 | 0.002505 | CYP27B1 |
| 0.610906 | 0.002578 | ACER3 |
| 0.642384 | 0.002602 | ALMS1-IT1 /// ALMS1-IT1 |
| 0.629326 | 0.002685 | MARVELD2 |
| 0.713395 | 0.002759 | SLC30A1 |
| 0.596699 | 0.002816 | DHRS11 |
| 0.84316 | 0.002928 | ASL |
| 0.67486 | 0.002994 | ZNF79 |
| 0.671456 | 0.003003 | KLF4 |
| 0.586976 | 0.003038 | ZC3H12C |
| 0.706361 | 0.003075 | NPAT |
| 0.647033 | 0.003245 | EGF |
| 0.587104 | 0.003314 | CHST11 |
| 0.825545 | 0.00342 | PCSK1 |
| 0.795492 | 0.003532 | THAP9 |
| 0.606352 | 0.003841 | ARL14 |
| 0.664771 | 0.003927 | ARC |
| 0.608274 | 0.004364 | MC4R |
| 0.660538 | 0.004728 | ATP1B3 |
| 0.605462 | 0.005068 | LIMK2 |
| 0.855198 | 0.005534 | EGR3 |
| 0.817398 | 0.005829 | ZNF492 |
| 0.588484 | 0.006245 | CEP290 |
| 0.734264 | 0.006295 | SERAC1 |
| 0.661607 | 0.006841 | ETV5 |
| 0.74729 | 0.007315 | CCL4 |
| 0.81209 | 0.007415 | ZNF530 |
| 0.713521 | 0.007422 | IDI2-AS1 |
| 0.59717 | 0.01076 | FLJ14186 /// LOC100996903 /// LOC101059947 /// LOC441124 /// LOC729021 /// LOC729218 |
| 0.628214 | 0.011435 | ZNF256 |
| 0.657042 | 0.011529 | ZNF507 |
| 0.934619 | 0.013083 | LOC100506377 |
| 0.817388 | 0.013128 | AC104135.4 /// OTTHUMG00000152931 |
| 0.769119 | 0.013141 | HMOX1 |
| 0.69466 | 0.013754 | C10orf118 |
| 0.759616 | 0.015173 | ARID5B |
| 0.629751 | 0.015755 | MFAP5 |
| 0.622542 | 0.018238 | ALS2 |
| 0.625584 | 0.023146 | OTTHUMG00000172020 /// RP11-432J9.6 |
| 0.593408 | 0.025729 | FAM105A |
| 0.669004 | 0.034604 | CCDC96 |
