# Supplementary material for: p53 modeling as a route to mesothelioma patients stratification and novel therapeutic identification
Source: J Transl Med. 2018 Oct 13;16:282. doi: 10.1186/s12967-018-1650-0 (PMC6186085; doi:10.1186/s12967-018-1650-0)
Supplement: Supplementary file 6 — Additional file 6: Table S6. Gem vs ctrl downregulated genes. [file 12967_2018_1650_MOESM6_ESM.docx]

**Table S6:** Gem vs ctrl downregulated genes

| **logFC** | **P.Value** | **gene_symbol** |
| --- | --- | --- |
| -2.27579 | 1.03E-07 | ID2 /// ID2B |
| -2.06401 | 1.34E-07 | KIF20A |
| -1.55548 | 8.28E-07 | PSRC1 |
| -1.54778 | 9.23E-07 | VIT |
| -1.6876 | 1.10E-06 | H1F0 |
| -1.75477 | 1.20E-06 | GAL3ST1 |
| -1.50969 | 1.84E-06 | ID2 |
| -1.35358 | 2.13E-06 | DDIT4 |
| -1.38506 | 2.30E-06 | MN1 |
| -1.44462 | 2.35E-06 | OTTHUMG00000032910 /// RP11-157P1.4 |
| -1.51293 | 2.57E-06 | HIST1H2BC |
| -1.40607 | 3.67E-06 | BTN3A3 |
| -1.60693 | 3.87E-06 | SERPINB9 |
| -1.28142 | 3.94E-06 | PIF1 |
| -1.31015 | 4.07E-06 | SEMA6D |
| -1.22501 | 4.49E-06 | LOC100996341 |
| -1.47149 | 4.60E-06 | DLGAP5 |
| -1.17886 | 5.02E-06 | BTN3A2 |
| -1.52651 | 5.13E-06 | LOC100507311 |
| -1.14541 | 5.61E-06 | LOC150622 /// LOC400940 |
| -1.16478 | 5.74E-06 | AGTR1 |
| -1.23486 | 6.13E-06 | LOC339535 |
| -1.19969 | 6.41E-06 | BTN3A2 /// BTN3A3 |
| -1.1605 | 6.43E-06 | SMTNL2 |
| -1.56973 | 6.58E-06 | SPA17 |
| -1.15312 | 6.97E-06 | FAM162A |
| -1.19779 | 7.21E-06 | PTTG1 |
| -1.38703 | 7.98E-06 | MIR210HG |
| -1.13996 | 8.08E-06 | ITIH5 |
| -1.12748 | 8.19E-06 | AK9 |
| -1.34286 | 8.54E-06 | PTGIS |
| -1.11662 | 8.77E-06 | PLK1 |
| -1.30827 | 9.01E-06 | HIST2H2BE |
| -1.0962 | 9.30E-06 | INHBB |
| -1.48025 | 9.64E-06 | LOC150622 |
| -1.58909 | 9.85E-06 | TYRP1 |
| -1.018 | 1.13E-05 | PCDHB5 |
| -1.32903 | 1.15E-05 | HIST2H2AA3 /// HIST2H2AA4 |
| -1.18462 | 1.16E-05 | NUDT13 |
| -1.13913 | 1.18E-05 | CRYZ |
| -1.12027 | 1.22E-05 | SLC16A4 |
| -1.0471 | 1.24E-05 | TSPAN31 |
| -0.99342 | 1.26E-05 | FOXO6 /// FOXO6 |
| -1.18969 | 1.29E-05 | C1orf213 |
| -1.86253 | 1.37E-05 | RNASE4 |
| -1.07852 | 1.42E-05 | IER5L |
| -1.10093 | 1.42E-05 | LOC100506934 |
| -1.70833 | 1.43E-05 | MLLT4-AS1 |
| -1.3042 | 1.47E-05 | TNFAIP8L1 |
| -1.00159 | 1.56E-05 | CCDC85C |
| -1.04605 | 1.59E-05 | FAM224A /// FAM224B |
| -1.99279 | 1.59E-05 | HIST1H3H |
| -1.08648 | 1.64E-05 | SLITRK5 |
| -1.15079 | 1.67E-05 | TPBG |
| -0.96886 | 1.75E-05 | DHRS3 |
| -0.94595 | 1.77E-05 | SUN2 |
| -1.27412 | 1.79E-05 | LOC151009 /// LOC440894 |
| -1.01917 | 1.80E-05 | CNTN3 |
| -1.08511 | 1.83E-05 | H2BFS |
| -0.96272 | 1.93E-05 | C10orf114 |
| -1.04169 | 1.93E-05 | ZSCAN31 |
| -0.97052 | 1.93E-05 | EBPL |
| -1.13895 | 2.03E-05 | HIST1H2BH |
| -1.18901 | 2.08E-05 | HIST1H2BD |
| -0.97898 | 2.09E-05 | KANK4 |
| -0.96012 | 2.23E-05 | TMEM256 |
| -0.93677 | 2.31E-05 | CENPA /// SLC35F6 |
| -0.96458 | 2.35E-05 | ST6GAL2 |
| -1.09008 | 2.35E-05 | NFATC4 |
| -1.03773 | 2.37E-05 | LAMA5 |
| -1.01444 | 2.46E-05 | FBXO16 /// ZNF395 |
| -0.94185 | 2.49E-05 | SYT17 |
| -1.1682 | 2.51E-05 | MGC24103 |
| -0.92119 | 2.54E-05 | PLCXD1 |
| -0.9345 | 2.56E-05 | TPM1 |
| -0.88183 | 2.63E-05 | DHRS2 |
| -0.98574 | 2.70E-05 | LOC100507486 |
| -1.49106 | 2.74E-05 | CCDC80 |
| -1.06474 | 2.76E-05 | GPSM2 |
| -0.91092 | 2.84E-05 | GPI |
| -0.89665 | 2.85E-05 | HIST1H2BK |
| -0.86833 | 2.87E-05 | ERMP1 |
| -0.95679 | 2.99E-05 | ATF7IP2 /// LOC100287628 |
| -0.88948 | 2.99E-05 | RNF150 |
| -0.87363 | 3.00E-05 | OTTHUMG00000162476 /// RP11-974F13.6 |
| -1.28946 | 3.04E-05 | LOC100507303 |
| -1.04803 | 3.20E-05 | PPP1R3C |
| -0.86212 | 3.20E-05 | LOC100507316 |
| -1.16703 | 3.31E-05 | BNC2 |
| -0.89507 | 3.39E-05 | RPS15A |
| -1.12284 | 3.39E-05 | CCNG2 |
| -0.88386 | 3.41E-05 | LXN |
| -0.93978 | 3.46E-05 | CARNS1 |
| -1.01994 | 3.46E-05 | MIRLET7BHG |
| -0.87654 | 3.49E-05 | MLLT3 |
| -0.84373 | 3.60E-05 | GSE1 |
| -0.94105 | 3.61E-05 | STON1 |
| -0.92639 | 3.62E-05 | PRSS16 |
| -0.83951 | 3.64E-05 | FGF18 |
| -0.928 | 3.66E-05 | TNNI3K |
| -0.88294 | 3.68E-05 | PTRHD1 |
| -1.03455 | 3.72E-05 | CEP70 |
| -0.88862 | 3.72E-05 | FAM13C |
| -0.88834 | 3.73E-05 | THAP2 |
| -0.90201 | 3.76E-05 | PGK1 |
| -1.13869 | 3.76E-05 | CDH18 |
| -0.89144 | 3.88E-05 | KBTBD7 |
| -0.88037 | 3.89E-05 | ERVFRD-1 |
| -1.20743 | 3.90E-05 | BNIP3 |
| -0.87903 | 3.91E-05 | PBX1 |
| -1.09533 | 3.95E-05 | HIST1H1C |
| -1.08673 | 4.02E-05 | KLHL4 |
| -1.14842 | 4.04E-05 | PPFIA4 |
| -0.91901 | 4.12E-05 | DAPK1 |
| -0.9152 | 4.19E-05 | SLC39A10 |
| -0.98738 | 4.21E-05 | ITGA3 |
| -0.81702 | 4.23E-05 | IGSF9 |
| -0.85414 | 4.23E-05 | OTTHUMG00000180272 /// RP11-567M16.4 |
| -0.85649 | 4.31E-05 | H19 /// MIR675 |
| -0.82445 | 4.32E-05 | RHOU |
| -0.89096 | 4.34E-05 | CDCA3 |
| -0.83316 | 4.35E-05 | BCHE |
| -0.87727 | 4.35E-05 | ARL6IP5 |
| -0.88989 | 4.42E-05 | GYS1 |
| -0.84975 | 4.43E-05 | PTTG3P |
| -0.94537 | 4.45E-05 | YPEL2 |
| -0.87065 | 4.47E-05 | ANG |
| -1.49812 | 4.50E-05 | TTC30A |
| -0.87476 | 4.51E-05 | SSBP3 |
| -0.8937 | 4.52E-05 | HIST3H2A |
| -1.04699 | 4.56E-05 | WT1-AS |
| -0.96169 | 4.70E-05 | SLC2A1 |
| -1.09045 | 4.72E-05 | LOC151009 |
| -0.81736 | 4.74E-05 | NIPSNAP1 |
| -1.62856 | 4.79E-05 | STC1 |
| -1.08487 | 5.02E-05 | MXI1 |
| -0.79305 | 5.06E-05 | PAK4 |
| -0.82871 | 5.11E-05 | MED11 |
| -1.05049 | 5.32E-05 | HIST1H2BC /// HIST1H2BE /// HIST1H2BF /// HIST1H2BG /// HIST1H2BI |
| -0.92819 | 5.34E-05 | PTPRF |
| -0.83884 | 5.56E-05 | SHB |
| -0.77723 | 5.63E-05 | ZNF395 |
| -1.02236 | 5.64E-05 | RPL31 /// TBC1D8 |
| -0.94685 | 5.65E-05 | CTA-29F11.1 /// OTTHUMG00000172744 |
| -0.78681 | 5.66E-05 | IL7 |
| -0.83029 | 5.72E-05 | LDOC1 |
| -0.77754 | 5.78E-05 | GSTT1 |
| -1.30097 | 5.82E-05 | NDRG1 |
| -0.96649 | 5.83E-05 | SLITRK6 |
| -1.00367 | 5.98E-05 | LOC100287896 |
| -0.84828 | 5.99E-05 | TRAM1L1 |
| -0.79424 | 6.09E-05 | ANGPTL4 |
| -0.82591 | 6.25E-05 | LAMB1 |
| -0.79434 | 6.37E-05 | AURKA |
| -1.13493 | 6.49E-05 | LOC646903 |
| -0.86165 | 6.54E-05 | MUS81 |
| -0.7685 | 6.66E-05 | SELENBP1 |
| -0.8389 | 6.72E-05 | HCFC1R1 |
| -0.88894 | 6.77E-05 | GUSBP3 /// GUSBP9 /// LOC100653061 /// LOC101060519 |
| -0.76063 | 6.82E-05 | HMHA1 |
| -0.85059 | 6.85E-05 | H1FX |
| -0.8541 | 6.93E-05 | S100A4 |
| -0.74671 | 6.97E-05 | PKM |
| -0.77222 | 7.09E-05 | ELFN1 |
| -0.84981 | 7.09E-05 | BTN3A1 |
| -0.97196 | 7.16E-05 | FKBP7 |
| -1.0739 | 7.23E-05 | C4orf3 |
| -0.83006 | 7.26E-05 | CBX7 |
| -0.86907 | 7.26E-05 | LOC100128822 |
| -0.91932 | 7.35E-05 | ZADH2 |
| -0.82043 | 7.55E-05 | CAV1 |
| -0.75241 | 7.58E-05 | IDH1 |
| -0.84842 | 7.60E-05 | CCNA1 |
| -0.85106 | 7.64E-05 | C1GALT1C1 |
| -0.78512 | 7.71E-05 | TMEM161B-AS1 |
| -1.04327 | 7.83E-05 | ZDHHC8P1 |
| -0.75243 | 7.92E-05 | DEPDC1 |
| -0.73236 | 8.02E-05 | GLIS2 |
| -0.92842 | 8.02E-05 | THNSL1 |
| -0.78975 | 8.07E-05 | FZD2 |
| -0.87949 | 8.10E-05 | MSL1 |
| -0.75696 | 8.24E-05 | OTTHUMG00000176823 /// RP11-846E15.2 |
| -1.24919 | 8.29E-05 | HIST1H2BC /// HIST1H2BE /// HIST1H2BF /// HIST1H2BG /// HIST1H2BI /// NCALD |
| -0.74407 | 8.32E-05 | FBXL20 |
| -0.79102 | 8.38E-05 | LOC100506990 |
| -0.73366 | 8.40E-05 | C3orf70 |
| -0.79411 | 8.58E-05 | PODXL |
| -0.73475 | 8.65E-05 | ACO1 |
| -0.73719 | 8.69E-05 | CRIP2 |
| -0.74104 | 8.71E-05 | CASP6 |
| -1.09166 | 8.74E-05 | DAPK1-IT1 |
| -1.06995 | 8.77E-05 | GLTSCR1L |
| -0.74216 | 8.87E-05 | QPRT |
| -0.95472 | 8.94E-05 | RPL31 |
| -0.73033 | 9.04E-05 | CLDN15 |
| -0.71831 | 9.27E-05 | C10orf54 |
| -0.75719 | 9.30E-05 | FLJ38717 |
| -0.95254 | 9.48E-05 | PPP3CB |
| -0.84198 | 9.51E-05 | BNIP3L |
| -1.56942 | 9.52E-05 | HIST1H2AC |
| -0.87575 | 9.57E-05 | AUH |
| -0.81765 | 9.61E-05 | LINC00173 |
| -0.92835 | 9.66E-05 | C11orf70 |
| -1.31088 | 9.68E-05 | SATB1 |
| -0.78332 | 9.71E-05 | LRP2 |
| -0.81582 | 9.73E-05 | CAHM |
| -0.9777 | 9.77E-05 | PTOV1 |
| -0.73649 | 9.97E-05 | S100A10 |
| -0.71906 | 1.00E-04 | LGALS3BP |
| -1.1586 | 0.000101 | CXorf24 |
| -0.72244 | 0.000101 | SSNA1 |
| -0.89266 | 0.000102 | RAB33A |
| -0.73477 | 0.000102 | VPS28 |
| -0.72011 | 0.000103 | NDRG3 |
| -0.94749 | 0.000104 | ZMYND8 |
| -1.04806 | 0.000104 | ZBED5-AS1 |
| -0.94474 | 0.000104 | POSTN |
| -0.94187 | 0.000105 | DFFB |
| -0.73362 | 0.000106 | HSD17B11 |
| -1.12299 | 0.000106 | PRO2964 |
| -0.70577 | 0.000108 | OLFML1 |
| -0.72399 | 0.000108 | MORF4L2-AS1 |
| -0.71502 | 0.000113 | SLC44A1 |
| -0.74291 | 0.000114 | FRY |
| -0.80429 | 0.000115 | FAT4 |
| -0.88625 | 0.000116 | ZNF703 |
| -0.75418 | 0.000117 | MRPL48 |
| -0.86743 | 0.000118 | HIST1H2AD /// HIST1H3A /// HIST1H3B /// HIST1H3C /// HIST1H3D /// HIST1H3E /// HIST1H3F /// HIST1H3G /// HIST1H3H /// HIST1H3I /// HIST1H3J |
| -0.73988 | 0.000118 | LOC101060440 /// LOC101060471 /// LOC101060522 /// LOC440434 /// NPEPPS /// TBC1D3 |
| -0.76881 | 0.000118 | AGAP1 |
| -0.90311 | 0.00012 | TNS3 |
| -0.79461 | 0.000121 | CRB2 |
| -0.70207 | 0.000122 | ST3GAL5 |
| -1.17499 | 0.000123 | ATG4C |
| -0.68773 | 0.000123 | SLC7A7 |
| -0.77431 | 0.000124 | PHYKPL |
| -0.89367 | 0.000124 | ZFX |
| -0.77759 | 0.000126 | KRT15 |
| -0.78666 | 0.000126 | OTTHUMG00000173203 /// RP11-680G24.5 |
| -0.88828 | 0.000128 | OTTHUMG00000178019 /// RP11-199F11.2 |
| -0.82737 | 0.000129 | BCAR1 |
| -0.75822 | 0.000129 | FAM168A |
| -0.74509 | 0.00013 | CRELD1 |
| -0.87508 | 0.00013 | E2F5 |
| -0.85512 | 0.00013 | GSTA4 |
| -0.68327 | 0.000132 | GTPBP8 |
| -1.17397 | 0.000137 | SLITRK1 |
| -0.72166 | 0.000141 | LINC00920 |
| -0.95505 | 0.000143 | SPAG4 |
| -0.71026 | 0.000143 | KRT8 |
| -0.72038 | 0.000143 | NEO1 |
| -0.77702 | 0.000144 | POLG2 |
| -0.79308 | 0.000145 | FANCF |
| -0.80125 | 0.000146 | FAM196A |
| -0.69389 | 0.000147 | CASP8 |
| -0.72273 | 0.000147 | SARS2 |
| -0.65864 | 0.000148 | LOC100505715 |
| -0.73504 | 0.000148 | CHMP2A |
| -0.66213 | 0.000149 | CNOT8 |
| -0.67091 | 0.000149 | TNFRSF1A |
| -1.13417 | 0.00015 | PCDHB16 |
| -0.67155 | 0.00015 | DANCR |
| -0.97619 | 0.00015 | GRB14 |
| -0.92966 | 0.000151 | INSIG2 |
| -0.71399 | 0.000153 | SLC16A3 |
| -1.00817 | 0.000153 | KBTBD3 |
| -0.83019 | 0.000153 | OSBPL1A |
| -0.82195 | 0.000153 | CBLN2 |
| -0.66164 | 0.000154 | ATP2B4 |
| -0.9381 | 0.000155 | COL11A1 |
| -0.82665 | 0.000155 | TRIM59 |
| -0.65528 | 0.000156 | FAM83D |
| -0.92394 | 0.000156 | SRSF8 |
| -0.65678 | 0.000158 | CCNB1 |
| -0.70477 | 0.000158 | UPK3B |
| -0.68102 | 0.000159 | EBAG9 |
| -0.78583 | 0.00016 | FAM102B |
| -0.6838 | 0.000161 | PPP2R4 |
| -0.64819 | 0.000161 | TOP2A |
| -0.86037 | 0.000162 | PSD3 |
| -0.65439 | 0.000162 | LOC100507054 |
| -0.82914 | 0.000163 | ITGB4 |
| -0.66057 | 0.000163 | DYNC2H1 |
| -0.71312 | 0.000166 | VPS51 |
| -1.03901 | 0.000166 | SSPN |
| -0.87839 | 0.000167 | NAP1L3 |
| -0.67103 | 0.000169 | FOXF2 |
| -0.74616 | 0.000169 | RBM38 |
| -0.71926 | 0.00017 | KBTBD11 |
| -0.64 | 0.00017 | THOC6 |
| -0.66557 | 0.00017 | S100A11 |
| -0.78834 | 0.00017 | LIX1L /// LOC101060547 |
| -0.66968 | 0.000171 | CBLB |
| -0.70557 | 0.000172 | SEMA4B |
| -0.97183 | 0.000172 | BAIAP2-AS1 |
| -0.65581 | 0.000173 | YY1 |
| -0.73649 | 0.000174 | FBXL2 |
| -1.08168 | 0.000174 | C12orf76 |
| -0.72408 | 0.000175 | AMN1 |
| -0.64147 | 0.000178 | TPRN |
| -0.63363 | 0.00018 | LOC646762 |
| -0.67979 | 0.000181 | APRT |
| -0.8256 | 0.000182 | GLS |
| -0.84382 | 0.000182 | ZNF514 |
| -0.6993 | 0.000184 | PFKFB3 |
| -0.66078 | 0.000185 | PEX2 |
| -0.79242 | 0.000185 | C14orf132 |
| -0.6698 | 0.000186 | VASN |
| -0.90004 | 0.000186 | C15orf57 |
| -0.65184 | 0.000186 | ALDH1A3 |
| -0.64373 | 0.000188 | NARF |
| -0.74258 | 0.000188 | ACTG1P4 /// AMY2B /// RNPC3 |
| -0.71275 | 0.000191 | CAV2 |
| -0.63494 | 0.000192 | DDX41 |
| -0.62665 | 0.000195 | COL18A1 |
| -0.62712 | 0.000196 | ZNF618 |
| -0.65008 | 0.000197 | ALKBH5 |
| -0.81151 | 0.000198 | CENPA |
| -0.6455 | 0.0002 | NDRG4 |
| -0.85406 | 0.000202 | BTD |
| -0.77841 | 0.000203 | CDC20 |
| -0.92289 | 0.000204 | RAD51-AS1 |
| -0.65802 | 0.000205 | FAM8A1 |
| -0.6375 | 0.000206 | DOK7 |
| -0.76424 | 0.000206 | SBF2 |
| -0.66707 | 0.000208 | SALL2 |
| -1.58554 | 0.000208 | DUSP5P1 |
| -0.88443 | 0.00021 | TOB1-AS1 |
| -0.63817 | 0.00021 | KIAA1522 |
| -0.63509 | 0.000211 | TMEM45A |
| -0.6269 | 0.000211 | LSM14A |
| -0.66687 | 0.000212 | SULF1 |
| -0.68234 | 0.000212 | TBC1D5 |
| -0.61974 | 0.000213 | SLC40A1 |
| -0.69218 | 0.000215 | LOC100507547 /// PRRT1 |
| -0.85093 | 0.000216 | PEX11A |
| -0.6199 | 0.000217 | SEMA3F |
| -0.72451 | 0.000218 | IPW /// LOC100506948 /// SNORD107 /// SNORD115-13 /// SNORD115-26 /// SNORD115-7 /// SNORD116-28 /// SNRPN |
| -0.86957 | 0.000218 | SAPCD2 |
| -0.65241 | 0.000224 | TMEM219 |
| -0.61945 | 0.000225 | IFT172 |
| -0.61928 | 0.000228 | NPEPPS |
| -1.44232 | 0.000229 | NRN1 |
| -0.80542 | 0.000231 | AK4 /// LOC100507855 |
| -0.60769 | 0.000231 | AES |
| -0.63004 | 0.000233 | ERGIC1 |
| -0.80775 | 0.00024 | FSTL3 |
| -0.60758 | 0.000241 | TTLL1 |
| -1.07048 | 0.000241 | CDK19 |
| -0.62329 | 0.000241 | C17orf49 /// RNASEK-C17orf49 |
| -1.03965 | 0.000242 | ALDH6A1 |
| -0.68687 | 0.000242 | LYPD1 |
| -0.66153 | 0.000242 | LOC100510707 /// LOC101060303 /// LOC101060321 /// LOC101060351 /// LOC101060367 /// LOC101060376 /// LOC101060389 /// LOC101060403 /// LOC101060421 /// LOC101060440 /// LOC101060471 /// LOC101060489 /// LOC101060506 /// LOC101060522 /// LOC440434 /// NPEPPS /// TBC1D3 /// TBC1D3F |
| -0.7458 | 0.000242 | ANKRD29 |
| -0.70248 | 0.000242 | FAM217B |
| -0.63896 | 0.000244 | ABHD14B |
| -0.60132 | 0.000244 | ROBO1 |
| -0.83282 | 0.000245 | B9D2 |
| -0.66436 | 0.000245 | CA11 |
| -0.94194 | 0.000245 | PROC |
| -0.73667 | 0.00025 | SHISA3 |
| -0.77386 | 0.00025 | FUT11 |
| -0.64798 | 0.000251 | AGAP2-AS1 |
| -0.62637 | 0.000251 | RRAGA |
| -0.65645 | 0.000254 | NME3 |
| -0.61166 | 0.000254 | SLIT3 |
| -0.77046 | 0.000255 | TRO |
| -0.63122 | 0.000255 | PFN1 |
| -0.74498 | 0.000256 | DET1 |
| -0.81813 | 0.000256 | EIF3J-AS1 |
| -0.74823 | 0.000257 | MIR181A2HG |
| -0.65193 | 0.000259 | LIMCH1 |
| -0.59869 | 0.000259 | UBE2E3 |
| -0.61829 | 0.00026 | VTI1B |
| -0.64559 | 0.000261 | C7orf55 |
| -0.60213 | 0.000262 | LZTS2 |
| -0.63352 | 0.000265 | C15orf52 |
| -0.66273 | 0.000268 | TRIM52 |
| -0.80813 | 0.000274 | LOC100507535 |
| -0.75943 | 0.000276 | NFIA |
| -0.72955 | 0.000276 | SNUPN |
| -0.6668 | 0.000277 | ZNF251 |
| -0.68663 | 0.00028 | RUNX1T1 |
| -0.59567 | 0.00028 | TBCE |
| -0.61112 | 0.000283 | LOC101060235 /// TMSB15A /// TMSB15B |
| -0.64696 | 0.000284 | CENPF |
| -0.60982 | 0.000285 | BBS2 |
| -0.65674 | 0.000285 | USP3 |
| -0.98431 | 0.000286 | PLEKHA2 |
| -0.61683 | 0.000286 | SPIN2A /// SPIN2B |
| -0.83639 | 0.000287 | NRDE2 |
| -0.60326 | 0.000287 | SLC35A5 |
| -0.67514 | 0.000288 | BNC1 |
| -0.66757 | 0.000288 | LY6E |
| -0.65589 | 0.000289 | ILF3-AS1 |
| -1.24856 | 0.00029 | YPEL1 |
| -0.61366 | 0.00029 | ZNF532 |
| -0.83322 | 0.000291 | TSHZ1 |
| -0.89633 | 0.000293 | SERPINB1 |
| -0.75183 | 0.000293 | RAB11FIP4 |
| -0.77383 | 0.000294 | PYGM |
| -0.60746 | 0.000296 | MIR4647 /// SLC35B2 |
| -0.58562 | 0.000299 | EIF2A |
| -0.61817 | 0.000299 | COG7 |
| -0.6328 | 0.0003 | OTTHUMG00000175805 /// RP1-39G22.7 |
| -0.74016 | 0.0003 | NEFL |
| -0.73426 | 0.000301 | C19orf33 |
| -0.59529 | 0.000302 | WASF3 |
| -0.70904 | 0.000304 | FBXL19 |
| -0.59645 | 0.000304 | SLC9A3R1 |
| -0.72838 | 0.000305 | FBXL4 |
| -0.59872 | 0.000305 | SEC31B |
| -0.60811 | 0.000306 | HEATR2 |
| -0.6515 | 0.000306 | TM4SF1 |
| -0.78716 | 0.000307 | LYRM9 |
| -0.7235 | 0.000311 | PITX2 |
| -0.60315 | 0.000311 | JUP |
| -1.49957 | 0.000311 | CTD-2292M16.8 /// OTTHUMG00000178843 |
| -0.88324 | 0.000312 | SMAD7 |
| -0.66451 | 0.000313 | KIF14 |
| -0.84879 | 0.000318 | CTB-174D11.3 /// OTTHUMG00000163878 |
| -0.73266 | 0.000324 | OTTHUMG00000167230 /// RP11-736K20.4 |
| -0.68957 | 0.000324 | GRAMD4 |
| -0.59844 | 0.000326 | RNF44 |
| -0.78832 | 0.000327 | ZSCAN18 |
| -0.61618 | 0.000328 | LOC286161 |
| -1.10761 | 0.000331 | CYP26A1 |
| -0.75311 | 0.000332 | GJA1 |
| -0.663 | 0.000332 | CCNF |
| -1.07794 | 0.000334 | EPYC |
| -0.59786 | 0.000335 | TSPO |
| -0.64008 | 0.000335 | SRI |
| -0.66898 | 0.000337 | MGC21881 |
| -0.65366 | 0.000342 | GXYLT2 |
| -0.62551 | 0.000344 | NDUFB10 |
| -0.70337 | 0.000345 | CTC-504A5.1 /// OTTHUMG00000162885 |
| -0.88306 | 0.000348 | PAPSS2 |
| -0.62689 | 0.00035 | LMBRD1 |
| -0.8303 | 0.000352 | LOC100996464 /// ZNF84 |
| -0.61515 | 0.000352 | LRRN4 |
| -0.59723 | 0.000356 | C8orf82 |
| -0.60288 | 0.00036 | PDGFRB |
| -0.72311 | 0.000361 | GALNS |
| -0.75102 | 0.000363 | LOC100506965 |
| -0.62919 | 0.000364 | SEPT9 |
| -0.6574 | 0.000375 | MICAL2 |
| -0.62839 | 0.000376 | DDHD2 |
| -0.63726 | 0.000377 | SLC18B1 |
| -0.61543 | 0.000378 | MFI2-AS1 |
| -0.70255 | 0.000378 | FIG4 |
| -0.73382 | 0.000382 | LOC150381 |
| -0.6403 | 0.000383 | PAPOLA |
| -0.91457 | 0.000384 | PCBP2 |
| -0.6453 | 0.000385 | CCDC167 |
| -0.69499 | 0.000392 | OTTHUMG00000176821 /// RP11-846E15.4 |
| -0.64306 | 0.000395 | MIR4746 /// UBXN6 |
| -0.62366 | 0.000395 | INO80B /// INO80B-WBP1 /// WBP1 |
| -0.58562 | 0.000395 | LOC100506948 /// SNORD107 /// SNORD115-13 /// SNORD115-26 /// SNORD115-7 /// SNORD116-28 |
| -0.60176 | 0.000396 | CLN3 |
| -0.61911 | 0.000397 | MCC |
| -1.02684 | 0.000398 | LOC101060527 /// NAIP |
| -0.86366 | 0.000399 | ZFX /// ZFY |
| -0.68861 | 0.000401 | PPP1R21 |
| -0.64576 | 0.000403 | ARRB1 |
| -0.7302 | 0.000407 | PABPC5 |
| -0.64579 | 0.000407 | MOSPD3 |
| -0.59634 | 0.000412 | PNPLA6 |
| -0.60304 | 0.000413 | GBE1 |
| -0.7532 | 0.000417 | LINC00094 |
| -0.67528 | 0.000417 | ZNF25 |
| -0.95259 | 0.000418 | LOC100272216 |
| -0.63709 | 0.000421 | SBNO1 |
| -1.11945 | 0.000428 | TTC30B |
| -0.74262 | 0.000434 | SORD |
| -0.86805 | 0.000435 | EID2B |
| -0.65152 | 0.000443 | ZYG11B |
| -0.65858 | 0.000444 | PCDH20 |
| -0.64762 | 0.000445 | TRIM34 /// TRIM6-TRIM34 |
| -0.96792 | 0.000447 | MIR100HG |
| -0.66237 | 0.000449 | AC091133.1 /// OTTHUMG00000132443 |
| -0.60295 | 0.000449 | MRPS28 |
| -0.59908 | 0.000449 | MEGF6 |
| -0.65648 | 0.00045 | TMED4 |
| -0.60129 | 0.000452 | ZNF20 /// ZNF625-ZNF20 |
| -0.74985 | 0.000466 | FRMD4A |
| -0.61897 | 0.000466 | OSER1-AS1 |
| -0.59273 | 0.000468 | FEZ2 |
| -0.62158 | 0.000473 | CCNDBP1 |
| -0.64662 | 0.000477 | NICN1 |
| -0.73046 | 0.000477 | METTL25 |
| -0.62971 | 0.000479 | ILVBL |
| -0.6211 | 0.00048 | ANKZF1 |
| -0.59827 | 0.000482 | FIS1 |
| -0.69599 | 0.000488 | FLJ39632 |
| -0.59091 | 0.000489 | OTTHUMG00000164865 /// RP11-410L14.2 |
| -0.63688 | 0.000498 | MAGED2 |
| -0.69976 | 0.000501 | CYFIP2 |
| -0.91589 | 0.000502 | HCAR3 |
| -0.60951 | 0.000502 | OTTHUMG00000175906 /// RP11-457M11.5 |
| -0.90275 | 0.000505 | NANOG |
| -1.11953 | 0.000507 | OTTHUMG00000167487 /// RP11-178H8.7 |
| -0.59957 | 0.000508 | RAB5B |
| -0.72995 | 0.00051 | GNAZ |
| -0.69581 | 0.000516 | LOC154761 |
| -0.68559 | 0.000518 | NUBP1 |
| -0.66138 | 0.000522 | COL5A1 |
| -0.59217 | 0.000523 | PABPN1 |
| -0.64598 | 0.000536 | OTTHUMG00000170856 /// RP11-33E12.2 |
| -0.6411 | 0.000548 | LOC401397 |
| -0.61818 | 0.000548 | SEMA3C |
| -0.66903 | 0.000549 | C4orf47 |
| -0.81561 | 0.000558 | OTTHUMG00000172405 /// RP11-752G15.7 |
| -1.35515 | 0.000564 | BCL11A |
| -0.63304 | 0.000567 | LOC338620 |
| -0.70777 | 0.000572 | MYO10 |
| -0.96446 | 0.000577 | NREP |
| -1.46144 | 0.000579 | KIAA1984 |
| -0.69154 | 0.000607 | GMEB1 |
| -0.63862 | 0.000608 | KLHL31 |
| -0.82504 | 0.000617 | C1orf191 |
| -0.79263 | 0.000621 | CDKN1C |
| -0.62614 | 0.000623 | GABPB2 |
| -0.61032 | 0.00063 | SLC38A4 |
| -0.86367 | 0.000652 | OTTHUMG00000021298 /// RP11-268G12.1 |
| -0.86461 | 0.000655 | GPM6A |
| -0.7517 | 0.000656 | MIR4800 /// MXD4 |
| -0.62392 | 0.000656 | MARCKS |
| -0.59947 | 0.000661 | ANKRD13B |
| -0.62011 | 0.000662 | APPL1 |
| -1.00806 | 0.000666 | FNTB |
| -0.58769 | 0.000666 | PON2 |
| -0.85892 | 0.000669 | DOK6 |
| -0.8649 | 0.000675 | ARHGAP19 |
| -0.97779 | 0.000689 | FLJ11235 |
| -0.68664 | 0.000691 | AF001548.5 /// OTTHUMG00000177385 |
| -0.63675 | 0.000693 | PLXNB2 |
| -0.63852 | 0.000696 | LOC389834 /// MAFIP /// TEKT4P2 |
| -0.61786 | 0.000697 | TPPP |
| -0.97288 | 0.000707 | MIR10A |
| -0.61013 | 0.000713 | LOC339803 |
| -0.66022 | 0.000725 | VAPA |
| -0.71133 | 0.000728 | MDFI |
| -0.78507 | 0.000736 | CTB-58E17.7 /// OTTHUMG00000178506 |
| -0.63378 | 0.000749 | WDR54 |
| -0.6265 | 0.000756 | MED17 |
| -0.64631 | 0.000759 | ATF6B |
| -0.75215 | 0.000759 | CCDC23 |
| -0.65434 | 0.000763 | CDRT4 /// TVP23C-CDRT4 |
| -0.75599 | 0.000765 | FAM172A |
| -0.82238 | 0.000767 | LOC644656 |
| -0.60761 | 0.000782 | NUDCD2 |
| -0.60278 | 0.000786 | CYB561D1 |
| -0.79653 | 0.000797 | CAT |
| -0.7858 | 0.00081 | ST3GAL4-AS1 |
| -0.59105 | 0.000812 | SPIN3 |
| -0.58967 | 0.000813 | CHRNB1 |
| -0.65115 | 0.000821 | MYO5B |
| -0.58863 | 0.000838 | C7orf55 /// C7orf55-LUC7L2 |
| -0.62394 | 0.000847 | STEAP3 |
| -0.62268 | 0.000849 | SGOL2 |
| -0.66854 | 0.000857 | LOC100507165 |
| -0.63465 | 0.000861 | PDK1 |
| -0.79715 | 0.000862 | LRRFIP1 |
| -0.66433 | 0.000866 | LPHN2 |
| -1.1387 | 0.000873 | GPR160 |
| -0.88542 | 0.00088 | SNRPN /// SNURF |
| -0.58851 | 0.000884 | FLJ44896 |
| -0.6022 | 0.000884 | PRKCDBP |
| -0.76343 | 0.000885 | VMP1 |
| -0.84465 | 0.000887 | MEX3B |
| -0.85015 | 0.000889 | FPGT |
| -0.66544 | 0.0009 | MRPL54 |
| -0.59824 | 0.000907 | BCL9L |
| -0.62922 | 0.000918 | OPN3 |
| -0.61803 | 0.00093 | JARID2 |
| -0.71356 | 0.000938 | PCLO |
| -0.70525 | 0.000944 | CRLS1 |
| -0.67944 | 0.000948 | ZNF880 |
| -0.59768 | 0.000959 | NECAB1 |
| -0.65788 | 0.000961 | LOC254057 |
| -0.58946 | 0.000965 | LINC00963 |
| -0.8985 | 0.000976 | PELI1 |
| -0.77595 | 0.00098 | OTTHUMG00000176181 /// RP11-119F7.5 |
| -0.75986 | 0.000985 | OTTHUMG00000180314 /// RP1-193H18.2 |
| -0.67128 | 0.000987 | BCKDHA |
| -0.69714 | 0.000987 | EDIL3 |
| -0.59924 | 0.000999 | LPCAT2 |
| -0.89599 | 0.001009 | GLI2 |
| -0.58867 | 0.001014 | KCND2 |
| -0.65183 | 0.001015 | HOTAIRM1 |
| -0.76029 | 0.001019 | LOC100507018 |
| -0.59502 | 0.001024 | CTIF |
| -0.81909 | 0.001026 | OTTHUMG00000019884 /// RP11-112J3.16 |
| -0.7353 | 0.001028 | LOC100506303 /// LOC100653149 /// LOC101060483 |
| -0.7349 | 0.001033 | LINC00260 |
| -0.65595 | 0.001033 | IGFBP3 |
| -0.68611 | 0.001052 | MPPED2 |
| -0.74567 | 0.001061 | PRR15 |
| -0.60434 | 0.001064 | RBBP6 |
| -0.83302 | 0.001066 | ZNF785 |
| -0.59733 | 0.001121 | LOC100507577 /// LONP2 |
| -0.61685 | 0.001124 | THRA |
| -0.74009 | 0.001137 | ZNF177 /// ZNF559-ZNF177 |
| -1.03052 | 0.001138 | AKR1C3 |
| -0.59434 | 0.001139 | GABRE /// MIR224 /// MIR452 |
| -0.70317 | 0.001177 | C2orf68 |
| -0.62836 | 0.001189 | MMP24-AS1 |
| -0.59669 | 0.001202 | PFKFB4 |
| -0.64976 | 0.001205 | ZNF558 |
| -0.69458 | 0.001207 | ACADSB |
| -0.58937 | 0.001211 | C19orf70 |
| -0.74823 | 0.001211 | TMEM132B |
| -0.95737 | 0.001221 | TRMT10A |
| -0.63244 | 0.001231 | NR2F2 |
| -0.59932 | 0.00125 | GGA1 |
| -0.98444 | 0.001274 | C7orf60 |
| -1.0087 | 0.001293 | EVI2B |
| -0.66495 | 0.001328 | CTC-241N9.1 /// OTTHUMG00000163262 |
| -0.74754 | 0.001331 | IRS1 |
| -0.60488 | 0.00134 | FAM171A1 |
| -0.73998 | 0.001346 | NOL3 |
| -0.66238 | 0.001348 | MT1X |
| -0.62166 | 0.001348 | CHD2 /// LOC100507217 |
| -0.70291 | 0.001364 | OTTHUMG00000176825 /// RP11-124L9.5 |
| -0.59082 | 0.001377 | FAM64A |
| -0.62252 | 0.001387 | C1orf21 |
| -0.76479 | 0.0014 | TLDC1 |
| -0.6937 | 0.001421 | L3MBTL3 |
| -0.79744 | 0.001445 | TDRD3 |
| -0.66351 | 0.001449 | ZNF493 |
| -0.90929 | 0.001454 | CPE |
| -0.69492 | 0.001456 | RSBN1 |
| -0.60687 | 0.00146 | SMA4 |
| -0.60014 | 0.001463 | DAB1 /// OMA1 |
| -0.70113 | 0.001486 | CA8 |
| -0.63764 | 0.001529 | FSTL5 |
| -0.89245 | 0.001536 | OTTHUMG00000172119 /// RP11-208K4.2 |
| -0.70638 | 0.001537 | SLC35D2 |
| -0.90663 | 0.001544 | CTB-31O20.2 /// OTTHUMG00000175708 |
| -0.60747 | 0.001545 | ACTR3C |
| -0.65394 | 0.001549 | SLC4A2 |
| -0.62241 | 0.001565 | INADL |
| -0.66049 | 0.001571 | LPHN3 |
| -0.60069 | 0.001624 | OSGEPL1 |
| -0.59885 | 0.001631 | ANXA8 /// LOC100996760 /// LOC101060462 |
| -0.59137 | 0.001672 | KLK10 |
| -0.59514 | 0.001689 | PDZD2 |
| -0.61937 | 0.001701 | POLI |
| -0.62978 | 0.001728 | ANP32A-IT1 |
| -0.77342 | 0.001769 | OTTHUMG00000179824 /// RP11-173M1.8 |
| -0.63285 | 0.001796 | LOC100128108 |
| -1.06761 | 0.0018 | ZNF404 |
| -0.58649 | 0.001813 | DYRK2 |
| -0.63983 | 0.001843 | CCDC89 |
| -0.86085 | 0.001862 | LOC100996653 |
| -0.61108 | 0.001928 | TRIM13 |
| -0.70744 | 0.001934 | CTD-3064H18.1 /// OTTHUMG00000181988 |
| -0.59137 | 0.001954 | RBL2 |
| -0.79764 | 0.001962 | SMIM17 |
| -0.60151 | 0.001984 | LOC100505501 |
| -0.65398 | 0.00201 | ZNF607 |
| -0.93025 | 0.002033 | NR2F2-AS1 |
| -0.64739 | 0.002058 | SLC35E2 |
| -0.8206 | 0.002088 | AP001462.6 /// OTTHUMG00000066836 |
| -0.67584 | 0.00209 | VSIG10L |
| -0.76449 | 0.002159 | LINC00521 |
| -0.7473 | 0.002326 | SETBP1 |
| -0.68633 | 0.002396 | CTD-2336O2.1 /// OTTHUMG00000163625 |
| -0.65765 | 0.002404 | OTTHUMG00000175832 /// RP11-274H2.5 |
| -0.94263 | 0.00244 | TET1 |
| -0.71461 | 0.002451 | FAM226A /// FAM226B |
| -0.83535 | 0.002499 | FLJ10038 |
| -0.60816 | 0.002523 | SNHG3 /// SNORA73A |
| -0.63917 | 0.002557 | FLJ39632 /// LOC100506303 /// LOC100653149 /// LOC101060483 /// LOC400879 /// LOC440157 |
| -0.77369 | 0.00267 | TRIM73 |
| -0.6151 | 0.002721 | H2AFB1 /// H2AFB2 /// H2AFB3 |
| -1.10222 | 0.002726 | LINC00115 |
| -0.58577 | 0.002727 | ATXN3 |
| -0.72067 | 0.002769 | SOX11 |
| -0.64473 | 0.003055 | OTTHUMG00000162817 /// RP11-549J18.1 |
| -0.78045 | 0.003089 | PAN3-AS1 |
| -0.59411 | 0.003147 | EFCAB7 |
| -0.94314 | 0.003157 | RNFT2 |
| -0.68467 | 0.003184 | PCDHB10 /// PCDHB9 |
| -0.64367 | 0.00319 | KIAA1967 |
| -0.74704 | 0.003264 | LOC153682 |
| -0.68184 | 0.003521 | SCG5 |
| -0.67347 | 0.003539 | USP48 |
| -0.66114 | 0.003564 | IRAK1BP1 |
| -0.75312 | 0.003605 | ZBTB20 |
| -0.74403 | 0.003678 | MINOS1P1 |
| -0.68711 | 0.003742 | FBXO9 |
| -0.59802 | 0.003745 | AC092620.2 /// OTTHUMG00000153633 |
| -0.66239 | 0.003747 | EXD2 |
| -0.60444 | 0.003872 | TCAIM |
| -0.72274 | 0.004055 | LRP2BP |
| -0.82953 | 0.004099 | GHR |
| -0.63219 | 0.0041 | FCF1 |
| -0.93611 | 0.004287 | OTTHUMG00000022286 /// RP3-327A19.5 |
| -0.58852 | 0.004449 | TMEM158 |
| -0.63609 | 0.004574 | ATF6B /// LOC101060681 /// TNXB |
| -0.71576 | 0.004634 | ZNF225 |
| -0.71133 | 0.00487 | PLGLB1 /// PLGLB2 |
| -0.64959 | 0.004973 | AGBL2 |
| -0.59246 | 0.004982 | NAGS |
| -0.63462 | 0.004998 | PLA2R1 |
| -0.59869 | 0.005013 | GSAP |
| -0.65919 | 0.005162 | GNB1L |
| -0.66922 | 0.005331 | EPHX2 |
| -1.1082 | 0.005346 | HIST1H2AB /// HIST1H2AE |
| -0.71081 | 0.00572 | ZNF224 |
| -0.78977 | 0.005741 | PMEPA1 |
| -0.60415 | 0.00627 | OTTHUMG00000176135 /// OTTHUMG00000179776 /// RP11-390B4.5 |
| -0.60867 | 0.006444 | LOC100130987 |
| -0.65733 | 0.006877 | TOX2 |
| -0.64063 | 0.00728 | AC004076.5 /// OTTHUMG00000183379 |
| -0.61933 | 0.007655 | SPATA17 |
| -0.63686 | 0.007939 | ZNF616 |
| -0.68125 | 0.008097 | RNF128 |
| -0.63886 | 0.00816 | BEX5 |
| -0.62003 | 0.008172 | ZBTB3 |
| -0.63176 | 0.008353 | KDM4C |
| -0.64475 | 0.00842 | MT1P3 |
| -0.73269 | 0.008445 | OLFML2B |
| -0.76206 | 0.008787 | OTTHUMG00000176822 /// RP11-846E15.3 |
| -0.66343 | 0.008847 | GUSBP3 /// GUSBP9 /// SMA4 /// SMA5 |
| -1.16146 | 0.008963 | LOX |
| -0.62908 | 0.009375 | DPP7 |
| -0.69475 | 0.010056 | FAM149B1 |
| -0.61016 | 0.010166 | MDFIC |
| -0.59703 | 0.010354 | PIK3C3 |
| -0.60762 | 0.010804 | CDC25C |
| -0.70097 | 0.011167 | CDH12 |
| -0.65966 | 0.011268 | GPR155 |
| -0.83985 | 0.011432 | LINC00842 |
| -0.80729 | 0.011473 | LINC00622 |
| -0.59277 | 0.011893 | RBM4 |
| -0.88786 | 0.012447 | LOC286272 |
| -0.81483 | 0.012896 | PCDHB14 |
| -0.62845 | 0.013142 | CTC-204F22.1 /// OTTHUMG00000175772 |
| -0.71178 | 0.015177 | MAF |
| -0.6388 | 0.016679 | IPW /// LOC100506948 /// SNORD107 /// SNORD115-13 /// SNORD115-26 /// SNORD115-7 /// SNORD116-28 |
| -0.62116 | 0.021109 | OTTHUMG00000180633 /// RP11-384O8.1 |
| -0.62152 | 0.023179 | MGAT3 |
| -0.66069 | 0.025313 | ZNF285 |
| -0.63634 | 0.025778 | FBXL21 |
