# Supplementary material for: p53 modeling as a route to mesothelioma patients stratification and novel therapeutic identification
Source: J Transl Med. 2018 Oct 13;16:282. doi: 10.1186/s12967-018-1650-0 (PMC6186085; doi:10.1186/s12967-018-1650-0)
Supplement: Supplementary file 7 — Additional file 7: Table S7. Mero-14_STSFA_results. [file 12967_2018_1650_MOESM7_ESM.docx]

**Table S7:** Mero14_STSFA_results

| **Gene** | **Control_1** | **Control_2** | **Gem_1** | **Gem_2** | **Etop_1** | **Etop_2** |
| --- | --- | --- | --- | --- | --- | --- |
| AATF | 1387 | 1388 | 1375 | 1359 | 1371 | 1375 |
| ABCB1 | 603 | 604 | 654 | 641 | 658 | 653 |
| ABCC1 | 992 | 992 | 951 | 943 | 916 | 904 |
| AIFM2 | 824 | 821 | 817 | 809 | 811 | 824 |
| APAF1 | 773 | 758 | 721 | 743 | 713 | 751 |
| AR | 245 | 241 | 243 | 238 | 235 | 241 |
| ARID3A | 634 | 644 | 594 | 614 | 608 | 598 |
| ATF3 | 645 | 639 | 919 | 921 | 904 | 882 |
| ATM | 610 | 611 | 594 | 595 | 596 | 587 |
| ATR | 800 | 799 | 851 | 866 | 869 | 859 |
| AURKA | 971 | 962 | 885 | 889 | 845 | 869 |
| AXIN1 | 698 | 702 | 669 | 658 | 703 | 690 |
| Apoptosis | 14745 | 14716 | 14191 | 14212 | 14643 | 14224 |
| BAK1 | 665 | 646 | 691 | 681 | 674 | 678 |
| BAX | 3415 | 3351 | 3016 | 3011 | 3151 | 2949 |
| BBC3 | 236 | 220 | 235 | 236 | 222 | 224 |
| BCL2 | 153 | 124 | 253 | 237 | 282 | 263 |
| BCL3 | 492 | 488 | 503 | 498 | 475 | 471 |
| BCL6 | 696 | 684 | 655 | 675 | 688 | 681 |
| BDKRB1 | 585 | 589 | 609 | 622 | 587 | 577 |
| BNIP3L | 1099 | 1101 | 1004 | 1021 | 1053 | 1009 |
| BRCA1 | 1433 | 1434 | 1522 | 1531 | 1542 | 1565 |
| BTG2 | 502 | 493 | 577 | 582 | 544 | 535 |
| CALD1 | 610 | 585 | 575 | 584 | 572 | 558 |
| CASP8 | 566 | 565 | 488 | 497 | 535 | 562 |
| CCNA2 | 0 | 0 | 0 | 0 | 0 | 0 |
| CCNB1 | 743 | 706 | 590 | 580 | 459 | 539 |
| CCND1 | 3009 | 3072 | 3151 | 3157 | 2937 | 2974 |
| CCNG1 | 1338 | 1341 | 1327 | 1327 | 1324 | 1346 |
| CD44 | 1151 | 1115 | 1097 | 1170 | 1275 | 1254 |
| CD58 | 787 | 786 | 789 | 801 | 824 | 812 |
| CD59 | 938 | 929 | 922 | 928 | 928 | 902 |
| CD82 | 819 | 813 | 807 | 810 | 783 | 788 |
| CDC20 | 820 | 809 | 730 | 749 | 747 | 755 |
| CDC25A | 247 | 250 | 347 | 360 | 337 | 353 |
| CDK2 | 0 | 0 | 0 | 0 | 0 | 0 |
| CDK4 | 1185 | 1183 | 1224 | 1226 | 1248 | 1265 |
| CDK5 | 1647 | 1637 | 1578 | 1601 | 1552 | 1582 |
| CDK9 | 513 | 518 | 506 | 486 | 502 | 500 |
| CDKN1A | 749 | 729 | 1017 | 1027 | 1022 | 1011 |
| CDKN1B | 968 | 1021 | 926 | 892 | 814 | 833 |
| CDKN2A | 330 | 326 | 275 | 321 | 319 | 303 |
| CHEK1 | 724 | 733 | 735 | 734 | 748 | 756 |
| CHEK2 | 1121 | 1093 | 1105 | 1114 | 1097 | 1105 |
| CIAPIN1 | 863 | 844 | 872 | 877 | 863 | 868 |
| CKB | 829 | 821 | 842 | 849 | 835 | 840 |
| CKM | 293 | 285 | 298 | 280 | 289 | 280 |
| CKS2 | 1147 | 1145 | 1129 | 1133 | 1124 | 1124 |
| COL18A1 | 865 | 864 | 799 | 800 | 795 | 789 |
| CSNK2A2 | 866 | 857 | 892 | 894 | 897 | 889 |
| CXCR4 | 837 | 878 | 794 | 924 | 864 | 718 |
| Cellularsenescence | -10437 | -10392 | -10130 | -10136 | -9972 | -10093 |
| DDB2 | 711 | 698 | 728 | 735 | 776 | 765 |
| DDIT4 | 1138 | 1132 | 993 | 995 | 1037 | 1021 |
| DDX5 | 728 | 720 | 741 | 756 | 762 | 724 |
| DFNA5 | 0 | 0 | 0 | 0 | 0 | 0 |
| DKK1 | 858 | 866 | 1096 | 1085 | 1184 | 1150 |
| DNAdamage | 0 | 0 | 0 | 0 | 0 | 0 |
| DUSP2 | 465 | 497 | 578 | 596 | 573 | 565 |
| DUSP4 | 683 | 693 | 738 | 742 | 739 | 735 |
| DUSP5 | 800 | 825 | 1092 | 1090 | 949 | 953 |
| DYRK2 | 705 | 710 | 630 | 652 | 684 | 674 |
| E2F1 | 2067 | 2048 | 2193 | 2142 | 2086 | 2135 |
| ECT2 | 583 | 584 | 606 | 614 | 617 | 624 |
| EDA2R | 479 | 451 | 485 | 471 | 464 | 466 |
| EGFR | 1223 | 1272 | 1134 | 1084 | 1008 | 1061 |
| EIF2AK2 | 844 | 843 | 840 | 859 | 893 | 875 |
| ELAVL1 | 683 | 686 | 678 | 691 | 686 | 689 |
| EPHB4 | 497 | 502 | 488 | 488 | 475 | 485 |
| ERBB2 | 573 | 540 | 551 | 558 | 515 | 554 |
| ESR1 | 660 | 683 | 657 | 648 | 627 | 646 |
| EZH2 | 1679 | 1685 | 1822 | 1826 | 1831 | 1840 |
| FAS | 853 | 864 | 925 | 918 | 937 | 934 |
| FDXR | 466 | 425 | 433 | 427 | 452 | 462 |
| FEN1 | 969 | 974 | 1078 | 1066 | 1077 | 1085 |
| FGF2 | 942 | 943 | 1018 | 1033 | 1088 | 1076 |
| FHL2 | 711 | 715 | 811 | 809 | 757 | 749 |
| FOS | 383 | 350 | 465 | 500 | 426 | 318 |
| FOXM1 | 841 | 827 | 848 | 850 | 857 | 880 |
| GADD45A | 712 | 709 | 956 | 953 | 950 | 936 |
| GAPDH | 1417 | 1415 | 1395 | 1392 | 1383 | 1383 |
| GSTP1 | 1266 | 1263 | 1255 | 1255 | 1238 | 1269 |
| GTSE1 | 784 | 781 | 788 | 788 | 765 | 784 |
| H2AFZ | 1218 | 1222 | 1263 | 1254 | 1255 | 1262 |
| HDAC1 | 1090 | 1083 | 1064 | 1059 | 1065 | 1062 |
| HIC1 | 487 | 490 | 516 | 489 | 510 | 475 |
| HIF1A | 3027 | 3031 | 3139 | 3128 | 3123 | 3105 |
| HIPK2 | 489 | 494 | 493 | 506 | 508 | 506 |
| HIPK4 | 584 | 597 | 602 | 573 | 562 | 593 |
| HMMR | 913 | 908 | 855 | 865 | 858 | 869 |
| HNF4A | 347 | 337 | 349 | 331 | 349 | 331 |
| HOXA11 | 375 | 379 | 403 | 396 | 409 | 429 |
| HSP90AB1 | 1000 | 1006 | 990 | 985 | 993 | 1002 |
| HSPA4 | 2856 | 2887 | 2907 | 2911 | 2821 | 2828 |
| HTATIP2 | 421 | 397 | 411 | 420 | 412 | 413 |
| ICAM1 | 725 | 727 | 753 | 762 | 728 | 710 |
| ID3 | 719 | 701 | 733 | 758 | 797 | 787 |
| IER3 | 1038 | 1030 | 1057 | 1085 | 1098 | 1050 |
| IFI16 | 1014 | 1017 | 1071 | 1073 | 1117 | 1099 |
| IFITM2 | 1036 | 1035 | 1030 | 1031 | 1048 | 1027 |
| IFNA1 | 0 | 0 | 0 | 0 | 0 | 0 |
| IGF1R | 597 | 624 | 563 | 599 | 570 | 560 |
| IGFBP1 | 256 | 242 | 235 | 246 | 237 | 240 |
| IGFBP7 | 765 | 756 | 770 | 797 | 811 | 732 |
| IL6 | 725 | 728 | 865 | 850 | 864 | 864 |
| IQCB1 | 757 | 764 | 819 | 819 | 812 | 800 |
| ISG15 | 1311 | 1305 | 1341 | 1343 | 1383 | 1337 |
| KAT2B | 930 | 939 | 930 | 920 | 894 | 894 |
| KLF4 | 491 | 533 | 576 | 589 | 685 | 643 |
| KRT19 | 648 | 653 | 582 | 603 | 582 | 585 |
| KRT8 | 1256 | 1255 | 1189 | 1175 | 1152 | 1176 |
| LATS2 | 698 | 702 | 745 | 743 | 732 | 728 |
| LTF | 434 | 424 | 451 | 408 | 405 | 426 |
| MAP4 | 645 | 652 | 621 | 630 | 628 | 610 |
| MAP4K4 | 776 | 786 | 789 | 798 | 764 | 738 |
| MAPK1 | 0 | 0 | 0 | 0 | 0 | 0 |
| MAPK14 | 433 | 429 | 381 | 381 | 382 | 379 |
| MAPK8 | 592 | 604 | 606 | 631 | 625 | 609 |
| MAPK9 | 770 | 756 | 769 | 764 | 758 | 744 |
| MCL1 | 571 | 549 | 626 | 668 | 707 | 679 |
| MCTS1 | 630 | 638 | 607 | 618 | 620 | 582 |
| MDM2 | 247 | 251 | 255 | 287 | 286 | 271 |
| MDM4 | 351 | 341 | 356 | 389 | 396 | 384 |
| MGMT | 798 | 791 | 764 | 772 | 744 | 729 |
| MMP1 | 1121 | 1064 | 1213 | 1313 | 1401 | 1158 |
| MMP13 | 0 | 0 | 0 | 0 | 0 | 0 |
| MMP2 | 2757 | 2698 | 2396 | 2397 | 2520 | 2331 |
| MSH2 | 1118 | 1106 | 1181 | 1161 | 1170 | 1199 |
| MUC1 | 436 | 439 | 438 | 429 | 411 | 420 |
| MYC | 3144 | 3164 | 3111 | 3097 | 3055 | 3108 |
| MYCN | 692 | 689 | 709 | 701 | 640 | 694 |
| NCL | 267 | 248 | 266 | 268 | 253 | 255 |
| NLRC4 | 236 | 242 | 257 | 244 | 243 | 249 |
| NME1 | 1434 | 1418 | 1390 | 1387 | 1383 | 1401 |
| NOTCH1 | 593 | 574 | 616 | 608 | 618 | 632 |
| NOV | 332 | 327 | 353 | 361 | 373 | 378 |
| NR2C1 | 534 | 533 | 509 | 537 | 523 | 529 |
| NTN1 | 346 | 370 | 373 | 335 | 337 | 394 |
| PADI4 | 267 | 266 | 281 | 278 | 299 | 299 |
| PARK2 | 243 | 225 | 229 | 231 | 235 | 240 |
| PCBP4 | 617 | 608 | 624 | 639 | 602 | 607 |
| PCNA | 0 | 0 | 0 | 0 | 0 | 0 |
| PDGFRB | 693 | 684 | 626 | 635 | 615 | 576 |
| PDRG1 | 830 | 824 | 908 | 913 | 921 | 916 |
| PEG3 | 244 | 240 | 258 | 236 | 247 | 247 |
| PERP | 463 | 433 | 417 | 415 | 411 | 415 |
| PIDD1 | 576 | 577 | 653 | 650 | 648 | 635 |
| PLAUR | 1247 | 1233 | 1301 | 1284 | 1356 | 1339 |
| POU4F1 | 188 | 180 | 191 | 181 | 196 | 204 |
| PPM1A | 737 | 739 | 714 | 732 | 751 | 748 |
| PPM1D | 645 | 632 | 686 | 708 | 695 | 680 |
| PRC1 | 984 | 976 | 966 | 968 | 947 | 963 |
| PRKCA | 508 | 496 | 539 | 486 | 502 | 507 |
| PRKD1 | 456 | 462 | 458 | 479 | 454 | 435 |
| PRKDC | 840 | 838 | 852 | 842 | 833 | 846 |
| PRKG1 | 337 | 339 | 346 | 359 | 358 | 354 |
| PRSS50 | 391 | 396 | 433 | 382 | 390 | 421 |
| PSEN1 | 420 | 469 | 444 | 435 | 465 | 485 |
| PSMD10 | 1036 | 1035 | 992 | 993 | 997 | 994 |
| PTEN | 590 | 593 | 548 | 561 | 575 | 565 |
| PTGS2 | 2516 | 2413 | 2780 | 2928 | 3345 | 3182 |
| PTTG1 | 1063 | 1049 | 935 | 938 | 941 | 948 |
| RAD51 | 0 | 0 | 0 | 0 | 0 | 0 |
| RAF1 | 1023 | 1028 | 982 | 990 | 986 | 996 |
| RAS | 1105 | 1125 | 1096 | 1116 | 1115 | 1130 |
| RECQL4 | 642 | 642 | 686 | 693 | 690 | 706 |
| RGCC | 275 | 270 | 266 | 248 | 265 | 265 |
| RGS16 | 413 | 405 | 442 | 430 | 439 | 446 |
| RPRM | 869 | 859 | 808 | 809 | 832 | 814 |
| RREB1 | 324 | 330 | 361 | 298 | 304 | 329 |
| RRM2B | 754 | 745 | 744 | 751 | 774 | 742 |
| S100A2 | 661 | 654 | 685 | 718 | 745 | 700 |
| S100A6 | 886 | 870 | 865 | 880 | 876 | 875 |
| SEMA3B | 480 | 474 | 493 | 505 | 469 | 483 |
| SERPINB5 | 242 | 254 | 259 | 245 | 232 | 267 |
| SERPINF1 | 343 | 350 | 349 | 339 | 309 | 325 |
| SESN2 | 588 | 581 | 708 | 703 | 754 | 767 |
| SFN | 488 | 474 | 532 | 537 | 498 | 494 |
| SGK1 | 1181 | 1182 | 1237 | 1248 | 1261 | 1273 |
| SIAH1 | 2416 | 2422 | 2456 | 2440 | 2456 | 2436 |
| SIVA1 | 1238 | 1215 | 1292 | 1277 | 1251 | 1281 |
| SLC2A1 | 1129 | 1120 | 974 | 994 | 1038 | 946 |
| SLC2A4 | 307 | 310 | 319 | 315 | 318 | 327 |
| SLC6A6 | 367 | 359 | 358 | 366 | 346 | 378 |
| SOX4 | 776 | 771 | 710 | 738 | 799 | 765 |
| SP7 | 323 | 294 | 313 | 327 | 319 | 318 |
| TCF7L2 | 507 | 504 | 528 | 529 | 504 | 509 |
| TFDP1 | 618 | 627 | 645 | 653 | 658 | 658 |
| TGFA | 348 | 355 | 381 | 357 | 326 | 392 |
| TGFB1 | 1234 | 1248 | 1452 | 1457 | 1544 | 1510 |
| THBS1 | 0 | 0 | 0 | 0 | 0 | 0 |
| TIAF1 | 371 | 386 | 413 | 378 | 349 | 394 |
| TIGAR | 236 | 220 | 235 | 236 | 222 | 224 |
| TLR3 | 408 | 393 | 434 | 450 | 527 | 500 |
| TNFRSF10A | 959 | 961 | 931 | 946 | 990 | 985 |
| TNFRSF10B | 1060 | 1094 | 1093 | 1172 | 1162 | 1071 |
| TP53 | 4162 | 4136 | 4404 | 4219 | 4276 | 4174 |
| TP53AIP1 | 792 | 830 | 758 | 750 | 773 | 757 |
| TP53I13 | 513 | 507 | 508 | 504 | 507 | 517 |
| TP53INP1 | 421 | 433 | 453 | 425 | 434 | 427 |
| VEGFA | 1733 | 1702 | 1782 | 1803 | 1974 | 1870 |
| VRK1 | 797 | 793 | 880 | 882 | 862 | 860 |
| WWP1 | 700 | 699 | 662 | 686 | 691 | 668 |
| XAF1 | 758 | 763 | 781 | 790 | 864 | 841 |
| YBX1 | 1137 | 1137 | 1157 | 1153 | 1155 | 1170 |
| ZMAT3 | 500 | 512 | 500 | 528 | 526 | 521 |
