# Supplementary material for: p53 modeling as a route to mesothelioma patients stratification and novel therapeutic identification
Source: J Transl Med. 2018 Oct 13;16:282. doi: 10.1186/s12967-018-1650-0 (PMC6186085; doi:10.1186/s12967-018-1650-0)
Supplement: Supplementary file 10 — Additional file 10: Table S10. KEGG pathways. [file 12967_2018_1650_MOESM10_ESM.docx]

**Table S10 KEGG pathways**

| **TP53 WT T patients** | | **TP53 WT UT patients** | | **TP53 Mut UT patients** | |  |
| --- | --- | --- | --- | --- | --- | --- |
| **ID** | **Gene Name** | **ID** | **Gene Name** | **ID** | **Gene Name** | **KEGG_PATHWAY** |
| CKS2 | CDC28 protein kinase regulatory subunit 2(CKS2) |  |  |  |  | Pathways in cancer, Small cell lung cancer |
| E2F1 | E2F transcription factor 1(E2F1) |  |  | E2F1 | E2F transcription factor 1(E2F1) | Cell cycle, Pathways in cancer, MicroRNAs in cancer |
| AURKA | aurora kinase A(AURKA) |  |  | AURKA | aurora kinase A(AURKA) | Oocyte meiosis |
| CHEK1 | checkpoint kinase 1(CHEK1) |  |  |  |  | Cell cycle, p53 signaling pathway, Viral carcinogenesis |
| CKB | creatine kinase B(CKB) |  |  |  |  | Arginine and proline metabolism, Metabolic pathways |
| FEN1 | flap structure-specific endonuclease 1(FEN1) |  |  |  |  | DNA replication, Base excision repair, Non-homologous end-joining |
| HSP90AB1 | heat shock protein 90 alpha family class B member 1(HSP90AB1) |  |  | HSP90AB1 | heat shock protein 90 alpha family class B member 1(HSP90AB1) | Protein processing in endoplasmic reticulum, PI3K-Akt signaling pathway, Antigen processing and presentation, Estrogen signaling pathway, Pathways in cancer |
| HMMR | hyaluronan mediated motility receptor(HMMR) |  |  |  |  | ECM-receptor interaction |
| PTTG1 | pituitary tumor-transforming 1(PTTG1) |  |  |  |  | Cell cycle, Oocyte meiosis, HTLV-I infection |
| SFN | Stratifin (SFN) |  |  |  |  | Cell cycle, p53 signaling pathway |
|  |  | DDIT4 | DNA damage inducible transcript 4(DDIT4) |  |  | mTOR signaling pathway, PI3K-Akt signaling pathway, MicroRNAs in cancer |
|  |  | MMP2 | matrix metallopeptidase 2(MMP2) |  |  | Leukocyte transendothelial migration, GnRH signaling pathway, Estrogen signaling pathway, Pathways in cancer, Proteoglycans in cancer |
|  |  |  |  | BRCA1 | BRCA1, DNA repair associated(BRCA1) | Fanconi anemia pathway, Ubiquitin mediated proteolysis, PI3K-Akt signaling pathway, MicroRNAs in cancer |
|  |  |  |  | NLRC4 | NLR family CARD domain containing 4(NLRC4) | NOD-like receptor signaling pathway, Salmonella infection, Legionellosis |
|  |  |  |  | APAF1 | apoptotic peptidase activating factor 1(APAF1) | p53 signaling pathway, Apoptosis, Alzheimer's disease, Parkinson's disease, Amyotrophic lateral sclerosis (ALS), Huntington's disease Small cell lung cancer |
|  |  |  |  | CDC20 | cell division cycle 20(CDC20) | Cell cycle, Oocyte meiosis, Ubiquitin mediated proteolysis, Viral carcinogenesis |
|  |  |  |  | EZH2 | enhancer of zeste 2 polycomb repressive complex 2 subunit(EZH2) | MicroRNAs in cancer |
|  |  |  |  | GAPDH | glyceraldehyde-3-phosphate dehydrogenase(GAPDH) | Glycolysis / Gluconeogenesis, Metabolic pathways, HIF-1 signaling pathway, Alzheimer's disease |
|  |  |  |  | HIF1A | hypoxia inducible factor 1 alpha subunit(HIF1A) | HIF-1 signaling pathway, Thyroid hormone signaling pathway, Pathways in cancer, Renal cell carcinoma, Central carbon metabolism in cancer |
|  |  |  |  | MAPK14 | mitogen-activated protein kinase 14(MAPK14) | MAPK signaling pathway, FoxO signaling pathway, VEGF signaling pathway, Signaling pathways regulating pluripotency of stem cells, NOD-like receptor signaling pathway, T cell receptor signaling pathway, TNF signaling pathway |
|  |  |  |  | NCL | nucleolin(NCL) | Pathogenic Escherichia coli infection |
|  |  |  |  | PLAUR | plasminogen activator, urokinase receptor(PLAUR) | Complement and coagulation cascades, Proteoglycans in cancer, |
|  |  |  |  | SIAH1 | siah E3 ubiquitin protein ligase 1(SIAH1) | p53 signaling pathway, Ubiquitin mediated proteolysis, Wnt signaling pathway |
