# Supplementary material for: p53 modeling as a route to mesothelioma patients stratification and novel therapeutic identification
Source: J Transl Med. 2018 Oct 13;16:282. doi: 10.1186/s12967-018-1650-0 (PMC6186085; doi:10.1186/s12967-018-1650-0)
Supplement: Supplementary file 11 — Additional file 11: Table S11. Approved drugs that target FEN1, MMP2 and SIAH1 indirectly and experimental drugs that target FEN1, MMP2 and SIAH1 directly or indirectly (DRUGSURV database). [file 12967_2018_1650_MOESM11_ESM.docx]

**Table S11:** Approved drugs that target FEN1, MMP2 and SIAH1 indirectly and experimental drugs that target FEN1, MMP2 and SIAH1 directly or indirectly (DRUGSURV database)

| **Drugs (*experimental*) which target FEN1 directly** |  |
| --- | --- |
| **GENE** | **Drug-Target Details** |
| FEN1 | DB08468 |
|  | DB08167 |
|  | Myricetin |
|  | DB03374 |
|  | DB02232 |
|  | PICEATANNOL |
|  | DB03167 |
|  | DB02285 |
|  | Quercetin |
|  | DB08238 |
|  | Dequadin |
|  | DB07715 |
| **Drugs (*aproved*) which target FEN1 indirectly** |  |
| **en1->Interaction partner** | **Drugs which Target interaction partner of fen1** |
| fen1 -> BLM | Methyldopa |
|  | Fluorescein |
|  | Daunorubicin |
|  | Idarubicin |
|  | Trimetrexate |
|  | Norepinephrine |
|  | Menadione |
|  | Bexarotene |
|  | Mitoxantrone |
| fen1 -> WRN | Cefalotin |
|  | Alpha-Linolenic Acid |
|  | Mitoxantrone |
|  | Levodopa |
| **Drugs (*experimental*) which target FEN1 indirectly** |  |
| **fen1->Interaction partner** | **Drugs which Target interaction partner of fen1** |
| fen1 -> WRN | DB07534 |
|  | Myricetin |
|  | DB02285 |
| fen1 -> BLM | DB08468 |
|  | Myricetin |
|  | DB08167 |
|  | Propidium |
|  | Quercetin |
| **Drugs (*experimental*) which target MMP2 directly** |  |
| **GENE** | **Drug-Target Details** |
| MMP2 | DB08030 |
|  | DB07713 |
|  | DB08489 |
|  | DB07988 |
|  | Batimastat |
|  | DB07534 |
|  | DB04140 |
|  | DB07145 |
|  | DB07013 |
|  | DB03368 |
|  | DB06945 |
|  | DB07772 |
|  | DB07290 |
|  | DB08490 |
|  | DB08403 |
|  | DB07964 |
|  | DB07390 |
|  | SC-74020 |
|  | DB08493 |
|  | GM6001 |
|  | DB08507 |
|  | DB07926 |
|  | DB06837 |
| **Drugs (*approved*) which target MMP2 indirectly** |  |
| **mmp2->Interaction partner** | **Drugs which Target interaction partner of mmp2** |
| mmp2 -> HSP90AA1 | Clotrimazole |
|  | Fluconazole |
|  | Disulfiram |
|  | Clioquinol |
|  | Chloroxine |
|  | Hexachlorophene |
|  | Nedocromil |
|  | Rifabutin |
|  | Nitroxoline |
|  | Terconazole |
|  | Miconazole |
|  | Bifonazole |
| **Drugs (*experimental*) which target MMP2 indirectly** |  |
| **mmp2->Interaction partner** | **Drugs which Target interaction partner of mmp2** |
| mmp2 -> HSP90AA1 | DB08789 |
|  | DB08788 |
|  | DB07877 |
|  | DB08356 |
|  | DB07324 |
|  | DB04254 |
|  | DB06961 |
|  | DB03093 |
|  | DB08443 |
|  | DB02550 |
|  | DB02365 |
|  | DB02840 |
|  | DB08442 |
|  | DB07495 |
|  | 17-Dmag |
|  | DB07594 |
|  | DB06957 |
|  | DB07100 |
|  | DB04505 |
|  | DB03809 |
|  | DB06969 |
|  | DB06956 |
|  | DB06937 |
|  | DB04588 |
|  | DB08194 |
|  | DB02909 |
|  | DB04054 |
|  | DB07319 |
|  | DB07615 |
|  | Geldanamycin |
|  | DB08059 |
|  | DB03431 |
|  | DB07601 |
|  | DB07407 |
|  | DB08786 |
|  | DB08197 |
|  | DB02359 |
|  | DB02754 |
|  | TRICLOSAN |
|  | DB06958 |
|  | DB03749 |
|  | DB07502 |
|  | DB07317 |
|  | DB08557 |
|  | DB07325 |
|  | DB08436 |
|  | DB06964 |
|  | DB03504 |
|  | DB08787 |
|  | DB03899 |
|  | DB03137 |
| **Drugs (*approved*) which target SIAH1 indirectly** |  |
| **siah1->Interaction partner** | **Drugs which Target interaction partner of siah1** |
| siah1 -> UBE2N | Perphenazine |
|  | Astemizole |
|  | Terfenadine |
|  | Ifosfamide |
|  | Tamoxifen |
|  | Prochlorperazine |
|  | Thiothixene |
| siah1 -> STAT3 | Podofilox |
|  | Ouabain |
|  | Hexachlorophene |
|  | Celecoxib |
|  | Niclosamide |
|  | Digitoxin |
| siah1 -> TNK2 | DB00171 |
|  | Sunitinib |
|  | Dasatinib |
| **Drugs (*experimental*) which target SIAH1 indirectly** |  |
| **siah1->Interaction partner** | **Drugs which Target interaction partner of siah1** |
| siah1 -> XIAP | DB02628 |
|  | DB04612 |
| siah1 -> UBE2N | DB08567 |
|  | Resveratrol |
| siah1 -> TNK2 | DB06999 |
|  | DB04367 |
|  | PD173955 |
|  | DB07159 |
| siah1 -> STAT3 | DB07614 |
| siah1 -> UBE2D2 | DB02418 |
