# Supplementary material for: p53 modeling as a route to mesothelioma patients stratification and novel therapeutic identification
Source: J Transl Med. 2018 Oct 13;16:282. doi: 10.1186/s12967-018-1650-0 (PMC6186085; doi:10.1186/s12967-018-1650-0)
Supplement: Supplementary file 12 — Additional file 12: Table S12. Stage of patients. [file 12967_2018_1650_MOESM12_ESM.docx]

**Table S12:** Stage of patients

| **ID** | **Survival** | **Age** | **Stage** | **treatment** |
| --- | --- | --- | --- | --- |
| 617PT | 0.64 | 63.3 | ypT2N1Mx | 1 |
| 618PT | 1.59 | 72.3 | ypT3N2Mx | 1 |
| 634PT | 4.67 | 56 | ypT3N0Mx | 1 |
| 655PT | 1.49 | 79.2 | ypT3N0Mx | 1 |
| M101PT | 0.67 | 62.1 | ypT4_N2_(7th) | 1 |
| M614PT | 0.64 | 62 | ypT4N1Mx | 1 |
| M626PT | 1.22 | 72.8 | ypT2N2Mx | 1 |
| M632PT | 0.28 | 58.9 | ypT4N2Mx | 1 |
| M637PT | 3.89 | 44.1 | ypT2N0Mx | 1 |
| M640PT | 0.77 | 72.1 | ypT3N2Mx | 1 |
| M645PT | 1.26 | 67.7 | ypT1N0Mx | 1 |
| M663PT | 1.11 | 61.1 | ypT4N2 | 1 |
| M664PT | 2.1 | 76.9 | ypT4N0Mx | 1 |
| M666PT | 2.13 | 62.7 | ypT4N0Mx | 1 |
| M668PT | 0.47 | 61.7 | ypT3N1 | 1 |
| M669PT | 2.05 | 58.7 | ypT4N1 | 1 |
| M684PT | 1.39 | 67.4 | ypT3N1 | 1 |
| M685PT | 0.55 | 74.4 | ypT3N1Mx | 1 |
| M686PT | 0.36 | 64.2 | ypT2N0Mx | 1 |
| M687PT | 2.63 | 82.1 | ypT3N0Mx | 1 |
| M689PT | 0.37 | 72.8 | ypT1N2Mx | 1 |
| M690PT | 0.53 | 65.7 | ypT4N2Mx | 1 |
| M694PT | 2.51 | 34.4 | ypT2N2Mx | 1 |
| M703PT | 0.54 | 77 | ypT3N2 | 1 |
| M705PT | 0.17 | 75.3 | ypT2N0Mx | 1 |
| M714PT | 0.16 | 73.1 | ypT3N2Mx | 1 |
| M719PT | 0.34 | 73.7 | ypT3N2Mx | 1 |
| M47PT | 1.57 | 84.5 | NA | 0 |
| M48PT | 1.65 | 69.1 | T4_N0_MX_(6th) | 0 |
| M52PT | 0.74 | 47.8 | T3_N0_MX_(6th) | 0 |
| M53PT | 0.63 | 63.2 | pT4_N3_MX_(6th) | 0 |
| M58PT | 0.25 | 73.3 | T4_N0_MX (6th) | 0 |
| M608PT | 2.76 | 60.1 | pT4N2Mx | 0 |
| M63PT | 0.52 | 66.5 | NA | 0 |
| M67PT | 2.29 | 52.8 | T3_N0_MX_(6th) | 0 |
| M68PT | 5.01 | 38.3 | T3_N2_MX (6th) | 0 |
| M691PT | 0.39 | 69.7 | pT2N0Mx | 0 |
| M697PT | 0.47 | 69.5 | pT3N2Mx | 0 |
| M699PT | 1.47 | 68.9 | pT3N1 | 0 |
| M6PT | 0.65 | 70.5 | pT2_N0_(7th) | 0 |
| M700PT | 0.77 | 45.5 | ypT3N0 | 0 |
| M701PT | 1.18 | 78 | pT3N0Mx | 0 |
| M704PT | 1.81 | 73.2 | pT3N0Mx | 0 |
| M70PT | 1.42 | 68.7 | T2_N0_Mx_(6th) | 0 |
| M710PT | 1.09 | 51.9 | pT2N0Mx | 0 |
| M71PT | 2.78 | 74.5 | T4_N2_MX_(6th) | 0 |
| M73PT | 3.79 | 56.8 | NA | 0 |
| M75PT | 11.05 | 62.3 | T2_N1_MX_(6th) | 0 |
| M76PT | 1.22 | 66.4 | T4_N0_MX_(6th) | 0 |
| M80PT | 0.28 | 71.5 | chest_wall_invasion | 0 |
| M82PT | 2.22 | 67.8 | positive_level8_nodes | 0 |
| M94PT | 3.83 | 60.8 | pT3_N1_Mx_(7th) | 0 |
| M98PT | 2.05 | 65.2 | pT4_N1_(7th) | 0 |
| 649PT | 0.58 | 53.8 | ypT3N1Mx | 1 |
| 602PT | 2.28 | 66.9 | T3N0Mx (7th) | 0 |
| 667PT | 0.25 | 54.3 | pT3N0Mx | 0 |
| M17PT | 0.65 | 69.6 | NA | 0 |
| M20PT | 0.21 | 67.2 | T2_N2_MX_(6th) | 0 |
| M37PT | 0.73 | 65 | T3_N2_MX_(6th) | 0 |
| M43PT | 0.42 | 82.3 | NA | 0 |
| M49PT | 0.34 | 84.2 | yT3_N0_MX_(6th) | 0 |
| M50PT | 2.04 | 61.4 | T2_N2_MX_(6th) | 0 |
| M57PT | 0.81 | 69 | T3_N2_MX_(6th) | 0 |
| M60PT | 0.68 | 54.1 | T4_N1_MX_(6th) | 0 |
| M61PT | 1.59 | 60.2 | T3_N2_M0_(6th) | 0 |
| M62PT | 5.02 | 43.2 | NA | 0 |
| M69PT | 1.69 | 78 | T3_N2_MX_(6th) | 0 |
| M77PT | 0.95 | 59.7 | "extensive_chest_wall_invasion" | 0 |
| M8PT | 0.82 | 59.2 | T2_N0_MX_(6th) | 0 |
| M97PT | 0.26 | 68.2 | pT3_N0_(7th) | 0 |
| M99PT | 0.29 | 57.7 | T3_N0_Mx_(7th) | 0 |
