# Supplementary material for: p53 modeling as a route to mesothelioma patients stratification and novel therapeutic identification
Source: J Transl Med. 2018 Oct 13;16:282. doi: 10.1186/s12967-018-1650-0 (PMC6186085; doi:10.1186/s12967-018-1650-0)
Supplement: Supplementary file 15 — Additional file 15: Table S14. Signaling pathways controlled by genes correlated with tumor stages. [file 12967_2018_1650_MOESM15_ESM.docx]

**Table S14: Signaling pathways controlled by genes correlated with tumor stages**

| **ID** | **Gene Name** | **KEGG_PATHWAY** | **BBID_PATHWAY** | **BIOCARTA_PATHWAY** |
| --- | --- | --- | --- | --- |
| KAT2B | K(lysine) acetyltransferase 2B | Notch signaling pathway | Nuclear Receptor Coactivator Complex | hpcaf pathway  hrarrxr pathway  h_ vdr pathway |
| CDKN1B | cyclin-dependent kinase inhibitor 1B (p27, Kip1) | ErbB signaling pathway, Cell cycle, Pathways in cancer | RB phosphor E2F, cyclin-CDK_complexes, cyclins & p27_cell cycle, G1-phase progression by Myc, Ubiquitination Pathways Cell Cycle | hcell cycle pathway  hctcf pathway  hg1 pathway  hmcm pathway  hp27 pathway  hpten pathway  hRac CycD pathway |
| MAP4K4 | mitogen-activated protein kinase kinase kinase kinase 4 | MAPK signaling pathway |  | hmapk pathway |
| RRM2B | ribonucleotide reductase M2 B (TP53 inducible) | Purine metabolism, Pyrimidine metabolism, Glutathione metabolism, p53 signaling pathway |  |  |
| SLC2A4 | solute carrier family 2 (facilitated glucose transporter), member 4 | Insulin signaling pathway, Adipocytokine signaling pathway, Type II diabetes mellitus |  | hgh pathway  h_insulin pathway  hpgc1a pathway |
| ZMAT3 | zinc finger, matrin type 3 | p53 signaling pathway |  |  |
