# Supplementary material for: p53 modeling as a route to mesothelioma patients stratification and novel therapeutic identification
Source: J Transl Med. 2018 Oct 13;16:282. doi: 10.1186/s12967-018-1650-0 (PMC6186085; doi:10.1186/s12967-018-1650-0)
Supplement: Supplementary file 16 — Additional file 16: Table S15. Approved and experimental drugs that target PDGR1 indirectly (DRUGSURV database). [file 12967_2018_1650_MOESM16_ESM.docx]

**Table S15:** Approved and experimental drugs that target PDGR1 indirectly (DRUGSURV database)

| **PDRG1** |  |
| --- | --- |
| **Drugs (*approved*) which target PDRG1 indirectly** |  |
| **pdrg1->Interaction partner** | **Drugs which Target interaction partner of pdrg1** |
| pdrg1 -> HSP90AA1 | Nitroxoline |
|  | Clioquinol |
|  | Terconazole |
|  | Rifabutin |
|  | Miconazole |
|  | Nedocromil |
|  | Disulfiram |
|  | Hexachlorophene |
|  | Clotrimazole |
|  | Chloroxine |
|  | Fluconazole |
|  | Bifonazole |
| pdrg1 -> MAP3K3 | Desoximetasone |
|  | Erlotinib |
|  | Dasatinib |
|  | Gefitinib |
|  | Sunitinib |
|  | Norfloxacin |
|  | Ticlopidine |
|  | Aripiprazole |
