# Supplementary material for: p53 modeling as a route to mesothelioma patients stratification and novel therapeutic identification
Source: J Transl Med. 2018 Oct 13;16:282. doi: 10.1186/s12967-018-1650-0 (PMC6186085; doi:10.1186/s12967-018-1650-0)
Supplement: Supplementary file 17 — Additional file 17: Figure S2. TP53 protein is stabilized by DNA damage in Mero-14 cells. (A) Mero-14 cells were untreated or treated with etoposide (20 µM) for 24 h, after which cells were lysed and protein harvested and subjected to Western blotting using antibodies against TP53 and actin. (B) ImageJ quantification of TP53 expression in Mero-14 cells following etoposide treatment. (C) Sequence alignment of the coding sequence of the TP53 gene (exons 1–11) from Mero-14 cell line. [file 12967_2018_1650_MOESM17_ESM.pdf]

**Figure S2**

A

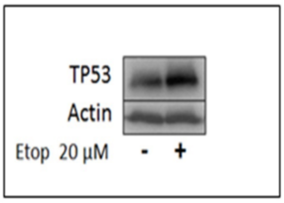


B

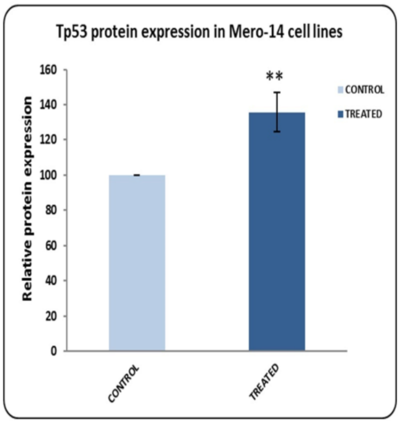


**P53 protein levels and stabilisation induced by DNA damage in Mero-14 cells A**. Detection of the Total p53 protein levels in the Mero-14 cell lines with and without DNA damage by using SDS PAGE and Western Blotting. The cell lines were incubated for 24 hrs with or without 20µM etoposide. **B.** Quantification of the wild-type p53 protein levels in the Mero-14 shown in Figure S2A, which was generated by using ImageJ software. Data is representative of three experiments; band intensity was normalised to β-actin.

*indicates $p<0.05$.

**(C)** Sequence alignment of the coding sequence of the TP53 gene (exons 1–11) from Mero-14 cell line, as described in Methods.

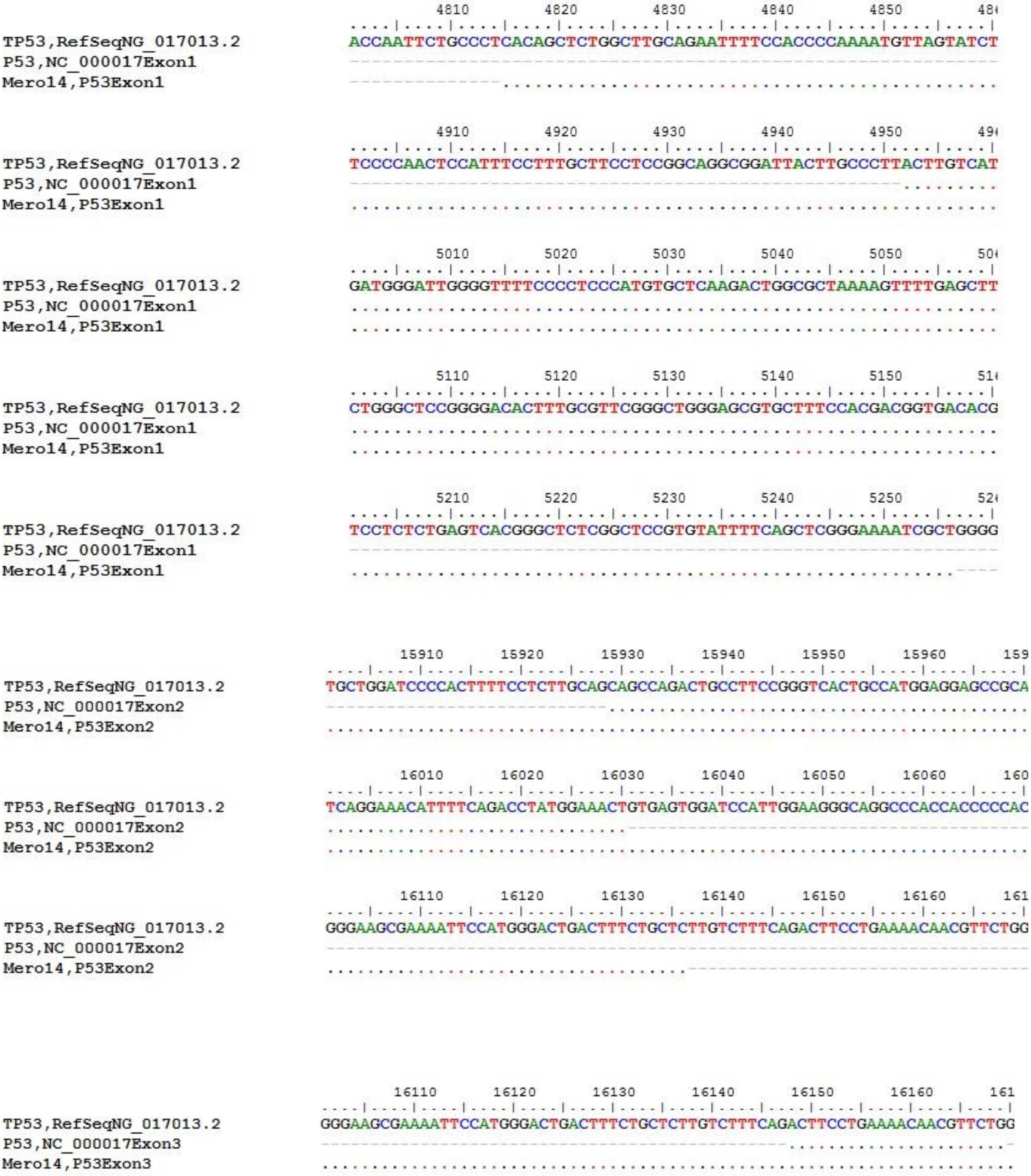

```
                                       16310     16320     16330     16340     16350
                             ....|....|....|....|....|....|....|....|....|....|...
TP53,RefSeqNG_017013.2       TGGATGATTTGATGCTGTCCCCGGACGATATTGAACAATGGTTCACTGAAGAC
P53,NC_000017Exon4           .....................................................
Mero14,P53Exon4              .....................................................

                                       16410     16420     16430     16440     16450
                             ....|....|....|....|....|....|....|....|....|....|...
TP53,RefSeqNG_017013.2       GGCCCCTGCACCAGCAGCTCCTACACCGGCGGCCCCTGCACCAGCCCCCTCCT
P53,NC_000017Exon4           .....................................................
Mero14,P53Exon4              .....................................................

                                       16510     16520     16530     16540     16550
                             ....|....|....|....|....|....|....|....|....|....|...
TP53,RefSeqNG_017013.2       TACGGTTTCCGTCTGGGCTTCTTGCATTCTGGGACAGCCAAGTCTGTGACTTG
P53,NC_000017Exon4           .....................................................
Mero14,P53Exon4              .....................................................

                                       16610     16620     16630     16640     16650
                             ....|....|....|....|....|....|....|....|....|....|...
TP53,RefSeqNG_017013.2       TGGCCGTATCCCCCTGCATTTCTTTTGTTTGGAACTTTGGGATTCCTCTTCAC
P53,NC_000017Exon4           -----------------------------------------------------
Mero14,P53Exon4              .....................................................


                                       17310     17320     17330     17340     17350
                             ....|....|....|....|....|....|....|....|....|....|...
TP53,RefSeqNG_017013.2       TCCTCTTCCTACAGTACTCCCCTGCCCTCAACAAGATGTTTTGCCAACTGGCC
P53,NC_000017Exon5,6         --------------.......................................
Mero14,P53Exon5,6            .....................................................

                                       17410     17420     17430     17440     17450
                             ....|....|....|....|....|....|....|....|....|....|...
TP53,RefSeqNG_017013.2       CACCCGCGTCCGCGCCATGGCCATCTACAAGCAGTCACAGCACATGACGGAGG
P53,NC_000017Exon5,6         .....................................................
Mero14,P53Exon5,6            .....................................................

                                       17510     17520     17530     17540     17550
                             ....|....|....|....|....|....|....|....|....|....|...
TP53,RefSeqNG_017013.2       GAGCAGCTGGGGCTGGAGAGACGACAGGGCTGGTTGCCCAGGGTCCCCAGGCC
P53,NC_000017Exon5,6         .....................................................
Mero14,P53Exon5,6            .....................................................

                                       17610     17620     17630     17640     17650
                             ....|....|....|....|....|....|....|....|....|....|...
TP53,RefSeqNG_017013.2       TTATCCGAGTGGAAGGAAATTTGCGTGTGGAGTATTTGGATGACAGAAACACT
P53,NC_000017Exon5,6         .....................................................
Mero14,P53Exon5,6            .....................................................

                                       17710     17720     17730     17740     17750
                             ....|....|....|....|....|....|....|....|....|....|...
TP53,RefSeqNG_017013.2       TGCAACTGGGGTCTCTGGGAGGAGGGGTTAAGGGTGGTTGTCAGTGGCCCTCC
P53,NC_000017Exon5,6         -----------------------------------------------------
Mero14,P53Exon5,6            ..............................................-------
```

```
                                  18210     18220     18230     18240     18250     18260     182
                         ....|....|....|....|....|....|....|....|....|....|....|....|....|....|
TP53,RefSeqNG_017013.2   GCTTGCCACAGGTCTCCCCAAGGCGCACTGGCCTCATCTTGGGCCTGTGTTATCTCCTAGGTTGGCTCTG
P53,NC_000017Exon7       ------------------------------------------------------------..........
Mero14,P53Exon7          ......................................................................

                                  18310     18320     18330     18340     18350     18360     183
                         ....|....|....|....|....|....|....|....|....|....|....|....|....|....|
TP53,RefSeqNG_017013.2   GTAACAGTTCCTGCATGGGCGGCATGAACCGGAGGCCCATCCTCACCATCATCACACTGGAAGACTCCAG
P53,NC_000017Exon7       ......................................................................
Mero14,P53Exon7          ......................................................................

                                  18410     18420     18430     18440     18450     18460     184
                         ....|....|....|....|....|....|....|....|....|....|....|....|....|....|
TP53,RefSeqNG_017013.2   GCCTGCTGTGCCCCAGCCTCTGCTTGCCTCTGACCCCTGGGCCCACCTCTTACCGATTTCTTCCATACTA
P53,NC_000017Exon7       ----------------------------------------------------------------------
Mero14,P53Exon7          ......................................................................
```

```
                                  18710     18720     18730     18740     18750     187
                         ....|....|....|....|....|....|....|....|....|....|....|....|
TP53,RefSeqNG_017013.2   CTATCCTGAGTAGTGGTAATCTACTGGGACGGAACAGCTTTGAGGTGCGTGTTTGTGCCT
P53,NC_000017Exon8,9     -------------...............................................
Mero14,P53Exon8,9        ............................................................

                                  18810     18820     18830     18840     18850     188
                         ....|....|....|....|....|....|....|....|....|....|....|....|
TP53,RefSeqNG_017013.2   CAAGAAAGGGGAGCCTCACCACGAGCTGCCCCCAGGGAGCACTAAGCGAGGTAAGCAAGC
P53,NC_000017Exon8,9     ............................................................
Mero14,P53Exon8,9        ............................................................

                                  18910     18920     18930     18940     18950     189
                         ....|....|....|....|....|....|....|....|....|....|....|....|
TP53,RefSeqNG_017013.2   CCTCAGATTCACTTTTATCACCTTTCCTTGCCTCTTTCCTAGCACTGCCCAACAACACCA
P53,NC_000017Exon8,9     ............................................................
Mero14,P53Exon8,9        ............................................................

                                  19010     19020     19030     19040     19050     190
                         ....|....|....|....|....|....|....|....|....|....|....|....|
TP53,RefSeqNG_017013.2   ATATTTCACCCTTCAGGTACTAAGTCTTGGGACCTCTTATCAAGTGGAAAGTTTCCAGTC
P53,NC_000017Exon8,9     ................--------------------------------------------
Mero14,P53Exon8,9        ............................................................
```

```
                                  21810     21820     21830     21840     21850     21860     218
                         ....|....|....|....|....|....|....|....|....|....|....|....|....|....|
TP53,RefSeqNG_017013.2   ATATACTTACTTCTCCCCCTCCTCTGTTGCTGCAGATCCGTGGGCGTGAGCGCTTCGAGATGTTCCGAGA
P53,NC_000017Exon10      -----------------------------------...................................
Mero14,P53Exon10         ......................................................................

                                  21910     21920     21930     21940     21950     21960     219
                         ....|....|....|....|....|....|....|....|....|....|....|....|....|....|
TP53,RefSeqNG_017013.2   CCAGGCTGGGAAGGAGCCAGGGGGGAGCAGGGCTCACTCCAGGTGAGTGACCTCAGCCCCTTCCTGGCCC
P53,NC_000017Exon10      ..........................................----------------------------
Mero14,P53Exon10         ......................................................................

                                  22010     22020     22030     22040     22050     22060     220
                         ....|....|....|....|....|....|....|....|....|....|....|....|....|....|
TP53,RefSeqNG_017013.2   ATAGGATTCCATTCTCATCCTGCCTTCATGGTCAAAGGCAGCTGACCCCATCTCATTGGGTCCCAGCCCT
P53,NC_000017Exon10      ----------------------------------------------------------------------
Mero14,P53Exon10         ......................................................................
```

```
                                   22710     22720     22730     22740     22750     22760     227
                            ----|----|----|----|----|----|----|----|----|----|----|----|----|----|
TP53,RefSeqNG_017013.2      GGTGCTCAGTAAACATATTTGCATGGGGTGTGGCCACCATCTTGATTTGAATTCCCGTTGTCCCAGCCTT
P53,NC_000017Exon11         ----------------------------------------------------------------------
Mero14,P53Exon11            ----------------------------------------------------..................

                                   22810     22820     22830     22840     22850     22860     228
                            ----|----|----|----|----|----|----|----|----|----|----|----|----|----|
TP53,RefSeqNG_017013.2      GGGCACAGACCCTCTCACTCATGTGATGTCATCTCTCCTCCCTGCTTCTGTCTCCTACAGCCACCTGAAG
P53,NC_000017Exon11         ------------------------------------------------------------..........
Mero14,P53Exon11            ......................................................................

                                   22910     22920     22930     22940     22950     22960     229
                            ----|----|----|----|----|----|----|----|----|----|----|----|----|----|
TP53,RefSeqNG_017013.2      AAAAAACTCATGTTCAAGACAGAAGGGCCTGACTCAGACTGACATTCTCCACTTCTTGTTCCCCACTGAC
P53,NC_000017Exon11         ......................................................................
Mero14,P53Exon11            ......................................................................

                                   23010     23020     23030     23040     23050     23060     230
                            ----|----|----|----|----|----|----|----|----|----|----|----|----|----|
TP53,RefSeqNG_017013.2      CCATTTTGGGTTTTGGGTCTTTGAACCCTTGCTTGCAATAGGTGTGCGTCAGAAGCACCCAGGACTTCCA
P53,NC_000017Exon11         ......................................................................
Mero14,P53Exon11            ......................................................................

                                   23110     23120     23130     23140     23150     23160     231
                            ----|----|----|----|----|----|----|----|----|----|----|----|----|----|
TP53,RefSeqNG_017013.2      GTTGGCCTGCACTGGTGTTTTGTTGTGGGGAGGAGGATGGGGAGTAGGACATACCAGCTTAGATTTTAAG
P53,NC_000017Exon11         ......................................................................
Mero14,P53Exon11            ......................................................................

                                   23210     23220     23230     23240     23250     23260     232
                            ----|----|----|----|----|----|----|----|----|----|----|----|----|----|
TP53,RefSeqNG_017013.2      TAAGAAATGTTCTTGCAGTTAAGGGTTAGTTTACAATCAGCCACATTCTAGGTAGGGGCCCACTTCACCG
P53,NC_000017Exon11         ......................................................................
Mero14,P53Exon11            ............................------------------------------------------
```
