# Supplementary figures and images for: p53 modeling as a route to mesothelioma patients stratification and novel therapeutic identification
Source: J Transl Med. 2018 Oct 13;16:282. doi: 10.1186/s12967-018-1650-0 (PMC6186085; doi:10.1186/s12967-018-1650-0)

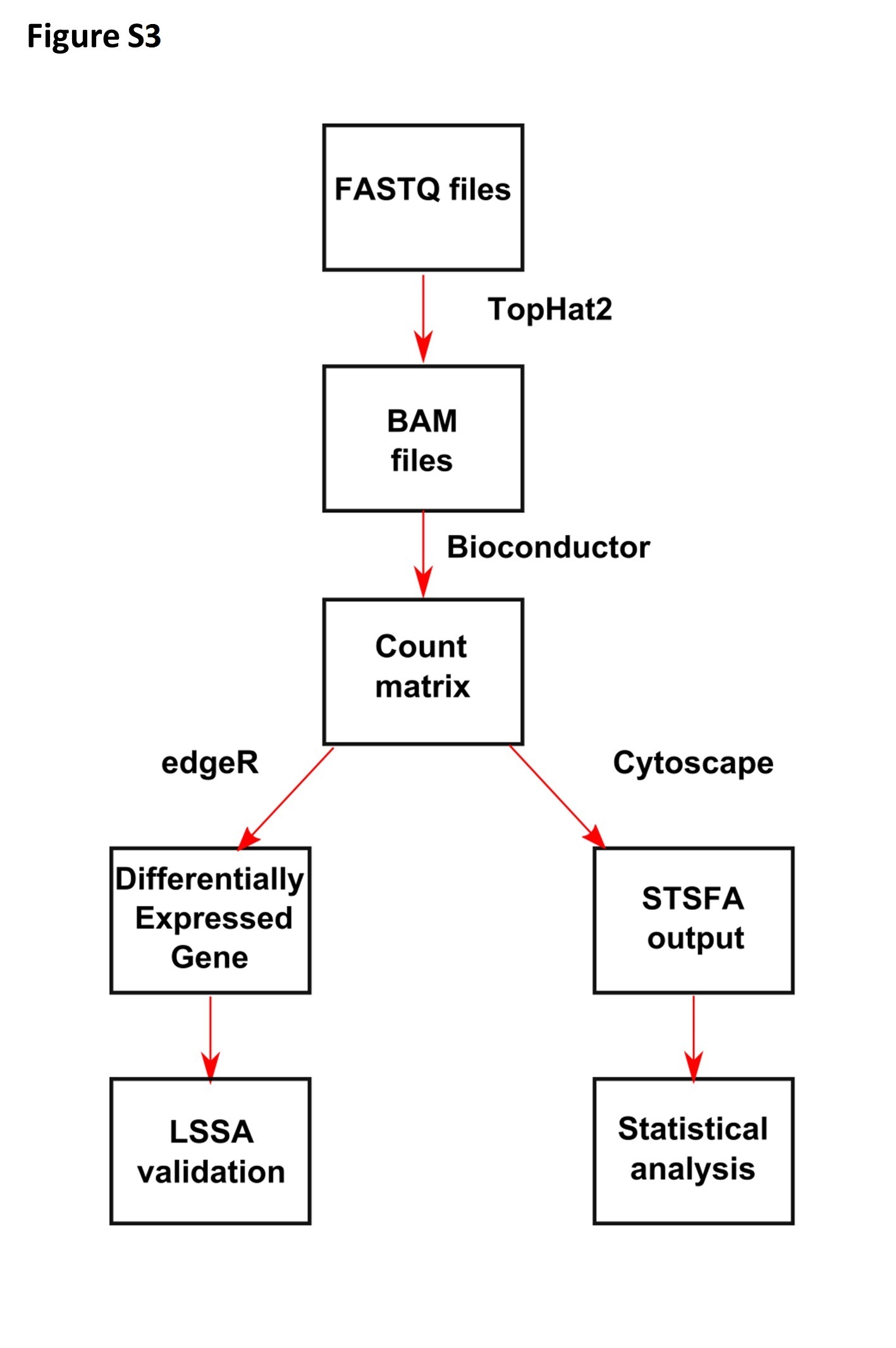

Supplement: Supplementary file 18 — Additional file 18: Figure S3. The schematic workflow of the RNA sequencing analysis for the patient’s data. The schematic diagram depicts the workflow for the RNA sequencing analysis of the patients data. The sequencing data in the format of FASTQ file are aligned by the TopHat2 to generate the input BAM files. The BAM files are processed to obtain the count matrix for the differential expression analysis and the statistical analysis based on the STSFA score of each gene. Differentially expressed genes are identified by R script based on the edgeR packages and utilized to validate the LSSA predictions. The STSFA score of each gene in the model are calculated by the Cytoscape platform and processed for the further statistical analysis. [file 12967_2018_1650_MOESM18_ESM.jpg]
